# Inflammatory myofibroblastic tumors of the colon in pediatrics: clinical presentation, management, and outcomes—A case report and systematic review of literature

**DOI:** 10.1007/s00384-025-04869-y

**Published:** 2025-04-15

**Authors:** Ismael Elhalaby, Omar Koura, Rofyda Elhalaby, Wael Zeina, Mohamed Shareef, Essam Elhalaby

**Affiliations:** 1 Department of Surgery, Faculty of Medicine, Tanta University, Tanta, Egypt; 2 Kenanah Children’s Medical Center, Tanta, Egypt; 3 Department of Pathology, Faculty of Medicine, Tanta University, Tanta, Egypt; 4 Benha Children Hospital, Benha, Egypt

**Keywords:** Inflammatory myofibroblastic tumor, Pediatric, IMT, Colorectal, Neoplasm, Case report

## Abstract

**Purpose:**

Inflammatory myofibroblastic tumors (IMTs) of the colon represent an exceptionally rare entity in the pediatric population. This systematic review aims to comprehensively analyze the clinical presentation, diagnostic workup, management strategies, and outcomes of colorectal IMTs in children.

**Methods:**

A systematic literature review was conducted across multiple electronic databases (inception to January 2025), including MEDLINE (via PubMed), Embase, Cochrane, Web of Science, and Google Scholar. Two independent reviewers screened abstracts, reviewed studies, and extracted data on all reported cases of colorectal IMTs in the pediatric population, including one previously unreported case from our institution.

**Results:**

Including our case, 53 pediatric patients with colorectal IMTs were identified from 39 studies. The mean age at diagnosis was 7 years (range: 5 months-17 years) with a slight female preponderance. The IMTs comprised a wide range of anatomic locations with rectum (27%) and ascending colon (24%) being the most common. Abdominal pain (54%), gastrointestinal bleeding (29%), and fever (21%) were the predominant symptoms. Anemia was the most common laboratory abnormality (62%). Surgical resection was the primary treatment modality in 98% of cases. After a mean follow-up of 38 months (

range: 3–181 months), the local recurrence rate was 11%, with no distant metastases reported.

**Conclusion:**

Colorectal IMTs in children present diagnostic and therapeutic challenges. While complete surgical resection remains the gold standard treatment, emerging therapies such as ALK inhibitors and NSAIDs warrant further investigation. The potential for late recurrence mandates long term follow-up.

**Supplementary Information:**

The online version contains supplementary material available at 10.1007/s00384-025-04869-y.

## Introduction

Inflammatory myofibroblastic tumors (IMTs), also known as plasma cell granulomas or inflammatory pseudotumors (IPT), are rare neoplasms characterized by the proliferation of myofibroblasts accompanied by inflammatory infiltration. While the lungs are considered the most frequently involved site, IMTs can arise in various locations, including lymph nodes, spleen, liver, mediastinum, diaphragm, and mesentery [1]. The exact etiology of IMTs remains elusive, with several factors potentially playing a role, including trauma, surgery, infections, radiation, steroid use, and autoimmune diseases [2].

Colorectal IMTs in the pediatric population represent an exceptionally rare subset of these tumors, with limited data available in the literature [1, 3, 4]. The scarcity of reported cases, predominantly presented as isolated case reports, has hindered the development of a comprehensive understanding of their clinical behavior, optimal management strategies, and long-term outcomes in children.

This study aims to synthesize the available evidence on clinical presentation, diagnostic approaches, treatment modalities, and outcomes of these rare tumors by conducting a systematic review of colorectal IMTs in pediatric patients. Additionally, we present a case of colonic IMT managed at our institution, which has been integrated into this systematic review.

## Methods

### Search Strategy

A comprehensive literature search was conducted across multiple electronic databases, including MEDLINE (via PubMed), Embase, Cochrane, Web of Science, and Google Scholar, from inception to January 2025. The search strategy employed a combination of Medical Subject Headings (MeSH) terms and free-text keywords, including but not limited to "inflammatory myofibroblastic tumor," "inflammatory pseudotumor," "colon," "rectum," "colorectal," "pediatric," and "children". A broad search approach was implemented to capture all potentially relevant reports. The detailed search strategy implemented during the PubMed search and retrieved articles have been provided in supplementary files 1 and 2. Reference lists of included studies were manually examined to identify additional pertinent articles. The search process adhered to the Preferred Reporting Items for Systematic Reviews and Meta-Analyses (PRISMA) statement [5] (supplementary file 3).

### Inclusion/Exclusion criteria

Studies were included if they met the following criteria: (1) participants ≤ 18 years of age; (2) confirmed diagnosis of inflammatory myofibroblastic tumors originating from colonic or rectal tissue; (3) detailed management strategies and follow-up. All study designs were considered eligible. Exclusion criteria encompassed non-English language publications, lack of detailed management and outcome data, and tumors not arising from the colon or rectum. Review articles, editorials, and abstracts were excluded unless they provided sufficient information to meet the inclusion criteria.

### Data extraction and analysis

Two reviewers (IE, OK) screened titles and abstracts independently using Rayyan (https://www.rayyan.ai) followed by full-text assessment for eligibility. After the inclusion of the relevant studies, data extraction was performed independently using a standardized form that captured key information from each included study, encompassing author(s), country, year of publication, patient demographics, preoperative diagnostic workup, management strategies, and outcomes including recurrence data. Discrepancies in data extraction were resolved through consensus or consultation with a third reviewer (EE) when necessary. One additional case from the authors' institution was included, with data collected according to the CARE checklist (Supplementary file 4). Descriptive statistics were used to analyse the data, reporting continuous variables as mean and range, and categorical variables as percentages. Cases were systematically grouped based on demographics, tumor location, preoperative management, treatment modality, and outcomes to facilitate meaningful synthesis.

### Risk of bias assessment and reporting

While a formal risk of bias assessment and reporting was initially considered, the nature of the included studies—predominantly case reports and small series—rendered traditional assessment tools inapplicable. Nevertheless, we critically evaluated the completeness of reporting in each study to ensure a robust synthesis of the available evidence.

### Certainty assessment

The certainty of evidence for key findings was assessed using a modified GRADE (Grading of Recommendations, Assessment, Development, and Evaluations) approach, adapted for case series and reports. We considered factors such as study design, risk of bias, consistency of results across studies, and precision of estimates.

## Results

### Search results

**Fig. 1:**
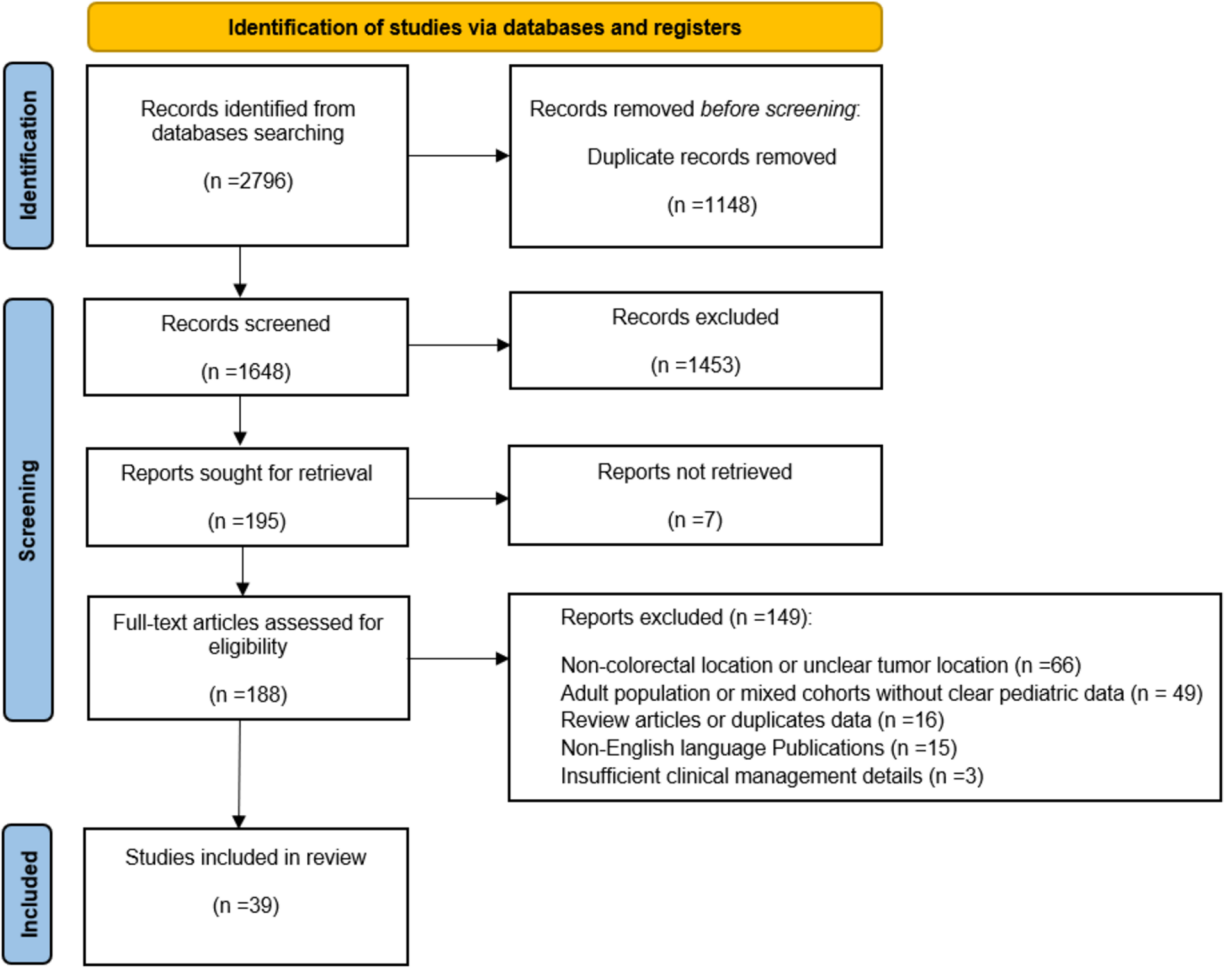
Selection of the relevant studies using the Preferred Reporting Items for Systematic Review and Meta-Analysis (PRISMA) flow diagram

Our systematic review identified 2796 records through the initial search strategy. After removing 1148 duplicates, 1648 records remained for screening. Title and abstract screening resulted in the exclusion of 1,453 records, leaving 195 articles for full-text assessment. Of these, 7 records could not be retrieved, and 149 failed to meet the inclusion criteria and were excluded. Finally, a total of 39 articles were included in the final analysis, comprising 23 case reports, 18 case series, and 12 retrospective cohort studies. A detailed PRISMA flow diagram illustrating the search results and selection process is presented in Fig. 1.

### Certainty of evidence

The certainty of evidence for key findings was generally assessed as low to very low, primarily due to the observational nature of the included studies, small sample sizes, and potential for reporting bias. However, the consistency of findings across multiple case reports and series, particularly regarding common presenting symptoms and the effectiveness of surgical management, provides some confidence in these key results.

### Details of our case

**Fig. 2:**
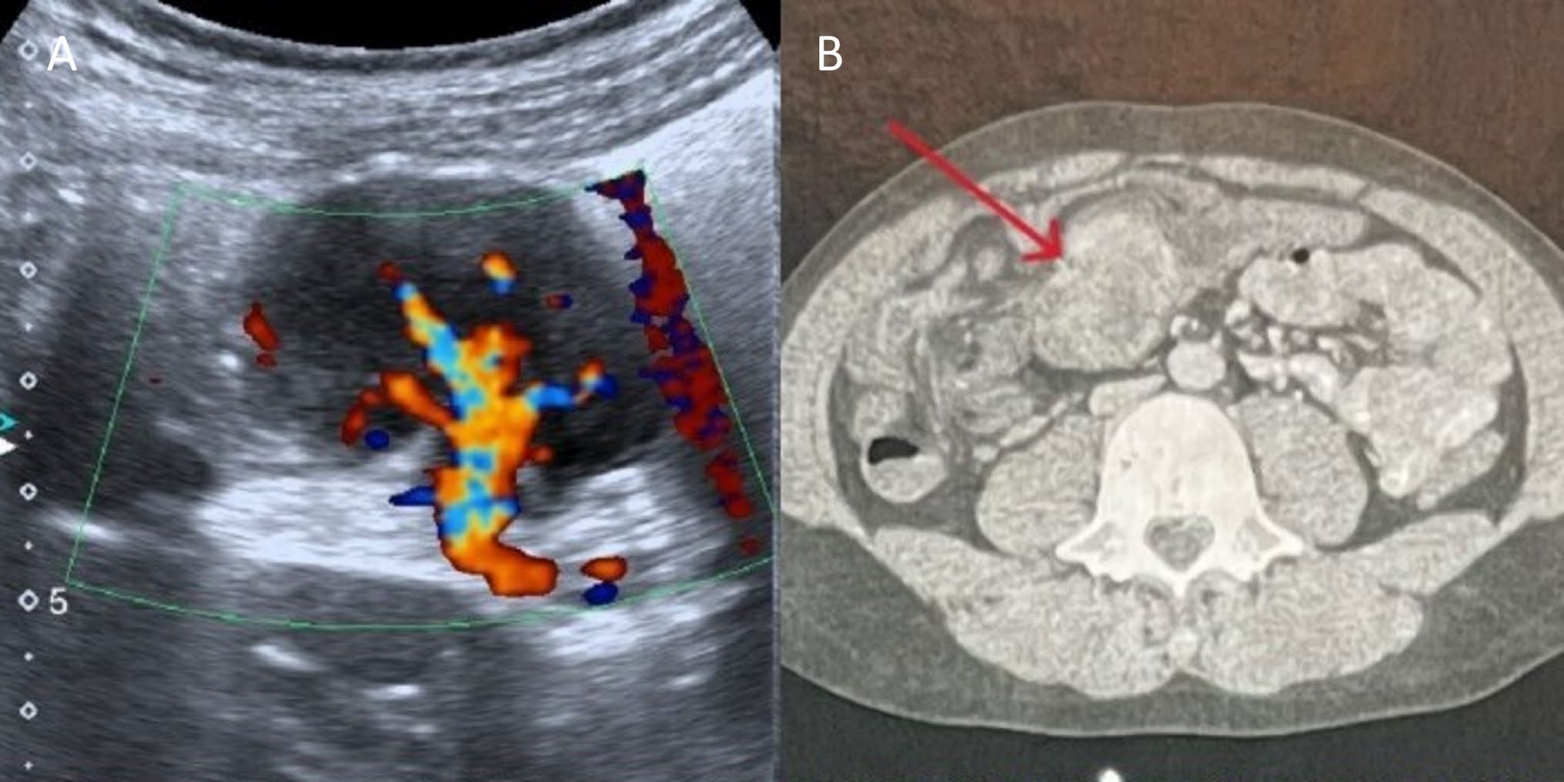
A Abdominal ultrasound view of the tumor showing a highly vascular central pedicle. B Tumor localized at the ascending colon (arrow)

A 9-year-old male presented with a 4-month history of recurrent abdominal pain previously managed conservatively. Physical examination was unremarkable, and laboratory studies revealed only anemia. Abdominal ultrasonography demonstrated a 4.5 × 3.8 × 3 cm hypoechoic lesion in the right hypochondrium with a hypervascular central pedicle (Fig. 2A). Subsequent computed tomography revealed a well-defined, 4.8 × 3.2 cm soft tissue mass within the right colon, exhibiting marked internal vascularity and adherence to surrounding structures (Fig. 2B).

Following family consultation, the patient underwent a diagnostic laparoscopy, which revealed a mass lesion involving the ascending colon without evidence of intussusception. The umbilical port was surgically extended for mass retrieval and right hemicolectomy was performed with ileo-transverse anastomosis. The patient’s postoperative course was uneventful.

**Fig. 3:**
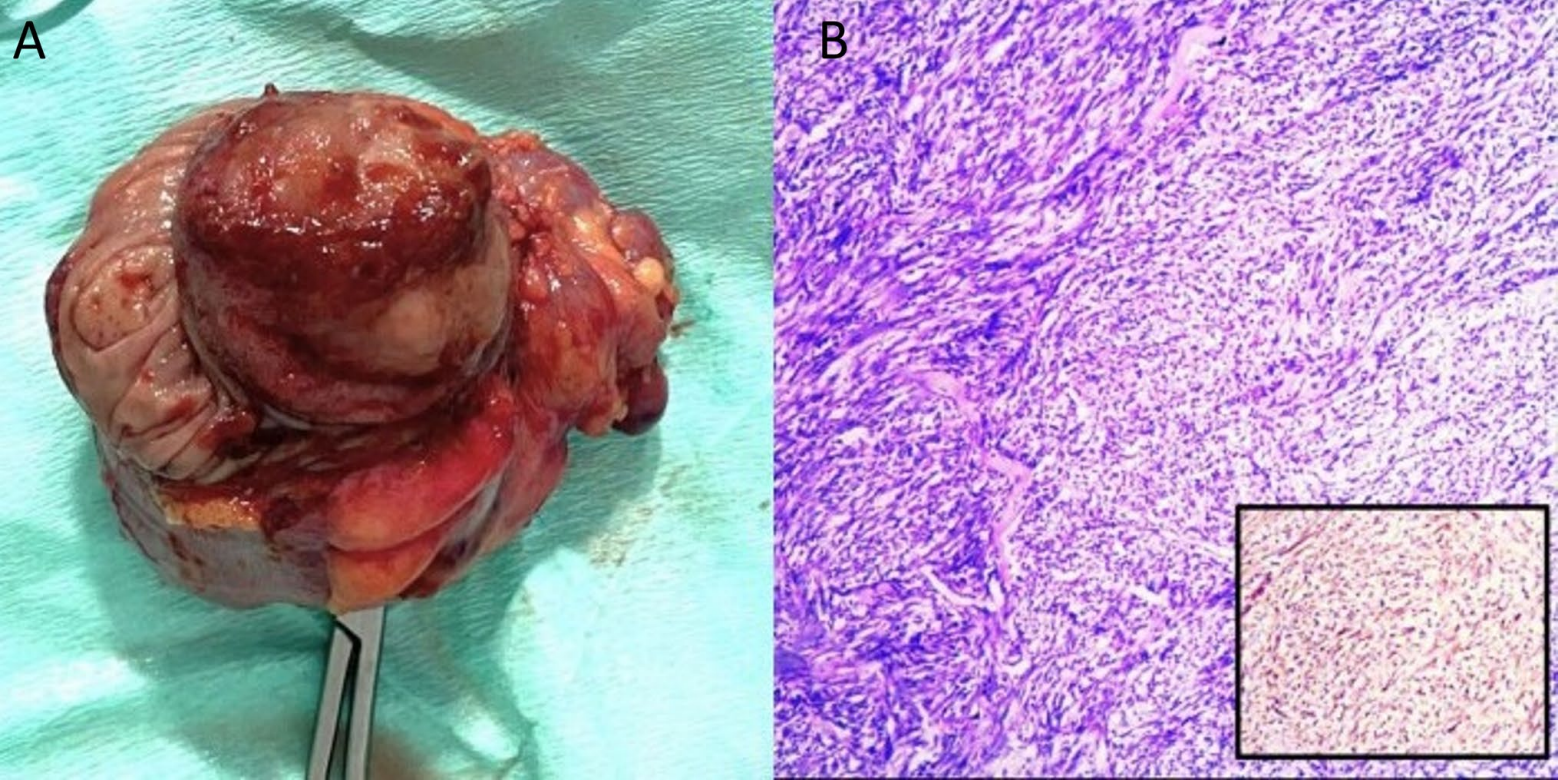
A Gross appearance of the resected specimen. B H&E-stained section shows neoplastic spindle cell proliferation infiltrated by inflammatory cells, inset shows focal smooth muscle actin positivity by immunohistochemistry (× 100)

Gross pathological examination revealed a 4.5 × 4 × 3.5 cm polypoid mass with an ulcerated surface (Fig. 3A). Serial sections revealed a hard, translucent cut surface invading the muscularis propria. Microscopic examination demonstrated a spindle cell tumor formed of moderately atypical cells arranged in a fascicular pattern with alternating hypercellular areas and loose myxoid foci admixed with inflammatory cells. All surgical margins (proximal, distal, and radial) were negative, and dissected pericolic lymph nodes were tumor-free (0/29). Immunohistochemical studies showed positive staining for smooth muscle actin (SMA) (Fig. 3B), consistent with myofibroblastic lineage, while ALK-1, DOGl, C-kit, and S100 stains were all negative. These findings established the diagnosis of IMT. The patient has remained asymptomatic with no evidence of recurrence on ultrasound imaging during two years of follow-up and no adjuvant treatment was required. Informed consent was obtained to share the intervention details.

### Patient characteristics and demographics

The review encompassed 53 patients, including our case, from 39 published English-language articles [6–44]. Geographic distribution revealed a predominance of cases from Asian (n = 26, 49%) and European (n = 17, 32%) countries. A slight female preponderance was noted (26 females versus 25 males), with two reports lacking gender specification. The mean age at diagnosis was 7 years (range: 3 months-17 years), with no significant gender-based differences in age distribution. The studies and patient characteristics are depicted in Table 1.

**Table 1:** Summary of demographic and clinical characteristics of reported patients with colorectal IMT in the literature

Patient no	Authors, year (reference no.)	Country	Age (years), Sex	Location	Presentation	Significant laboratory0 results	Preoperative biopsy	Intervention	Follow-up (months)	Recurrence
1	Biselli et al., 1996 [6]	Italy	11, M	N/S	N/S	N/S	N/S	N/S	34	Recurrence; subsequent management undocumented
2	Biselli et al., 1996 [6]	Italy	2, F	N/S	N/S	N/S	N/S	N/S	25	No
3	Jimenez et al., 1997 [7]	USA	13, F	Rectum	Abdominal pain, hematochezia, constipation, weight loss	N/S	Transanal biopsy: confirmed IMT	Laparotomy and segmental resection with rectal and vaginal reconstruction and proximal diverting colostomy	42	No
4	Cviko et al., 1999 [8]	Croatia	7, F	Cecum	Abdominal pain, distention	Normal	No	Laparotomy and right hemicolectomy	24	No
5	Velitchkov et al., 2000 [9]	Bulgaria	3, F	Transverse	Incidental intraoperative finding (hepatic hydatid cyst)	Normal	No	Laparotomy and segmental resection	6	No
6	Karnak et al., 2001 [10]	Turkey	11, M	Descending	Weight loss, anorexia	N/S	No	Laparotomy and segmental resection	N/S	No
7	Sanders et al., 2001 [11]	USA	11, F	Sigmoid	Abdominal pain, distension, fever, intestinal obstruction	Normal	No	Initial surgery-drainage of pelvic abscess. Second surgery at 2 months- appendectomy and omentectomy. Third surgery at 4 months-Laparotomy and segmental resection with end colostomy and Hartmann's pouch	72	Multiple recurrences; 2 mo (appendix and omentum, reoperation), 4 mo (sigmoid, reoperation), 10 mo (right colon, anti-inflammatory therapy). NED at 6 yr follow-up
8	Sanders et al., 2001 [11]	USA	15, F	Rectum	Abdominal pain, nausea, diarrhea, weight loss	Anemia, ↑ESR, + CRP	Colonoscopy biopsy: inflammatory reaction	Transanal resection	12	No
9	Lykavieris et al., 2003 [12]	France	9 mo, F	Ascending	Abdominal pain, distension, fever, vomiting, intestinal obstruction	Anemia	No	Laparotomy and right hemicolectomy with proximal diverting ileostomy	26	No
10	Katara et al., 2004 [13]	India	16, F	Ascending	Abdominal mass, fever of unknown origin	↑ESR	CT guided FNAC: inconclusive	Laparotomy and right hemicolectomy	N/S	No
11	Mergan et al., 2005 [14]	France	6, F	Rectum	Hematochezia	N/S	N/S	Laparotomy and partial resection	72	Recurrence at 2 mo; reoperation. NED at 6 yr follow-up
12	Chun et al., 2005 [15]	USA	1, F	Hepatic flexure	Abdominal pain, abdominal mass, melena, fever	Anemia, Thrombocytosis	No	Laparotomy and segmental resection	12	No
13	Khoddami et al., 2006 [16]	Iran	11, M	Rectum	Abdominal pain, bloody diarrhea, fecal incontinence, fatigue, weight loss	Anemia, thrombocytosis, ↑ESR, + CRP	No	Laparotomy and segmental resection	36	No
14	Saleem et al., 2007 [17]	UK	9, F	Cecum	Abdominal pain, lethargy, chest pain, fever, weight loss	Anemia, Thrombocytosis, ↑ ESR, + CRP	FNAC: confirmed IMT	A course of NSAIDs failed to achieve regression of the tumor followed by laparotomy and right hemicolectomy	N/S	No
15	Shi et al., 2010 [18]	China	16, M	Ascending	Abdominal mass	N/S	No	Laparotomy and right hemicolectomy	60	No
16	Shi et al., 2010 [18]	China	15, F	Descending	Abdominal pain, abdominal mass	N/S	No	Laparotomy and segmental resection	48	No
17	Fragoso et al., 2011 [19]	Portugal	4, M	N/S	Abdominal pain, intestinal obstruction	N/S	No	Laparotomy and segmental resection	60	No
18	Fragoso et al., 2011 [19]	Portugal	12, M	N/S	Intestinal obstruction	N/S	No	Laparotomy and segmental resection	36	No
19	Mirshemirani et al., 2011 [20]	Iran	12, M	Splenic flexure	Abdominal pain, abdominal mass, vomiting	N/S	No	Laparotomy and segmental resection	78	Recurrence at 18 mo (abdominal wall mass); reoperation + chemotherapy. NED at 5 yr follow-up
20	Salameh et al., 2011 [21]	Jordan	32 mo, M	Transverse	Abdominal pain, abdominal mass, distention, chronic constipation, hematochezia	Anemia	No	Laparotomy and segmental resection	N/S	N/S
21	Zhou et al., 2011 [22]	China	1, F	Rectum	Rectal mass	Anemia, Leukocytosis, ↑ESR, + CRP	No	Transanal resection	54	No
22	Ntloko et al., 2011 [23]	South Africa	3, M	Ascending	Incidental intraoperative finding (exploration for abdominal trauma)	N/S	No	Laparotomy and hemicolectomy	18	No
23	Shahnoor et al., 2012 [24]	Bangladesh	10, F	Descending	Abdominal mass, lethargy, pallor, anorexia, fever, constipation, weight loss	Anemia, Thrombocytosis, ↑ESR	U/S guided FNAC: few round oval cells, spindle cells, no malignancy	Laparotomy and segmental resection	12	No
24	Satahoo et al., 2013 [25]	Bahamas	14, M	Rectum	Rectal mass, tenesmus, bloody diarrhea, constipation, weight loss	Leukocytosis, ↑ESR, + CRP	Colonoscopy biopsy: chronic inflammation; Transanal biopsy: fibroinflammatory changes, ischemic necrosis	Laparotomy and sigmoid colostomy to relieve obstruction followed by tumor resolution after a six-week course of high-dose oral NSAID therapy	84	No
25	Appak et al., 2014 [26]	Turkey	7, F	Ascending	Abdominal pain, abdominal mass	Anemia, Thrombocytosis, ↑ESR, + CRP	No	Laparotomy and right hemicolectomy	12	No
26	Walia et al., 2014 [27]	USA	10, F	Ascending	Abdominal pain, bloody diarrhea, weight loss, intestinal obstruction	Anemia	No	Laparotomy and right hemicolectomy	N/S	N/S
27	Buccoliero et al., 2014 [28]	Italy	9 m, M	Transverse	Abdominal mass	Normal	No	Laparotomy and segmental resection	N/S	N/S
28	Oguz et al., 2015 [29]	Turkey	3, M	Rectum	Hematochezia	N/S	No	Laparotomy and segmental resection	N/S	No
29	Höhne et al., 2015 [30]	Germany	9, M	Transverse	Abdominal pain, fever	Anemia, Leukocytosis, Thrombocytosis, ↑ESR, + CRP	No	Laparoscopic exploration converted to laparotomy and segmental resection	N/S	N/S
30	Dalton et al., 2015 [31]	USA	6, N/S	N/S	Feeding intolerance	N/S	N/S	Laparotomy and segmental resection	34	No
31	Sherman et al.,2015 [32]	USA	12, F	Descending	Abdominal pain, constipation interspersed with bloody diarrhea, weight loss	Normal	Colonoscopy biopsy: normal mucosa, rare spindle cells	Laparoscopic-assisted segmental resection	11	No
32	Yamamoto et al., 2016 [33]	China	4, F	Rectum	N/S	N/S	N/S	N/S	16	Recurrence at 2 mo; subsequent management undocumented
33	Yamamoto et al., 2016 [33]	China	1, M	Cecum	N/S	N/S	N/S	N/S	181	No
34	Yu et al., 2016 [34]	China	15, F	Transverse	Abdominal pain, abdominal mass	N/S	N/S	Laparotomy and segmental resection	7	No
35	Soyer et al., 2017 [35]	Turkey	3, M	Rectum	Hematochezia, rectal mass	N/S	Tru-cut biopsy: confirmed IMT	Laparotomy and segmental resection	N/S	N/S
36	Soyer et al., 2017 [35]	Turkey	3, M	N/S	Abdominal mass, vomiting	N/S	Tru-cut biopsy: confirmed IMT	Laparotomy and segmental resection	36	No
37	Qian et al., 2019 [36]	China	4, M	Ascending	N/S	N/S	No	Laparotomy and right hemicolectomy	3	No
38	Qian et al., 2019 [36]	China	4, F	Ascending	Abdominal mass	N/S	No	Laparotomy and right hemicolectomy	6	No
39	Qian et al., 2019 [36]	China	4, F	N/S	Abdominal pain	N/S	No	Laparotomy and resection followed by chemotherapy (regimen not specified)	36	No
40	Qian et al., 2019 [36]	China	5, M	N/S	Abdominal pain	N/S	No	Laparotomy and resection	8	No
41	Sharma et al., 2020 [37]	India	7, M	Ascending	Abdominal pain, fever, vomiting, intestinal obstruction	Anemia, Neutropenia	No	Laparotomy and right hemicolectomy	N/S	Died during same admission due to worsening sepsis
42	Garnier et al., 2021[38]	Poland	5 m, N/S	Cecum	Abdominal distention, hematochezia	+ CRP	No	Laparotomy and right hemicolectomy followed by HIPEC procedure	12	No
43	Da et al., 2021 [39]	China	3, F	N/S	Abdominal pain	N/S	No	Laparotomy and resection	131	No
44	Da et al., 2021 [39]	China	6 mo, M	N/S	Hematochezia	N/S	No	Laparotomy and resection	N/S	N/S
45	Da et al., 2021 [39]	China	20 mo, F	N/S	Abdominal pain	N/S	No	Laparotomy and resection	69	No
46	Da et al., 2021 [39]	China	8, M	Rectum	Hematochezia	N/S	No	Laparotomy and resection	N/S	N/S
47	Da et al., 2021 [39]	China	5, M	N/S	Abdominal pain, hematochezia	N/S	No	Laparotomy and resection	40	No
48	Narihiro et al., 2022 [40]	Japan	17, F	Rectum	Fever, diarrhea, constipation, proctalgia	Anemia, ↑ESR	Endoscopic U/S guided FNAC: inconclusive; CT guided biopsy: confirmed IMT	Laparoscopic low anterior resection with transanal total mesorectal excision and proximal diverting ileostomy	N/S	N/S
49	Kavirayani et al., 2023 [41]	India	8 mo, F	Sigmoid	Abdominal mass	Anemia, Leukocytosis	U/S guided core biopsy: spindle cell neoplasm with inflammatory infiltrate	Laparotomy and segmental resection	6	No
50	Wu et al., 2023 [42]	China	11 mo, M	Sigmoid	Abdominal mass, fever, vomiting	Anemia	No	Laparotomy and segmental resection	24	No
51	Khibiani et al., 2023[43]	Iran	10, M	Sigmoid	Abdominal pain, abdominal distention, constipation, vomiting, intestinal obstruction, shock	Leucocytosis, ↑ BUN, acidosis	No	Laparotomy and segmental resection with end colostomy and Hartmann's pouch	N/S	N/S
52	Hu et al., 2024 [44]	China	10, F	Sigmoid	Abdominal pain, abdominal mass	Normal	No	Laparotomy and radical resection with proximal diverting ileostomy	6	No
53	Our Case	Egypt	9, M	Ascending	Abdominal pain	Anemia	No	Laparoscopic-assisted right hemicolectomy	24	No

^*HIPEC*Hyperthermic intraperitoneal chemotherapy*, IMT*inflammatory myofibroblastic tumor, *FNAC*fine needle aspiration cytology, *N/S*not specified, *NED*no evidence of disease, *NSAID*nonsteroidal anti−inflammatory drug, *U/S*ultrasound^

### Tumor location

**Fig. 4:**
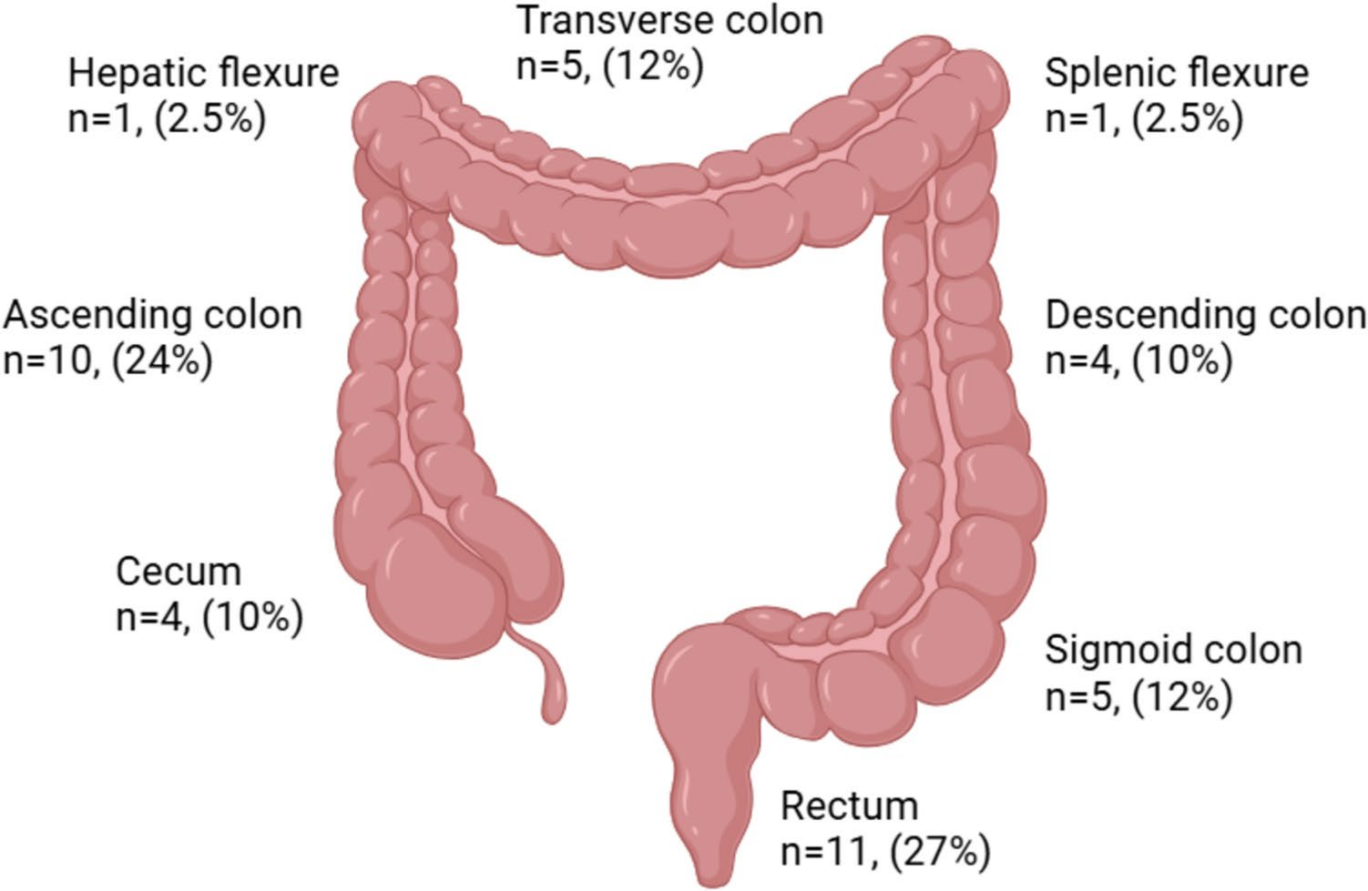
Distribution of cases by location (n = 41). Twelve additional cases (23% of total) had unspecified locations and are not included in this distribution

Tumor location was specified in 48 (77%) cases. The rectum was the most frequent site (n = 11, 27%), followed by the ascending colon (n = 10, 24%), transverse colon (n = 5, 12%), sigmoid colon (n = 5, 12%), descending colon (n = 4, 10%), cecum (n = 4, 10%), hepatic flexure (n = 1, 2.5%), and splenic flexure (n = 1, 2.5%). Figure 4 illustrates the distribution of cases by colonic location.

### Clinical presentation

Clinical presentation data were available for 48 (91%) patients. Predominant symptoms included abdominal pain (54%), gastrointestinal bleeding (melena, bloody diarrhea, or haematochezia) (27%), fever (24%), and weight loss (19%). Interestingly, IMT was an incidental intraoperative finding in two cases [7, 23]. Physical examination revealed an abdominal mass in 14 (29%) patients, while 3 of 11 patients with rectal IMT presented with a rectal mass. Intestinal obstruction was the presenting symptom in 7 patients (15%) and intussusception was reported in 7 patients (15%) on preoperative imaging or intraoperatively.

### Preoperative workup

Preoperative laboratory data were available for 26 patients (49%). Anemia was the most prevalent abnormality (62%), followed by elevated inflammatory markers: ESR (35%) and CRP (31%). Thrombocytosis and leukocytosis were noted in 23% and 19% of cases, respectively. Abdominal ultrasound and computed tomography (CT) were the most frequently utilized imaging modalities. Preoperative biopsy, reported in 11 patients, employed various techniques including colonoscopic biopsies, CT and US-guided FNAC, Tru-cut biopsies, and transanal biopsy. Notably, preoperative biopsy confirmed IMT diagnosis in only 5 of 11 cases (45%).

### Surgical interventions and medical management

Management details were available for 49 patients (92%), with 48 (98%) of them undergoing surgical resection. One case of rectal involvement was initially managed with a proximal diverting stoma, followed by successful non-surgical treatment using a six-week course of high-dose oral nonsteroidal anti-inflammatory drugs (NSAIDs) (600 mg ibuprofen, four times daily) [25]. Two additional cases employed NSAIDs with variable success, though details regarding dose, type or duration were not specified [11, 17]. Laparoscopy was utilized in 4 cases, including ours, with one case converting to open laparotomy and laparoscopic-assisted resection in the other three cases. Margin status was documented in 18 of the patients (38%) who underwent resection. Among these, two patients had microscopic positive margins and two had gross positive margins; however, recurrence occurred in only one of these patients. Regarding lymph node sampling, details about mesocolic lymph nodes were recorded in only 10 patients (19%). Histopathological evaluations revealed negative results in six patients, reactive changes in three patients, and spindle cell fibroblastic/myofibroblastic proliferation in one case [8].

### Follow-up and recurrence

Follow-up duration was reported for 39 patients, with a mean of 38 months (range: 3–181 months). Surveillance protocols demonstrated significant heterogeneity and were reported in only 15/53 patients (28%). Ultrasound was the predominant surveillance modality (n = 7), followed by computed tomography (n = 5) and colonoscopy (n = 3). Two patients with rectal IMTs underwent surveillance via clinical examination. The timing of initial postoperative imaging varied considerably: 5 patients underwent initial assessment at intervals between 1–12 months, 4 patients at 6 months, and individual patients at 2, 11, and 12 months, respectively. Notably, no studies in this review established evidence-based recommendations regarding optimal surveillance duration.

The overall prognosis was favorable, with only one postoperative death due to worsening sepsis [37]. Recurrence data were available for 45 cases, with local recurrence documented in 5 (11%) cases and no distant metastases reported. Recurrence timing varied, occurring at 2 months postoperatively in 3 cases and at 18 months in another case presenting as an abdominal wall mass. Management of recurrence was detailed in 3 cases, with approaches ranging from reoperation to chemotherapy and NSAID therapy [11, 14, 20]. In one case, multiple recurrences occurred but the patient achieved disease-free status at 6-year follow-up after NSAID therapy [11].

## Discussion

Inflammatory myofibroblastic tumors represent a distinctive category of neoplasms that can manifest in various anatomical locations, with a notable prevalence in the lungs, soft tissues, and abdominal organs of pediatric patients. However, their presence in the colorectal region of children is exceedingly rare, with only 52 documented cases to date. This comprehensive review consolidates all reported pediatric colorectal IMT cases, along with a previously unreported case from our institution, offering a focused examination of diagnostic challenges, and management strategies specific to children, thus addressing a gap in current literature where pediatric cases are often included within broader cohorts rather than analyzed as a distinct entity.

The etiology of IMTs remains elusive, with multiple proposed factors lacking substantial evidence. While infectious agents, autoimmune disorders, post-surgical inflammatory responses, malignancies, and genetic mutations have been suggested as potential causes [45], the current literature provides limited support for any single factor [39]. In our review, only five cases reported potential etiological associations: one linked to Clostridium difficile in a liver transplant patient [12], Epstein-Barr virus [14], Cytomegalovirus [30], Escherichia coli [37], and previous abdominal trauma [36]. Interestingly, two cases were incidentally discovered during abdominal exploration for hepatic hydatic cyst [9], and abdominal trauma [23].

The various clinical presentations of IMTs necessitate their inclusion in the differential diagnosis for pediatric patients presenting with abdominal masses or vague abdominal symptoms. The spectrum of presentations encompasses fever, failure to thrive, abdominal pain, abdominal or rectal mass, intestinal obstruction, and gastrointestinal bleeding [4]. Given that colonic IMTs can serve as a lead point for intussusception [26], it is noteworthy that our review identified 7 cases (15%) where IMTs either presented with intussusception or were diagnosed intra-operatively. Likewise, intestinal obstruction was reported in 15% of the patients, with four cases attributed to concurrent intussusception and three cases resulting from luminal obstruction by the mass.

The diagnostic complexity of colorectal IMTs extends to preoperative workup modalities, owing to the nonspecificity of laboratory findings [35]. Anemia, the most prevalent abnormality in our cohort (62%), likely reflects the chronicity of these tumors or gastrointestinal bleeding, reported in 29% of cases. The predominance of right-sided colonic IMTs (39%) suggests a propensity for occult blood loss, analogous to other right-sided colonic pathologies [46]. Other laboratory abnormalities, including elevated ESR and thrombocytosis, were observed in 35%, and 23% of cases respectively, reflecting the underlying inflammatory nature of these neoplasms [1, 47].

Imaging modalities play a crucial role in the diagnostic algorithm for colorectal IMTs, providing essential information about tumor location, size, and relationships with adjacent structures. While abdominal ultrasound serves as an initial screening tool, computed tomography and magnetic resonance imaging can offer superior delineation of tumor characteristics [29]. In patients with obstructive symptoms, IMTs typically manifest as eccentric bowel wall thickening, contrasting with the circumferential thickening more commonly associated with inflammatory processes or infections [48]. Larger lesions may exhibit calcifications and central necrosis, with varying degrees and patterns of contrast enhancement. These patterns can include early peripheral enhancement with delayed central filling, heterogeneous enhancement, homogeneous enhancement, or no enhancement [49, 50]. The potential for IMTs to mimic colonic adenocarcinoma radiologically, including the classic "apple core" sign, underscores the importance of meticulous radiological assessment to prevent misdiagnosis and guide appropriate management strategies [25].

Preoperative tissue diagnosis of colorectal IMTs remains challenging, with various biopsy techniques yielding inconsistent results [13, 24, 25, 32]. Our review revealed that only 45% (5/11) of patients who underwent preoperative biopsies achieved a definitive diagnosis, with Tru-cut biopsy techniques demonstrating marginally superior efficacy [35]. The limited diagnostic yield of fine needle aspiration biopsies aligns with previous findings [10], while core biopsies have also demonstrated suboptimal diagnostic accuracy [51]. Based on our findings and review of the literature, preoperative biopsy may be considered in select clinical scenarios: non-urgent presentations allowing time for diagnostic workup, suspected unresectable or ALK-positive tumors where neoadjuvant therapy might be beneficial, medically high-risk patients where avoiding unnecessary surgery is crucial, and lesions in anatomically accessible sites with diagnostic uncertainty. When biopsy is pursued, image-guided Tru-cut techniques (ultrasound or CT-guided) appear to offer improved diagnostic yield [51–54]. However, it should be emphasized that inconclusive biopsy results often necessitate surgical resection for definitive diagnosis. This diagnostic dilemma underscores the need for a balanced approach, carefully weighing the potential diagnostic benefits against additional anesthetic exposure and procedural risks in the pediatric population.

From a histopathological standpoint, IMTs exhibit a complex biological behavior characterized by local invasiveness and recurrence potential, yet with a low propensity for metastasis [55–57]. The microscopic architecture of IMTs is diverse, encompassing three distinct histological patterns: (1) a myxoid variant featuring loosely arranged spindle-shaped myofibroblasts, (2) a cellular pattern with densely packed stromal cells admixed with inflammatory infiltrates, and (3) a hypocellular pattern dominated by hyalinized stroma [1]. These patterns may occur in isolation or in combination within a single lesion, contributing to the histological heterogeneity of IMTs [58]. A key diagnostic feature distinguishing IMTs from more aggressive neoplasms, particularly sarcomas, is the absence or paucity of nuclear atypia and mitotic figures [11]. The immunohistochemical profile of IMTs is characterized by consistent, strong vimentin positivity, while the expression of desmin and smooth muscle actin (SMA) can be variable [59]. The accurate diagnosis of IMTs, particularly those arising in the sigmoid colon, relies heavily on a comprehensive immunohistochemical panel [41]. This panel should include staining for Anaplastic Lymphoma Kinase (ALK), which has emerged as a crucial diagnostic marker. Additionally, the evaluation of S-100 and CD-117 expression provides valuable information for differential diagnosis and tumor characterization [33, 59].

The management of colorectal IMTs necessitates a tailored approach, given their generally benign nature and infrequent malignant transformation [35]. While various treatment modalities have been reported, including chemotherapy, radiotherapy, corticosteroids, and immunomodulators, complete surgical resection remains the gold-standard treatment when technically feasible without significant functional compromise [19, 41, 42]. IMTs are primarily locally invasive tumors with rare instances of distant metastasis, raising questions about the necessity of extensive lymph node sampling. However, preoperative diagnostic uncertainty often necessitates adherence to standard oncologic principles, including en bloc resection.

Regarding post resection margin status, our review found documentation in only 38% of resection cases, highlighting a significant reporting gap in the literature. Among the four patients with positive margins (two microscopic, two gross), only one developed recurrence. This finding aligns with previous studies suggesting that occasional IMTs with positive resection margins neither recur nor progress [19, 35] Other multi-institutional studies have similarly observed that positive margins did not universally correlate with recurrence, suggesting that surgical approaches potentially compromising form or function solely to achieve negative margins may not always be warranted [60, 61]. In this sense, it is imperative for the treating surgeon to be cognizant of the natural history of this tumor to avoid excessive morbidity or mortality that may result from overly aggressive surgical interventions for this relatively benign entity [1].

Of particular interest is the emerging role of ALK inhibitors, either as monotherapy or as neoadjuvant therapy to facilitate subsequent surgical resection [62, 63]. While preoperative diagnosis of IMTs is generally challenging, neoadjuvant ALK inhibitor therapy is specifically considered in cases where IMT has been confirmed through preoperative tissue sampling and where the tumor is deemed initially unresectable [54, 60, 64]. ALK inhibitors specifically target tyrosine kinase activity in ALK-rearranged tumors, which represent approximately 50% of IMTs [65–67]. The treatment duration with these targeted agents typically ranges from several months to years, with recent multi-institutional studies documenting treatment duration ranging between 1 to 48 months in pediatric patients [60, 65]. This relatively prolonged treatment timeline reflects their mechanism of action, which requires sustained inhibition of oncogenic signalling pathways to control neoplastic proliferation. In contrast, the therapeutic efficacy of NSAIDs in IMTs operates through different mechanisms, including vascular endothelial growth factor (VEGF) downregulation, cyclooxygenase-2 (COX-2) inhibition, and cytokine suppression which would target the inflammatory processes potentially driving tumor development [68]. Despite previous reports depicting successful management of IMT with NSAIDS [69–71], our review identified only three patients treated with NSAIDs, with variable outcomes. Among these, only one case specified the dosage and duration (600 mg ibuprofen four times daily for 6 weeks) [25].

A review of the broader literature on IMTs reveals a notable lack of guidelines or consensus regarding NSAID use. Most available data derive from expert opinions rather than standardized protocols, resulting in variability concerning drug selection, dosage, and treatment duration [69, 72, 73]. The typically shorter treatment duration with NSAIDs, when effective, may reflect their mechanism of targeting the inflammatory processes involved with tumor development rather than primary oncogenic drivers. Such significant heterogeneity in protocols for both NSAIDs and ALK inhibitors, coupled with the absence of prospective clinical trials and the limited data on their use in pediatric patients underscores the critical need for further investigation into optimal treatment strategies for colorectal IMT management.

The recurrence of IMTs presents an ongoing clinical challenge, with implications for long-term management strategies. Recurrence is primarily attributed to incomplete excision during initial surgery [14], or often due to technical challenges in anatomically complex locations such as the deep rectum. While the overall recurrence rate of IMTs in pediatric patients is approximately 14%, intra-abdominal IMTs have been reported to have recurrence rates exceeding 75% [74]. Our focused review of colorectal IMTs revealed a local recurrence rate of 11% (5 cases), with three cases recurring within two months and one case at 18 months post-resection. Importantly, no distant metastases were reported.

The optimal follow-up protocol for IMTs following surgical resection remains an area without definitive consensus. Our analysis revealed marked heterogeneity in both the frequency and modality of surveillance across reported cases, reflecting the lack of standardized guidelines. Despite most recurrences occurring within the first two years after resection [19, 65], we observed considerable variation in the timing of initial postoperative evaluation and subsequent monitoring. While ultrasound was most frequently employed, followed by CT and colonoscopy, the selection appears to have been influenced by institutional preferences rather than evidence-based protocols. Based on recurrence patterns identified in our review and the broader literature, we propose a risk-stratified surveillance approach. For standard-risk cases, cross-sectional imaging every 6 months for the first 2 years, followed by annual imaging for at least 3 additional years appears reasonable. More intensive surveillance should be considered for high-risk features including positive or close resection margins, ALK-negative status, larger tumor size (> 5 cm), locally invasive behavior, or recurrent disease [60, 75–77]. The choice between ultrasound, CT, MRI, or endoscopic evaluation should be individualized based on tumor location, patient age, radiation exposure considerations, and institutional expertise. Prospective, multi-institutional studies are urgently needed to establish evidence-based surveillance protocols for these rare tumors.

The optimal treatment option for recurrent disease remains unclear; however, surgical re-excision is generally the preferred management strategy for when feasible [3, 78]. In our review, surgical re-excision was performed in one case following initial partial excision [14]. Additionally, successful regression was achieved in two recurrent cases using NSAIDs and chemotherapy [11, 20]. In instances of recurrent IMTs with aggressive abdominal invasion, palliative resection may be considered for symptom alleviation [39]. Furthermore, recent multicentre trials have led to the FDA approval of Crizotinib, an ALK inhibitor, for use in adult and pediatric patients with unresectable, recurrent, or refractory ALK-positive IMTs, representing a promising advancement in the management of recurrence [63].

When interpreting our results, it is essential to acknowledge several limitations inherent to this systematic review. Firstly, our stringent inclusion criteria, which required precise tumor localization and detailed management and outcome data, led to the exclusion of studies that lacked such specificity. While this approach ensured a homogeneous population of pediatric colonic IMT cases, it may have inadvertently limited the comprehensiveness of our literature overview. Secondly, the predominance of case reports and small case series in our included studies introduces potential biases in patient selection and data reporting. The retrospective nature of these studies, coupled with heterogeneous patient cohorts, limited sample sizes, and the lack of standardized follow-up protocols, restricts broader statistical analysis and constrains the generalizability of our findings. These challenges are particularly pertinent in rare conditions such as pediatric colonic IMTs, where large-scale prospective studies are inherently difficult to conduct. The methodological approach employed in this study—combining a case report with a systematic review of predominantly case-level data— was specifically chosen given the ultra-rare nature of pediatric colonic inflammatory myofibroblastic tumors. For such conditions, randomized controlled trials or large cohort studies are not feasible, making the systematic collection and synthesis of case reports the highest attainable level of evidence. Additionally, we considered the potential for publication bias, acknowledging that positive outcomes might be more likely to be reported, which is common in rare disease literature. Lastly, the heterogeneity in reporting outcomes across studies further precluded meaningful statistical comparisons or meta-analyses. This limitation underscores the need for more standardized reporting protocols in future studies of rare pediatric colorectal pathologies.

Despite these limitations, our systematic review represents the most comprehensive analysis of pediatric colonic IMTs to date, providing valuable insights into this rare entity. Future multi-institutional collaborative efforts and the establishment of international registries could help overcome these limitations and further enhance our understanding of this challenging condition.

## Conclusion

The occurrence of inflammatory myofibroblastic tumor is extremely rare but it should be considered in the differential diagnosis of any colic mass in the pediatric age group. Good results can be expected from a complete resection with advisory long-term follow-up due to the potential for recurrence of the tumor even many years after resection.

## Supplementary Information

Below is the link to the electronic supplementary material.

Supplementary file1 (DOCX 17 KB)

Supplementary file2 (DOCX 3787 KB)

Supplementary file3 (DOCX 32 KB)

Supplementary file4 (DOCX 87 KB)

## Data Availability

No datasets were generated or analysed during the current study.

## Declarations

### Protocol registration

The study protocol was not registered.
