# Supplementary material for: Inflammatory myofibroblastic tumors of the colon in pediatrics: clinical presentation, management, and outcomes—A case report and systematic review of literature
Source: Int J Colorectal Dis. 2025 Apr 15;40(1):94. doi: 10.1007/s00384-025-04869-y (PMC12000112; doi:10.1007/s00384-025-04869-y)
Supplement: Supplementary file 1 — Supplementary file1 (DOCX 17 KB) [file 384_2025_4869_MOESM1_ESM.docx]

Search strategy implemented during PubMed search:

#1 Search: **((((inflammatory[Title/Abstract])) AND (myofibroblastic[Title/Abstract] OR pseudotumor[Title/Abstract])) AND (tumor[Title/Abstract] OR tumour[Title/Abstract] OR tumors[Title/Abstract] OR tumours[Title/Abstract])**

#2 Search: **colon OR rectum OR colorectal OR sigmoid OR rectosigmoid OR rectal OR abdomen OR abdominal OR pelvis OR bowel OR intestinal OR colonic**

"colon"[MeSH Terms] OR "colon"[All Fields] OR "colonic"[All Fields] OR "colons"[All Fields] OR "colon s"[All Fields] OR "colonal"[All Fields] OR "colonically"[All Fields] OR "colonitis"[All Fields] OR ("rectum"[MeSH Terms] OR "rectum"[All Fields] OR "rectums"[All Fields]) OR "colorectal"[All Fields] OR ("colon, sigmoid"[MeSH Terms] OR ("colon"[All Fields] AND "sigmoid"[All Fields]) OR "sigmoid colon"[All Fields] OR "sigmoid"[All Fields] OR "sigmoidal"[All Fields] OR "sigmoidally"[All Fields] OR "sigmoidicity"[All Fields] OR "sigmoiditis"[All Fields] OR "sigmoids"[All Fields]) OR ("proctocolitis"[MeSH Terms] OR "proctocolitis"[All Fields] OR "rectosigmoiditis"[All Fields] OR "rectosigmoid"[All Fields] OR "rectosigmoidal"[All Fields] OR "rectosigmoideal"[All Fields]) OR ("administration, rectal"[MeSH Terms] OR ("administration"[All Fields] AND "rectal"[All Fields]) OR "rectal administration"[All Fields] OR "rectal"[All Fields]) OR ("abdomen"[MeSH Terms] OR "abdomen"[All Fields] OR "abdomens"[All Fields] OR "abdomen s"[All Fields] OR "abdominal cavity"[MeSH Terms] OR ("abdominal"[All Fields] AND "cavity"[All Fields]) OR "abdominal cavity"[All Fields]) OR ("abdomen"[MeSH Terms] OR "abdomen"[All Fields] OR "abdominal"[All Fields] OR "abdominally"[All Fields] OR "abdominals"[All Fields]) OR ("pelvi"[All Fields] OR "pelvis"[MeSH Terms] OR "pelvis"[All Fields]) OR ("bowel s"[All Fields] OR "bowell"[All Fields] OR "intestines"[MeSH Terms] OR "intestines"[All Fields] OR "bowel"[All Fields] OR "bowels"[All Fields]) OR ("intestinalization"[All Fields] OR "intestinalized"[All Fields] OR "intestinally"[All Fields] OR "intestinals"[All Fields] OR "intestine s"[All Fields] OR "intestines"[MeSH Terms] OR "intestines"[All Fields] OR "intestinal"[All Fields] OR "intestine"[All Fields]) OR ("colon"[MeSH Terms] OR "colon"[All Fields] OR "colonic"[All Fields] OR "colons"[All Fields] OR "colon s"[All Fields] OR "colonal"[All Fields] OR "colonically"[All Fields] OR "colonitis"[All Fields])

**Translations**

**colon:** "colon"[MeSH Terms] OR "colon"[All Fields] OR "colonic"[All Fields] OR "colons"[All Fields] OR "colon's"[All Fields] OR "colonal"[All Fields] OR "colonically"[All Fields] OR "colonitis"[All Fields]

**rectum:** "rectum"[MeSH Terms] OR "rectum"[All Fields] OR "rectums"[All Fields]

**sigmoid:** "colon, sigmoid"[MeSH Terms] OR ("colon"[All Fields] AND "sigmoid"[All Fields]) OR "sigmoid colon"[All Fields] OR "sigmoid"[All Fields] OR "sigmoidal"[All Fields] OR "sigmoidally"[All Fields] OR "sigmoidicity"[All Fields] OR "sigmoiditis"[All Fields] OR "sigmoids"[All Fields]

**rectosigmoid:** "proctocolitis"[MeSH Terms] OR "proctocolitis"[All Fields] OR "rectosigmoiditis"[All Fields] OR "rectosigmoid"[All Fields] OR "rectosigmoidal"[All Fields] OR "rectosigmoideal"[All Fields]

**rectal:** "administration, rectal"[MeSH Terms] OR ("administration"[All Fields] AND "rectal"[All Fields]) OR "rectal administration"[All Fields] OR "rectal"[All Fields]

**abdomen:** "abdomen"[MeSH Terms] OR "abdomen"[All Fields] OR "abdomens"[All Fields] OR "abdomen's"[All Fields] OR "abdominal cavity"[MeSH Terms] OR ("abdominal"[All Fields] AND "cavity"[All Fields]) OR "abdominal cavity"[All Fields]

**abdominal:** "abdomen"[MeSH Terms] OR "abdomen"[All Fields] OR "abdominal"[All Fields] OR "abdominally"[All Fields] OR "abdominals"[All Fields]

**pelvis:** "pelvi"[All Fields] OR "pelvis"[MeSH Terms] OR "pelvis"[All Fields]

**bowel:** "bowel's"[All Fields] OR "bowell"[All Fields] OR "intestines"[MeSH Terms] OR "intestines"[All Fields] OR "bowel"[All Fields] OR "bowels"[All Fields]

**intestinal:** "intestinalization"[All Fields] OR "intestinalized"[All Fields] OR "intestinally"[All Fields] OR "intestinals"[All Fields] OR "intestine's"[All Fields] OR "intestines"[MeSH Terms] OR "intestines"[All Fields] OR "intestinal"[All Fields] OR "intestine"[All Fields]

**colonic:** "colon"[MeSH Terms] OR "colon"[All Fields] OR "colonic"[All Fields] OR "colons"[All Fields] OR "colon's"[All Fields] OR "colonal"[All Fields] OR "colonically"[All Fields] OR "colonitis"[All Fields]

#3 Search: **pediatric OR child OR children OR Childhood OR boy OR girl OR infant OR toddler OR ALK OR pediatric OR pediatrics**

"paediatrics"[All Fields] OR "pediatrics"[MeSH Terms] OR "pediatrics"[All Fields] OR "paediatric"[All Fields] OR "pediatric"[All Fields] OR "child"[MeSH Terms] OR "child"[All Fields] OR "children"[All Fields] OR "child s"[All Fields] OR "children s"[All Fields] OR "childrens"[All Fields] OR "childs"[All Fields] OR "child"[MeSH Terms] OR "child"[All Fields] OR "children"[All Fields] OR "child s"[All Fields] OR "children s"[All Fields] OR "childrens"[All Fields] OR "childs"[All Fields] OR "childhood"[All Fields] OR "childhoods"[All Fields] OR "men"[MeSH Terms] OR "men"[All Fields] OR "boy"[All Fields] OR "women"[MeSH Terms] OR "women"[All Fields] OR "girl"[All Fields] OR "infant"[MeSH Terms] OR "infant"[All Fields] OR "infants"[All Fields] OR "infant s"[All Fields] OR "toddler"[All Fields] OR "toddler s"[All Fields] OR "toddlers"[All Fields] OR "ALK"[All Fields] OR "paediatrics"[All Fields] OR "pediatrics"[MeSH Terms] OR "pediatrics"[All Fields] OR "paediatric"[All Fields] OR "pediatric"[All Fields] OR "paediatrics"[All Fields] OR "pediatrics"[MeSH Terms] OR "pediatrics"[All Fields] OR "paediatric"[All Fields] OR "pediatric"[All Fields]

**Translations**

**pediatric:** "paediatrics"[All Fields] OR "pediatrics"[MeSH Terms] OR "pediatrics"[All Fields] OR "paediatric"[All Fields] OR "pediatric"[All Fields]

**child:** "child"[MeSH Terms] OR "child"[All Fields] OR "children"[All Fields] OR "child's"[All Fields] OR "children's"[All Fields] OR "childrens"[All Fields] OR "childs"[All Fields]

**children:** "child"[MeSH Terms] OR "child"[All Fields] OR "children"[All Fields] OR "child's"[All Fields] OR "children's"[All Fields] OR "childrens"[All Fields] OR "childs"[All Fields]

**Childhood:** "childhood"[All Fields] OR "childhoods"[All Fields]

**boy:** "men"[MeSH Terms] OR "men"[All Fields] OR "boy"[All Fields]

**girl:** "women"[MeSH Terms] OR "women"[All Fields] OR "girl"[All Fields]

**infant:** "infant"[MeSH Terms] OR "infant"[All Fields] OR "infants"[All Fields] OR "infant's"[All Fields]

**toddler:** "toddler"[All Fields] OR "toddler's"[All Fields] OR "toddlers"[All Fields]

**pediatric:** "paediatrics"[All Fields] OR "pediatrics"[MeSH Terms] OR "pediatrics"[All Fields] OR "paediatric"[All Fields] OR "pediatric"[All Fields]

**pediatrics:** "paediatrics"[All Fields] OR "pediatrics"[MeSH Terms] OR "pediatrics"[All Fields] OR "paediatric"[All Fields] OR "pediatric"[All Fields]

#4 Search: **#1 AND #2 AND #3**

"inflammatory"[Title/Abstract] AND ("myofibroblastic"[Title/Abstract] OR "pseudotumor"[Title/Abstract]) AND ("tumor"[Title/Abstract] OR "tumour"[Title/Abstract] OR "tumors"[Title/Abstract] OR "tumours"[Title/Abstract]) AND ("colon"[MeSH Terms] OR "colon"[All Fields] OR "colonic"[All Fields] OR "colons"[All Fields] OR "colon s"[All Fields] OR "colonal"[All Fields] OR "colonically"[All Fields] OR "colonitis"[All Fields] OR ("rectum"[MeSH Terms] OR "rectum"[All Fields] OR "rectums"[All Fields]) OR "colorectal"[All Fields] OR ("colon, sigmoid"[MeSH Terms] OR ("colon"[All Fields] AND "sigmoid"[All Fields]) OR "sigmoid colon"[All Fields] OR "sigmoid"[All Fields] OR "sigmoidal"[All Fields] OR "sigmoidally"[All Fields] OR "sigmoidicity"[All Fields] OR "sigmoiditis"[All Fields] OR "sigmoids"[All Fields]) OR ("proctocolitis"[MeSH Terms] OR "proctocolitis"[All Fields] OR "rectosigmoiditis"[All Fields] OR "rectosigmoid"[All Fields] OR "rectosigmoidal"[All Fields] OR "rectosigmoideal"[All Fields]) OR ("administration, rectal"[MeSH Terms] OR ("administration"[All Fields] AND "rectal"[All Fields]) OR "rectal administration"[All Fields] OR "rectal"[All Fields]) OR ("abdomen"[MeSH Terms] OR "abdomen"[All Fields] OR "abdomens"[All Fields] OR "abdomen s"[All Fields] OR "abdominal cavity"[MeSH Terms] OR ("abdominal"[All Fields] AND "cavity"[All Fields]) OR "abdominal cavity"[All Fields]) OR ("abdomen"[MeSH Terms] OR "abdomen"[All Fields] OR "abdominal"[All Fields] OR "abdominally"[All Fields] OR "abdominals"[All Fields]) OR ("pelvi"[All Fields] OR "pelvis"[MeSH Terms] OR "pelvis"[All Fields]) OR ("bowel s"[All Fields] OR "bowell"[All Fields] OR "intestines"[MeSH Terms] OR "intestines"[All Fields] OR "bowel"[All Fields] OR "bowels"[All Fields]) OR ("intestinalization"[All Fields] OR "intestinalized"[All Fields] OR "intestinally"[All Fields] OR "intestinals"[All Fields] OR "intestine s"[All Fields] OR "intestines"[MeSH Terms] OR "intestines"[All Fields] OR "intestinal"[All Fields] OR "intestine"[All Fields]) OR ("colon"[MeSH Terms] OR "colon"[All Fields] OR "colonic"[All Fields] OR "colons"[All Fields] OR "colon s"[All Fields] OR "colonal"[All Fields] OR "colonically"[All Fields] OR "colonitis"[All Fields])) AND ("paediatrics"[All Fields] OR "pediatrics"[MeSH Terms] OR "pediatrics"[All Fields] OR "paediatric"[All Fields] OR "pediatric"[All Fields] OR ("child"[MeSH Terms] OR "child"[All Fields] OR "children"[All Fields] OR "child s"[All Fields] OR "children s"[All Fields] OR "childrens"[All Fields] OR "childs"[All Fields]) OR ("child"[MeSH Terms] OR "child"[All Fields] OR "children"[All Fields] OR "child s"[All Fields] OR "children s"[All Fields] OR "childrens"[All Fields] OR "childs"[All Fields]) OR ("childhood"[All Fields] OR "childhoods"[All Fields]) OR ("men"[MeSH Terms] OR "men"[All Fields] OR "boy"[All Fields]) OR ("women"[MeSH Terms] OR "women"[All Fields] OR "girl"[All Fields]) OR ("infant"[MeSH Terms] OR "infant"[All Fields] OR "infants"[All Fields] OR "infant s"[All Fields]) OR ("toddler"[All Fields] OR "toddler s"[All Fields] OR "toddlers"[All Fields]) OR "ALK"[All Fields] OR ("paediatrics"[All Fields] OR "pediatrics"[MeSH Terms] OR "pediatrics"[All Fields] OR "paediatric"[All Fields] OR "pediatric"[All Fields]) OR ("paediatrics"[All Fields] OR "pediatrics"[MeSH Terms] OR "pediatrics"[All Fields] OR "paediatric"[All Fields] OR "pediatric"[All Fields]))

**Translations**

**colon:** "colon"[MeSH Terms] OR "colon"[All Fields] OR "colonic"[All Fields] OR "colons"[All Fields] OR "colon's"[All Fields] OR "colonal"[All Fields] OR "colonically"[All Fields] OR "colonitis"[All Fields]

**rectum:** "rectum"[MeSH Terms] OR "rectum"[All Fields] OR "rectums"[All Fields]

**sigmoid:** "colon, sigmoid"[MeSH Terms] OR ("colon"[All Fields] AND "sigmoid"[All Fields]) OR "sigmoid colon"[All Fields] OR "sigmoid"[All Fields] OR "sigmoidal"[All Fields] OR "sigmoidally"[All Fields] OR "sigmoidicity"[All Fields] OR "sigmoiditis"[All Fields] OR "sigmoids"[All Fields]

**rectosigmoid:** "proctocolitis"[MeSH Terms] OR "proctocolitis"[All Fields] OR "rectosigmoiditis"[All Fields] OR "rectosigmoid"[All Fields] OR "rectosigmoidal"[All Fields] OR "rectosigmoideal"[All Fields]

**rectal:** "administration, rectal"[MeSH Terms] OR ("administration"[All Fields] AND "rectal"[All Fields]) OR "rectal administration"[All Fields] OR "rectal"[All Fields]

**abdomen:** "abdomen"[MeSH Terms] OR "abdomen"[All Fields] OR "abdomens"[All Fields] OR "abdomen's"[All Fields] OR "abdominal cavity"[MeSH Terms] OR ("abdominal"[All Fields] AND "cavity"[All Fields]) OR "abdominal cavity"[All Fields]

**abdominal:** "abdomen"[MeSH Terms] OR "abdomen"[All Fields] OR "abdominal"[All Fields] OR "abdominally"[All Fields] OR "abdominals"[All Fields]

**pelvis:** "pelvi"[All Fields] OR "pelvis"[MeSH Terms] OR "pelvis"[All Fields]

**bowel:** "bowel's"[All Fields] OR "bowell"[All Fields] OR "intestines"[MeSH Terms] OR "intestines"[All Fields] OR "bowel"[All Fields] OR "bowels"[All Fields]

**intestinal:** "intestinalization"[All Fields] OR "intestinalized"[All Fields] OR "intestinally"[All Fields] OR "intestinals"[All Fields] OR "intestine's"[All Fields] OR "intestines"[MeSH Terms] OR "intestines"[All Fields] OR "intestinal"[All Fields] OR "intestine"[All Fields]

**colonic:** "colon"[MeSH Terms] OR "colon"[All Fields] OR "colonic"[All Fields] OR "colons"[All Fields] OR "colon's"[All Fields] OR "colonal"[All Fields] OR "colonically"[All Fields] OR "colonitis"[All Fields]

**pediatric:** "paediatrics"[All Fields] OR "pediatrics"[MeSH Terms] OR "pediatrics"[All Fields] OR "paediatric"[All Fields] OR "pediatric"[All Fields]

**child:** "child"[MeSH Terms] OR "child"[All Fields] OR "children"[All Fields] OR "child's"[All Fields] OR "children's"[All Fields] OR "childrens"[All Fields] OR "childs"[All Fields]

**children:** "child"[MeSH Terms] OR "child"[All Fields] OR "children"[All Fields] OR "child's"[All Fields] OR "children's"[All Fields] OR "childrens"[All Fields] OR "childs"[All Fields]

**Childhood:** "childhood"[All Fields] OR "childhoods"[All Fields]

**boy:** "men"[MeSH Terms] OR "men"[All Fields] OR "boy"[All Fields]

**girl:** "women"[MeSH Terms] OR "women"[All Fields] OR "girl"[All Fields]

**infant:** "infant"[MeSH Terms] OR "infant"[All Fields] OR "infants"[All Fields] OR "infant's"[All Fields]

**toddler:** "toddler"[All Fields] OR "toddler's"[All Fields] OR "toddlers"[All Fields]

**pediatric:** "paediatrics"[All Fields] OR "pediatrics"[MeSH Terms] OR "pediatrics"[All Fields] OR "paediatric"[All Fields] OR "pediatric"[All Fields]

**pediatrics:** "paediatrics"[All Fields] OR "pediatrics"[MeSH Terms] OR "pediatrics"[All Fields] OR "paediatric"[All Fields] OR "pediatric"[All Fields]
