# Supplementary material for: Inflammatory myofibroblastic tumors of the colon in pediatrics: clinical presentation, management, and outcomes—A case report and systematic review of literature
Source: Int J Colorectal Dis. 2025 Apr 15;40(1):94. doi: 10.1007/s00384-025-04869-y (PMC12000112; doi:10.1007/s00384-025-04869-y)
Supplement: Supplementary file 2 — Supplementary file2 (DOCX 3787 KB) [file 384_2025_4869_MOESM2_ESM.docx]

PMID- 39816042

OWN - NLM

STAT- PubMed-not-MEDLINE

LR - 20250116

IS - 2078-6891 (Print)

IS - 2219-679X (Electronic)

IS - 2078-6891 (Linking)

VI - 15

IP - 6

DP - 2024 Dec 31

TI - Inflammatory pseudotumor-like extranodal classic Hodgkin lymphoma manifesting as

bowel perforation in acquired immunodeficiency syndrome patient with disseminated

leishmaniasis: a case report and approach to differential diagnosis.

PG - 2684-2691

LID - 10.21037/jgo-24-499 [doi]

AB - BACKGROUND: Classic Hodgkin lymphoma (CHL) is an extremely common non-acquired

immunodeficiency syndrome (AIDS) defining malignancy and its incidence is rising.

CHL is usually present in the lymph node and extranodal involvement is rare.

Primary CHL of the gastrointestinal (GI) tract is exceedingly rare. CASE

DESCRIPTION: In this case, a patient with human immunodeficiency virus (HIV)/AIDS

and disseminated leishmaniasis presented with a small bowel mass leading to bowel

perforation. Histologically, the small bowel mass showed a transmural infiltrate

of scattered large atypical multinucleated cells surrounded by histiocytes and

T-cells. Initial differential diagnosis was wide due to the unusual presentation

and cytologic atypia of the tumor cells. Identifying the Hodgkins Reed-Sternberg

(HRS) cells with their unique immunophenotype was key for diagnosis. CONCLUSIONS:

It is critical to identify secondary CHL in HIV/AIDS patients especially in the

presence of immunodeficiency and disseminated opportunistic infection. Extranodal

primary GI tract CHL is exceedingly rare and thus awareness of this entity, which

can mimic many other tumors, especially in immunocompromised individuals, is

important. Special stains and cultures are helpful for the diagnosis, and

antimicrobial therapy will induce successful clinical outcome. Overall, the

unusual combination of acute clinical presentation, leishmaniasis, and HIV made

the histological recognition of CHL crucial to avoid misdiagnosis and guide

successful clinical management.

CI - 2024 AME Publishing Company. All rights reserved.

FAU - Mehr, Joshua

AU - Mehr J

AUID- ORCID: 0000-0002-2646-3041

AD - Department of Pathology, University of Texas Southwestern Medical Center, Dallas,

TX, USA.

FAU - Germans, Sharon

AU - Germans S

AD - Department of Pathology, University of Texas Southwestern Medical Center, Dallas,

TX, USA.

FAU - Chen, Weina

AU - Chen W

AD - Department of Pathology, University of Texas Southwestern Medical Center, Dallas,

TX, USA.

FAU - Mahsoub, Sameh

AU - Mahsoub S

AD - Department of Pathology, University of Texas Southwestern Medical Center, Dallas,

TX, USA.

FAU - Chen, Mingyi

AU - Chen M

AD - Department of Pathology, University of Texas Southwestern Medical Center, Dallas,

TX, USA.

LA - eng

PT - Case Reports

PT - Journal Article

DEP - 20241227

PL - China

TA - J Gastrointest Oncol

JT - Journal of gastrointestinal oncology

JID - 101557751

PMC - PMC11732359

OTO - NOTNLM

OT - Hodgkin lymphoma

OT - Human immunodeficiency virus (HIV)

OT - case report

OT - gastrointestinal complications (GI complications)

OT - leishmaniasis

COIS- Conflicts of Interest: All authors have completed the ICMJE uniform disclosure

form (available at

https://jgo.amegroups.com/article/view/10.21037/jgo-24-499/coif). The authors

have no conflicts of interest to declare.

EDAT- 2025/01/16 06:21

MHDA- 2025/01/16 06:22

PMCR- 2024/12/31

CRDT- 2025/01/16 04:33

PHST- 2024/07/02 00:00 [received]

PHST- 2024/10/21 00:00 [accepted]

PHST- 2025/01/16 06:22 [medline]

PHST- 2025/01/16 06:21 [pubmed]

PHST- 2025/01/16 04:33 [entrez]

PHST- 2024/12/31 00:00 [pmc-release]

AID - jgo-15-06-2684 [pii]

AID - 10.21037/jgo-24-499 [doi]

PST - ppublish

SO - J Gastrointest Oncol. 2024 Dec 31;15(6):2684-2691. doi: 10.21037/jgo-24-499. Epub

2024 Dec 27.

PMID- 39790401

OWN - NLM

STAT- PubMed-not-MEDLINE

LR - 20250111

IS - 2772-9931 (Electronic)

IS - 2772-9931 (Linking)

VI - 2

IP - 3

DP - 2024 Sep

TI - Inflammatory Myofibroblastic Tumor of the Lung: An Incidental Finding of a

Pediatric Case.

PG - 477-480

LID - 10.1016/j.atssr.2024.02.006 [doi]

AB - Inflammatory myofibroblastic tumor is a rare occurring benign tumor composed of

myofibroblastic spindle cells. Lung inflammatory myofibroblastic tumor is

difficult to diagnose and may mimic lung cancer or infectious etiology. Surgical

intervention with final histopathologic confirmation remains the mainstay of

diagnosis. We report an incidental finding of a pediatric case of lung

inflammatory myofibroblastic tumor with clinical presentation, management, and

outcomes at more than 2 years of follow-up at a tertiary care hospital in Saudi

Arabia.

CI - © 2024 The Authors.

FAU - Hammad, Rotana Sadaqah

AU - Hammad RS

AD - Department of Pediatric Pulmonary Medicine, King Fahd Armed Forces Hospital,

Jeddah, Saudi Arabia.

FAU - Alsehali, Awatif

AU - Alsehali A

AD - Department of Pediatric Pulmonary Medicine, King Fahd Armed Forces Hospital,

Jeddah, Saudi Arabia.

FAU - Tewfik, Khourshed

AU - Tewfik K

AD - Department of Pediatric Pulmonary Medicine, King Fahd Armed Forces Hospital,

Jeddah, Saudi Arabia.

FAU - AlHarbi, Reem

AU - AlHarbi R

AD - Department of Pediatric Hematology Oncology, King Saud Medical City, Riyadh,

Saudi Arabia.

FAU - Junainah, Elaf

AU - Junainah E

AD - Pediatric Pulmonary Medicine Department, King Faisal Specialist Hospital and

Research Centre, Jeddah, Saudi Arabia.

FAU - Felemban, Mohammed

AU - Felemban M

AD - Health Surveillance Centers, Ministry of Health, Jeddah Saudi Arabia.

LA - eng

PT - Case Reports

PT - Journal Article

DEP - 20240307

PL - United States

TA - Ann Thorac Surg Short Rep

JT - Annals of thoracic surgery short reports

JID - 9918506088006676

PMC - PMC11708618

EDAT- 2025/01/10 06:19

MHDA- 2025/01/10 06:20

PMCR- 2024/03/07

CRDT- 2025/01/10 04:17

PHST- 2024/02/26 00:00 [accepted]

PHST- 2025/01/10 06:20 [medline]

PHST- 2025/01/10 06:19 [pubmed]

PHST- 2025/01/10 04:17 [entrez]

PHST- 2024/03/07 00:00 [pmc-release]

AID - S2772-9931(24)00109-8 [pii]

AID - 10.1016/j.atssr.2024.02.006 [doi]

PST - epublish

SO - Ann Thorac Surg Short Rep. 2024 Mar 7;2(3):477-480. doi:

10.1016/j.atssr.2024.02.006. eCollection 2024 Sep.

PMID- 39730007

OWN - NLM

STAT- MEDLINE

DCOM- 20250110

LR - 20250110

IS - 1536-3732 (Electronic)

IS - 1049-2275 (Linking)

VI - 36

IP - 1

DP - 2025 Jan-Feb 01

TI - Locally Aggressive and Recurrent Facial Inflammatory Myofibroblastic Tumor in a

Pediatric Patient.

PG - e70-e72

LID - 10.1097/SCS.0000000000010771 [doi]

AB - Inflammatory myofibroblastic tumor (IMT) is a rare tumor type with a prognosis

ranging from benign to locally aggressive. Initially described as a reactive

lesion most commonly of the lungs, cases of IMT have now been reported in rare

instances in the head and neck, which may be more aggressive than other tumor

locations. IMT frequently afflicts children and adolescents, but pediatric cases

of IMT in the head and neck region are rare. This report serves to describe a

rare presentation of a recurrent and locally aggressive facial IMT in a pediatric

patient that required multiple surgical resections alongside medical management.

CI - Copyright © 2024 by Mutaz B. Habal, MD.

FAU - Clore, Lauren N

AU - Clore LN

AD - Department of Plastic Surgery, MedStar Georgetown University Hospital,

Washington, DC.

FAU - Margulies, Ilana G

AU - Margulies IG

FAU - Baker, Stephen B

AU - Baker SB

LA - eng

PT - Case Reports

PT - Journal Article

DEP - 20241015

PL - United States

TA - J Craniofac Surg

JT - The Journal of craniofacial surgery

JID - 9010410

SB - IM

MH - Humans

MH - *Neoplasm Recurrence, Local/pathology/surgery

MH - Facial Neoplasms/pathology/surgery

MH - Male

MH - Diagnosis, Differential

MH - Child

MH - Granuloma, Plasma Cell/surgery/pathology/diagnostic imaging/diagnosis

MH - Female

MH - Neoplasms, Muscle Tissue/surgery/pathology/diagnosis

MH - Adolescent

COIS- The authors report no conflicts of interest.

EDAT- 2024/12/28 11:45

MHDA- 2025/01/10 18:19

CRDT- 2024/12/27 18:52

PHST- 2024/09/10 00:00 [received]

PHST- 2024/09/10 00:00 [accepted]

PHST- 2025/01/10 18:19 [medline]

PHST- 2024/12/28 11:45 [pubmed]

PHST- 2024/12/27 18:52 [entrez]

AID - 00001665-990000000-02057 [pii]

AID - 10.1097/SCS.0000000000010771 [doi]

PST - ppublish

SO - J Craniofac Surg. 2025 Jan-Feb 01;36(1):e70-e72. doi:

10.1097/SCS.0000000000010771. Epub 2024 Oct 15.

PMID- 39713609

OWN - NLM

STAT- PubMed-not-MEDLINE

LR - 20250104

IS - 2050-313X (Print)

IS - 2050-313X (Electronic)

IS - 2050-313X (Linking)

VI - 12

DP - 2024

TI - Anaplastic lymphoma kinase1 positive inflammatory myofibroblastic tumor of the

urinary bladder: A rare mesenchymal neoplasm with diagnostic and therapeutic

implications.

PG - 2050313X241308992

LID - 10.1177/2050313X241308992 [doi]

LID - 2050313X241308992

AB - Inflammatory myofibroblastic tumors (IMTs) are rare mesenchymal neoplasms

characterized by spindle-cell morphology with accompanying inflammatory

infiltrates. Originally described in 1939, these tumors can arise in various

anatomic locations, with the urinary bladder being a rare site of occurrence but

the most common within the genitourinary tract. IMTs typically present as

polypoid masses or firm submucosal nodules, often with painless hematuria in

bladder cases. Histopathologically, IMTs are composed of myofibroblasts with

myxoid stroma and mixed inflammatory cells, predominantly lymphocytes and plasma

cells. Immunohistochemically, these tumors commonly express anaplastic lymphoma

kinase1 (ALK1), vimentin, smooth muscle actin (SMA), and cytokeratin, with ALK1

serving as a crucial marker for diagnosis. This report details the case of a

31-year-old female presenting with hematuria, found to have a soft tissue mass in

the urinary bladder (5.0 × 3.0 cm). Imaging revealed a well-defined lesion with

vascularity. Histopathological examination confirmed an IMT, with

immunohistochemistry showing diffuse ALK1 positivity, patchy SMA staining, and

variable desmin expression, consistent with the diagnosis. IMTs are generally

considered neoplasms of intermediate malignant potential. While metastasis is

exceedingly rare in bladder IMTs, local recurrence has been reported,

particularly in cases of incomplete surgical resection. Recent advances highlight

the role of ALK inhibitors in managing unresectable cases, enabling partial

cystectomy in select patients. This article underscores the importance of

achieving complete surgical excision and highlights the role of ALK expression in

diagnosis and differentiation from other spindle-cell neoplasms. Further studies

are needed to elucidate the molecular and clinical factors influencing prognosis

and to refine treatment strategies for IMTs.

CI - © The Author(s) 2024.

FAU - Poombal, Fnu

AU - Poombal F

AUID- ORCID: 0009-0005-8569-6805

AD - Department of Pathology, UMass Chan Medical School - Baystate Regional Campus

Ringgold standard institution, Springfield, MA, USA.

FAU - Mansoor, Ibrahim

AU - Mansoor I

AD - Department of Pathology, Kings College London, Jeddah, Saudi Arabia.

FAU - Abdellatif, Randa M

AU - Abdellatif RM

AD - Consultant Histopathology, Department of Pathology & Lab Med, Jeddah, Saudi

Arabia.

FAU - Shaker, Nada

AU - Shaker N

AUID- ORCID: 0000-0001-6145-256X

AD - Department of Pathology, University of California San Francisco, UCSF, San

Francisco, CA, USA.

LA - eng

PT - Case Reports

PT - Journal Article

DEP - 20241219

PL - England

TA - SAGE Open Med Case Rep

JT - SAGE open medical case reports

JID - 101638686

PMC - PMC11660058

OTO - NOTNLM

OT - ALK

OT - ALK1

OT - Inflammatory myofibroblastic tumor (IMT)

OT - urinary bladder

COIS- The author(s) declared no potential conflicts of interest with respect to the

research, authorship, and/or publication of this article.

EDAT- 2024/12/23 17:24

MHDA- 2024/12/23 17:25

PMCR- 2024/12/19

CRDT- 2024/12/23 06:26

PHST- 2023/12/25 00:00 [received]

PHST- 2024/12/05 00:00 [accepted]

PHST- 2024/12/23 17:25 [medline]

PHST- 2024/12/23 17:24 [pubmed]

PHST- 2024/12/23 06:26 [entrez]

PHST- 2024/12/19 00:00 [pmc-release]

AID - 10.1177_2050313X241308992 [pii]

AID - 10.1177/2050313X241308992 [doi]

PST - epublish

SO - SAGE Open Med Case Rep. 2024 Dec 19;12:2050313X241308992. doi:

10.1177/2050313X241308992. eCollection 2024.

PMID- 39712775

OWN - NLM

STAT- PubMed-not-MEDLINE

LR - 20250104

IS - 2168-8184 (Print)

IS - 2168-8184 (Electronic)

IS - 2168-8184 (Linking)

VI - 16

IP - 11

DP - 2024 Nov

TI - Asthma Symptoms Mimicking Myofibroblastic Tracheal Tumor in Pediatric Diagnosis.

PG - e74097

LID - 10.7759/cureus.74097 [doi]

LID - e74097

AB - Tracheal tumors in pediatric patients are rare, accounting for 2% of all airway

abnormalities and 0.2% of all pediatric tumors. Diagnosis is often delayed due to

the heterogeneity of presenting symptoms, such as stridor and wheezing, which are

frequently misattributed to other conditions. We report the case of a previously

healthy nine-year-old male who was diagnosed with an inflammatory myofibroblastic

tumor (IMT) following five months of persistent airway symptoms, including cough,

biphasic stridor, wheezing, and dyspnea. Despite evaluation by multiple

physicians and treatment for presumed asthma, his symptoms did not fully resolve.

Imaging studies ultimately confirmed the diagnosis, and surgical resection of the

tracheal tumor was performed. In the late postoperative period (12 weeks), the

patient continued to experience cough and dyspnea. Given a family history of

asthma (father with asthma), spirometry with a bronchodilator was conducted,

confirming a diagnosis of asthma alongside IMT. The patient is currently alive

and undergoing treatment in Step 2 of the Global Initiative for Asthma (GINA)

guidelines. This case highlights the importance of a thorough evaluation in

children with persistent stridor and wheezing to rule out underlying

tracheobronchial pathologies.

CI - Copyright © 2024, Figueroa-Hurtado et al.

FAU - Figueroa-Hurtado, Esperanza

AU - Figueroa-Hurtado E

AD - Respiratory Diseases Clinic, Regional Hospital of High Specialty of the Yucatan

Peninsula, Instituto Mexicano del Seguro Social-Bienestar, Merida, MEX.

FAU - Peña, Mario J

AU - Peña MJ

AD - Thoracic Surgery, Hospital Infantil de México Federico Gomez, Mexico City, MEX.

FAU - Cortes-Telles, Arturo

AU - Cortes-Telles A

AD - Respiratory Diseases Clinic, Regional Hospital of High Specialty of the Yucatan

Peninsula, Instituto Mexicano del Seguro Social-Bienestar, Merida, MEX.

LA - eng

PT - Case Reports

PT - Journal Article

DEP - 20241120

PL - United States

TA - Cureus

JT - Cureus

JID - 101596737

PMC - PMC11661893

OTO - NOTNLM

OT - asthma

OT - pediatric

OT - stridor

OT - tracheal tumor

OT - wheezing

COIS- Human subjects: Consent for treatment and open access publication was obtained or

waived by all participants in this study. Conflicts of interest: In compliance

with the ICMJE uniform disclosure form, all authors declare the following:

Payment/services info: All authors have declared that no financial support was

received from any organization for the submitted work. Financial relationships:

All authors have declared that they have no financial relationships at present or

within the previous three years with any organizations that might have an

interest in the submitted work. Other relationships: All authors have declared

that there are no other relationships or activities that could appear to have

influenced the submitted work.

EDAT- 2024/12/23 17:24

MHDA- 2024/12/23 17:25

PMCR- 2024/11/20

CRDT- 2024/12/23 06:10

PHST- 2024/11/19 00:00 [accepted]

PHST- 2024/12/23 17:25 [medline]

PHST- 2024/12/23 17:24 [pubmed]

PHST- 2024/12/23 06:10 [entrez]

PHST- 2024/11/20 00:00 [pmc-release]

AID - 10.7759/cureus.74097 [doi]

PST - epublish

SO - Cureus. 2024 Nov 20;16(11):e74097. doi: 10.7759/cureus.74097. eCollection 2024

Nov.

PMID- 39703852

OWN - NLM

STAT- PubMed-not-MEDLINE

LR - 20250104

IS - 2234-943X (Print)

IS - 2234-943X (Electronic)

IS - 2234-943X (Linking)

VI - 14

DP - 2024

TI - Rare giant epithelioid inflammatory myofibroblastic sarcoma of the abdominal

cavity in a child: a case report and review of the literature.

PG - 1417918

LID - 10.3389/fonc.2024.1417918 [doi]

LID - 1417918

AB - Epithelioid inflammatory myofibroblastic sarcoma (EIMS) is a distinct subtype of

inflammatory myofibroblastoma tumor (IMT) that is recognized as a rare malignant

tumor characterized by anaplastic lymphoma kinase (ALK) positivity, significant

aggressiveness, treatment challenges, and a poor prognosis. We report on the case

of an 8-year-old boy presenting with abdominal pain and vomiting. Computed

tomography (CT) of the abdomen revealed a large tumor, and the pathology results

following a biopsy confirmed the diagnosis of EIMS. The patient underwent radical

tumor resection, and genetic testing identified the presence of the RANBP2-ALK

fusion. To our knowledge, this represents the largest pediatric case of abdominal

EIMS documented in the literature. Currently, there is no standard therapy for

EIMS; however, existing studies advocate for the use of ALK tyrosine kinase

inhibitors (TKIs) in its treatment. This case was reported to be in remission

following treatment with crizotinib, thereby contributing to the understanding of

the specific pathology of EIMS and facilitating accurate diagnosis and targeted

therapy.

CI - Copyright © 2024 Li, Su, Zhang, Chen, Hou and Cheng.

FAU - Li, Jinzhou

AU - Li J

AD - Department of General Surgery, Xi'an Children's Hospital/Children's Hospital

Affiliated to Xi'an Jiaotong University, Xi'an, China.

FAU - Su, Haixing

AU - Su H

AD - Department of General Surgery, Xi'an Children's Hospital/Children's Hospital

Affiliated to Xi'an Jiaotong University, Xi'an, China.

FAU - Zhang, Sheng

AU - Zhang S

AD - Department of General Surgery, Xi'an Children's Hospital/Children's Hospital

Affiliated to Xi'an Jiaotong University, Xi'an, China.

FAU - Chen, Xianyun

AU - Chen X

AD - Department of General Surgery, Xi'an Children's Hospital/Children's Hospital

Affiliated to Xi'an Jiaotong University, Xi'an, China.

FAU - Hou, Chongzhi

AU - Hou C

AD - Department of General Surgery, Xi'an Children's Hospital/Children's Hospital

Affiliated to Xi'an Jiaotong University, Xi'an, China.

FAU - Cheng, Tao

AU - Cheng T

AD - Department of General Surgery, Xi'an Children's Hospital/Children's Hospital

Affiliated to Xi'an Jiaotong University, Xi'an, China.

LA - eng

PT - Case Reports

PT - Journal Article

DEP - 20241205

PL - Switzerland

TA - Front Oncol

JT - Frontiers in oncology

JID - 101568867

PMC - PMC11655461

OTO - NOTNLM

OT - anaplastic lymphoma kinase

OT - childhood tumor

OT - crizotinib

OT - epithelioid inflammatory myofibroblastic sarcoma

OT - targeted therapy

COIS- The authors declare that the research was conducted in the absence of any

commercial or financial relationships that could be construed as a potential

conflict of interest.

EDAT- 2024/12/20 06:23

MHDA- 2024/12/20 06:24

PMCR- 2024/01/01

CRDT- 2024/12/20 04:16

PHST- 2024/07/15 00:00 [received]

PHST- 2024/11/13 00:00 [accepted]

PHST- 2024/12/20 06:24 [medline]

PHST- 2024/12/20 06:23 [pubmed]

PHST- 2024/12/20 04:16 [entrez]

PHST- 2024/01/01 00:00 [pmc-release]

AID - 10.3389/fonc.2024.1417918 [doi]

PST - epublish

SO - Front Oncol. 2024 Dec 5;14:1417918. doi: 10.3389/fonc.2024.1417918. eCollection

2024.

PMID- 39701088

OWN - NLM

STAT- Publisher

LR - 20241219

IS - 2005-9256 (Electronic)

IS - 1598-2998 (Linking)

DP - 2024 Dec 18

TI - ALK Inhibition in a Patient with Inflammatory Myofibroblastic Tumor Harboring

CARS1-ALK Fusion.

LID - 10.4143/crt.2024.1184 [doi]

AB - Inflammatory myofibroblastic tumor (IMT) is a rare entity, primarily affecting

young individuals, often involving the abdomen, pelvis, or lung. Approximately

50% of IMTs harbor ALK gene rearrangements, making ALK inhibitors a viable

treatment. We report a case of a 40-year-old female with metastatic IMT harboring

a CARS1-ALK fusion. Initial chemotherapy failed, but targeted therapy with

alectinib through the KOrean Precision Medicine Networking Group Study of

MOlecular profiling guided therapy based on genomic alterations in advanced Solid

tumors (KOSMOS)-II study led to significant tumor regression and ongoing, durable

clinical improvement of 19 months. This case highlights the importance of

precision medicine and raises the reappraisal of targeted agents outside of

approved indications for rare cancers with actionable genomic alterations.

FAU - Choi, Songji

AU - Choi S

AD - Department of Genomic Medicine, Seoul National University Hospital, Seoul, Korea.

FAU - Kim, Miso

AU - Kim M

AD - Department of Internal Medicine, Seoul National University Hospital, Seoul,

Korea.

FAU - Kim, Sheehyun

AU - Kim S

AD - Department of Genomic Medicine, Seoul National University Hospital, Seoul, Korea.

FAU - Park, Taekeun

AU - Park T

AD - Department of Internal Medicine, Seoul National University Hospital, Seoul,

Korea.

FAU - Kwak, Yoonjin

AU - Kwak Y

AD - Department of Pathology, Seoul National University Hospital, Seoul, Korea.

FAU - Bae, Jeong Mo

AU - Bae JM

AD - Department of Pathology, Seoul National University Hospital, Seoul, Korea.

FAU - Yun, Hongseok

AU - Yun H

AD - Department of Genomic Medicine, Seoul National University Hospital, Seoul, Korea.

FAU - Kim, Jee Hyun

AU - Kim JH

AD - Department of Internal Medicine, Department of Genomic Medicine, Seoul National

University Bundang Hospital, Seoul National University College of Medicine,

Seongnam, Korea.

LA - eng

PT - Case Reports

PT - Journal Article

DEP - 20241218

PL - Korea (South)

TA - Cancer Res Treat

JT - Cancer research and treatment

JID - 101155137

SB - IM

OTO - NOTNLM

OT - ALK fusion

OT - Alectinib

OT - Inflammatory myofibroblastic tumor

OT - Precision medicine

EDAT- 2024/12/20 00:23

MHDA- 2024/12/20 00:23

CRDT- 2024/12/19 18:35

PHST- 2024/12/10 00:00 [received]

PHST- 2024/12/14 00:00 [accepted]

PHST- 2024/12/20 00:23 [medline]

PHST- 2024/12/20 00:23 [pubmed]

PHST- 2024/12/19 18:35 [entrez]

AID - crt.2024.1184 [pii]

AID - 10.4143/crt.2024.1184 [doi]

PST - aheadofprint

SO - Cancer Res Treat. 2024 Dec 18. doi: 10.4143/crt.2024.1184.

PMID- 39688771

OWN - NLM

STAT- Publisher

LR - 20241217

IS - 1876-7931 (Electronic)

IS - 1876-7931 (Linking)

DP - 2024 Dec 17

TI - A case report of hepatic inflammatory myofibroblastic tumor in a pediatric

patient: diagnostic challenges and management strategies.

LID - 10.1007/s40477-024-00975-9 [doi]

AB - PURPOSE: The aim of this study is to present a case of inflammatory

myofibroblastic tumor (IMT) of the liver in a 4-year-old girl. We will discuss

the diagnostic challenges, the role of the radiologist in differential diagnosis,

treatment modalities, and clinical outcomes. METHODS: A case report of a

4-year-old girl with IMT of the liver is presented. We will review the patient's

clinical presentation, diagnostic imaging findings, histological features,

treatment, and follow-up. RESULTS: The patient presented with vomiting and

jaundice, but no fever. Imaging studies revealed an hepatic lesion suspicious for

IMT; pathology confirmed the diagnosis. The patient underwent surgical

intervention with successful resolution. CONCLUSION: IMT of the liver is a rare

but important diagnosis to consider in children with abdominal masses.

Radiologists play a crucial role in differential diagnosis. Surgical resection

can be an effective treatment option for IMT, leading to favorable clinical

outcomes.

CI - © 2024. Società Italiana di Ultrasonologia in Medicina e Biologia (SIUMB).

FAU - Napolitano, Marcello

AU - Napolitano M

AD - Department of Pediatric Radiology and Neuroradiology, V. Buzzi Children's

Hospital, Milan, Italy.

FAU - Lama, Beatrice

AU - Lama B

AUID- ORCID: 0009-0009-3477-8236

AD - Department of Advanced Biomedical Sciences, University of Naples "Federico II",

80131, Naples, Italy. beatrice.lama1196@gmail.com.

FAU - Ierardi, Anna Maria

AU - Ierardi AM

AD - Department of Diagnostic and Interventional Radiology, Foundation IRCCS Cà

Granda-Ospedale Maggiore Policlinico, 20122, Milan, Italy.

FAU - Valle, Clarissa

AU - Valle C

AD - Department of Radiology, ASST Papa Giovanni XXIII, Piazza OMS, 24127, Bergamo,

BG, Italy.

FAU - Rossi, Eugenio

AU - Rossi E

AD - U.O.S.D. Diagnostica per Immagini A.O.R.N. Santobono-Pausilipon, Pausilipon

Hospital, Naples, Italy.

FAU - Minelli, Rocco

AU - Minelli R

AD - Department of Medicine and Health Sciences "Vincenzo Tiberio", University of

Molise, Via Francesco De Sanctis, 1, 86100, Campobasso, CB, Italy.

FAU - Paviglianiti, Giuseppe

AU - Paviglianiti G

AD - UOC Radiologia Pediatrica ARNASCivico-Di Cristina-Benfratelli, Palermo, Italy.

FAU - Di Rosa, Gianpaolo

AU - Di Rosa G

AD - Città della salute e della scienza di Torino Presidio Regina Margherita S. C.

Radiologia Pediatrica, Turin, Italy.

FAU - Baldazzi, Michelangelo

AU - Baldazzi M

AD - Pediatric and Adult CardioThoracic and Vascular, Oncohematologic and Emergency

Radiology Unit IRCCS Azienda Ospedaliero, Universitaria Di Bologna, Bologna,

Italy.

FAU - Carrafiello, Gian Paolo

AU - Carrafiello GP

AD - Department of Diagnostic and Interventional Radiology, Foundation IRCCS Cà

Granda-Ospedale Maggiore Policlinico, 20122, Milan, Italy.

LA - eng

PT - Journal Article

DEP - 20241217

PL - Italy

TA - J Ultrasound

JT - Journal of ultrasound

JID - 101315005

SB - IM

OTO - NOTNLM

OT - CT

OT - Inflammatory myofibroblastic tumor

OT - Liver

OT - MRI

OT - Pediatric

OT - Ultrasonography

COIS- Declarations. Conflict of interest: The authors have no relevant financial or

non-financial interests to disclose. Informed consent: Informed consent was

obtained from all individual participants included in the study.

EDAT- 2024/12/17 12:30

MHDA- 2024/12/17 12:30

CRDT- 2024/12/17 11:18

PHST- 2024/10/01 00:00 [received]

PHST- 2024/11/03 00:00 [accepted]

PHST- 2024/12/17 12:30 [medline]

PHST- 2024/12/17 12:30 [pubmed]

PHST- 2024/12/17 11:18 [entrez]

AID - 10.1007/s40477-024-00975-9 [pii]

AID - 10.1007/s40477-024-00975-9 [doi]

PST - aheadofprint

SO - J Ultrasound. 2024 Dec 17. doi: 10.1007/s40477-024-00975-9.

PMID- 26389361

STAT- Publisher

CTDT- 20241217

PB - National Cancer Institute (US)

DP - 2002

TI - Childhood Soft Tissue Sarcoma Treatment (PDQ®): Health Professional Version.

BTI - PDQ Cancer Information Summaries

AB - This PDQ cancer information summary for health professionals provides

comprehensive, peer-reviewed, evidence-based information about the treatment of

childhood soft tissue sarcoma. It is intended as a resource to inform and assist

clinicians in the care of their patients. It does not provide formal guidelines

or recommendations for making health care decisions. This summary is reviewed

regularly and updated as necessary by the PDQ Pediatric Treatment Editorial

Board, which is editorially independent of the National Cancer Institute (NCI).

The summary reflects an independent review of the literature and does not

represent a policy statement of NCI or the National Institutes of Health (NIH).

CN - PDQ Pediatric Treatment Editorial Board

LA - eng

PT - Review

PT - Book Chapter

PL - Bethesda (MD)

OTO - NOTNLM

OT - childhood alveolar soft-part sarcoma

OT - childhood angiosarcoma

OT - childhood desmoplastic small round cell tumor

OT - childhood fibrosarcoma

OT - childhood leiomyosarcoma

OT - childhood liposarcoma

OT - childhood neurofibrosarcoma

OT - childhood soft tissue sarcoma

OT - childhood synovial sarcoma

OT - clear cell sarcoma of soft tissue

OT - dermatofibrosarcoma protuberans

OT - desmoid-type fibromatosis

OT - ectomesenchymoma

OT - epithelioid hemangioendothelioma

OT - epithelioid sarcoma

OT - extraskeletal mesenchymal chondrosarcoma

OT - extraskeletal myxoid chondrosarcoma

OT - extraskeletal osteosarcoma

OT - infantile fibrosarcoma

OT - infantile myofibromatosis

OT - inflammatory myofibroblastic tumor

OT - low-grade fibromyxoid sarcoma

OT - malignant peripheral nerve sheath tumor

OT - malignant triton tumor

OT - myopericytoma

OT - myxofibrosarcoma

OT - PEComa

OT - plexiform fibrohistiocytic tumor

OT - sclerosing epithelioid fibrosarcoma

OTO - NLM

OT - childhood soft tissue sarcoma

OT - childhood alveolar soft-part sarcoma

OT - childhood angiosarcoma

OT - childhood desmoplastic small round cell tumor

OT - childhood fibrosarcoma

OT - childhood leiomyosarcoma

OT - childhood liposarcoma

OT - childhood neurofibrosarcoma

OT - childhood synovial sarcoma

OT - dermatofibrosarcoma protuberans

EDAT- 2024/12/17 00:00

CRDT- 2024/12/17 00:00

AID - NBK65923 [bookaccession]

PMID- 39661077

OWN - NLM

STAT- Publisher

LR - 20241211

IS - 2366-0058 (Electronic)

DP - 2024 Dec 11

TI - CT and MRI characteristics of inflammatory pseudotumor-like follicular dendritic

cell sarcoma of the spleen: a report of 11 patients with pathological

correlation.

LID - 10.1007/s00261-024-04736-4 [doi]

AB - OBJECTIVE: This study aimed to outline the computed tomography (CT) and magnetic

resonance imaging (MRI) characteristics of inflammatory pseudotumor-like

follicular dendritic cell sarcoma (IPT-like FDCS) in the spleen and to link these

imaging features with histopathological findings. MATERIALS AND METHODS: A

retrospective analysis was conducted on 11 patients (3 males, 8 females; mean

age, 63.3 ± 9.66 years; age range, 44-76 years; median age, 62 years) with

histopathologically confirmed inflammatory pseudotumor-like follicular dendritic

cell sarcoma of the spleen (IPT-like FDCS). The study encompassed a comprehensive

review of initial clinical manifestations, macroscopic pathological alterations,

along with histopathological and corresponding immunohistochemical findings. CT

(n = 10) and MRI (n = 8) characteristics were meticulously assessed by two

experienced radiologists, who evaluated parameters including lesion number, size,

shape, margin definition, capsular integrity, attenuation, signal intensity,

presence of hemosiderin deposition, and patterns of contrast enhancement.

RESULTS: In this study, all eleven patients (11/11; 100%) presented with solitary

inflammatory pseudotumor-like follicular dendritic cell sarcoma (IPT-like FDCS).

The majority of these cases were incidentally detected during routine physical

examinations, while one case was revealed through abdominal CT following a

decline in platelet count post-radiation and chemotherapy for prostate cancer.

The splenic lesions were predominantly oval in shape (10/11; 91%), with a single

case exhibiting a lobulated appearance (1/11; 9%). The largest lesion diameter

was 18 cm, with diameters ranging from 3.3 to 18 cm, averaged 6.5 cm, and a

median diameter of 5.0 cm. On plain CT images, the lesions were primarily

observed as hypodense with poorly defined margins. On contrast-enhanced CT, all

lesions (10/10; 100%) promptly became sharply demarcated in the arterial phase,

encircled by a perceptible, relatively hypodense capsule-like rim. On T2-weighted

MRI, all lesions (8/8; 100%) displayed a characteristic rim-like low-intensity

capsular margin. These low-signal/density capsular margins were pathologically

confirmed to be capsules containing fibrous components. CONCLUSIONS: IPT-like

FDCS is very rare but has characteristic imaging features that correlate with its

pathology.

CI - © 2024. The Author(s), under exclusive licence to Springer Science+Business

Media, LLC, part of Springer Nature.

FAU - Liu, Chang

AU - Liu C

AD - Department of Radiology, The First Affiliated Hospital, Zhejiang University

School of Medicine, No.79, Qingchun Road, Hangzhou, Zhejiang Province, China.

FAU - Li, Yun-Yun

AU - Li YY

AD - Department of Pathology, The First Affiliated Hospital, Zhejiang University

School of Medicine, No.79, Qingchun Road, Hangzhou, Zhejiang Province, China.

FAU - Zhu, Xian-Di

AU - Zhu XD

AD - Department of Nuclear Medicine, Tongde Hospital of Zhejiang Province, No.234,

Gucui Road, Hangzhou, Zhejiang Province, China.

FAU - Xiang, Xue-Lian

AU - Xiang XL

AD - Department of Radiology, Tongde Hospital of Zhejiang Province, No.234, Gucui

Road, Hangzhou, Zhejiang Province, China. xxl18106535713@163.com.

LA - eng

PT - Journal Article

DEP - 20241211

PL - United States

TA - Abdom Radiol (NY)

JT - Abdominal radiology (New York)

JID - 101674571

SB - IM

OTO - NOTNLM

OT - Computed tomography (CT)

OT - Histopathology

OT - Inflammatory pseudotumor-like follicular dendritic cell sarcoma (IPT-like FDCS)

OT - Magnetic resonance imaging (MRI)

COIS- Declarations. Conflict of interest: The authors declare no competing interests.

EDAT- 2024/12/11 12:34

MHDA- 2024/12/11 12:34

CRDT- 2024/12/11 11:02

PHST- 2024/10/15 00:00 [received]

PHST- 2024/11/30 00:00 [accepted]

PHST- 2024/11/28 00:00 [revised]

PHST- 2024/12/11 12:34 [medline]

PHST- 2024/12/11 12:34 [pubmed]

PHST- 2024/12/11 11:02 [entrez]

AID - 10.1007/s00261-024-04736-4 [pii]

AID - 10.1007/s00261-024-04736-4 [doi]

PST - aheadofprint

SO - Abdom Radiol (NY). 2024 Dec 11. doi: 10.1007/s00261-024-04736-4.

PMID- 35412727

STAT- Publisher

CTDT- 20241203

PB - National Cancer Institute (US)

DP - 2002

TI - Childhood Pulmonary Inflammatory Myofibroblastic Tumors Treatment (PDQ®): Health

Professional Version.

BTI - PDQ Cancer Information Summaries

AB - This PDQ cancer information summary for health professionals provides

comprehensive, peer-reviewed, evidence-based information about the treatment of

childhood pulmonary inflammatory myofibroblastic tumors. It is intended as a

resource to inform and assist clinicians in the care of their patients. It does

not provide formal guidelines or recommendations for making health care

decisions. This summary is reviewed regularly and updated as necessary by the PDQ

Pediatric Treatment Editorial Board, which is editorially independent of the

National Cancer Institute (NCI). The summary reflects an independent review of

the literature and does not represent a policy statement of NCI or the National

Institutes of Health (NIH).

CN - PDQ Pediatric Treatment Editorial Board

LA - eng

PT - Review

PT - Book Chapter

PL - Bethesda (MD)

OTO - NOTNLM

OT - Lung cancer

OT - Pediatric lung cancer

OT - Pulmonary inflammatory myofibroblastic tumors

OTO - NLM

OT - lung cancer

EDAT- 2024/12/03 00:00

CRDT- 2024/12/03 00:00

AID - NBK579422 [bookaccession]

PMID- 39615002

OWN - NLM

STAT- MEDLINE

DCOM- 20241130

LR - 20241130

IS - 1433-0350 (Electronic)

IS - 0256-7040 (Linking)

VI - 41

IP - 1

DP - 2024 Nov 30

TI - Inflammatory myofibroblastic tumor in the intradural extramedullary space of the

lumbosacral spine: a case report and review of the literature.

PG - 24

LID - 10.1007/s00381-024-06690-4 [doi]

AB - Inflammatory myofibroblastic tumor (IMT) is a rare, benign lesion of uncertain

etiology, predominantly affecting soft tissues such as the lungs, with spinal

involvement being exceedingly rare. We present the case of a 10-year-old male

with a year-long history of low back pain, constipation, and difficulty in

urination. MRI revealed an intradural extramedullary lesion at L5-S1, initially

suspected to be a schwannoma or neurofibroma. The patient underwent L5

laminectomy and near-total excision of the tumor. Histopathological examination

confirmed IMT, characterized by spindle cells and dense inflammatory

infiltration. At 18 months follow-up, MRI showed no tumor progression with

complete clinical improvement. This is the first reported case of IMT in

pediatric age, affecting the lumbosacral spine. It highlights the importance of

considering IMT in the differential diagnosis of spinal tumors, particularly when

multiple nerve roots are involved, with intraoperative findings of an

inflammatory lesion. Surgery remains the definitive treatment.

CI - © 2024. The Author(s), under exclusive licence to Springer-Verlag GmbH Germany,

part of Springer Nature.

FAU - Singh, Guramritpal

AU - Singh G

AD - Sanjay Gandhi Post Graduate Institute of Medical Sciences, Lucknow, India.

FAU - Kumar, Ashutosh

AU - Kumar A

AD - Sanjay Gandhi Post Graduate Institute of Medical Sciences, Lucknow, India.

draashutoshkumarr@gmail.com.

FAU - J, Anantha Chaitanya

AU - J AC

AD - Sanjay Gandhi Post Graduate Institute of Medical Sciences, Lucknow, India.

FAU - Jaiswal, Sushila

AU - Jaiswal S

AD - Sanjay Gandhi Post Graduate Institute of Medical Sciences, Lucknow, India.

FAU - Verma, Pawan

AU - Verma P

AD - Sanjay Gandhi Post Graduate Institute of Medical Sciences, Lucknow, India.

FAU - Mehrotra, Anant

AU - Mehrotra A

AD - Sanjay Gandhi Post Graduate Institute of Medical Sciences, Lucknow, India.

FAU - Jaiswal, Awadhesh

AU - Jaiswal A

AD - Sanjay Gandhi Post Graduate Institute of Medical Sciences, Lucknow, India.

LA - eng

PT - Case Reports

PT - Journal Article

PT - Review

DEP - 20241130

PL - Germany

TA - Childs Nerv Syst

JT - Child's nervous system : ChNS : official journal of the International Society for

Pediatric Neurosurgery

JID - 8503227

SB - IM

MH - Humans

MH - Male

MH - Child

MH - *Magnetic Resonance Imaging

MH - Spinal Cord Neoplasms/surgery/diagnostic imaging/pathology

MH - Granuloma, Plasma Cell/surgery/diagnostic imaging/pathology

MH - Lumbosacral Region/surgery

MH - Lumbar Vertebrae/surgery/diagnostic imaging

MH - Laminectomy/methods

MH - Diagnosis, Differential

OTO - NOTNLM

OT - Inflammatory

OT - Intradural extramedullary tumor

OT - Myofibroblastic tumor

OT - Pediatric

COIS- Declarations. Informed consent: The patient consented to the publication in the

journal. Conflict of interest: The authors declare no competing interests.

EDAT- 2024/12/01 15:22

MHDA- 2024/12/01 15:23

CRDT- 2024/11/30 11:14

PHST- 2024/09/30 00:00 [received]

PHST- 2024/11/22 00:00 [accepted]

PHST- 2024/12/01 15:23 [medline]

PHST- 2024/12/01 15:22 [pubmed]

PHST- 2024/11/30 11:14 [entrez]

AID - 10.1007/s00381-024-06690-4 [pii]

AID - 10.1007/s00381-024-06690-4 [doi]

PST - epublish

SO - Childs Nerv Syst. 2024 Nov 30;41(1):24. doi: 10.1007/s00381-024-06690-4.

PMID- 39592917

OWN - NLM

STAT- MEDLINE

DCOM- 20250113

LR - 20250116

IS - 1759-7714 (Electronic)

IS - 1759-7706 (Print)

IS - 1759-7706 (Linking)

VI - 16

IP - 1

DP - 2025 Jan

TI - Clinicopathological Characteristics of Inflammatory Myofibroblastic Tumor: A

Single Center Retrospective Cohort Study.

PG - e15496

LID - 10.1111/1759-7714.15496 [doi]

LID - e15496

AB - BACKGROUND: Inflammatory myofibroblastic tumor (IMT) is a rare intermediate-grade

neoplasm. It presents a great challenge in diagnosis and treatment. This study

aims to identify the clinicopathological characteristics of IMT. METHODS: A

retrospective study was conducted, enrolling patients with IMT at Peking Union

Medical College Hospital from January 2013 to October 2023. Clinical information,

treatments, and efficacy were analyzed. RESULTS: A total of 72 patients were

enrolled, including 38 men and 34 women, with a median age of 46.5 years. The

most common primary site included the lung (n = 15, 20.8%), intestinal tract

(n = 8, 11.1%), abdominal cavity (n = 7, 9.7%), and nasal sinus (n = 5, 6.9%).

Thirty patients harbored anaplastic lymphoma kinase (ALK) fusion genes;

Sixty-five (90.3%) patients underwent surgical resection, and 11 of them had

postoperative recurrence. Thirty patients received systemic therapy, including

nonsteroidal anti-inflammatory drugs (n = 1), steroids (n = 5), chemotherapy

(n = 7), targeted therapy (n = 2), and immune checkpoint inhibitor (n = 1).

CONCLUSIONS: The most common site of IMT is the lung. Surgery is the main

treatment for IMT, and postoperative adjuvant therapy for ALK-positive patients

needs to be focused. The molecular testing is essential for all patients

diagnosed with IMTs. Systemic treatment needs further research.

CI - © 2024 The Author(s). Thoracic Cancer published by John Wiley & Sons Australia,

Ltd.

FAU - Si, Xiaoyan

AU - Si X

AUID- ORCID: 0000-0003-2913-3045

AD - Department of Pulmonary and Critical Care Medicine, Peking Union Medical College

Hospital, Chinese Academy of Medical Sciences, Peking Union Medical College,

Beijing, China.

FAU - Wu, Shafei

AU - Wu S

AD - Department of Pathology, Peking Union Medical College Hospital, Chinese Academy

of Medical Sciences, Peking Union Medical College, Beijing, China.

FAU - Feng, Ruie

AU - Feng R

AUID- ORCID: 0000-0002-1609-9901

AD - Department of Pathology, Peking Union Medical College Hospital, Chinese Academy

of Medical Sciences, Peking Union Medical College, Beijing, China.

FAU - Wang, Mengzhao

AU - Wang M

AD - Department of Pulmonary and Critical Care Medicine, Peking Union Medical College

Hospital, Chinese Academy of Medical Sciences, Peking Union Medical College,

Beijing, China.

FAU - Wang, Hanping

AU - Wang H

AUID- ORCID: 0000-0003-3540-2280

AD - Department of Pulmonary and Critical Care Medicine, Peking Union Medical College

Hospital, Chinese Academy of Medical Sciences, Peking Union Medical College,

Beijing, China.

FAU - Zhang, Xiaotong

AU - Zhang X

AUID- ORCID: 0000-0001-8937-820X

AD - Department of Pulmonary and Critical Care Medicine, Peking Union Medical College

Hospital, Chinese Academy of Medical Sciences, Peking Union Medical College,

Beijing, China.

FAU - Zhang, Li

AU - Zhang L

AUID- ORCID: 0000-0002-8101-672X

AD - Department of Pulmonary and Critical Care Medicine, Peking Union Medical College

Hospital, Chinese Academy of Medical Sciences, Peking Union Medical College,

Beijing, China.

FAU - Xu, Kaifeng

AU - Xu K

AD - Department of Pulmonary and Critical Care Medicine, Peking Union Medical College

Hospital, Chinese Academy of Medical Sciences, Peking Union Medical College,

Beijing, China.

LA - eng

GR - 2022-PUMCH-B-107/National High-Level Hospital Clinical Research Funding/

PT - Journal Article

DEP - 20241126

PL - Singapore

TA - Thorac Cancer

JT - Thoracic cancer

JID - 101531441

SB - IM

MH - Humans

MH - Male

MH - Female

MH - Retrospective Studies

MH - Middle Aged

MH - Adult

MH - *Neoplasms, Muscle Tissue/pathology

MH - Aged

MH - Young Adult

MH - Adolescent

MH - Granuloma, Plasma Cell/pathology

PMC - PMC11729751

OTO - NOTNLM

OT - anaplastic lymphoma kinase

OT - chemotherapy

OT - inflammatory myofibroblastic tumor

OT - targeted therapy

COIS- The authors declare no conflicts of interest.

EDAT- 2024/11/27 00:21

MHDA- 2025/01/14 00:20

PMCR- 2024/11/26

CRDT- 2024/11/26 23:51

PHST- 2024/10/11 00:00 [revised]

PHST- 2024/07/16 00:00 [received]

PHST- 2024/11/11 00:00 [accepted]

PHST- 2025/01/14 00:20 [medline]

PHST- 2024/11/27 00:21 [pubmed]

PHST- 2024/11/26 23:51 [entrez]

PHST- 2024/11/26 00:00 [pmc-release]

AID - TCA15496 [pii]

AID - 10.1111/1759-7714.15496 [doi]

PST - ppublish

SO - Thorac Cancer. 2025 Jan;16(1):e15496. doi: 10.1111/1759-7714.15496. Epub 2024 Nov

26.

PMID- 39574496

OWN - NLM

STAT- PubMed-not-MEDLINE

LR - 20241123

IS - 1758-8340 (Print)

IS - 1758-8359 (Electronic)

IS - 1758-8340 (Linking)

VI - 16

DP - 2024

TI - Epithelioid inflammatory myofibroblastic sarcoma with exceptionally long response

to lorlatinib-a case report.

PG - 17588359241298489

LID - 10.1177/17588359241298489 [doi]

LID - 17588359241298489

AB - Epithelioid inflammatory myofibroblastic sarcoma (EIMS) is a rare and aggressive

subtype of inflammatory myofibroblastic tumor. The disease is associated with

rearrangements of the anaplastic lymphoma kinase (ALK). In this paper, we present

the clinicopathological features and treatment of a female patient diagnosed with

EIMS. In 2019, an 18-year-old female patient was admitted to the hospital with

abdominal pain. Radiological examinations confirmed a large pelvic mass which was

subsequently resected. After re-evaluation of the initial histologic diagnosis,

the final diagnosis of EIMS was established. Consequently, due to the lack of

response to chemotherapy and deteriorating clinical condition, she began the

therapy with ALK inhibitors. In total, the patient was treated with crizotinib,

alectinib, and lorlatinib. As a result, after over 4 years since the initial

diagnosis, she is still alive with significantly improved clinical condition and

quality of life. This paper demonstrates the clinical benefits of sequential

therapy of ALK inhibitors and an exceptionally long response to lorlatinib, a

third-generation tyrosine kinase inhibitor.

CI - © The Author(s), 2024.

FAU - Becht, Rafał

AU - Becht R

AUID- ORCID: 0000-0003-4820-0629

AD - Rafał Becht Department of Clinical Oncology, Chemotherapy and Cancer

Immunotherapy, Pomeranian Medical University in Szczecin, Unii Lubelskiej 1,

Szczecin 71-252, Poland.

FAU - Kiełbowski, Kajetan

AU - Kiełbowski K

AD - Department of Clinical Oncology, Chemotherapy and Cancer Immunotherapy,

Pomeranian Medical University in Szczecin, Szczecin, Poland.

FAU - Żychowska, Justyna

AU - Żychowska J

AUID- ORCID: 0009-0007-3477-3430

AD - Department of Clinical Oncology, Chemotherapy and Cancer Immunotherapy,

Pomeranian Medical University in Szczecin, Szczecin, Poland.

FAU - Poncyljusz, Wojciech

AU - Poncyljusz W

AD - Department of Diagnostic Imaging and Interventional Radiology, Pomeranian Medical

University in Szczecin, Szczecin, Poland.

FAU - Łanocha, Aleksandra

AU - Łanocha A

AD - Department of Hematology and Transplantology, Pomeranian Medical University in

Szczecin, Szczecin, Poland.

FAU - Kozak, Katarzyna

AU - Kozak K

AD - Department of Soft Tissue/Bone Sarcoma and Melanoma, The Maria Sklodowska-Curie

National Research Institute of Oncology, Warsaw, Poland.

FAU - Gabrysz-Trybek, Ewa

AU - Gabrysz-Trybek E

AD - Department of Diagnostic Imaging and Interventional Radiology, Pomeranian Medical

University in Szczecin, Szczecin, Poland.

FAU - Domagała, Paweł

AU - Domagała P

AD - Department of Pathology, Pomeranian Medical University in Szczecin, Szczecin,

Poland.

LA - eng

PT - Case Reports

PT - Journal Article

DEP - 20241120

PL - England

TA - Ther Adv Med Oncol

JT - Therapeutic advances in medical oncology

JID - 101510808

PMC - PMC11580051

OTO - NOTNLM

OT - ALK inhibitors

OT - ALK rearrangement

OT - epithelioid inflammatory myofibroblastic sarcoma

OT - lorlatinib

COIS- The authors declare that there is no conflict of interest.

EDAT- 2024/11/22 06:22

MHDA- 2024/11/22 06:23

PMCR- 2024/11/20

CRDT- 2024/11/22 04:15

PHST- 2024/07/09 00:00 [received]

PHST- 2024/10/23 00:00 [accepted]

PHST- 2024/11/22 06:23 [medline]

PHST- 2024/11/22 06:22 [pubmed]

PHST- 2024/11/22 04:15 [entrez]

PHST- 2024/11/20 00:00 [pmc-release]

AID - 10.1177_17588359241298489 [pii]

AID - 10.1177/17588359241298489 [doi]

PST - epublish

SO - Ther Adv Med Oncol. 2024 Nov 20;16:17588359241298489. doi:

10.1177/17588359241298489. eCollection 2024.

PMID- 39565115

OWN - NLM

STAT- MEDLINE

DCOM- 20241120

LR - 20241120

IS - 1098-2264 (Electronic)

IS - 1045-2257 (Linking)

VI - 63

IP - 11

DP - 2024 Nov

TI - An Inflammatory Myofibroblastic Tumor With a Novel ALK(V1180L) Mutation Leading

to Acquired Resistance to Tyrosine Kinase Inhibitors.

PG - e70012

LID - 10.1002/gcc.70012 [doi]

AB - Inflammatory myofibroblastic tumor (IMT) is a rare mesenchymal neoplasm that can

locally recur and potentially metastasize. Approximately 50% of IMTs harbor

rearrangements in the gene encoding anaplastic lymphoma kinase (ALK), a receptor

tyrosine kinase that can be therapeutically targeted with tyrosine kinase

inhibitors (TKIs). With successful application of TKI in ALK-positive nonsmall

cell carcinoma (NSCLC), ALK inhibitors are often first-line treatments for

patients with unresectable or metastatic IMTs. Although acquired resistance to

these agents may develop, resistance mechanisms are sparsely reported for IMTs.

Here we report a case of a 71 year-old man with metastatic pulmonary IMT

harboring a DCTN1::ALK fusion that progressed during alectinib TKI treatment.

Whole exome sequencing of an enlarging metastatic lesion in right 4th rib

revealed a novel p.V1180L mutation in the ALK tyrosine kinase domain as the

mechanism of acquired resistance. To our knowledge, this is the first report of

acquired p. V1180L mutation in IMTs treated with TKIs. In cases of ALK-positive

IMTs that progress on TKI therapy, targeted sequencing for acquired ALK mutations

may inform clinical decisions to adopt second-line therapeutic strategies.

CI - © 2024 Wiley Periodicals LLC.

FAU - Sharpe, Brittney

AU - Sharpe B

AD - University of West Indies, Mona, Jamaica.

FAU - Green, Donald C

AU - Green DC

AD - Department of Pathology and Laboratory Medicine, Dartmouth-Hitchcock Medical

Center, Lebanon, New Hampshire, USA.

FAU - Tafe, Laura J

AU - Tafe LJ

AD - Department of Pathology and Laboratory Medicine, Dartmouth-Hitchcock Medical

Center, Lebanon, New Hampshire, USA.

AD - Geisel School of Medicine at Dartmouth, Hanover, New Hampshire, USA.

FAU - Wasp, Garrett T

AU - Wasp GT

AD - Department of Oncology, Dartmouth-Hitchcock Medical Center, Lebanon, New

Hampshire, USA.

FAU - Kerr, Darcy A

AU - Kerr DA

AD - Department of Pathology and Laboratory Medicine, Dartmouth-Hitchcock Medical

Center, Lebanon, New Hampshire, USA.

AD - Geisel School of Medicine at Dartmouth, Hanover, New Hampshire, USA.

FAU - Dashti, Nooshin K

AU - Dashti NK

AD - Department of Pathology and Laboratory Medicine, Dartmouth-Hitchcock Medical

Center, Lebanon, New Hampshire, USA.

AD - Geisel School of Medicine at Dartmouth, Hanover, New Hampshire, USA.

LA - eng

PT - Case Reports

PT - Journal Article

PL - United States

TA - Genes Chromosomes Cancer

JT - Genes, chromosomes & cancer

JID - 9007329

RN - EC 2.7.10.1 (Anaplastic Lymphoma Kinase)

RN - 0 (Protein Kinase Inhibitors)

RN - EC 2.7.10.1 (ALK protein, human)

RN - LIJ4CT1Z3Y (alectinib)

RN - 0 (Piperidines)

RN - 0 (Carbazoles)

RN - 0 (Tyrosine Kinase Inhibitors)

SB - IM

MH - Humans

MH - *Anaplastic Lymphoma Kinase/genetics/antagonists & inhibitors

MH - Male

MH - *Protein Kinase Inhibitors/therapeutic use

MH - *Drug Resistance, Neoplasm/genetics

MH - Aged

MH - Piperidines/therapeutic use

MH - Mutation

MH - Neoplasms, Muscle Tissue/genetics/drug therapy/pathology

MH - Lung Neoplasms/genetics/drug therapy/pathology

MH - Carbazoles/therapeutic use

MH - Tyrosine Kinase Inhibitors

OTO - NOTNLM

OT - ALK

OT - DCTN1

OT - IMT

OT - TKI

OT - alectinib

OT - c.3538G>C

OT - inflammatory myofibroblastic tumor

OT - p.V1180L

OT - resistance

OT - tyrosine kinase inhibitor

EDAT- 2024/11/20 12:25

MHDA- 2024/11/20 12:26

CRDT- 2024/11/20 09:03

PHST- 2024/09/17 00:00 [revised]

PHST- 2024/08/16 00:00 [received]

PHST- 2024/11/02 00:00 [accepted]

PHST- 2024/11/20 12:26 [medline]

PHST- 2024/11/20 12:25 [pubmed]

PHST- 2024/11/20 09:03 [entrez]

AID - 10.1002/gcc.70012 [doi]

PST - ppublish

SO - Genes Chromosomes Cancer. 2024 Nov;63(11):e70012. doi: 10.1002/gcc.70012.

PMID- 39544284

OWN - NLM

STAT- PubMed-not-MEDLINE

LR - 20241116

IS - 2090-6900 (Print)

IS - 2090-6919 (Electronic)

VI - 2024

DP - 2024

TI - A Rare Case of Inflammatory Myofibroblastic Tumor Mimicking Fibrous Adhesions

Resulting in Bowel Obstruction.

PG - 7782678

LID - 10.1155/2024/7782678 [doi]

LID - 7782678

AB - Inflammatory myofibroblastic tumor (IMT) is a rare mesenchymal tumors of unknown

etiology composed of myofibroblastic cells admixed with inflammatory cells.

Presented is a 72-year-old male hospitalized for severe abdominal pain and

hematochezia with onset of associated symptoms of fever and sweats a few hours

prior to abdominal pain. A computed tomography (CT) demonstrated left colonic

thickening interpreted as partial obstruction, gross adhesions, and ischemia. At

surgery, marked bowel ischemia from the distal transverse to proximal sigmoid

colon was seen with extensive gross adhesions. Histopathology revealed a

mesenteric mass chiefly composed of stellate-to-spindled myofibroblastic cells

and fibrous adhesions, intermixed with lymphocytes, histiocytes, and plasma

cells. The tumor was positive for desmin, smooth muscle actin, and keratin; tumor

staging, grade, and postsurgical follow-up were not completed as the patient

expired postoperatively. Illustrated is a rare pathologic mimic of ischemic

colitis with fibrous adhesions, IMT. Thus, it should not be assumed that fibrous

adhesions are always the etiology of obstruction when "adhesions" between

sections of bowel are noted radiologically or surgically.

CI - Copyright © 2024 Stephanie Washburn et al.

FAU - Washburn, Stephanie

AU - Washburn S

AD - Edward Via College of Osteopathic Medicine, Blacksburg, Virginia, USA.

FAU - Jessica Thomas, Raj

AU - Jessica Thomas R

AD - Department of Internal Medicine, Cleveland Clinic Akron General, Akron, Ohio,

USA.

FAU - Grider, Douglas

AU - Grider D

AUID- ORCID: 0000-0002-6346-094X

AD - Department of Internal Medicine, Dermatology Division, Carilion Clinic and

Virginia Tech Carilion School of Medicine, Roanoke, Virginia, USA.

AD - Department of Internal Medicine, Edward Via College of Osteopathic Medicine,

Blacksburg, Virginia, USA.

AD - Department of Basic Science Education, Virginia Tech Carilion School of Medicine,

Roanoke, Virginia, USA.

AD - Dominion Pathology Associates, Roanoke, Virginia, USA.

LA - eng

PT - Case Reports

PT - Journal Article

DEP - 20241106

PL - United States

TA - Case Rep Surg

JT - Case reports in surgery

JID - 101580191

PMC - PMC11561171

OTO - NOTNLM

OT - bowel obstruction

OT - inflammatory myofibroblastic tumor

OT - ischemic colitis

OT - mesenteric mass

COIS- The authors declare no conflicts of interest.

EDAT- 2024/11/15 06:26

MHDA- 2024/11/15 06:27

PMCR- 2024/11/06

CRDT- 2024/11/15 04:25

PHST- 2024/03/16 00:00 [received]

PHST- 2024/07/06 00:00 [revised]

PHST- 2024/10/09 00:00 [accepted]

PHST- 2024/11/15 06:27 [medline]

PHST- 2024/11/15 06:26 [pubmed]

PHST- 2024/11/15 04:25 [entrez]

PHST- 2024/11/06 00:00 [pmc-release]

AID - 10.1155/2024/7782678 [doi]

PST - epublish

SO - Case Rep Surg. 2024 Nov 6;2024:7782678. doi: 10.1155/2024/7782678. eCollection

2024.

PMID- 39532638

OWN - NLM

STAT- Publisher

LR - 20241112

IS - 0219-3108 (Electronic)

IS - 1015-9584 (Linking)

DP - 2024 Nov 11

TI - ALK-negative inflammatory myofibroblastic tumor of the lung: A case report.

LID - S1015-9584(24)02348-0 [pii]

LID - 10.1016/j.asjsur.2024.10.039 [doi]

FAU - Wang, Enze

AU - Wang E

AD - Department of Thoracic Surgery, The First Affiliated Hospital of Henan

Polytechnic University, The Second People's Hospital of Jiaozuo, Jiaozuo, China.

FAU - Lu, Jiabin

AU - Lu J

AD - Department of Thoracic Surgery, The First Affiliated Hospital of Henan

Polytechnic University, The Second People's Hospital of Jiaozuo, Jiaozuo, China.

FAU - Qiao, Chengrui

AU - Qiao C

AD - Department of Thoracic Surgery, The First Affiliated Hospital of Henan

Polytechnic University, The Second People's Hospital of Jiaozuo, Jiaozuo, China.

FAU - Guo, Jincheng

AU - Guo J

AD - Department of Thoracic Surgery, The First Affiliated Hospital of Henan

Polytechnic University, The Second People's Hospital of Jiaozuo, Jiaozuo, China.

Electronic address: acheng69102@hotmail.com.

LA - eng

PT - Letter

DEP - 20241111

PL - Netherlands

TA - Asian J Surg

JT - Asian journal of surgery

JID - 8900600

SB - IM

OTO - NOTNLM

OT - ALK-Negative

OT - Inflammatory myofibroblastic tumor

COIS- Declaration of competing interest The authors declare that they have no conflicts

of interest.

EDAT- 2024/11/13 13:49

MHDA- 2024/11/13 13:49

CRDT- 2024/11/12 22:00

PHST- 2024/09/04 00:00 [received]

PHST- 2024/10/07 00:00 [accepted]

PHST- 2024/11/13 13:49 [medline]

PHST- 2024/11/13 13:49 [pubmed]

PHST- 2024/11/12 22:00 [entrez]

AID - S1015-9584(24)02348-0 [pii]

AID - 10.1016/j.asjsur.2024.10.039 [doi]

PST - aheadofprint

SO - Asian J Surg. 2024 Nov 11:S1015-9584(24)02348-0. doi:

10.1016/j.asjsur.2024.10.039.

PMID- 39524388

OWN - NLM

STAT- PubMed-not-MEDLINE

LR - 20241114

IS - 2224-4344 (Print)

IS - 2224-4344 (Electronic)

IS - 2224-4336 (Linking)

VI - 13

IP - 10

DP - 2024 Oct 1

TI - Efficacy of core biopsies for diagnosing inflammatory myofibroblastic tumors in

pediatric patients: case series from a single tertiary referral center.

PG - 1799-1809

LID - 10.21037/tp-24-239 [doi]

AB - BACKGROUND: Inflammatory myofibroblastic tumors (IMTs) are rare, often

non-metastasizing neoplasms characterized by fibro/myofibroblastic spindle cells

with varying infiltrates of plasma cells, lymphocytes, and/or eosinophils.

Despite their generally indolent nature, IMTs can exhibit locally aggressive

behavior and a significant tendency for local recurrence, making complete

surgical resection the standard treatment approach. Accurate diagnosis can be

challenging due to the overlap in imaging features with more aggressive tumors,

necessitating preoperative biopsies to enable differential diagnosis and guide

treatment decisions. The complexity of distinguishing IMTs from other

malignancies underscores the importance of biopsy in establishing an accurate

diagnosis and planning appropriate management strategies. CASE DESCRIPTION: This

study presents the cases of four pediatric patients (three males, one female)

diagnosed with IMT, involving tumors located in the lung (one case), bladder (one

case), and liver (two cases). Initial minimally invasive biopsies, including a

US-guided tru-cut core biopsy and a percutaneous core biopsy in one case, as well

as endoscopic core biopsies in two other cases, yielded inconclusive results.

These initial procedures failed to provide definitive diagnostic information,

necessitating the use of more precise diagnostic techniques to achieve a

definitive histological diagnosis of IMT. CONCLUSIONS: The findings indicate that

when initial biopsy results are inconclusive in cases suspected to be IMT, more

precise diagnostic procedures may be necessary to secure a definitive diagnosis.

This highlights the need for careful consideration of alternative biopsy methods

to ensure accurate identification and effective management of IMT in pediatric

patients.

CI - 2024 AME Publishing Company. All rights reserved.

FAU - Pierucci, Ugo Maria

AU - Pierucci UM

AD - Department of Pediatric Surgery, "V. Buzzi" Children's Hospital, Milan, Italy.

FAU - Paraboschi, Irene

AU - Paraboschi I

AD - Department of Biomedical and Clinical Sciences, University of Milan, Milan,

Italy.

FAU - Ardenghi, Carlotta

AU - Ardenghi C

AD - Department of Pediatric Surgery, "V. Buzzi" Children's Hospital, Milan, Italy.

FAU - Viglio, Camilla

AU - Viglio C

AD - Department of Pediatric Surgery, "V. Buzzi" Children's Hospital, Milan, Italy.

FAU - Selvaggio, Giorgio Giuseppe Orlando

AU - Selvaggio GGO

AD - Department of Pediatric Surgery, "V. Buzzi" Children's Hospital, Milan, Italy.

FAU - Lanfranchi, Giulia

AU - Lanfranchi G

AD - Department of Pediatric Surgery, "V. Buzzi" Children's Hospital, Milan, Italy.

FAU - Casanova, Michela

AU - Casanova M

AD - Pediatric Oncology Unit, Medical Oncology Department, Fondazione IRCCS Istituto

Nazionale Tumori, Milan, Italy.

FAU - Collini, Paola

AU - Collini P

AD - Department of Pathology and Laboratory Medicine, Fondazione IRCCS Istituto

Nazionale Tumori, Milan, Italy.

FAU - Barisella, Marta

AU - Barisella M

AD - Pathology Unit, ASST Fatebenefratelli Sacco, Milan, Italy.

FAU - Napolitano, Marcello

AU - Napolitano M

AD - Department of Pediatric Radiology and Neuroradiology, "V. Buzzi" Children's

Hospital, Milan, Italy.

FAU - Camporesi, Anna

AU - Camporesi A

AD - Pediatric Anesthesia and Intensive Care Unit, "V. Buzzi" Children's Hospital,

Milan, Italy.

FAU - Pelizzo, Gloria

AU - Pelizzo G

AD - Department of Pediatric Surgery, "V. Buzzi" Children's Hospital, Milan, Italy.

AD - Department of Biomedical and Clinical Sciences, University of Milan, Milan,

Italy.

LA - eng

PT - Case Reports

PT - Journal Article

DEP - 20241028

PL - China

TA - Transl Pediatr

JT - Translational pediatrics

JID - 101649179

PMC - PMC11543115

OTO - NOTNLM

OT - Inflammatory myofibroblastic tumor (IMT)

OT - case series

OT - core biopsy

OT - neoplasms

OT - pediatric

COIS- Conflicts of Interest: All authors have completed the ICMJE uniform disclosure

form (available at

https://tp.amegroups.com/article/view/10.21037/tp-24-239/coif). The authors have

no conflicts of interest to declare.

EDAT- 2024/11/13 13:59

MHDA- 2024/11/13 14:00

PMCR- 2024/10/01

CRDT- 2024/11/11 05:29

PHST- 2024/06/20 00:00 [received]

PHST- 2024/09/04 00:00 [accepted]

PHST- 2024/11/13 14:00 [medline]

PHST- 2024/11/13 13:59 [pubmed]

PHST- 2024/11/11 05:29 [entrez]

PHST- 2024/10/01 00:00 [pmc-release]

AID - tp-13-10-1799 [pii]

AID - 10.21037/tp-24-239 [doi]

PST - ppublish

SO - Transl Pediatr. 2024 Oct 1;13(10):1799-1809. doi: 10.21037/tp-24-239. Epub 2024

Oct 28.

PMID- 39516994

OWN - NLM

STAT- PubMed-not-MEDLINE

LR - 20241116

IS - 2731-6203 (Electronic)

IS - 2731-6203 (Linking)

VI - 3

IP - 1

DP - 2024 May 21

TI - Multiple anaplastic lymphoma kinase-positive primary inflammatory myofibroblastic

tumors with spontaneously expanding and shrinking nodules in both lungs: a case

report.

PG - 29

LID - 10.1186/s44215-024-00153-7 [doi]

LID - 29

AB - BACKGROUND: Inflammatory myofibroblastic tumors (IMTs) are uncommon neoplasms

most prevalent in individuals under 40 years old and predominantly in the lungs.

Despite their rarity, multiple anaplastic lymphoma kinase (ALK)-positive IMTs,

especially those of various sizes, have not been widely reported. This report

describes a case of multiple ALK-positive IMTs in the lungs, aiming to further

our understanding of their behavior and management. CASE PRESENTATION: Herein, we

present the case of a 64-year-old woman who presented with an abnormal shadow on

chest examination. Chest computed tomography revealed a main tumor in the right

middle lobe and multiple irregularly shaped small nodules in both lungs. Thus,

thoracoscopic wedge resection of the left lower lobe was performed for diagnosis.

Pathological findings indicated smooth muscle proliferation without malignancy.

IMT was diagnosed following thoracoscopic right middle lobectomy. Twenty months

postoperatively, one residual nodule shrank, but another grew. CONCLUSIONS: This

is the first report of multiple ALK-positive IMTs in both lungs, highlighting the

need for definitive diagnosis and treatment of IMTs based on surgical resection.

Although caution is required in patients with lymph node metastases or distant

metastases, careful follow-up is acceptable unless there is a tendency for

nodules to increase in size on imaging.

CI - © 2024. The Author(s).

FAU - Nomura, Shunsuke

AU - Nomura S

AD - Department of Thoracic Surgery, Sapporo Minami-Sanjo Hospital, 4-2 S3W6,

Chuou-Ku, Sapporo, Hokkaido, 060-0063, Japan.

FAU - Kaji, Mitsuhito

AU - Kaji M

AD - Department of Thoracic Surgery, Sapporo Minami-Sanjo Hospital, 4-2 S3W6,

Chuou-Ku, Sapporo, Hokkaido, 060-0063, Japan.

FAU - Shiina, Nobuyuki

AU - Shiina N

AD - Department of Thoracic Surgery, Sapporo Minami-Sanjo Hospital, 4-2 S3W6,

Chuou-Ku, Sapporo, Hokkaido, 060-0063, Japan.

FAU - Chiba, Ryohei

AU - Chiba R

AD - Department of Thoracic Surgery, Sapporo Minami-Sanjo Hospital, 4-2 S3W6,

Chuou-Ku, Sapporo, Hokkaido, 060-0063, Japan.

FAU - Cho, Yasushi

AU - Cho Y

AD - Department of Thoracic Surgery, Sapporo-Kosei General Hospital, N3E8, Chuou-Ku,

Sapporo, Hokkaido, 060-0033, Japan.

FAU - Shiiya, Haruhiko

AU - Shiiya H

AD - Department of Thoracic Surgery, Hokkaido University Hospital, N14W5, Kita-Ku,

Sapporo, Hokkaido, 060-8648, Japan.

FAU - Kato, Tatsuya

AU - Kato T

AUID- ORCID: 0000-0003-4063-0042

AD - Department of Thoracic Surgery, Hokkaido University Hospital, N14W5, Kita-Ku,

Sapporo, Hokkaido, 060-8648, Japan. katotatu@huhp.hokudai.ac.jp.

LA - eng

PT - Journal Article

DEP - 20240521

PL - England

TA - Gen Thorac Cardiovasc Surg Cases

JT - General Thoracic and Cardiovascular Surgery Cases

JID - 9918900337406676

PMC - PMC11533626

OTO - NOTNLM

OT - Anaplastic lymphoma kinase

OT - Inflammatory myofibroblastic tumor

OT - Multiple

COIS- The authors declare that they have no competing interests.

EDAT- 2024/11/13 13:57

MHDA- 2024/11/13 13:58

PMCR- 2024/05/21

CRDT- 2024/11/09 00:03

PHST- 2023/12/05 00:00 [received]

PHST- 2024/04/26 00:00 [accepted]

PHST- 2024/11/13 13:58 [medline]

PHST- 2024/11/13 13:57 [pubmed]

PHST- 2024/11/09 00:03 [entrez]

PHST- 2024/05/21 00:00 [pmc-release]

AID - 10.1186/s44215-024-00153-7 [pii]

AID - 153 [pii]

AID - 10.1186/s44215-024-00153-7 [doi]

PST - epublish

SO - Gen Thorac Cardiovasc Surg Cases. 2024 May 21;3(1):29. doi:

10.1186/s44215-024-00153-7.

PMID- 39505375

OWN - NLM

STAT- MEDLINE

DCOM- 20241106

LR - 20241106

IS - 0412-4081 (Print)

IS - 0412-4081 (Linking)

VI - 60

IP - 11

DP - 2024 Nov 11

TI - [Orbital and eyelid inflammatory myofibroblastic tumors: a clinicopathological

analysis of 13 cases].

PG - 900-906

LID - 10.3760/cma.j.cn112142-20240109-00016 [doi]

AB - Objective: To investigate the clinicopathological features of orbital and eyelid

inflammatory myofibroblastic tumors (IMTs). Methods: A retrospective analysis was

conducted among 13 patients with IMTs treated at Tianjin Eye Hospital between

January 2000 and October 2023. The clinicopathological data, immunohistochemical

staining characteristics, molecular phenotypes, and follow-up outcomes were

collected and analyzed. Results: The study cohort comprised 7 males and 6

females. The median age was 44 (22, 68) years. The tumor was observed in the left

eye in 6 cases and the right eye in 7 cases. The age at onset ranged from 5 to 76

years, with a disease course of 1 month to 2 years before tumor excision. The

tumor was located within the orbit in 10 cases (including one case of a systemic

multifocal lesion) and in the eyelid in 3 cases. Pathological characteristics of

the tumors were mainly proliferation of spindle or ovoid fibroblasts and

myofibroblasts, with varying degrees of mucoid degeneration or collagenization of

the mesenchyme, and varying amounts of lymphocytes and plasma cells infiltrated

in the background. Immunohistochemical analysis revealed that Vimentin and α-SMA

were positive in all cases (13/13, 13/13), ALK1 was positive in 5 cases (5/11),

CK was positive in 1 case (1/1), and Desmin was positive in 1 case (1/6). ALK

fluorescence in situ hybridization was positive in 2 out of 3 cases, and EBER in

situ hybridization was negative in 6 cases. Nine patients were available for the

follow-up of 7 to 139 months. Seven patients remained tumor-free, 2 experienced

relapses, and no deaths or metastases were reported. Conclusions: Orbital and

eyelid IMTs predominantly occurred in adults, with localized lesions in most

cases and favorable prognoses. The disease was histopathologically characterized

by proliferation of fibroblasts and myofibroblasts. The accurate diagnosis

depended on a comprehensive assessment of the morphological features of the tumor

histopathology as well as immunohistochemical markers such as ALK and α-SMA, and

molecular detection.

FAU - Li, J

AU - Li J

AD - Tianjin Eye Hospital, Nankai University Affiliated Eye Hospital, Clinical College

of Ophthalmology of Tianjin Medical University, Tianjin Eye Institute, Tianjin

Key Laboratory of Ophthalmology and Visual Science, Tianjin 300020, China.

FAU - Wang, Y C

AU - Wang YC

AD - Tianjin Eye Hospital, Nankai University Affiliated Eye Hospital, Clinical College

of Ophthalmology of Tianjin Medical University, Tianjin Eye Institute, Tianjin

Key Laboratory of Ophthalmology and Visual Science, Tianjin 300020, China.

FAU - Lin, J Y

AU - Lin JY

AD - Tianjin Eye Hospital, Nankai University Affiliated Eye Hospital, Clinical College

of Ophthalmology of Tianjin Medical University, Tianjin Eye Institute, Tianjin

Key Laboratory of Ophthalmology and Visual Science, Tianjin 300020, China.

LA - chi

GR - TJYXZDXK‑016A/Tianjin Key Medical Discipline (Specialty) Construction Project/

PT - English Abstract

PT - Journal Article

PL - China

TA - Zhonghua Yan Ke Za Zhi

JT - [Zhonghua yan ke za zhi] Chinese journal of ophthalmology

JID - 16210540R

RN - 0 (Actins)

SB - IM

MH - Humans

MH - Male

MH - Female

MH - Middle Aged

MH - Retrospective Studies

MH - Adult

MH - Aged

MH - *Neoplasms, Muscle Tissue/pathology

MH - *Orbital Neoplasms/pathology

MH - Myofibroblasts/pathology

MH - Young Adult

MH - Eyelid Neoplasms/pathology

MH - Actins/metabolism

MH - Eyelids/pathology

EDAT- 2024/11/07 04:20

MHDA- 2024/11/07 04:21

CRDT- 2024/11/06 20:31

PHST- 2024/11/07 04:21 [medline]

PHST- 2024/11/07 04:20 [pubmed]

PHST- 2024/11/06 20:31 [entrez]

AID - 10.3760/cma.j.cn112142-20240109-00016 [doi]

PST - ppublish

SO - Zhonghua Yan Ke Za Zhi. 2024 Nov 11;60(11):900-906. doi:

10.3760/cma.j.cn112142-20240109-00016.

PMID- 39499317

OWN - NLM

STAT- Publisher

LR - 20241116

IS - 1432-2307 (Electronic)

IS - 0945-6317 (Linking)

DP - 2024 Nov 5

TI - What is new in fibroblastic/myofibroblastic tumors in children.

LID - 10.1007/s00428-024-03964-9 [doi]

AB - Fibroblastic and myofibroblastic neoplasms represent about 12% of pediatric soft

tissue tumors. Most of these neoplasms in children are either benign or locally

aggressive with rare metastasis, while malignant cases are uncommon. Diagnosing

these tumors is challenging due to overlapping morphologies and the limited

utility of immunohistochemistry. Advances in molecular techniques, especially RNA

sequencing, have improved our understanding of the molecular drivers of these

tumors, leading to better classification. Key molecular alterations, such as RTK

and MAPK activation, are central in the development of tumors like infantile

fibrosarcoma (IFS) and inflammatory myofibroblastic tumors (IMT). The

identification of alternative fusions in IFS and IMT underscores the importance

of an integrated diagnostic approach. Furthermore, new RTK-driven lesions, now

included in the WHO's "NTRK-rearranged mesenchymal neoplasms", have been

identified. This review provides an update on recent findings in RTK-driven

myofibroblastic tumors and highlights novel entities still in need of

classification.

CI - © 2024. The Author(s), under exclusive licence to Springer-Verlag GmbH Germany,

part of Springer Nature.

FAU - Al-Ibraheemi, Alyaa

AU - Al-Ibraheemi A

AUID- ORCID: 0000-0003-1769-7839

AD - Department of Pathology, Boston Children's Hospital, 300 Longwood Avenue, Boston,

MA, 02115, USA. Alyaa.Al-Ibraheemi@childrens.harvard.edu.

FAU - Zhou, Yan

AU - Zhou Y

AD - Department of Laboratory Medicine and Pathology, University of Minnesota,

Minneapolis, MN, 55455, USA.

FAU - Rullo, Emma

AU - Rullo E

AD - IRCCS Ospedale Pediatrico Bambino Gesù, Pathology Unit (Rome), Piazza

Sant'Onofrio 4, 00165, Rome, Italy.

FAU - Alaggio, Rita

AU - Alaggio R

AD - IRCCS Ospedale Pediatrico Bambino Gesù, Pathology Unit (Rome), Piazza

Sant'Onofrio 4, 00165, Rome, Italy. rita.alaggio@opbg.net.

AD - Department of Medical-Surgical Biotechnological Sciences, Sapienza University of

Rome, Polo Pontino, 00185, Rome, Italy. rita.alaggio@opbg.net.

LA - eng

PT - Journal Article

PT - Review

DEP - 20241105

PL - Germany

TA - Virchows Arch

JT - Virchows Archiv : an international journal of pathology

JID - 9423843

SB - IM

EIN - Virchows Arch. 2024 Nov 16. doi: 10.1007/s00428-024-03981-8. PMID: 39547970

OTO - NOTNLM

OT - ALK

OT - Fibroblastic

OT - IMT

OT - Infantile fibrosarcoma

OT - NTRK

OT - Receptor tyrosine kinase

COIS- Declarations Ethics approval This article does not contain any studies with human

participants or animals performed by any of the authors. Consent to participate

N/A. Consent for publication N/A. Conflict of interest The authors declare no

competing interests.

EDAT- 2024/11/05 19:20

MHDA- 2024/11/05 19:20

CRDT- 2024/11/05 11:15

PHST- 2024/08/27 00:00 [received]

PHST- 2024/10/26 00:00 [accepted]

PHST- 2024/10/23 00:00 [revised]

PHST- 2024/11/05 19:20 [pubmed]

PHST- 2024/11/05 19:20 [medline]

PHST- 2024/11/05 11:15 [entrez]

AID - 10.1007/s00428-024-03964-9 [pii]

AID - 10.1007/s00428-024-03964-9 [doi]

PST - aheadofprint

SO - Virchows Arch. 2024 Nov 5. doi: 10.1007/s00428-024-03964-9.

PMID- 39498315

OWN - NLM

STAT- PubMed-not-MEDLINE

LR - 20241106

IS - 2194-7619 (Print)

IS - 2194-7627 (Electronic)

IS - 2194-7619 (Linking)

VI - 12

IP - 1

DP - 2024 Jan

TI - Pulmonary Inflammatory Myofibroblastic Tumor: A Case Report.

PG - e73-e76

LID - 10.1055/a-2430-0053 [doi]

AB - An inflammatory myofibroblastic tumor (IMT) is a rare mesenchymal tumor that

occurs predominantly in children and young adults. Etiology remains unclear. But

based on the frequent detection of chromosomic alterations, especially near the

anaplastic lymphoma kinase (ALK) gene, IMT is now considered to be a true

neoplasm. In addition, the possible aggressive behavior, and the ability to

metastasize suggest at least an intermediate malignant potential. Surgery remains

the treatment of choice, but the use of chemotherapy, nonsteroidal

anti-inflammatory drugs, immunotherapy, and targeted therapy are reported. We

describe a case of a pulmonary IMT in a 6-year-old boy with an incidental finding

of a lesion in the right upper lobe. A video-assisted thoracoscopic right upper

lobectomy with lymph node resection was performed. Microscopic examination

confirmed the diagnosis of IMT with the nodule showing spindle cells in a

background of plasma cells. ALK immunohistochemical expression was negative.

CI - The Author(s). This is an open access article published by Thieme under the terms

of the Creative Commons Attribution License, permitting unrestricted use,

distribution, and reproduction so long as the original work is properly cited. (

https://creativecommons.org/licenses/by/4.0/ ).

FAU - Bruyninckx, Lotte

AU - Bruyninckx L

AD - Department of Thoracic Surgery, University Hospitals Leuven, Leuven, Belgium.

FAU - De Leyn, Paul

AU - De Leyn P

AD - Department of Thoracic Surgery, University Hospitals Leuven, Leuven, Belgium.

FAU - Van Raemdonck, Dirk

AU - Van Raemdonck D

AD - Department of Thoracic Surgery, University Hospitals Leuven, Leuven, Belgium.

FAU - Jansen, Yanina

AU - Jansen Y

AD - Department of Thoracic Surgery, University Hospitals Leuven, Leuven, Belgium.

FAU - Coppens, Katrien

AU - Coppens K

AD - Department of Paediatrics, Imelda Hospital, Bonheiden, Belgium.

FAU - Vermeulen, Francois

AU - Vermeulen F

AD - Department of Paediatrics, University Hospitals Leuven, Leuven, Vlaams-Brabant,

Belgium.

FAU - Weynand, Birgit

AU - Weynand B

AD - Department of Pathology, University Hospitals Leuven, Leuven, Vlaams-Brabant,

Belgium.

FAU - Gieraerts, Christopher

AU - Gieraerts C

AD - Department of Radiology, Imelda Hospital, Bonheiden, Belgium.

FAU - Decaluwé, Herbert

AU - Decaluwé H

AD - Department of Thoracic Surgery, University Hospitals Leuven, Leuven, Belgium.

AD - Department of Thoracovascular Surgery, Ziekenhuis Oost-Limburg, Genk, Limburg,

Belgium.

LA - eng

PT - Case Reports

PT - Journal Article

DEP - 20241104

PL - Germany

TA - European J Pediatr Surg Rep

JT - European journal of pediatric surgery reports

JID - 101620104

PMC - PMC11534498

OTO - NOTNLM

OT - anaplastic lymphoma kinase

OT - histopathology

OT - inflammatory myofibroblastic tumor

OT - pediatric surgery

COIS- Conflict of Interest None declared.

EDAT- 2024/11/05 10:54

MHDA- 2024/11/05 10:55

PMCR- 2024/11/01

CRDT- 2024/11/05 04:37

PHST- 2023/12/03 00:00 [received]

PHST- 2024/09/17 00:00 [accepted]

PHST- 2024/11/05 10:55 [medline]

PHST- 2024/11/05 10:54 [pubmed]

PHST- 2024/11/05 04:37 [entrez]

PHST- 2024/11/01 00:00 [pmc-release]

AID - EJPSR-2023-12-0740-CR [pii]

AID - 10.1055/a-2430-0053 [doi]

PST - epublish

SO - European J Pediatr Surg Rep. 2024 Nov 4;12(1):e73-e76. doi: 10.1055/a-2430-0053.

eCollection 2024 Jan.

PMID- 39463656

OWN - NLM

STAT- PubMed-not-MEDLINE

LR - 20241029

IS - 2168-8184 (Print)

IS - 2168-8184 (Electronic)

IS - 2168-8184 (Linking)

VI - 16

IP - 9

DP - 2024 Sep

TI - Inflammatory Myofibroblastic Tumor of the Lung: A Case Report.

PG - e70207

LID - 10.7759/cureus.70207 [doi]

LID - e70207

AB - Inflammatory myofibroblastic tumor (IMT) is a rare mesenchymal tumor

classified as an intermediate malignancy that rarely metastasizes. The most

common site for IMTs is the lung, though they can develop in various other

anatomical locations. Pulmonary IMTs are more common in children and

adolescents and are infrequently diagnosed in adults. This case report describes

a 49-year-old woman with a history of breast cancer previously treated with

subtotal mastectomy and chemotherapy who developed an IMT in the lung. This case

emphasizes the rarity of the disease and the associated clinical challenges when

managing such cases.

CI - Copyright © 2024, Baltagianni et al.

FAU - Baltagianni, Marianthi

AU - Baltagianni M

AD - Department of Thoracic Surgery, Metaxa Cancer Hospital, Piraeus, GRC.

FAU - Leivaditis, Vasileios

AU - Leivaditis V

AD - Department of Cardiothoracic and Vascular Surgery, Westpfalz-Klinikum,

Kaiserslautern, DEU.

FAU - Baltayiannis, Nikolaos

AU - Baltayiannis N

AD - Department of Thoracic Surgery, Metaxa Cancer Hospital, Piraeus, GRC.

FAU - Stanc, Gabriela

AU - Stanc G

AD - Department of Pathology, Metaxa Cancer Hospital, Piraeus, GRC.

FAU - Souka, Efstathia

AU - Souka E

AD - Department of Pathology, Metaxa Cancer Hospital, Piraeus, GRC.

FAU - Batika, Pella

AU - Batika P

AD - Department of Thoracic Surgery, Metaxa Cancer Hospital, Piraeus, GRC.

FAU - Beltsios, Eleftherios

AU - Beltsios E

AD - Department of Anesthesiology and Critical Care, Hannover Medical School,

Hannover, DEU.

AD - Department of Cardiothoracic Surgery, General University Hospital of Patras,

Patras, GRC.

FAU - Mulita, Francesk

AU - Mulita F

AD - Department of Surgery, General University Hospital of Patras, Patras, GRC.

FAU - Papatriantafyllou, Athanasios

AU - Papatriantafyllou A

AD - Department of Cardiothoracic and Vascular Surgery, Westpfalz-Klinikum,

Kaiserslautern, DEU.

FAU - Koletsis, Efstratios N

AU - Koletsis EN

AD - Department of Cardiothoracic Surgery, General University Hospital of Patras,

Patras, GRC.

LA - eng

PT - Case Reports

PT - Journal Article

DEP - 20240925

PL - United States

TA - Cureus

JT - Cureus

JID - 101596737

PMC - PMC11510679

OTO - NOTNLM

OT - inflammation

OT - inflammatory myofibroblastic tumor

OT - lung tumor

OT - mesenchymal tumor

OT - thoracic

COIS- Human subjects: Consent was obtained or waived by all participants in this study.

Conflicts of interest: In compliance with the ICMJE uniform disclosure form, all

authors declare the following: Payment/services info: All authors have declared

that no financial support was received from any organization for the submitted

work. Financial relationships: All authors have declared that they have no

financial relationships at present or within the previous three years with any

organizations that might have an interest in the submitted work. Other

relationships: All authors have declared that there are no other relationships or

activities that could appear to have influenced the submitted work.

EDAT- 2024/10/28 06:23

MHDA- 2024/10/28 06:24

PMCR- 2024/09/25

CRDT- 2024/10/28 05:03

PHST- 2024/09/24 00:00 [accepted]

PHST- 2024/10/28 06:24 [medline]

PHST- 2024/10/28 06:23 [pubmed]

PHST- 2024/10/28 05:03 [entrez]

PHST- 2024/09/25 00:00 [pmc-release]

AID - 10.7759/cureus.70207 [doi]

PST - epublish

SO - Cureus. 2024 Sep 25;16(9):e70207. doi: 10.7759/cureus.70207. eCollection 2024

Sep.

PMID- 39451577

OWN - NLM

STAT- PubMed-not-MEDLINE

LR - 20241027

IS - 2075-4418 (Print)

IS - 2075-4418 (Electronic)

IS - 2075-4418 (Linking)

VI - 14

IP - 20

DP - 2024 Oct 10

TI - A Rare Endobronchial Tumor in a Pediatric Patient.

LID - 10.3390/diagnostics14202254 [doi]

LID - 2254

AB - A pediatric patient who presented with non-specific respiratory symptoms,

including mild hemoptysis, wheezing, and eventual respiratory distress, was found

to have a rare endobronchial inflammatory myofibroblastic tumor obstructing her

right mainstem bronchus. It was diagnosed and initially debulked using

bronchoscopy, which helped to stabilize the patient and eliminate the need for

supplemental oxygen. The patient subsequently underwent successful removal of the

residual tumor with parenchymal-sparing sleeve resection. This case highlights

the importance of pursuing appropriate imaging along with diagnostic and

therapeutic bronchoscopy for an endobronchial lesion to help manage pediatric

patients with persistent respiratory symptoms.

FAU - Parekh, Pooja

AU - Parekh P

AUID- ORCID: 0000-0002-0250-0434

AD - Department of Medicine, University of Chicago, Chicago, IL 60637, USA.

FAU - Wagh, Ajay

AU - Wagh A

AUID- ORCID: 0000-0003-3402-0272

AD - Division of Pulmonary and Critical Care, Department of Medicine, University of

Chicago, Chicago, IL 60637, USA.

LA - eng

PT - Journal Article

DEP - 20241010

PL - Switzerland

TA - Diagnostics (Basel)

JT - Diagnostics (Basel, Switzerland)

JID - 101658402

PMC - PMC11506295

OTO - NOTNLM

OT - bronchoscopy

OT - central airway obstruction

OT - pediatric endobronchial tumor

COIS- P.P. has no conflicts to declare. A.W. is a consultant for Noah Medical,

Medtronic, Ambu, Biodesix, and Cook Medical.

EDAT- 2024/10/25 12:25

MHDA- 2024/10/25 12:26

PMCR- 2024/10/10

CRDT- 2024/10/25 09:55

PHST- 2024/09/14 00:00 [received]

PHST- 2024/09/29 00:00 [revised]

PHST- 2024/10/07 00:00 [accepted]

PHST- 2024/10/25 12:26 [medline]

PHST- 2024/10/25 12:25 [pubmed]

PHST- 2024/10/25 09:55 [entrez]

PHST- 2024/10/10 00:00 [pmc-release]

AID - diagnostics14202254 [pii]

AID - diagnostics-14-02254 [pii]

AID - 10.3390/diagnostics14202254 [doi]

PST - epublish

SO - Diagnostics (Basel). 2024 Oct 10;14(20):2254. doi: 10.3390/diagnostics14202254.

PMID- 39445266

OWN - NLM

STAT- PubMed-not-MEDLINE

LR - 20241025

IS - 2168-8184 (Print)

IS - 2168-8184 (Electronic)

IS - 2168-8184 (Linking)

VI - 16

IP - 9

DP - 2024 Sep

TI - An Examination of the Diagnostic Utility of Ubiquitin-Specific Peptidase 6 (USP6)

Rearrangement in Differentiating Nodular Fasciitis From Inflammatory

Myofibroblastic Tumor: A Case Report.

PG - e69995

LID - 10.7759/cureus.69995 [doi]

LID - e69995

AB - Nodular fasciitis (NF) is a benign yet diagnostically challenging mesenchymal

myofibroblastic proliferation that often mimics the histological features of

inflammatory myofibroblastic tumors (IMTs) and soft tissue sarcomas. The overlap

in histopathological appearance, compounded by the variability in

immunohistochemical (IHC) staining, frequently leads to diagnostic uncertainty.

In this report, we present a case of a rapidly expanding lesion on the left

medial mandible, ultimately diagnosed as NF. Molecular analysis through

fluorescence in situ hybridization (FISH) identified a ubiquitin-specific

peptidase 6 (USP6; 17p13.2) gene rearrangement, a distinctive marker of NF, which

played a critical role in confirming the diagnosis. IHC analysis, including

negative staining for cytokeratin and ALK, further helped differentiate this

benign entity from other IMTs and malignancies, highlighting the importance of

combining molecular diagnostics with traditional histopathological techniques to

ensure accurate classification and avoid misdiagnosis.

CI - Copyright © 2024, Coleman et al.

FAU - Coleman, Bret-Ashleigh

AU - Coleman BA

AD - Dermatology, Edward Via College of Osteopathic Medicine, Auburn, USA.

FAU - Frasier, Kelly M

AU - Frasier KM

AD - Dermatology, Northwell Health, New Hyde Park, USA.

FAU - Farmer, William

AU - Farmer W

AD - Dermatology, Surgical Dermatology Group, Vestavia Hills, USA.

FAU - Harmon, Christopher B

AU - Harmon CB

AD - Dermatology, Surgical Dermatology Group, Vestavia Hills, USA.

FAU - Parrish, Charles A

AU - Parrish CA

AD - Dermatopathology, DermLab, Birmingham, USA.

LA - eng

PT - Case Reports

PT - Journal Article

DEP - 20240923

PL - United States

TA - Cureus

JT - Cureus

JID - 101596737

PMC - PMC11497858

OTO - NOTNLM

OT - benign neoplasm

OT - inflammatory myofibroblastic tumors

OT - nodular fasciitis

OT - soft-tissue sarcomas

OT - usp6 rearrangement

COIS- Human subjects: Consent was obtained or waived by all participants in this study.

Conflicts of interest: In compliance with the ICMJE uniform disclosure form, all

authors declare the following: Payment/services info: All authors have declared

that no financial support was received from any organization for the submitted

work. Financial relationships: All authors have declared that they have no

financial relationships at present or within the previous three years with any

organizations that might have an interest in the submitted work. Other

relationships: All authors have declared that there are no other relationships or

activities that could appear to have influenced the submitted work.

EDAT- 2024/10/24 16:50

MHDA- 2024/10/24 16:51

PMCR- 2024/09/23

CRDT- 2024/10/24 04:30

PHST- 2024/09/23 00:00 [accepted]

PHST- 2024/10/24 16:51 [medline]

PHST- 2024/10/24 16:50 [pubmed]

PHST- 2024/10/24 04:30 [entrez]

PHST- 2024/09/23 00:00 [pmc-release]

AID - 10.7759/cureus.69995 [doi]

PST - epublish

SO - Cureus. 2024 Sep 23;16(9):e69995. doi: 10.7759/cureus.69995. eCollection 2024

Sep.

PMID- 39437499

OWN - NLM

STAT- PubMed-not-MEDLINE

LR - 20241106

IS - 2210-2612 (Print)

IS - 2210-2612 (Electronic)

IS - 2210-2612 (Linking)

VI - 124

DP - 2024 Nov

TI - Inflammatory myofibroblastic tumor: A rare cause of intestinal obstruction: Case

report.

PG - 110438

LID - S2210-2612(24)01219-7 [pii]

LID - 10.1016/j.ijscr.2024.110438 [doi]

LID - 110438

AB - INTRODUCTION AND IMPORTANCE: Inflammatory myofibroblastic tumor IMT(1) is a rare

neoplasm with diverse clinical presentations, needing histological confirmation

for diagnosis. The tumor often mimics malignant conditions, making accurate

diagnosis challenging. CASE PRESENTATION: We report a case of a 29-year-old male

presenting with long standing recurrent abdominal pain, with more recent

increasing frequency and associated weight loss. Imaging revealed a heterogenous

soft tissue lesions causing mechanical small bowel obstruction, prompting

surgical intervention. Histology analysis confirmed the diagnosis of IMT.

CLINICAL DISCUSSION: This case highlights the importance of considering IMT in

the differential diagnosis of neoplastic mechanical small bowel obstruction,

particularly in patients presenting with atypical symptoms and image findings.

Despite its rarity, IMT should be considered, as it can present similarly to more

common malignancies. Further research is warranted to unravel the pathogenesis

and refine management strategies for this intriguing neoplasm. CONCLUSION: IMT is

a rare and intriguing neoplasm that presents significant diagnostic challenge.

This case emphasize the need for thorough histological evaluation to confirm rhe

diagnosis and guide appropriate management strategies.

CI - Copyright © 2024 The Authors. Published by Elsevier Ltd.. All rights reserved.

FAU - Maqbool, Nargis

AU - Maqbool N

AD - Department of Surgery, Aga Khan University Hospital, Karachi, Pakistan.

Electronic address: nargis.maqbool@aku.edu.

FAU - Pal, Khawaja Mohammad Babar

AU - Pal KMB

AD - Dow International Medical College, DUHS, Pakistan.

FAU - Uddin, Zeeshan

AU - Uddin Z

AD - Department of Pathology and Laboratory Medicine, Aga Khan University Hospital,

Karachi, Pakistan.

FAU - Safdar, Fatima

AU - Safdar F

AD - Department of Pathology and Laboratory Medicine, Aga Khan University Hospital,

Karachi, Pakistan.

LA - eng

PT - Case Reports

PT - Journal Article

DEP - 20241013

PL - Netherlands

TA - Int J Surg Case Rep

JT - International journal of surgery case reports

JID - 101529872

PMC - PMC11532891

OTO - NOTNLM

OT - Inflammatory myofibroblastic tumor

OT - Inflammatory pseudotumor

OT - Intestinal obstruction

OT - Plasma cell granuloma

EDAT- 2024/10/23 04:22

MHDA- 2024/10/23 04:23

PMCR- 2024/10/13

CRDT- 2024/10/22 18:02

PHST- 2024/08/19 00:00 [received]

PHST- 2024/10/07 00:00 [revised]

PHST- 2024/10/09 00:00 [accepted]

PHST- 2024/10/23 04:23 [medline]

PHST- 2024/10/23 04:22 [pubmed]

PHST- 2024/10/22 18:02 [entrez]

PHST- 2024/10/13 00:00 [pmc-release]

AID - S2210-2612(24)01219-7 [pii]

AID - 110438 [pii]

AID - 10.1016/j.ijscr.2024.110438 [doi]

PST - ppublish

SO - Int J Surg Case Rep. 2024 Nov;124:110438. doi: 10.1016/j.ijscr.2024.110438. Epub

2024 Oct 13.

PMID- 39424447

OWN - NLM

STAT- MEDLINE

DCOM- 20250112

LR - 20250112

IS - 0242-6498 (Print)

IS - 0242-6498 (Linking)

VI - 45

IP - 1

DP - 2025 Jan

TI - [Translocation-associated uterine mesenchymal tumors: The new without forgetting

the old. An integrated diagnostic approach].

PG - 53-77

LID - S0242-6498(24)00200-1 [pii]

LID - 10.1016/j.annpat.2024.09.011 [doi]

AB - This review focuses on uterine mesenchymal tumors that are defined on a molecular

level by a single and unique genetic alteration, that is somehow necessary and

sufficient to allow tumor growth and progression. Although diverse from a

clinical, morphological and immunohistochemical point of view, the different

entities we are going to talk about share both a simple genomic profile with a

low number of chromosomal alterations observed by CGH Array (few deletions, gains

or amplifications...) and a low mutational burden observed by sequencing

technics. Some of these entities are already well known and described in the

literature when found outside of the uterus and gynecological tract. It remains

intriguing that uterine mesenchymal pathology has been lagging behind when

compared to its extrauterine counterpart. How can we explain that when it comes

to inflammatory myofibroblastic tumors, abundant numbers of articles have been

published since the 70's, but it was only in the early 2000s that the first

relevant descriptions of this tumor in the uterus emerged? Certainly, the

increased accuracy, availability, and use of molecular biology technics and in

particular RNA sequencing in the area of uterine pathology can partly explain the

reduction of the gap between soft tissue and uterine pathology we currently

observe. Other reasons explaining this gap may be the high prevalence of smooth

muscle tumors in the uterus and the abounding diversity of their morphological

aspects, which may have partly eclipsed the array of differential diagnoses. Last

but not least, one can hypothesize that the relative "simplicity" of hysterectomy

procedures, referring to their safety and accessibility, has cured most of the

lesions and partly clouded our knowledge regarding the biological potential and

natural history of these newly described entities. As a consequence of this

situation, our reader will often encounter the wording "uncertain malignant

potential", as for some of these rare entities, evidence to establish reliable

prognostic variables is still insufficient. We hope this review to be a useful

tool to guide pathologists through the diversity and complexity of uterine

mesenchymal tumors. As a scientific and medical community, sharing this knowledge

will help us to collectively raise our vigilance and awareness by expanding the

array of our differential diagnoses. We hope this will lead to more cases being

accurately diagnosed, and ultimately, to a deeper knowledge regarding the

biological potential and clinical evolution of these tumors. From a therapeutical

point of view, the consequences of an accurate diagnosis for the patient are

already appreciable through the use of targeted therapy. Examples include: ALK

inhibitors in inflammatory myofibroblastic tumor, tyrosine-kinase inhibitors in

COL1A::PDGFB rearranged sarcomas or mTOR inhibitors in PEComa.

CI - Copyright © 2024 Elsevier Masson SAS. All rights reserved.

FAU - Fontanges, Quitterie

AU - Fontanges Q

AD - Département de pathologie, cliniques universitaires de Saint-Luc, Bruxelles,

Belgique. Electronic address: qfontanges@hotmail.com.

FAU - Truffaux, Nathalène

AU - Truffaux N

AD - Département de biopathologie, institut Bergonié, Bordeaux, France.

FAU - Azmani, Rihab

AU - Azmani R

AD - Unité bio-informatique, direction données et santé numérique, institut Bergonié,

Bordeaux, France.

FAU - Bourdon, Aurélien

AU - Bourdon A

AD - Unité bio-informatique, direction données et santé numérique, institut Bergonié,

Bordeaux, France.

FAU - Croce, Sabrina

AU - Croce S

AD - Département de biopathologie, institut Bergonié, Bordeaux, France; Unité Inserm

1312, Bordeaux, France.

LA - fre

PT - English Abstract

PT - Journal Article

PT - Review

TT - Tumeurs mésenchymateuses utérines associées à des translocations : du nouveau

sans oublier l’ancien. Une approche diagnostique intégrée.

DEP - 20241018

PL - France

TA - Ann Pathol

JT - Annales de pathologie

JID - 8106337

RN - 0 (Biomarkers, Tumor)

SB - IM

MH - Humans

MH - Female

MH - *Uterine Neoplasms/pathology/genetics/diagnosis

MH - Diagnosis, Differential

MH - Biomarkers, Tumor/analysis

MH - Mesenchymoma/pathology/diagnosis/genetics

OTO - NOTNLM

OT - Génomique simple

OT - Sarcome à translocation

OT - Simple genomic profile

OT - Translocation

OT - Tumeurs mésenchymateuses utérines

OT - Uterine mesenchymal neoplasms

EDAT- 2024/10/19 16:23

MHDA- 2025/01/13 00:20

CRDT- 2024/10/18 21:53

PHST- 2024/04/10 00:00 [received]

PHST- 2024/09/15 00:00 [revised]

PHST- 2024/09/16 00:00 [accepted]

PHST- 2025/01/13 00:20 [medline]

PHST- 2024/10/19 16:23 [pubmed]

PHST- 2024/10/18 21:53 [entrez]

AID - S0242-6498(24)00200-1 [pii]

AID - 10.1016/j.annpat.2024.09.011 [doi]

PST - ppublish

SO - Ann Pathol. 2025 Jan;45(1):53-77. doi: 10.1016/j.annpat.2024.09.011. Epub 2024

Oct 18.

PMID- 39391027

OWN - NLM

STAT- PubMed-not-MEDLINE

LR - 20241012

IS - 1930-0433 (Print)

IS - 1930-0433 (Electronic)

IS - 1930-0433 (Linking)

VI - 19

IP - 12

DP - 2024 Dec

TI - A rare case of inflammatory myofibroblastic tumour of the thigh : A case report

and literature review.

PG - 6528-6533

LID - 10.1016/j.radcr.2024.09.066 [doi]

AB - Towards the beginning of the twentieth century, inflammatory myofibroblastic

tumors were described as neoplasms characterized by myofibroblastic spindle cells

with an infiltrate of inflammatory cells. These rare tumors occur mainly in

children and young adults with a preferential pulmonary and abdominal location.

They have intermediate biological potency with a tendency to local recurrence and

rarely metastasize. We describe the case of an adolescent who presented with an

inflammatory myofibroblastic tumor of the thigh, a very rare entity that has only

been reported in the literature as case reports, and its clinical, radiological,

echographic and MRI appearance. The patient underwent complete surgical resection

of the mass with healthy margins and did not receive any additional treatment. No

sign of recurrence was detected after 6 months of follow-up.

CI - © 2024 The Authors. Published by Elsevier Inc. on behalf of University of

Washington.

FAU - Batou, Yassine

AU - Batou Y

AD - Faculty of Medicine and Pharmacy, Mohammed I University, Oujda, Morocco.

AD - Department of Traumatology, orthopedic Mohammed VI University Hospital Mohammed I

University, Oujda, Morocco.

FAU - Aissaoui, Taha El

AU - Aissaoui TE

AD - Faculty of Medicine and Pharmacy, Mohammed I University, Oujda, Morocco.

AD - Department of Traumatology, orthopedic Mohammed VI University Hospital Mohammed I

University, Oujda, Morocco.

FAU - Farhaoui, Amine El

AU - Farhaoui AE

AD - Faculty of Medicine and Pharmacy, Mohammed I University, Oujda, Morocco.

AD - Department of Traumatology, orthopedic Mohammed VI University Hospital Mohammed I

University, Oujda, Morocco.

FAU - Zeryouh, Brahim

AU - Zeryouh B

AD - Faculty of Medicine and Pharmacy, Mohammed I University, Oujda, Morocco.

AD - Department of Traumatology, orthopedic Mohammed VI University Hospital Mohammed I

University, Oujda, Morocco.

FAU - Lachkar, Adnane

AU - Lachkar A

AD - Faculty of Medicine and Pharmacy, Mohammed I University, Oujda, Morocco.

AD - Department of Traumatology, orthopedic Mohammed VI University Hospital Mohammed I

University, Oujda, Morocco.

FAU - Zouaidia, Fouad

AU - Zouaidia F

AD - Faculty of Medicine and Pharmacy, Mohammed V University, Rabat, Morocco.

AD - Department of Pathological Anatomy, Ibn Sina University Hospital, Mohammed V

University Rabat, Morocco.

FAU - Abdeljaouad, Najib

AU - Abdeljaouad N

AD - Faculty of Medicine and Pharmacy, Mohammed I University, Oujda, Morocco.

AD - Department of Traumatology, orthopedic Mohammed VI University Hospital Mohammed I

University, Oujda, Morocco.

FAU - Yacoubi, Hicham

AU - Yacoubi H

AD - Faculty of Medicine and Pharmacy, Mohammed I University, Oujda, Morocco.

AD - Department of Traumatology, orthopedic Mohammed VI University Hospital Mohammed I

University, Oujda, Morocco.

LA - eng

PT - Case Reports

PT - Journal Article

DEP - 20240927

PL - Netherlands

TA - Radiol Case Rep

JT - Radiology case reports

JID - 101467888

PMC - PMC11465054

OTO - NOTNLM

OT - Biopsy

OT - Enhancement

OT - IMT

OT - MRI

OT - Margins

OT - Recurrence

OT - Resection

OT - Spindle cells

OT - Thigh

EDAT- 2024/10/11 10:51

MHDA- 2024/10/11 10:52

PMCR- 2024/09/27

CRDT- 2024/10/11 04:14

PHST- 2024/08/06 00:00 [received]

PHST- 2024/09/11 00:00 [revised]

PHST- 2024/09/13 00:00 [accepted]

PHST- 2024/10/11 10:52 [medline]

PHST- 2024/10/11 10:51 [pubmed]

PHST- 2024/10/11 04:14 [entrez]

PHST- 2024/09/27 00:00 [pmc-release]

AID - S1930-0433(24)01014-8 [pii]

AID - 10.1016/j.radcr.2024.09.066 [doi]

PST - epublish

SO - Radiol Case Rep. 2024 Sep 27;19(12):6528-6533. doi: 10.1016/j.radcr.2024.09.066.

eCollection 2024 Dec.

PMID- 39382359

OWN - NLM

STAT- MEDLINE

DCOM- 20241009

LR - 20241208

IS - 1307-7945 (Electronic)

IS - 1306-696X (Print)

IS - 1306-696X (Linking)

VI - 30

IP - 10

DP - 2024 Oct

TI - Acute abdomen due to Meckel's diverticulitis with synchronous inflammatory

myofibroblastic tumor in the terminal ileum: A case report.

PG - 764-767

LID - 10.14744/tjtes.2024.82091 [doi]

AB - Meckel's diverticulum (MD) is the most common congenital anomaly of the

gastrointestinal system, occurring in approximately 2% of the population. It is

rare for MD to be symptomatic or complicated in adulthood. In this case report,

we describe a patient who was admitted to the clinic with Meckel's

diverticulitis, which had fistulized to the anterior abdominal wall, and was

incidentally found to have an ileal inflammatory myofibroblastic tumor (IMT) on

radiological imaging. A 46-year-old male patient presented to the emergency

department with abdominal pain. Physical examination revealed localized guarding

on the right side of the umbilicus. Blood tests showed elevated acute-phase

reactants, including a white blood cell count of 13,800/µL, and C-reactive

protein (CRP) level of 165 mg/L. Abdominal computed tomography demonstrated

Meckel's diverticulitis fistulizing to the anterior abdominal wall and a polypoid

structure in the ileum distal to the MD. The patient underwent emergency surgery,

during which segmental ileal resection and ileocolic anastomosis were performed.

On the fourth postoperative day, the patient developed an anastomotic leak.

Relaparotomy, right hemicolectomy with end ileostomy, and mucous fistula creation

were subsequently performed. Pathological examination of the resected ileum from

the initial surgery revealed a benign IMT distal to the MD. The patient was

discharged on the 40th postoperative day after developing a surgical site

infection following the second surgery. The end ileostomy was closed six months

later. In this case, it appears that the ileal IMT located distal to the MD may

have caused intermittent intestinal obstruction, fecal stasis, and the

development of Meckel's diverticulitis. Furthermore, a detailed examination of

the patient's history, laboratory results, and radiologic tests may contribute to

the detection of incidental pathologies and influence treatment choices.

FAU - Dinçer, Burak

AU - Dinçer B

AD - Department of General Surgery, Şişli Hamidiye Etfal Training and Research

Hospital, Istanbul-Türkiye.

FAU - Ömeroğlu, Sinan

AU - Ömeroğlu S

AD - Department of General Surgery, Şişli Hamidiye Etfal Training and Research

Hospital, Istanbul-Türkiye.

FAU - Güven, Onur

AU - Güven O

AD - Department of General Surgery, Şişli Hamidiye Etfal Training and Research

Hospital, Istanbul-Türkiye.

FAU - Celayir, Mustafa Fevzi

AU - Celayir MF

AD - Department of General Surgery, Şişli Hamidiye Etfal Training and Research

Hospital, Istanbul-Türkiye.

FAU - Demir, Uygar

AU - Demir U

AD - Department of General Surgery, Şişli Hamidiye Etfal Training and Research

Hospital, Istanbul-Türkiye.

LA - eng

PT - Case Reports

PT - Journal Article

TT - Meckel divertikülitine bağlı akut batın olgusunda terminal ileumda senkron

inflamatuar myofibroblastik tümör: Olgu sunumu.

PL - Turkey

TA - Ulus Travma Acil Cerrahi Derg

JT - Ulusal travma ve acil cerrahi dergisi = Turkish journal of trauma & emergency

surgery : TJTES

JID - 101274231

SB - IM

MH - Humans

MH - *Meckel Diverticulum/complications/surgery/diagnosis/pathology/diagnostic imaging

MH - Male

MH - Middle Aged

MH - *Abdomen, Acute/etiology/surgery

MH - Ileal Neoplasms/surgery/complications/diagnosis/pathology/diagnostic imaging

MH - Diverticulitis/complications/surgery/diagnosis/diagnostic imaging

MH - Tomography, X-Ray Computed

PMC - PMC11622708

COIS- Conflict of Interest: None declared.

EDAT- 2024/10/09 12:24

MHDA- 2024/10/09 12:25

PMCR- 2024/10/07

CRDT- 2024/10/09 09:23

PHST- 2024/10/09 12:25 [medline]

PHST- 2024/10/09 12:24 [pubmed]

PHST- 2024/10/09 09:23 [entrez]

PHST- 2024/10/07 00:00 [pmc-release]

AID - TJTES-30-764 [pii]

AID - 10.14744/tjtes.2024.82091 [doi]

PST - ppublish

SO - Ulus Travma Acil Cerrahi Derg. 2024 Oct;30(10):764-767. doi:

10.14744/tjtes.2024.82091.

PMID- 39377108

OWN - NLM

STAT- Publisher

LR - 20241008

IS - 1940-2465 (Electronic)

IS - 1066-8969 (Linking)

DP - 2024 Oct 8

TI - Benign Tumors and Tumor-Like Conditions of Ampulla and Small Intestine: The

PathologyOutlines.com Review.

PG - 10668969241283748

LID - 10.1177/10668969241283748 [doi]

AB - Small intestine and ampulla can be involved in a heterogeneous group of benign

tumors and tumor-like diseases, potentially mimicking malignant neoplasms.

In-depth knowledge of them is critical for practicing pathologists since they

represent potential pitfalls in routine diagnostic activity. Such conditions

include the following: (1) Brunner gland lesions, including Brunner gland

hyperplasia and hamartoma; (2) polyps, such as adenoma, hyperplastic,

hamartomatous, and inflammatory; (3) mesenchymal proliferations encompassing

inflammatory fibroid polyp, inflammatory myofibroblastic tumor, leiomyomas,

lipomas, and lipomatosis of the ileocecal valve; (4) fibrosis-associated

diseases, such as idiopathic retroperitoneal fibrosis, reactive nodular fibrous

pseudotumor, and sclerosing peritonitis; (5) disorders of lymphatic vessels,

including lymphangiectasia and lymphangiomas; and (6) other rare

conditions/miscellanea, such as enteritis cystica profunda, intussusception,

ischemia, and pneumatosis cystoides intestinalis. This review, inspired by the

content of the PathologyOutlines website (https://www.pathologyoutlines.com),

aims to provide a reference point in this complex scenario, summarizing the

essential histopathological features of all these entities for better addressing

routine practice and differential diagnoses.

FAU - Luchini, Claudio

AU - Luchini C

AUID- ORCID: 0000-0003-4901-4908

AD - Department of Diagnostics and Public Health, Section of Pathology, and ARC-NET

Research Center, University of Verona, Verona, Italy. RINGGOLD: 19051

CN - PathologyOutlines.com Review Committee

AD - See acknowledgments for the complete list of members.

CN - PathologyOutlines.com Contributors

AD - See acknowledgments for the complete list of members.

FAU - Pernick, Nat

AU - Pernick N

AD - PathologyOutlines.com Owner and Founder.

LA - eng

PT - Journal Article

PT - Review

DEP - 20241008

PL - United States

TA - Int J Surg Pathol

JT - International journal of surgical pathology

JID - 9314927

SB - IM

OTO - NOTNLM

OT - Vater

OT - ampulla

OT - duodenum

OT - ileum

OT - small intestine

COIS- Declaration of Conflicting InterestsThe authors declared no potential conflicts

of interest with respect to the research, authorship, and/or publication of this

article.

EDAT- 2024/10/08 07:03

MHDA- 2024/10/08 07:03

CRDT- 2024/10/08 04:49

PHST- 2024/10/08 07:03 [medline]

PHST- 2024/10/08 07:03 [pubmed]

PHST- 2024/10/08 04:49 [entrez]

AID - 10.1177/10668969241283748 [doi]

PST - aheadofprint

SO - Int J Surg Pathol. 2024 Oct 8:10668969241283748. doi: 10.1177/10668969241283748.

PMID- 39376959

OWN - NLM

STAT- PubMed-not-MEDLINE

LR - 20241009

IS - 1930-0433 (Print)

IS - 1930-0433 (Electronic)

IS - 1930-0433 (Linking)

VI - 19

IP - 12

DP - 2024 Dec

TI - Radiologic findings of an adolescent epithelioid inflammatory myofibroblastic

sarcoma.

PG - 6199-6204

LID - 10.1016/j.radcr.2024.08.143 [doi]

AB - Epithelioid inflammatory myofibroblastic sarcoma, a variant of the inflammatory

myofibroblastic tumor, is a rare tumor that is not well described in the

radiologic literature. We present a case of a 14-year-old male adolescent who

presented with fever, fatigue, and weight loss symptoms and was found to have an

abdominal mass on contrast enhanced CT. Initial differentials included lymphoma,

pheochromocytoma, desmoid, and sarcoma, and pathological evaluation revealed an

epithelioid inflammatory myofibroblastic sarcoma. The mass was separate from the

surrounding structures of the left upper abdomen with unique radiologic features

not previously described in the literature. Prior literature examples of

epithelioid inflammatory myofibroblastic sarcoma described a heterogenous,

enhancing lobulated mass, and our case was a lobulated, avidly enhancing

homogenous mass on CT with surrounding inflammation and avid uptake on PET/CT. In

addition to the imaging features, we describe the surgical findings, the

pathologic features of the tumor, and the oncologic treatment of this patient.

This case highlights the importance of including rare tumors such as epithelioid

inflammatory myofibroblastic sarcoma as a potential differential consideration of

an avidly enhancing homogenous abdominal mass in an adolescent.

CI - © 2024 The Authors. Published by Elsevier Inc. on behalf of University of

Washington.

FAU - Zezoff, David

AU - Zezoff D

AD - Department of Radiology, Saint Vincent Hospital, Worcester, MA 01608, USA.

FAU - Lowas, Stefanie

AU - Lowas S

AD - Department of Pediatrics, Division of Oncology, UMass Memorial Health, Worcester,

MA 01655, USA.

FAU - Cleary, Muriel

AU - Cleary M

AD - Department of Surgery, Division of Pediatric Surgery, UMass Memorial Health,

Worcester, MA 01655, USA.

FAU - Akalin, Ali

AU - Akalin A

AD - Department of Pathology, UMass Memorial Health, Worcester, MA 01655, USA.

FAU - Riaz, Farhana

AU - Riaz F

AD - Department of Radiology, Division of Pediatric Radiology, UMass Memorial Health,

Worcester, MA 01655, USA.

FAU - Gauguet, Jean-Marc

AU - Gauguet JM

AD - Department of Radiology, Division of Pediatric Radiology, UMass Memorial Health,

Worcester, MA 01655, USA.

LA - eng

PT - Case Reports

PT - Journal Article

DEP - 20240923

PL - Netherlands

TA - Radiol Case Rep

JT - Radiology case reports

JID - 101467888

PMC - PMC11456812

OTO - NOTNLM

OT - Anaplastic lymphoma kinase

OT - Crizotinib

OT - Epithelioid inflammatory myofibroblastic sarcoma

OT - Inflammatory myofibroblastic tumor

OT - Pediatrics

EDAT- 2024/10/08 10:55

MHDA- 2024/10/08 10:56

PMCR- 2024/09/23

CRDT- 2024/10/08 04:45

PHST- 2024/06/09 00:00 [received]

PHST- 2024/08/26 00:00 [revised]

PHST- 2024/08/28 00:00 [accepted]

PHST- 2024/10/08 10:56 [medline]

PHST- 2024/10/08 10:55 [pubmed]

PHST- 2024/10/08 04:45 [entrez]

PHST- 2024/09/23 00:00 [pmc-release]

AID - S1930-0433(24)00926-9 [pii]

AID - 10.1016/j.radcr.2024.08.143 [doi]

PST - epublish

SO - Radiol Case Rep. 2024 Sep 23;19(12):6199-6204. doi: 10.1016/j.radcr.2024.08.143.

eCollection 2024 Dec.

PMID- 39372869

OWN - NLM

STAT- PubMed-not-MEDLINE

LR - 20241008

IS - 2234-943X (Print)

IS - 2234-943X (Electronic)

IS - 2234-943X (Linking)

VI - 14

DP - 2024

TI - Uterine inflammatory myofibroblastic tumor: a retrospective analysis.

PG - 1461092

LID - 10.3389/fonc.2024.1461092 [doi]

LID - 1461092

AB - OBJECTIVE: Uterine inflammatory myofibroblastic tumor (UIMT) is a rare tumor of

the female reproductive tract with uncertain malignant potential. Previous case

series reports have limited our understanding of its diagnosis and treatment.

Therefore, we conducted a retrospective analysis of patient files at West China

Second University Hospital, Sichuan University to contribute valuable clinical

insights to future treatment strategies for this disease. METHOD: We

comprehensively reviewed patient files of individuals diagnosed with UIMT from

January 1st, 2013 to May 1st, 2023. RESULTS: We included twenty-seven cases of

uterine inflammatory myofibroblastic tumor in our study. Of these, 51.85% (14

cases) were diagnosed with abnormal uterine bleeding, 2 cases had dysmenorrhea,

and 12 were unexpectedly diagnosed with suspected uterine fibroids. Ten cases

performed total hysterectomy, and 17 cases underwent lesion resection. The

positive rate of anaplastic lymphoma kinase (ALK) immunohistochemistry reached

96.3%. After a median of 8 months follow-up time, all patients were disease-free

and had survived. CONCLUSION: Uterine inflammatory myofibroblastic tumor is

easily misdiagnosed, making its diagnosis challenging. Histological features,

immunohistochemical results, and molecular confirmation using fluorescence in

situ hybridization (FISH) or Next-generation sequencing should be used to confirm

the diagnosis. Positive ALK immunohistochemistry, ALK rearrangement, ALK fusion

are helpful in diagnosis and ALK inhibitor therapy. Total hysterectomy is often

performed for women who do not require fertility, while lesion resection and

close follow-up may be considered for those who require fertility preservation.

CI - Copyright © 2024 Bai, Han, Zheng and Chen.

FAU - Bai, Liping

AU - Bai L

AD - Department of Obstetrics and Gynecology, West China Second University Hospital,

Sichuan University, Chengdu, China.

AD - Key Laboratory of Birth defects and Related Diseases of Women and Children

(Sichuan University), Ministry of Education, Chengdu, China.

FAU - Han, Ling

AU - Han L

AD - Department of Obstetrics and Gynecology, West China Second University Hospital,

Sichuan University, Chengdu, China.

AD - Key Laboratory of Birth defects and Related Diseases of Women and Children

(Sichuan University), Ministry of Education, Chengdu, China.

FAU - Zheng, Ai

AU - Zheng A

AD - Department of Obstetrics and Gynecology, West China Second University Hospital,

Sichuan University, Chengdu, China.

AD - Key Laboratory of Birth defects and Related Diseases of Women and Children

(Sichuan University), Ministry of Education, Chengdu, China.

FAU - Chen, Yali

AU - Chen Y

AD - Department of Obstetrics and Gynecology, West China Second University Hospital,

Sichuan University, Chengdu, China.

AD - Key Laboratory of Birth defects and Related Diseases of Women and Children

(Sichuan University), Ministry of Education, Chengdu, China.

LA - eng

PT - Journal Article

DEP - 20240920

PL - Switzerland

TA - Front Oncol

JT - Frontiers in oncology

JID - 101568867

PMC - PMC11449678

OTO - NOTNLM

OT - anaplastic lymphoma kinase

OT - diagnosis

OT - inflammatory myofibroblastic tumor

OT - retrospective analysis

OT - uterine leiomyoma

OT - uterus

COIS- The authors declare that the research was conducted in the absence of any

commercial or financial relationships that could be construed as a potential

conflict of interest.

EDAT- 2024/10/07 12:57

MHDA- 2024/10/07 12:58

PMCR- 2024/01/01

CRDT- 2024/10/07 06:09

PHST- 2024/07/07 00:00 [received]

PHST- 2024/08/26 00:00 [accepted]

PHST- 2024/10/07 12:58 [medline]

PHST- 2024/10/07 12:57 [pubmed]

PHST- 2024/10/07 06:09 [entrez]

PHST- 2024/01/01 00:00 [pmc-release]

AID - 10.3389/fonc.2024.1461092 [doi]

PST - epublish

SO - Front Oncol. 2024 Sep 20;14:1461092. doi: 10.3389/fonc.2024.1461092. eCollection

2024.

PMID- 39347189

OWN - NLM

STAT- PubMed-not-MEDLINE

LR - 20241001

IS - 2168-8184 (Print)

IS - 2168-8184 (Electronic)

IS - 2168-8184 (Linking)

VI - 16

IP - 8

DP - 2024 Aug

TI - Epithelioid Inflammatory Myofibroblastic Sarcoma: A Report of a Rare Case.

PG - e68184

LID - 10.7759/cureus.68184 [doi]

LID - e68184

AB - Epithelioid inflammatory myofibroblastic sarcoma (EIMS), a variant of

inflammatory myofibroblastic tumor (IMT), is a rare malignant tumor commonly

associated with anaplastic lymphoma kinase (ALK) gene fusions and is aggressive

in nature with local recurrence. Here, we report a case of a 23-year-old female

who presented with a cough and, upon investigations, was found to have a mass in

the left upper lobe of the lung detected by chest computed tomography (CT).

Biopsy revealed EIMS with ALK and desmin protein expression. The patient

underwent a lobectomy via video-assisted thoracoscopic surgery (VATS). The

postoperative period was uneventful.

CI - Copyright © 2024, Ronanki et al.

FAU - Ronanki, Varun

AU - Ronanki V

AD - Pathology, NRI Medical College, Guntur, IND.

FAU - Tejeswini, Vaddatti

AU - Tejeswini V

AD - Pathology, NRI Medical College, Guntur, IND.

FAU - Venkata Renuka, Inuganti

AU - Venkata Renuka I

AD - Pathology, NRI Medical College, Guntur, IND.

FAU - Raheema, Shaik

AU - Raheema S

AD - Pathology, NRI Medical College, Guntur, IND.

FAU - S K Kanth, Bakkamanthala

AU - S K Kanth B

AD - Pathology, NRI Medical College, Guntur, IND.

LA - eng

PT - Case Reports

PT - Journal Article

DEP - 20240830

PL - United States

TA - Cureus

JT - Cureus

JID - 101596737

PMC - PMC11439187

OTO - NOTNLM

OT - anaplastic lymphoma kinase

OT - lung

OT - myofibroblastic

OT - rare

OT - sarcoma

COIS- Human subjects: Consent was obtained or waived by all participants in this study.

Conflicts of interest: In compliance with the ICMJE uniform disclosure form, all

authors declare the following: Payment/services info: All authors have declared

that no financial support was received from any organization for the submitted

work. Financial relationships: All authors have declared that they have no

financial relationships at present or within the previous three years with any

organizations that might have an interest in the submitted work. Other

relationships: All authors have declared that there are no other relationships or

activities that could appear to have influenced the submitted work.

EDAT- 2024/09/30 12:47

MHDA- 2024/09/30 12:48

PMCR- 2024/08/30

CRDT- 2024/09/30 10:01

PHST- 2024/08/30 00:00 [accepted]

PHST- 2024/09/30 12:48 [medline]

PHST- 2024/09/30 12:47 [pubmed]

PHST- 2024/09/30 10:01 [entrez]

PHST- 2024/08/30 00:00 [pmc-release]

AID - 10.7759/cureus.68184 [doi]

PST - epublish

SO - Cureus. 2024 Aug 30;16(8):e68184. doi: 10.7759/cureus.68184. eCollection 2024

Aug.

PMID- 39331206

OWN - NLM

STAT- PubMed-not-MEDLINE

LR - 20240930

IS - 2730-6011 (Electronic)

IS - 2730-6011 (Linking)

VI - 15

IP - 1

DP - 2024 Sep 27

TI - Thoracic epithelioid inflammatory myofibroblastic sarcoma: a rare and aggressive

disease with case report and literature review.

PG - 484

LID - 10.1007/s12672-024-01375-5 [doi]

LID - 484

AB - Epithelioid inflammatory myofibroblastic sarcoma (EIMS) is a rare subtype of

inflammatory myofibroblastic tumor, characterized to be an aggressive disease

with high frequency of ALK rearrangement, rapid recurrence, and poor prognosis.

Primary EIMS of thoracic origin is rarely observed. Herein, we described a case

of 28-year-old female developed primary EIMS in the anterior mediastinum with

hepatic metastasis. The EIMS displayed sheet-like growth of epithelioid and

spindle cells with enlarged nuclei, abundant and eosinophilic cytoplasm, and

infiltration of inflammatory cells. Immunohistochemical staining revealed

positive expression of ALK in the nuclear membrane, and ALK rearrangement was

identified by polymerase chain reaction assay. Alectinib showed partial response,

and achieved a meaningful survival benefit for four months. Based on this case

report and literature review, ALK inhibitor reveals promising activity on the

rare but aggressive EIMS. Awareness of EIMS in thoracic disease and its

clinicopathological features is essential to avoid erroneous diagnosis.

CI - © 2024. The Author(s).

FAU - Yang, Linke

AU - Yang L

AD - Department of Pathology, Shandong Cancer Hospital and Institute, Shandong First

Medical University, Shandong Academy of Medical Sciences, No. 440 Jiyan Road,

Jinan, 250117, People's Republic of China.

FAU - Li, Pei

AU - Li P

AD - Department of Respiratory Medical Oncology, Shandong Cancer Hospital and

Institute, Shandong First Medical University, Shandong Academy of Medical

Sciences, No. 440 Jiyan Road, Jinan, 250117, Jinan, People's Republic of China.

FAU - Liu, Runze

AU - Liu R

AD - Department of Radiation Oncology, Shandong Cancer Hospital and Institute,

Shandong First Medical University, Shandong Academy of Medical Sciences, No. 440

Jiyan Road, Jinan, 250117, People's Republic of China.

FAU - Feng, Baomin

AU - Feng B

AD - Department of Medical Imaging, Shandong Cancer Hospital and Institute, Shandong

First Medical University, Shandong Academy of Medical Sciences, No. 440 Jiyan

Road, Jinan, 250117, People's Republic of China.

FAU - Mao, Huiqing

AU - Mao H

AD - Department of Respiratory Medical Oncology, Shandong Cancer Hospital and

Institute, Shandong First Medical University, Shandong Academy of Medical

Sciences, No. 440 Jiyan Road, Jinan, 250117, Jinan, People's Republic of China.

FAU - Tang, Xiaoyong

AU - Tang X

AD - Department of Respiratory Medical Oncology, Shandong Cancer Hospital and

Institute, Shandong First Medical University, Shandong Academy of Medical

Sciences, No. 440 Jiyan Road, Jinan, 250117, Jinan, People's Republic of China.

FAU - Yang, Guangjian

AU - Yang G

AD - Department of Respiratory Medical Oncology, Shandong Cancer Hospital and

Institute, Shandong First Medical University, Shandong Academy of Medical

Sciences, No. 440 Jiyan Road, Jinan, 250117, Jinan, People's Republic of China.

yekong0806@126.com.

LA - eng

PT - Journal Article

DEP - 20240927

PL - United States

TA - Discov Oncol

JT - Discover oncology

JID - 101775142

PMC - PMC11436537

OTO - NOTNLM

OT - ALK rearrangement

OT - Alectinib

OT - Epithelioid inflammatory myofibroblastic sarcoma

OT - Inflammatory myofibroblastic tumor

OT - Thoracic

COIS- The authors declare no competing interests.

EDAT- 2024/09/27 12:43

MHDA- 2024/09/27 12:44

PMCR- 2024/09/27

CRDT- 2024/09/27 11:17

PHST- 2024/06/25 00:00 [received]

PHST- 2024/09/19 00:00 [accepted]

PHST- 2024/09/27 12:44 [medline]

PHST- 2024/09/27 12:43 [pubmed]

PHST- 2024/09/27 11:17 [entrez]

PHST- 2024/09/27 00:00 [pmc-release]

AID - 10.1007/s12672-024-01375-5 [pii]

AID - 1375 [pii]

AID - 10.1007/s12672-024-01375-5 [doi]

PST - epublish

SO - Discov Oncol. 2024 Sep 27;15(1):484. doi: 10.1007/s12672-024-01375-5.

PMID- 39281378

OWN - NLM

STAT- PubMed-not-MEDLINE

LR - 20240918

IS - 2234-943X (Print)

IS - 2234-943X (Electronic)

IS - 2234-943X (Linking)

VI - 14

DP - 2024

TI - Epithelioid inflammatory myofibroblastic sarcoma treated with Alectinib: a case

report and literature review.

PG - 1412225

LID - 10.3389/fonc.2024.1412225 [doi]

LID - 1412225

AB - Epithelioid inflammatory myofibroblastic sarcoma (EIMS) is an extremely rare and

aggressive form of inflammatory myofibroblastic tumor. Clinically, it has a high

risk of relapse and peripheral organ infiltration, and it responds poorly to

conventional chemotherapy. Anaplastic lymphoma kinase (ALK) inhibitors are

currently the most effective targeted therapy for EIMS. This report discusses a

typical case of abdominal EIMS in a 43-year-old woman. The tumors recurred

rapidly within one month after surgery. Alectinib was promptly administered upon

diagnosis. However, the patient developed a severe allergic reaction to the

medication. After a comprehensive assessment and symptomatic treatment, her

condition stabilized, leading to a favorable prognosis. This study summarizes

cases of abdominal EIMS, highlights the successful use of Alectinib for

treatment, and discusses the management of medication-related complications.

CI - Copyright © 2024 Wu, Zhu, Yan, Niu, Chen, Ning and Zhang.

FAU - Wu, Xinchun

AU - Wu X

AD - Department of Gastrointestinal Surgery, Peking University International Hospital,

Beijing, China.

FAU - Zhu, Junxi

AU - Zhu J

AD - Department of Gastrointestinal Surgery, Peking University International Hospital,

Beijing, China.

FAU - Yan, Yichao

AU - Yan Y

AD - Department of Gastrointestinal Surgery, Peking University International Hospital,

Beijing, China.

FAU - Niu, Dongfeng

AU - Niu D

AD - Department of Pathology, Peking University Cancer Hospital, Beijing, China.

FAU - Chen, Lin

AU - Chen L

AD - Department of Gastrointestinal Surgery, Peking University International Hospital,

Beijing, China.

FAU - Ning, Ning

AU - Ning N

AD - Department of Gastrointestinal Surgery, Peking University International Hospital,

Beijing, China.

FAU - Zhang, Yankai

AU - Zhang Y

AD - Department of Gastrointestinal Surgery, Peking University International Hospital,

Beijing, China.

LA - eng

PT - Case Reports

PT - Journal Article

DEP - 20240830

PL - Switzerland

TA - Front Oncol

JT - Frontiers in oncology

JID - 101568867

PMC - PMC11392682

OTO - NOTNLM

OT - Alectinib

OT - anaplastic lymphoma kinase

OT - drug allergy

OT - epithelioid inflammatory myofibroblastic sarcoma

OT - inflammatory myofibroblastic tumor

COIS- The authors declare that the research was conducted in the absence of any

commercial or financial relationships that could be construed as a potential

conflict of interest.

EDAT- 2024/09/17 10:45

MHDA- 2024/09/17 10:46

PMCR- 2024/01/01

CRDT- 2024/09/16 06:14

PHST- 2024/04/04 00:00 [received]

PHST- 2024/08/13 00:00 [accepted]

PHST- 2024/09/17 10:46 [medline]

PHST- 2024/09/17 10:45 [pubmed]

PHST- 2024/09/16 06:14 [entrez]

PHST- 2024/01/01 00:00 [pmc-release]

AID - 10.3389/fonc.2024.1412225 [doi]

PST - epublish

SO - Front Oncol. 2024 Aug 30;14:1412225. doi: 10.3389/fonc.2024.1412225. eCollection

2024.

PMID- 39280126

OWN - NLM

STAT- PubMed-not-MEDLINE

LR - 20240917

IS - 2214-4420 (Print)

IS - 2214-4420 (Electronic)

IS - 2214-4420 (Linking)

VI - 56

DP - 2024 Sep

TI - Urachal inflammatory myofibroblastic tumor with FN1: : ALK fusion: A case report

and literature review.

PG - 102844

LID - 10.1016/j.eucr.2024.102844 [doi]

LID - 102844

AB - Urachal tumors are rare and comprise of both benign and malignant neoplasms.

Epithelial origin tumors are more common than mesenchymal origin tumors. We

report a case Urachal inflammatory myofibroblastic tumor (IMFT) in a 12 year old

boy who presented with symptoms of lower abdominal pain and burning micturition.

Upon evaluation was found to have a soft tissue mass anterior to urinary bladder

wall. A laparoscopic excision of tumor was done. Histopathological and

immunohistochemical examination confirmed the diagnosis of IMFT. Next generation

sequencing identified FN1-ALK gene fusion.

CI - © 2024 The Authors. Published by Elsevier Inc.

FAU - Tara, Nair

AU - Tara N

AD - Department of Oncopathology, The Gujarat Cancer and Research Institute, India.

FAU - Mehta, Shailee

AU - Mehta S

AD - Department of Oncopathology, The Gujarat Cancer and Research Institute, India.

FAU - Trivedi, Priti P

AU - Trivedi PP

AD - Department of Oncopathology, The Gujarat Cancer and Research Institute, India.

FAU - Patel, Keval

AU - Patel K

AD - Department of Urology, The Gujarat Cancer and Research Institute, India.

FAU - Trivedi, Trupti

AU - Trivedi T

AD - Molecular diagnostics and Research Lab 1, Cancer Biology department, The Gujarat

Cancer and Research Institute, India.

LA - eng

PT - Case Reports

PT - Journal Article

DEP - 20240828

PL - United States

TA - Urol Case Rep

JT - Urology case reports

JID - 101626357

PMC - PMC11402050

EDAT- 2024/09/16 06:22

MHDA- 2024/09/16 06:23

PMCR- 2024/08/28

CRDT- 2024/09/16 05:59

PHST- 2024/07/29 00:00 [received]

PHST- 2024/08/27 00:00 [accepted]

PHST- 2024/09/16 06:23 [medline]

PHST- 2024/09/16 06:22 [pubmed]

PHST- 2024/09/16 05:59 [entrez]

PHST- 2024/08/28 00:00 [pmc-release]

AID - S2214-4420(24)00198-0 [pii]

AID - 102844 [pii]

AID - 10.1016/j.eucr.2024.102844 [doi]

PST - epublish

SO - Urol Case Rep. 2024 Aug 28;56:102844. doi: 10.1016/j.eucr.2024.102844.

eCollection 2024 Sep.

PMID- 39271327

OWN - NLM

STAT- Publisher

LR - 20240913

IS - 0219-3108 (Electronic)

IS - 1015-9584 (Linking)

DP - 2024 Sep 12

TI - Minimally invasive endoscopic approach to tracheal inflammatory myofibroblastic

tumor in a child.

LID - S1015-9584(24)02100-6 [pii]

LID - 10.1016/j.asjsur.2024.09.055 [doi]

FAU - Zhou, Li

AU - Zhou L

AD - Pediatric Department, Zigong First People's Hospital, Zigong, Sichuan, People's

Republic of China.

FAU - Wang, Gang

AU - Wang G

AD - Traditional Chinese Medicine Department, Zigong First People's Hospital, People's

Republic of China. Electronic address: 342358533@qq.com.

LA - eng

PT - Letter

DEP - 20240912

PL - Netherlands

TA - Asian J Surg

JT - Asian journal of surgery

JID - 8900600

SB - IM

OTO - NOTNLM

OT - Cough

OT - Inflammatory myofibroblastic tumor

OT - Minimally Invasive endoscopic

COIS- Declaration of competing interest A conflict of interest occurs when an

individual's objectivity is potentially compromised by a desire for financial

gain, prominence, professional advancement or a successful outcome. ASJSUR

Editors strive to ensure that what is published in the Journal is as balanced,

objective and evidence-based as possible. Since it can be difficult to

distinguish between an actual conflict of interest and a perceived conflict of

interest, the Journal requires authors to disclose all and any potential

conflicts of interest.

EDAT- 2024/09/14 10:47

MHDA- 2024/09/14 10:47

CRDT- 2024/09/13 21:56

PHST- 2024/08/14 00:00 [received]

PHST- 2024/09/05 00:00 [accepted]

PHST- 2024/09/14 10:47 [medline]

PHST- 2024/09/14 10:47 [pubmed]

PHST- 2024/09/13 21:56 [entrez]

AID - S1015-9584(24)02100-6 [pii]

AID - 10.1016/j.asjsur.2024.09.055 [doi]

PST - aheadofprint

SO - Asian J Surg. 2024 Sep 12:S1015-9584(24)02100-6. doi:

10.1016/j.asjsur.2024.09.055.

PMID- 39270376

OWN - NLM

STAT- PubMed-not-MEDLINE

LR - 20240928

IS - 2210-2612 (Print)

IS - 2210-2612 (Electronic)

IS - 2210-2612 (Linking)

VI - 123

DP - 2024 Oct

TI - Unusual gastric localization of an inflammatory myofibroblastic tumor: A case

report and review of the literature.

PG - 110262

LID - S2210-2612(24)01043-5 [pii]

LID - 10.1016/j.ijscr.2024.110262 [doi]

LID - 110262

AB - INTRODUCTION AND IMPORTANCE: Primary inflammatory myofibroblastic tumor is a rare

subgroup of mesenchymal tumors. Gastric localization is extremely rare, and

patients may present with abdominal pain and a palpable abdominal mass. Here, we

present a case of gastric inflammatory myofibroblastic tumor revealed by

abdominal pain, which was treated with wide local excision. CASE PRESENTATION:

This report illustrates the case of a 55-year-old female who presented with

abdominal pain. Imageology and gastrointestinal endoscopy revealed a posterior

gastric mass, which was treated with wide local excision. Immunohistochemical

analysis of the specimen confirmed the diagnosis of inflammatory myofibroblastic

gastric tumor. The patient had an uneventful postoperative course and she

remained in remission after 6 months of follow-up. DISCUSSION: Inflammatory

myofibroblastic tumor is a very rare mesenchymal tumor that usually affects

children and young adults. Gastric localization is also very rare and does not

typically cause specific clinical symptoms. Surgery is the mainstay of treatment,

and resection depends on the size and location of the lesion. The definitive

diagnosis is confirmed by immunohistochemical analysis of the specimen.

CONCLUSION: Myofibroblastic tumor is a rare subgroup of mesenchymal tumor.

Gastric localization is an uncommon presentation. Surgery is the mainstay of the

treatment. Histological analysis of the surgical specimen is essential for a

final diagnosis.

CI - Copyright © 2024 The Author(s). Published by Elsevier Ltd.. All rights reserved.

FAU - Laamiri, Ghazi

AU - Laamiri G

AD - Department of General Surgery, Hospital Mohamed Taher Maamouri, Nabeul, Tunisia;

University Tunis El Manar, Faculty of Medicine of Tunis, Tunisia.

FAU - Tormane, Mohamed Amine

AU - Tormane MA

AD - Department of General Surgery, Hospital Mohamed Taher Maamouri, Nabeul, Tunisia;

University Tunis El Manar, Faculty of Medicine of Tunis, Tunisia. Electronic

address: medaminetormane307@gmail.com.

FAU - Dougaz, Amel

AU - Dougaz A

AD - Department of Anatomopathology, Hospital Mohamed Taher Maamouri, Nabeul, Tunisia;

University Tunis El Manar, Faculty of Medicine of Tunis, Tunisia.

FAU - Bani, Amina

AU - Bani A

AD - Department of Anatomopathology, Hospital Mohamed Taher Maamouri, Nabeul, Tunisia;

University Tunis El Manar, Faculty of Medicine of Tunis, Tunisia.

FAU - Bouassida, Mahdi

AU - Bouassida M

AD - Department of General Surgery, Hospital Mohamed Taher Maamouri, Nabeul, Tunisia;

University Tunis El Manar, Faculty of Medicine of Tunis, Tunisia.

FAU - Touinsi, Hassen

AU - Touinsi H

AD - Department of General Surgery, Hospital Mohamed Taher Maamouri, Nabeul, Tunisia;

University Tunis El Manar, Faculty of Medicine of Tunis, Tunisia.

LA - eng

PT - Case Reports

PT - Journal Article

DEP - 20240910

PL - Netherlands

TA - Int J Surg Case Rep

JT - International journal of surgery case reports

JID - 101529872

PMC - PMC11417201

OTO - NOTNLM

OT - Benign tumors

OT - Case report

OT - Gastric

OT - Inflammatory

OT - Myofibroblastic tumor

COIS- Declaration of competing interest No conflicts of interest.

EDAT- 2024/09/14 10:45

MHDA- 2024/09/14 10:46

PMCR- 2024/09/10

CRDT- 2024/09/13 18:00

PHST- 2024/06/24 00:00 [received]

PHST- 2024/09/02 00:00 [revised]

PHST- 2024/09/06 00:00 [accepted]

PHST- 2024/09/14 10:46 [medline]

PHST- 2024/09/14 10:45 [pubmed]

PHST- 2024/09/13 18:00 [entrez]

PHST- 2024/09/10 00:00 [pmc-release]

AID - S2210-2612(24)01043-5 [pii]

AID - 110262 [pii]

AID - 10.1016/j.ijscr.2024.110262 [doi]

PST - ppublish

SO - Int J Surg Case Rep. 2024 Oct;123:110262. doi: 10.1016/j.ijscr.2024.110262. Epub

2024 Sep 10.

PMID- 39247479

OWN - NLM

STAT- PubMed-not-MEDLINE

LR - 20240910

IS - 1930-0433 (Print)

IS - 1930-0433 (Electronic)

IS - 1930-0433 (Linking)

VI - 19

IP - 11

DP - 2024 Nov

TI - Mesenteric inflammatory pseudotumor: A rare case report and review of the

literature.

PG - 4945-4949

LID - 10.1016/j.radcr.2024.07.124 [doi]

AB - Inflammatory pseudotumor (IP) is a rare type of benign tumor. Although initially

identified in the lung, it has now been identified in a number of somatic and

visceral sites, but mesenteric presentation is uncommon and has a variable

clinical presentation. As inflammatory pseudotumor mimic malignancy both

clinically and radiologically, the radiologist should be familiar with this

entity. The only effective treatment is complete surgical resection. We present

the case of a 55-year-old woman who presented with chronic abdominal pain and was

diagnosed with a mesenteric inflammatory pseudotumor, in an attempt to illustrate

the different imaging aspects of this benign condition in ultrasound, computed

tomography and magnetic resonance imaging, and to simplify the description of

these tumors.

CI - © 2024 The Authors. Published by Elsevier Inc. on behalf of University of

Washington.

FAU - Faraj, Chaymae

AU - Faraj C

AD - Department of Radiology, Ibn Sina Hospital, Faculty of medicine and pharmacy,

Mohammed V University, Rabat, Morocco.

FAU - Imrani, Kaoutar

AU - Imrani K

AD - Department of Radiology, Ibn Sina Hospital, Faculty of medicine and pharmacy,

Mohammed V University, Rabat, Morocco.

FAU - Essetti, Sara

AU - Essetti S

AD - Department of Radiology, Ibn Sina Hospital, Faculty of medicine and pharmacy,

Mohammed V University, Rabat, Morocco.

FAU - Chait, Fatima

AU - Chait F

AD - Department of Radiology, Ibn Sina Hospital, Faculty of medicine and pharmacy,

Mohammed V University, Rabat, Morocco.

FAU - Lahlou, Chaimae

AU - Lahlou C

AD - Department of Radiology, Ibn Sina Hospital, Faculty of medicine and pharmacy,

Mohammed V University, Rabat, Morocco.

FAU - M Billah, Nabil

AU - M Billah N

AD - Department of Radiology, Ibn Sina Hospital, Faculty of medicine and pharmacy,

Mohammed V University, Rabat, Morocco.

FAU - Nassar, Ittimade

AU - Nassar I

AD - Department of Radiology, Ibn Sina Hospital, Faculty of medicine and pharmacy,

Mohammed V University, Rabat, Morocco.

FAU - A Babana, Amina

AU - A Babana A

AD - Department of Surgery « A », Ibn Sina Hospital, Faculty of medicine and pharmacy,

Mohammed V University, Rabat, Morocco.

FAU - O Touhami, Youssef

AU - O Touhami Y

AD - Department of Surgery « A », Ibn Sina Hospital, Faculty of medicine and pharmacy,

Mohammed V University, Rabat, Morocco.

FAU - El Malki, Hadj Omar

AU - El Malki HO

AD - Department of Surgery « A », Ibn Sina Hospital, Faculty of medicine and pharmacy,

Mohammed V University, Rabat, Morocco.

FAU - Ifrine, Lahcen

AU - Ifrine L

AD - Department of Surgery « A », Ibn Sina Hospital, Faculty of medicine and pharmacy,

Mohammed V University, Rabat, Morocco.

FAU - Belkouchi, Abdelkader

AU - Belkouchi A

AD - Department of Surgery « A », Ibn Sina Hospital, Faculty of medicine and pharmacy,

Mohammed V University, Rabat, Morocco.

FAU - Belkouchi, Omar

AU - Belkouchi O

AD - Department of Surgery « A », Ibn Sina Hospital, Faculty of medicine and pharmacy,

Mohammed V University, Rabat, Morocco.

FAU - El Mohtarim, Rihane

AU - El Mohtarim R

AD - Department of Pathology, Ibn Sina Hospital, Faculty of medicine and pharmacy,

Mohammed V University, Rabat, Morocco.

FAU - Derqaoui, Sabrine

AU - Derqaoui S

AD - Department of Pathology, Ibn Sina Hospital, Faculty of medicine and pharmacy,

Mohammed V University, Rabat, Morocco.

FAU - Bernoussi, Zakiya

AU - Bernoussi Z

AD - Department of Pathology, Ibn Sina Hospital, Faculty of medicine and pharmacy,

Mohammed V University, Rabat, Morocco.

LA - eng

PT - Case Reports

PT - Journal Article

DEP - 20240817

PL - Netherlands

TA - Radiol Case Rep

JT - Radiology case reports

JID - 101467888

PMC - PMC11378093

OTO - NOTNLM

OT - Inflammatory pseudotumor

OT - Mesentery, Diagnosis, Imaging

EDAT- 2024/09/09 06:42

MHDA- 2024/09/09 06:43

PMCR- 2024/08/17

CRDT- 2024/09/09 05:10

PHST- 2024/07/09 00:00 [received]

PHST- 2024/07/18 00:00 [revised]

PHST- 2024/07/21 00:00 [accepted]

PHST- 2024/09/09 06:43 [medline]

PHST- 2024/09/09 06:42 [pubmed]

PHST- 2024/09/09 05:10 [entrez]

PHST- 2024/08/17 00:00 [pmc-release]

AID - S1930-0433(24)00710-6 [pii]

AID - 10.1016/j.radcr.2024.07.124 [doi]

PST - epublish

SO - Radiol Case Rep. 2024 Aug 17;19(11):4945-4949. doi: 10.1016/j.radcr.2024.07.124.

eCollection 2024 Nov.

PMID- 39214809

OWN - NLM

STAT- Publisher

LR - 20240830

IS - 0219-3108 (Electronic)

IS - 1015-9584 (Linking)

DP - 2024 Aug 29

TI - Surgical treatment of uterine inflammatory myofibroblastic tumor with local

invasion: A case report.

LID - S1015-9584(24)01846-3 [pii]

LID - 10.1016/j.asjsur.2024.08.106 [doi]

FAU - Mu, Dan

AU - Mu D

AD - Department of Obstetrics and Gynecology, West China Second University Hospital,

Sichuan University, Chengdu, 610041, China; Department of Medical Genetics /

Prenatal Diagnostic Center, West China Second University Hospital, Sichuan

University, Chengdu, 610041, China; Key Laboratory of Birth Defects and Related

Diseases of Women and Children (Sichuan University), Ministry of Education,

Sichuan University, Chengdu, 610041, China.

FAU - Tang, Furong

AU - Tang F

AD - Department of Obstetrics and Gynecology, West China Second University Hospital,

Sichuan University, Chengdu, 610041, China; Key Laboratory of Birth Defects and

Related Diseases of Women and Children (Sichuan University), Ministry of

Education, Sichuan University, Chengdu, 610041, China.

FAU - Chen, Yali

AU - Chen Y

AD - Department of Obstetrics and Gynecology, West China Second University Hospital,

Sichuan University, Chengdu, 610041, China; Key Laboratory of Birth Defects and

Related Diseases of Women and Children (Sichuan University), Ministry of

Education, Sichuan University, Chengdu, 610041, China.

FAU - Chen, Hualian

AU - Chen H

AD - Mianzhu Hospital of Traditional Chinese Medicine, Deyang, 618200, China.

Electronic address: hualianchen624@163.com.

LA - eng

PT - Letter

DEP - 20240829

PL - Netherlands

TA - Asian J Surg

JT - Asian journal of surgery

JID - 8900600

SB - IM

OTO - NOTNLM

OT - ALK

OT - Gynecologic neoplasms

OT - Inflammatory myofibroblastic tumor

OT - Mesenchymal tumor

COIS- Declaration of competing interest The authors declare that they have no competing

interests.

EDAT- 2024/08/31 09:50

MHDA- 2024/08/31 09:50

CRDT- 2024/08/30 22:00

PHST- 2024/07/09 00:00 [received]

PHST- 2024/08/15 00:00 [accepted]

PHST- 2024/08/31 09:50 [medline]

PHST- 2024/08/31 09:50 [pubmed]

PHST- 2024/08/30 22:00 [entrez]

AID - S1015-9584(24)01846-3 [pii]

AID - 10.1016/j.asjsur.2024.08.106 [doi]

PST - aheadofprint

SO - Asian J Surg. 2024 Aug 29:S1015-9584(24)01846-3. doi:

10.1016/j.asjsur.2024.08.106.

PMID- 39204372

OWN - NLM

STAT- PubMed-not-MEDLINE

LR - 20240903

IS - 1999-4923 (Print)

IS - 1999-4923 (Electronic)

IS - 1999-4923 (Linking)

VI - 16

IP - 8

DP - 2024 Aug 1

TI - Commercialization of the Xalkori Pediatric Multiparticulate Product Using

Quality-by-Design Principles.

LID - 10.3390/pharmaceutics16081027 [doi]

LID - 1027

AB - A pediatric dosage form for crizotinib (Xalkori) was commercialized using

quality-by-design principles in a material-sparing fashion. The dosage form

consists of spherical multiparticulates (microspheres or pellets) that are coated

and encapsulated in capsules for opening. The crizotinib (Xalkori)-coated pellet

product is approved in the US for pediatric patients 1 year of age and older and

young adults with relapsed or refractory, systemic anaplastic large cell lymphoma

(ALCL) and unresectable, recurrent, or refractory inflammatory myofibroblastic

tumor (IMT) that is ALK-positive. The product is also approved in the US for

adult patients with non-small cell lung cancer (NSCLC) who are unable to swallow

intact capsules. The lipid multiparticulate is composed of a lipid matrix, a

dissolution enhancer, and an active pharmaceutical ingredient (API). The API,

which remains crystalline, is embedded within the microsphere at a 60% drug

loading in the uncoated lipid multiparticulate to enable dose flexibility. The

melt spray congealing technique using a rotary atomizer is used to manufacture

the lipid multiparticulate. Following melt spray congealing, a barrier coating is

applied via fluid bed coating. Due to their particle size and content uniformity,

this dosage form provides the dosing flexibility and swallowability needed for

the pediatric population. The required pediatric dose is achieved by opening the

capsules and combining doses of different encapsulated dose strengths, followed

by administration of the multiparticulates directly to the mouth. The

encapsulation process was optimized through equipment modifications and by using

a design of experiments approach to understand the operating space. A limited

number of development batches produced using commercial-scale equipment were

leveraged to design, understand, and verify the manufacturing process space. The

quality by design and material-sparing approach taken to design the melt spray

congeal and encapsulation manufacturing processes resulted in a pediatric product

with exceptional content uniformity (a 95% confidence and 99% probability of

passing USP <905> content uniformity testing for future batches).

FAU - Bartlett, Jeremy

AU - Bartlett J

AD - Pfizer, Eastern Point Road, Groton, CT 06340, USA.

FAU - Culver, Natalie

AU - Culver N

AD - Pfizer, Eastern Point Road, Groton, CT 06340, USA.

FAU - Zhang, Xiang

AU - Zhang X

AD - Pfizer, Eastern Point Road, Groton, CT 06340, USA.

FAU - Waybrant, Brett

AU - Waybrant B

AD - Lonza, 63045 NE Corporate Place, Bend, OR 97701, USA.

FAU - Sullivan, Hannah

AU - Sullivan H

AD - Lonza, 63045 NE Corporate Place, Bend, OR 97701, USA.

FAU - Howell, Logan

AU - Howell L

AD - Lonza, 63045 NE Corporate Place, Bend, OR 97701, USA.

LA - eng

PT - Journal Article

DEP - 20240801

PL - Switzerland

TA - Pharmaceutics

JT - Pharmaceutics

JID - 101534003

PMC - PMC11360164

OTO - NOTNLM

OT - commercialization

OT - design of experiments (DoE)

OT - dose flexibility

OT - lipid multiparticulates

OT - material-sparing approach

OT - melt spray congeal (MSC)

OT - microspheres

OT - multiparticulates

OT - pediatrics

OT - pellets

OT - process optimization

OT - quality by design (QbD)

OT - taste masking

OT - uniformity

OT - volumetric dosator

COIS- Authors Jeremy Bartlett, Natalie Culver, and Xiang Zhang were employed by the

company Pfizer. Authors Brett Waybrant, Hannah Sullivan, and Logan Howell were

employed by the company Lonza. The authors declare no conflicts of interest.

EDAT- 2024/08/31 09:49

MHDA- 2024/08/31 09:50

PMCR- 2024/08/01

CRDT- 2024/08/29 01:29

PHST- 2024/06/28 00:00 [received]

PHST- 2024/07/23 00:00 [revised]

PHST- 2024/07/26 00:00 [accepted]

PHST- 2024/08/31 09:50 [medline]

PHST- 2024/08/31 09:49 [pubmed]

PHST- 2024/08/29 01:29 [entrez]

PHST- 2024/08/01 00:00 [pmc-release]

AID - pharmaceutics16081027 [pii]

AID - pharmaceutics-16-01027 [pii]

AID - 10.3390/pharmaceutics16081027 [doi]

PST - epublish

SO - Pharmaceutics. 2024 Aug 1;16(8):1027. doi: 10.3390/pharmaceutics16081027.

PMID- 39193013

OWN - NLM

STAT- PubMed-not-MEDLINE

LR - 20240829

IS - 2296-858X (Print)

IS - 2296-858X (Electronic)

IS - 2296-858X (Linking)

VI - 11

DP - 2024

TI - Imaging findings of inflammatory myofibroblastic tumor of sigmoid colon:

literature review and case report.

PG - 1461205

LID - 10.3389/fmed.2024.1461205 [doi]

LID - 1461205

AB - Inflammatory myofibroblastic tumor (IMT) is an intermediate tumor composed of

differentiated myofibroblastic spindle cells with inflammatory cell infiltration.

It can occur in all parts of the body, with the lungs being the most common,

while the tissues outside the lungs, including the sigmoid colon, are rare.

Herein, we present a case of a 10-year-old girl with sigmoid IMT who presented to

our hospital with abdominal pain. An abdominal computed tomography (CT) revealed

a well-defined, slightly low-density mass in her lower abdomen that was not

clearly demarcated from the sigmoid colon. The mass showed significant uneven

enhancement on contrast-enhanced CT and increased fluorine-18 fluorodeoxyglucose

((18)F-FDG) uptake on positron emission tomography (PET). Moreover, a systematic

review of the published literature on sigmoid IMT was conducted and its clinical

and radiographic features were summarized to increase the understanding of this

rare disease.

CI - Copyright © 2024 Hu, Zhao, Yu and Wang.

FAU - Hu, Xianwen

AU - Hu X

AD - Department of Nuclear Medicine, Affiliated Hospital of Zunyi Medical University,

Zunyi, China.

FAU - Zhao, Wei

AU - Zhao W

AD - Department of Pathology, Affiliated Hospital of Zunyi Medical University, Zunyi,

China.

FAU - Yu, Ronghua

AU - Yu R

AD - Department of Nuclear Medicine, Affiliated Hospital of Zunyi Medical University,

Zunyi, China.

FAU - Wang, Pan

AU - Wang P

AD - Department of Nuclear Medicine, Affiliated Hospital of Zunyi Medical University,

Zunyi, China.

LA - eng

PT - Case Reports

PT - Journal Article

DEP - 20240813

PL - Switzerland

TA - Front Med (Lausanne)

JT - Frontiers in medicine

JID - 101648047

PMC - PMC11347276

OTO - NOTNLM

OT - 18F-FDG

OT - CT

OT - PET/CT

OT - inflammatory myofibroblastic tumor

OT - sigmoid colon

COIS- The authors declare that the research was conducted in the absence of any

commercial or financial relationships that could be construed as a potential

conflict of interest.

EDAT- 2024/08/28 08:42

MHDA- 2024/08/28 08:43

PMCR- 2024/08/13

CRDT- 2024/08/28 04:05

PHST- 2024/07/08 00:00 [received]

PHST- 2024/07/29 00:00 [accepted]

PHST- 2024/08/28 08:43 [medline]

PHST- 2024/08/28 08:42 [pubmed]

PHST- 2024/08/28 04:05 [entrez]

PHST- 2024/08/13 00:00 [pmc-release]

AID - 10.3389/fmed.2024.1461205 [doi]

PST - epublish

SO - Front Med (Lausanne). 2024 Aug 13;11:1461205. doi: 10.3389/fmed.2024.1461205.

eCollection 2024.

PMID- 39171208

OWN - NLM

STAT- PubMed-not-MEDLINE

LR - 20240823

IS - 2314-4378 (Electronic)

IS - 2314-436X (Print)

IS - 2314-436X (Linking)

VI - 2024

DP - 2024

TI - Recurrent Inflammatory Myofibroblastic Tumor of Larynx Harboring a Novel

THBS1::ALK Fusion.

PG - 4937501

LID - 10.1155/2024/4937501 [doi]

LID - 4937501

AB - Inflammatory myofibroblastic tumor (IMT) is a rare soft tissue tumor primarily

occurring in the abdominopelvic region of young patients, and it is characterized

by spindle-shaped myofibroblasts, or fibroblasts surrounded by inflammatory

infiltrate. Herein, we report a case of a 24-year-old male with a firm submucosal

mass in the anterior right vocal fold diagnosed as an IMT that recurred 14 months

later. The tumor demonstrated a novel THBS1::ALK fusion containing Exons 1-7 of

the thrombospondin 1 (THBS1) gene fused to Exon 19 of the anaplastic lymphoma

kinase (ALK) gene via next-generation sequencing with the NextSeq sequencer. The

fusion of THBS1 to ALK potentially results in increased expression and

constitutive activation of the ALK kinase domain. These findings not only broaden

the repertoire of known ALK fusion partners implicated in tumorigenesis but also

provide a novel avenue for investigating the etiology of recurrent IMT by

considering this fusion event as a causal factor. To our knowledge, this is the

second case of IMT of the larynx with this novel mutation reported in the

literature and the first such case with a detailed description of this specific

fusion and clinical recurrence.

CI - Copyright © 2024 Namra Ajmal et al.

FAU - Ajmal, Namra

AU - Ajmal N

AUID- ORCID: 0000-0003-3016-3102

AD - Department of Pathology and Genomic Medicine Thomas Jefferson University

Hospital, Philadelphia, PA 19107, USA.

FAU - Gargano, Stacey M

AU - Gargano SM

AD - Department of Pathology and Genomic Medicine Thomas Jefferson University

Hospital, Philadelphia, PA 19107, USA.

FAU - Gosavi, Ujwala

AU - Gosavi U

AD - Department of Pathology and Genomic Medicine Thomas Jefferson University

Hospital, Philadelphia, PA 19107, USA.

FAU - Tuluc, Madalina

AU - Tuluc M

AD - Department of Pathology and Genomic Medicine Thomas Jefferson University

Hospital, Philadelphia, PA 19107, USA.

LA - eng

PT - Journal Article

PT - Review

DEP - 20240814

PL - United States

TA - Int J Genomics

JT - International journal of genomics

JID - 101605206

PMC - PMC11338662

OTO - NOTNLM

OT - ALK fusion

OT - IMT

OT - Inflammatory myofibroblastic tumor

OT - THBS1

OT - larynx

COIS- The authors declare no conflicts of interest.

EDAT- 2024/08/22 06:42

MHDA- 2024/08/22 06:43

PMCR- 2024/08/14

CRDT- 2024/08/22 04:49

PHST- 2024/01/05 00:00 [received]

PHST- 2024/07/01 00:00 [revised]

PHST- 2024/07/20 00:00 [accepted]

PHST- 2024/08/22 06:43 [medline]

PHST- 2024/08/22 06:42 [pubmed]

PHST- 2024/08/22 04:49 [entrez]

PHST- 2024/08/14 00:00 [pmc-release]

AID - 10.1155/2024/4937501 [doi]

PST - epublish

SO - Int J Genomics. 2024 Aug 14;2024:4937501. doi: 10.1155/2024/4937501. eCollection

2024.

PMID- 39144623

OWN - NLM

STAT- PubMed-not-MEDLINE

LR - 20240816

IS - 1663-9812 (Print)

IS - 1663-9812 (Electronic)

IS - 1663-9812 (Linking)

VI - 15

DP - 2024

TI - A case report: Pathological complete response to neoadjuvant lorlatinib for

Epithelioid inflammatory myofibroblastic sarcoma with EML4-ALK rearrangement.

PG - 1401428

LID - 10.3389/fphar.2024.1401428 [doi]

LID - 1401428

AB - Inflammatory myofibroblastic tumor (IMT) is a rare tumor originating from

mesenchymal tissue. Epithelioid inflammatory myofibroblastic sarcoma (EIMS)

represents a rare and particularly aggressive variant, associated with a worse

prognosis. Almost all EIMS cases exhibits activating anaplastic lymphoma kinase

(ALK) gene rearrangements, which suggests that EIMS patients may potentially

benefit from treatment with ALK tyrosine kinase inhibitors (TKIs). We presented a

case involving a 34-year-old woman who was diagnosed with mediastinal EIMS and

had a rare echinoderm microtubule-associated protein-like 4 (EML4) -ALK fusion.

Following 15 months of neoadjuvant lorlatinib treatment, the patient underwent a

complete surgical resection, resulting in a pathological complete response. Given

the heightened risk of postoperative recurrence associated with EIMS, the

patient's treatment plan included ongoing adjuvant therapy with lorlatinib. As of

the present moment, the patient has achieved an overall survival of over 2 years

with no observed tumor recurrence. Consequently, the case offers valuable

clinical evidence supporting the potential benefits of neoadjuvant lorlatinib

treatment for ALK-positive locally mediastinal EIMS patients, with a demonstrated

tolerable safety profile.

CI - Copyright © 2024 Zheng, Zhao, Ren, Xue, Yan and Huang.

FAU - Zheng, Yang

AU - Zheng Y

AD - Department of Thoracic Medical Oncology, Tianjin Medical University Cancer

Institute and Hospital, National Clinical Research Center for Cancer, Tianjin,

China.

AD - Key Laboratory of Cancer Prevention and Therapy, Tianjin's Clinical Research

Center for Cancer, Tianjin, China.

FAU - Zhao, Fanfei

AU - Zhao F

AD - Department of Thoracic Medical Oncology, Tianjin Medical University Cancer

Institute and Hospital, National Clinical Research Center for Cancer, Tianjin,

China.

AD - Key Laboratory of Cancer Prevention and Therapy, Tianjin's Clinical Research

Center for Cancer, Tianjin, China.

FAU - Ren, Yaqian

AU - Ren Y

AD - Department of Thoracic Medical Oncology, Tianjin Medical University Cancer

Institute and Hospital, National Clinical Research Center for Cancer, Tianjin,

China.

AD - Key Laboratory of Cancer Prevention and Therapy, Tianjin's Clinical Research

Center for Cancer, Tianjin, China.

FAU - Xue, Yaran

AU - Xue Y

AD - Department of Oncology, Tianjin Fourth Central Hospital, Tianjin, China.

FAU - Yan, Bing

AU - Yan B

AD - Department of Precision Oncology, Tianjin Cancer Hospital Airport Hospital,

Tianjin, China.

FAU - Huang, Chun

AU - Huang C

AD - Department of Thoracic Medical Oncology, Tianjin Medical University Cancer

Institute and Hospital, National Clinical Research Center for Cancer, Tianjin,

China.

AD - Key Laboratory of Cancer Prevention and Therapy, Tianjin's Clinical Research

Center for Cancer, Tianjin, China.

LA - eng

PT - Case Reports

PT - Journal Article

DEP - 20240731

PL - Switzerland

TA - Front Pharmacol

JT - Frontiers in pharmacology

JID - 101548923

PMC - PMC11321957

OTO - NOTNLM

OT - EML4-ALK

OT - Epithelioid inflammatory myofibroblastic sarcoma

OT - lorlatinib

OT - neoadjuvant treatment

OT - pathological complete response

COIS- The authors declare that the research was conducted in the absence of any

commercial or financial relationships that could be construed as a potential

conflict of interest.

EDAT- 2024/08/15 06:42

MHDA- 2024/08/15 06:43

PMCR- 2024/07/31

CRDT- 2024/08/15 04:51

PHST- 2024/03/18 00:00 [received]

PHST- 2024/07/22 00:00 [accepted]

PHST- 2024/08/15 06:43 [medline]

PHST- 2024/08/15 06:42 [pubmed]

PHST- 2024/08/15 04:51 [entrez]

PHST- 2024/07/31 00:00 [pmc-release]

AID - 1401428 [pii]

AID - 10.3389/fphar.2024.1401428 [doi]

PST - epublish

SO - Front Pharmacol. 2024 Jul 31;15:1401428. doi: 10.3389/fphar.2024.1401428.

eCollection 2024.

PMID- 39144250

OWN - NLM

STAT- PubMed-not-MEDLINE

LR - 20240816

IS - 1662-6575 (Print)

IS - 1662-6575 (Electronic)

IS - 1662-6575 (Linking)

VI - 17

IP - 1

DP - 2024 Jan-Dec

TI - New Perspectives in the Treatment of Inflammatory Myofibroblastic Tumor with ALK

Translocation: Case Report.

PG - 763-772

LID - 10.1159/000539739 [doi]

AB - INTRODUCTION: Inflammatory myofibroblastic tumor (IMT) is a rare entity,

classified within soft tissue sarcomas. It is an intermediate malignancy tumor,

which seldom presents as metastatic disease. The treatment of choice is surgery,

except in cases where surgery is not possible due to localization or if it

presents with metastatic disease. Approximately 50% of IMTs will exhibit ALK

translocation, providing a therapeutic target for these patients. CASE

PRESENTATION: A case is presented of a patient with metastatic IMT in complete

response to treatment with alectinib, maintained for over 4 years. CONCLUSION:

This case showed a long time complete response in patient with IMT treated with

alectinib.

CI - © 2024 The Author(s). Published by S. Karger AG, Basel.

FAU - Benedetti Pedroza, Johana

AU - Benedetti Pedroza J

AD - Medical Oncology Department, Virgen del Rocio University Hospital, Seville,

Spain.

FAU - Carrasco García, Irene

AU - Carrasco García I

AD - Medical Oncology Department, Virgen del Rocio University Hospital, Seville,

Spain.

FAU - Martínez Bernal, Gala

AU - Martínez Bernal G

AD - Medical Oncology Department, Virgen del Rocio University Hospital, Seville,

Spain.

FAU - Miras Rodriguez, Isabel

AU - Miras Rodriguez I

AD - Medical Oncology Department, Virgen del Rocio University Hospital, Seville,

Spain.

LA - eng

PT - Case Reports

PT - Journal Article

DEP - 20240720

PL - Switzerland

TA - Case Rep Oncol

JT - Case reports in oncology

JID - 101517601

PMC - PMC11324221

OTO - NOTNLM

OT - ALK inhibitor

OT - Alectinib

OT - Head and neck

OT - Inflammatory myofibroblastic tumor

COIS- Johana Benedetti Pedroza has collected research funding for clinical studies

(institutional) from PharmaMar, Eli Lilly and Company, AROG, Bayer, Eisai, Lixte,

Karyopharm, Deci-phera, GlaxoSmithKline, Novartis, Blueprint, Nektar, Forma,

Amgen, and Daichii-Sankyo. Irene Carrasco García has received personal fees for

advisory board, consulting, and travel expenses from Pharmamar. She has also

gotten research funding for clinical studies (institutional) from PharmaMar, Eli

Lilly and Company, AROG, Bayer, Eisai, Lixte, Karyopharm, Deci-phera,

GlaxoSmithKline, Novartis, Blueprint, Nektar, Forma, Amgen, and Daichii-Sankyo.

Gala Martínez Bernal has obtained research funding for clinical studies

(institutional) from PharmaMar, Eli Lilly and Company, AROG, Bayer, Eisai, Lixte,

Karyopharm, Deci-phera, GlaxoSmithKline, Novartis, Blueprint, Nektar, Forma,

Amgen, and Daichii-Sankyo. Lastly, Isabel Miras Rodriguez has obtained research

funding for clinical studies (institutional) from PharmaMar, Eli Lilly and

Company, AROG, Bayer, Eisai, Lixte, Karyopharm, Deci-phera, GlaxoSmithKline,

Novartis, Blueprint, Nektar, Forma, Amgen, and Daichii-Sankyo.

EDAT- 2024/08/15 06:42

MHDA- 2024/08/15 06:43

PMCR- 2024/07/20

CRDT- 2024/08/15 04:47

PHST- 2024/04/19 00:00 [received]

PHST- 2024/06/03 00:00 [accepted]

PHST- 2024/08/15 06:43 [medline]

PHST- 2024/08/15 06:42 [pubmed]

PHST- 2024/08/15 04:47 [entrez]

PHST- 2024/07/20 00:00 [pmc-release]

AID - 539739 [pii]

AID - 10.1159/000539739 [doi]

PST - epublish

SO - Case Rep Oncol. 2024 Jul 20;17(1):763-772. doi: 10.1159/000539739. eCollection

2024 Jan-Dec.

PMID- 39105041

OWN - NLM

STAT- PubMed-not-MEDLINE

LR - 20240807

IS - 2168-8184 (Print)

IS - 2168-8184 (Electronic)

IS - 2168-8184 (Linking)

VI - 16

IP - 7

DP - 2024 Jul

TI - Inflammatory Myofibroblastic Tumor of the Lung: A Report of a Rare Case.

PG - e63892

LID - 10.7759/cureus.63892 [doi]

LID - e63892

AB - The uncommon and mysterious pulmonary inflammatory myofibroblastic tumor (PIMT)

primarily affects children and young people. PIMT is characterized by the

proliferation of myofibroblastic spindle cells mixed with inflammatory cells. It

can resemble both benign and malignant disorders, both radiographically and

clinically. PIMT typically manifests as a solitary lung tumor. The genesis of the

tumor is linked to genetic anomalies, including those related to the ALK gene

(anaplastic lymphoma kinase); nonetheless, some cases are not ALK-positive,

indicating genetic variability. Clinically, patients may have non-specific

symptoms such as cough, chest pain, or hemoptysis, or they may not exhibit any

symptoms at all. In these cases, imaging tests may unintentionally reveal

unrelated conditions. From a histopathological perspective, PIMT is characterized

by a heterogeneous cellular makeup, encompassing lymphocytes, myofibroblasts,

plasma cells, and histiocytes, which generally exhibit a fascicular or storiform

pattern. The diagnosis is verified using immunohistochemical labeling, molecular

research, and histological examination. The cornerstone of treatment is still

surgical resection, which has a good prognosis and a low recurrence rate. On the

other hand, specific treatments, such as ALK inhibitors, have shown promise for

incurable or recurring instances. Even though PIMT usually has a benign history,

it is important to comprehend its biological behavior and molecular foundations

for precise diagnosis and efficient management. This underscores the need for

additional study into the pathophysiology and potential treatments of PIMT. This

report presents a case of a 53-year-old female who presented with complaints of

breathlessness and chest pain and was diagnosed with the condition accidentally.

CI - Copyright © 2024, Bhawani et al.

FAU - Bhawani, Jayashree

AU - Bhawani J

AD - Department of Pathology, Jawaharlal Nehru Medical College, Datta Meghe Institute

of Higher Education and Research, Wardha, IND.

FAU - Shukla, Samarth

AU - Shukla S

AD - Department of Pathology, Jawaharlal Nehru Medical College, Datta Meghe Institute

of Higher Education and Research, Wardha, IND.

FAU - Acharya, Sourya

AU - Acharya S

AD - Department of Medicine, Jawaharlal Nehru Medical College, Datta Meghe Institute

of Higher Education and Research, Wardha, IND.

LA - eng

PT - Case Reports

PT - Journal Article

DEP - 20240705

PL - United States

TA - Cureus

JT - Cureus

JID - 101596737

PMC - PMC11298322

OTO - NOTNLM

OT - alk-1

OT - imt

OT - pleural

OT - pseudotumor

OT - pulmonary neoplasm

COIS- Human subjects: All authors have confirmed that this study did not involve human

participants or tissue. Conflicts of interest: In compliance with the ICMJE

uniform disclosure form, all authors declare the following: Payment/services

info: All authors have declared that no financial support was received from any

organization for the submitted work. Financial relationships: All authors have

declared that they have no financial relationships at present or within the

previous three years with any organizations that might have an interest in the

submitted work. Other relationships: All authors have declared that there are no

other relationships or activities that could appear to have influenced the

submitted work.

EDAT- 2024/08/06 06:42

MHDA- 2024/08/06 06:43

PMCR- 2024/07/05

CRDT- 2024/08/06 04:23

PHST- 2024/06/08 00:00 [received]

PHST- 2024/07/04 00:00 [accepted]

PHST- 2024/08/06 06:43 [medline]

PHST- 2024/08/06 06:42 [pubmed]

PHST- 2024/08/06 04:23 [entrez]

PHST- 2024/07/05 00:00 [pmc-release]

AID - 10.7759/cureus.63892 [doi]

PST - epublish

SO - Cureus. 2024 Jul 5;16(7):e63892. doi: 10.7759/cureus.63892. eCollection 2024 Jul.

PMID- 39093163

OWN - NLM

STAT- MEDLINE

DCOM- 20240802

LR - 20240802

IS - 0717-6163 (Electronic)

IS - 0034-9887 (Linking)

VI - 151

IP - 9

DP - 2023 Sep

TI - [Hepatic Inflammatory Pseudotumor Mimicking Cholangiocarcinoma: A Rare Case and

Literature Review].

PG - 1255-1259

LID - S0034-98872023000901255 [pii]

LID - 10.4067/s0034-98872023000901255 [doi]

AB - We report the case of a 49-year-old man who attended the emergency department for

a two-month history of compromised general condition, weight loss, abdominal

pain, fever, and elevated inflammatory parameters. An imaging study demonstrates

a bulky liver tumor associated with dilation of the bile duct and retroperitoneal

adenopathies (hepatic hilum, intermediate, and right lumbar groups). These

findings raise intrahepatic cholangiocarcinoma within the differential diagnoses,

reason why segmental hepatectomy and regional lymphadenectomy were performed.

Histopathology and immunochemistry revealed a lymphoplasmacytic inflammatory

process with IgG4-positive plasma cells compatible with IgG4-associated disease.

After the resection, expectant management was decided, with the patient evolving

favorably, asymptomatic, and without signs of recurrence. We present a case and a

brief literature review of an hepatic inflammatory pseudotumor, a rare entity

with a benign behavior.

FAU - Schiappacasse F, Giancarlo

AU - Schiappacasse F G

AD - Hospital Militar de Santiago, Facultad de Medicina, Universidad de Santiago de

Chile, Santiago, Chile.

FAU - Vallejos C, Antonio

AU - Vallejos C A

AD - Hospital Militar de Santiago, Facultad de Medicina, Universidad de Santiago de

Chile, Santiago, Chile.

FAU - Figueroa D, Paula

AU - Figueroa D P

AD - Hospital Militar de Santiago, Facultad de Medicina, Universidad de Santiago de

Chile, Santiago, Chile.

FAU - Salcedo R, Amilcar

AU - Salcedo R A

AD - Hospital Militar de Santiago, Facultad de Medicina, Universidad de Santiago de

Chile, Santiago, Chile.

FAU - Bustamante M, Caroll

AU - Bustamante M C

AD - Hospital Militar, Santiago, Chile.

FAU - Humeres A, Roberto

AU - Humeres A R

AD - Hospital Militar, Santiago, Chile.

LA - spa

PT - Case Reports

PT - English Abstract

PT - Journal Article

PT - Review

TT - Pseudotumor inflamatorio hepático: caso clínico y revisión de la literatura.

PL - Chile

TA - Rev Med Chil

JT - Revista medica de Chile

JID - 0404312

SB - IM

MH - Humans

MH - Male

MH - *Cholangiocarcinoma/pathology/diagnosis/diagnostic imaging

MH - Middle Aged

MH - Diagnosis, Differential

MH - *Bile Duct Neoplasms/pathology/diagnosis/diagnostic imaging

MH - *Granuloma, Plasma Cell/diagnosis/diagnostic imaging/pathology

MH - Liver Diseases/pathology/diagnosis

MH - Hepatectomy

MH - Bile Ducts, Intrahepatic/pathology

MH - Liver Neoplasms/pathology/diagnosis/diagnostic imaging

MH - Tomography, X-Ray Computed

EDAT- 2024/08/02 12:42

MHDA- 2024/08/02 12:43

CRDT- 2024/08/02 10:23

PHST- 2023/04/05 00:00 [received]

PHST- 2023/02/05 00:00 [accepted]

PHST- 2024/08/02 12:43 [medline]

PHST- 2024/08/02 12:42 [pubmed]

PHST- 2024/08/02 10:23 [entrez]

AID - S0034-98872023000901255 [pii]

AID - 10.4067/s0034-98872023000901255 [doi]

PST - ppublish

SO - Rev Med Chil. 2023 Sep;151(9):1255-1259. doi: 10.4067/s0034-98872023000901255.

PMID- 39061052

OWN - NLM

STAT- MEDLINE

DCOM- 20240727

LR - 20240729

IS - 1749-8090 (Electronic)

IS - 1749-8090 (Linking)

VI - 19

IP - 1

DP - 2024 Jul 26

TI - Non-intubated tracheal resection and reconstruction for a tracheal tumor in an

8-year-old child.

PG - 468

LID - 10.1186/s13019-024-02949-8 [doi]

LID - 468

AB - INTRODUCTION: It has been reported that non-intubated anesthesia can be used

successfully in adult trachea reconstruction. Herein, our center reported a case

of a child undergoing non-intubated trachea reconstruction for benign tracheal

tumors. CASE DESCRIPTION: In January 2023, it was decided to attempt tracheal

resection and reconstruction (TRR) in an 8-year-old child with an inflammatory

myofibroblastic tumor under non-intubated spontaneous breathing. After anesthesia

induction, the laryngeal mask airway (LMA) was inserted. Thereafter, a bilateral

superficial cervical plexus block was performed with 15 mL of 0.25% ropivacaine

injected into each side. The patient was induced to resume spontaneous breathing

by artificially assisted ventilation with an oxygen flow of 2 to 5 L/min and

FiO(2)=1. After tracheotomy, the oxygen flow was increased to 15 L/min to improve

the local oxygen flow to maintain the pulse oxygen saturation (SpO(2)) above 90%

under spontaneous breathing. The patient had stable spontaneous breathing after

tracheal anastomosis. The anastomosis was perfect without leakage. The LMA was

removed and oxygen was given by the nasal catheter under light sedation at post

anesthesia care unit (PACU). CONCLUSION: Tracheal reconstruction under

spontaneous breathing may be an alternative anesthesia method for upper tracheal

surgery in children.

CI - © 2024. The Author(s).

FAU - Zhang, Yaoliang

AU - Zhang Y

AD - Department of Anesthesiology, the First Affiliated Hospital of Guangzhou Medical

University, No. 151, Yanjiang Xi Road, Guangzhou, Guangdong, People's Republic of

China.

FAU - Mo, Zhongqiao

AU - Mo Z

AD - Department of Anesthesiology, the First Affiliated Hospital of Guangzhou Medical

University, No. 151, Yanjiang Xi Road, Guangzhou, Guangdong, People's Republic of

China.

FAU - Yang, Chao

AU - Yang C

AD - Department of Thoracic Surgery, The First Affiliated Hospital of Guangzhou

Medical University, No. 151, Yanjiang Xi Road, Guangzhou, Guangdong, People's

Republic of China.

AD - National Clinical Research Center for Respiratory Disease, State Key Laboratory

of Respiratory Disease, Guangzhou Institute of Respiratory Health, the First

Affiliated Hospital of Guangzhou Medical University, Guangzhou, China.

FAU - He, Jianxing

AU - He J

AD - Department of Thoracic Surgery, The First Affiliated Hospital of Guangzhou

Medical University, No. 151, Yanjiang Xi Road, Guangzhou, Guangdong, People's

Republic of China.

AD - National Clinical Research Center for Respiratory Disease, State Key Laboratory

of Respiratory Disease, Guangzhou Institute of Respiratory Health, the First

Affiliated Hospital of Guangzhou Medical University, Guangzhou, China.

FAU - Li, Shuben

AU - Li S

AD - Department of Thoracic Surgery, The First Affiliated Hospital of Guangzhou

Medical University, No. 151, Yanjiang Xi Road, Guangzhou, Guangdong, People's

Republic of China. 13500030280@163.com.

AD - National Clinical Research Center for Respiratory Disease, State Key Laboratory

of Respiratory Disease, Guangzhou Institute of Respiratory Health, the First

Affiliated Hospital of Guangzhou Medical University, Guangzhou, China.

13500030280@163.com.

FAU - Lan, Lan

AU - Lan L

AD - Department of Anesthesiology, the First Affiliated Hospital of Guangzhou Medical

University, No. 151, Yanjiang Xi Road, Guangzhou, Guangdong, People's Republic of

China. lanlan@gzhmu.edu.cn.

LA - eng

PT - Case Reports

PT - Journal Article

DEP - 20240726

PL - England

TA - J Cardiothorac Surg

JT - Journal of cardiothoracic surgery

JID - 101265113

SB - IM

MH - Humans

MH - Child

MH - *Tracheal Neoplasms/surgery

MH - *Plastic Surgery Procedures/methods

MH - *Trachea/surgery

MH - Male

MH - Laryngeal Masks

MH - Tracheotomy/methods

PMC - PMC11282805

OTO - NOTNLM

OT - Children

OT - Non-intubated

OT - Trachea resection

OT - Tracheal tumors

COIS- The authors declare no competing interests.

EDAT- 2024/07/27 10:42

MHDA- 2024/07/28 14:54

PMCR- 2024/07/26

CRDT- 2024/07/26 23:51

PHST- 2024/01/29 00:00 [received]

PHST- 2024/06/29 00:00 [accepted]

PHST- 2024/07/28 14:54 [medline]

PHST- 2024/07/27 10:42 [pubmed]

PHST- 2024/07/26 23:51 [entrez]

PHST- 2024/07/26 00:00 [pmc-release]

AID - 10.1186/s13019-024-02949-8 [pii]

AID - 2949 [pii]

AID - 10.1186/s13019-024-02949-8 [doi]

PST - epublish

SO - J Cardiothorac Surg. 2024 Jul 26;19(1):468. doi: 10.1186/s13019-024-02949-8.

PMID- 39048371

OWN - NLM

STAT- Publisher

LR - 20240724

IS - 1349-7235 (Electronic)

IS - 0918-2918 (Linking)

DP - 2024 Jul 25

TI - Lorlatinib for the Treatment of Inflammatory Myofibroblastic Tumor after

Allogeneic Hematopoietic Stem Cell Transplantation: A Case Report.

LID - 10.2169/internalmedicine.3262-23 [doi]

AB - Inflammatory myofibroblastic tumors (IMTs) are rare sarcomas composed of

myofibroblastic and fibroblastic cells, accompanied by inflammatory cell

infiltration. Many IMTs exhibit clonal rearrangement of anaplastic lymphoma

kinase (ALK). We herein report a 56-year-old woman with uterine IMT harboring a

thrombospondin-1::ALK fusion that developed after allogeneic hematopoietic stem

cell transplantation (allo-HSCT). Laboratory data before systemic therapy

indicated increased interleukin-6 and severe leukocytosis. The patient was

treated with lorlatinib; however, the response duration was approximately two

months. Similar case reports need to be compiled and evaluated to elucidate the

efficacy of lorlatinib in post-allo-HSCT IMT with ALK rearrangement.

FAU - Yagi, Toshinari

AU - Yagi T

AD - Department of Outpatient Chemotherapy, Osaka International Cancer Institute,

Japan.

FAU - Kukita, Yoji

AU - Kukita Y

AD - Laboratory of Genomic Pathology, Osaka International Cancer Institute, Japan.

FAU - Matsuoka, Haruki

AU - Matsuoka H

AD - Department of Orthopedic Surgery, Osaka International Cancer Institute, Japan.

FAU - Wakamatsu, Toru

AU - Wakamatsu T

AD - Department of Orthopedic Surgery, Osaka International Cancer Institute, Japan.

FAU - Tamiya, Hironari

AU - Tamiya H

AD - Department of Orthopedic Surgery, Osaka International Cancer Institute, Japan.

FAU - Watanabe, Makiyo

AU - Watanabe M

AD - Department of Orthopedic Surgery, Osaka International Cancer Institute, Japan.

FAU - Kakunaga, Shigeki

AU - Kakunaga S

AD - Department of Orthopedic Surgery, Osaka International Cancer Institute, Japan.

FAU - Takenaka, Satoshi

AU - Takenaka S

AD - Department of Orthopedic Surgery, Osaka International Cancer Institute, Japan.

FAU - Kubo, Chiaki

AU - Kubo C

AD - Department of Pathology, Osaka International Cancer Institute, Japan.

FAU - Hashii, Yoshiko

AU - Hashii Y

AD - Department of Pediatrics, Osaka International Cancer Institute, Japan.

FAU - Nakanishi, Katsuyuki

AU - Nakanishi K

AD - Department of Diagnostic and Interventional Radiology, Osaka International Cancer

Institute, Japan.

LA - eng

PT - Journal Article

DEP - 20240725

PL - Japan

TA - Intern Med

JT - Internal medicine (Tokyo, Japan)

JID - 9204241

SB - IM

OTO - NOTNLM

OT - Allogeneic hematopoietic stem cell transplantation

OT - Inflammatory myofibroblastic tumor

OT - Interleukin-6

OT - Lorlatinib

OT - THBS1::ALK

EDAT- 2024/07/26 12:35

MHDA- 2024/07/26 12:35

CRDT- 2024/07/24 21:53

PHST- 2024/07/26 12:35 [medline]

PHST- 2024/07/26 12:35 [pubmed]

PHST- 2024/07/24 21:53 [entrez]

AID - 10.2169/internalmedicine.3262-23 [doi]

PST - aheadofprint

SO - Intern Med. 2024 Jul 25. doi: 10.2169/internalmedicine.3262-23.

PMID- 38994118

OWN - NLM

STAT- PubMed-not-MEDLINE

LR - 20240714

IS - 2405-8440 (Print)

IS - 2405-8440 (Electronic)

IS - 2405-8440 (Linking)

VI - 10

IP - 12

DP - 2024 Jun 30

TI - Epstein-barr virus (EBV)-positive inflammatory pseudotumor-like follicular

dendritic cell sarcoma (IPT-like FDCS) presenting as thrombocytopenia: A case

report and literature review.

PG - e32997

LID - 10.1016/j.heliyon.2024.e32997 [doi]

LID - e32997

AB - BACKGROUND: Follicular dendritic cell sarcoma (FDCS) represents an exceedingly

rare malignant neoplasm. Inflammatory pseudotumor-like follicular dendritic cell

sarcoma (IPT-like FDCS) is recognized as a variant manifestation of FDCS. The

clinical incidence of this particular disease is remarkably low, resulting in the

absence of established standardized clinical protocols for its management and

treatment. METHODS: Presented here is a case of primary Epstein-Barr virus

(EBV)-positive splenic IPT-like FDCS, noteworthy for manifesting thrombocytopenia

as its initial symptom. Our study analyzed the clinicopathologic characteristics

of this case and 29 previously reported cases identified in the literature. Also,

we conducted a comprehensive review of pertinent literature. RESULTS: We

administered splenectomy to this patient and verified the diagnosis of

EBV-positive IPT-like FDCS through immunohistochemical examination.

Postoperatively, the patient underwent a one-year follow-up period, demonstrating

no signs of recurrence. Analyzing a total of 30 cases revealed that this disease

is more prevalent in female patients (F:M = 1.14:1), with a median age of 62

years. Fifteen patients were asymptomatic, and nine patients presented with

abdominal discomfort or pain. All patients underwent surgical treatment. Among

the cases, histopathological and immunohistochemical information was unavailable

for five; however, in the remaining 25 cases, histopathology revealed a distinct

inflammatory cell infiltration and spindle tumor cells arranged in sheets or

fascicles. These tumor cells had vesicular chromatin and distinct nucleoli and

they expressed conventional FDC markers. In situ hybridization analysis of

Epstein-Barr virus-encoded small RNA (EBER) showed that all 30 cases were

EBV-positive. Follow-up information showed that no patients relapsed and one

(3.8 %) patient died. CONCLUSION: The clinical diagnosis of EBV-positive IPT-like

FDCS poses considerable challenges, necessitating a conclusive diagnosis through

pathological immunohistochemical examination. EBER in situ hybridization holds

significance for the definitive diagnosis of the disease. We advocate for

splenectomy as the treatment of choice for limited splenic IPT-like FDCS.

CI - © 2024 The Authors.

FAU - Jin, Jiawei

AU - Jin J

AD - Hospital of Soochow University, China.

FAU - Zhu, Xiaolong

AU - Zhu X

AD - Hospital of Soochow University, China.

FAU - Wan, Yi

AU - Wan Y

AD - Hospital of Soochow University, China.

FAU - Shi, Yang

AU - Shi Y

AD - Hospital of Soochow University, China.

LA - eng

PT - Case Reports

PT - Journal Article

DEP - 20240615

PL - England

TA - Heliyon

JT - Heliyon

JID - 101672560

PMC - PMC11238001

OTO - NOTNLM

OT - Case report

OT - Follicular dendritic cell sarcoma

OT - Spleen

OT - Surgery

COIS- The authors declare that they have no known competing financial interests or

personal relationships that could have appeared to influence the work reported in

this paper.

EDAT- 2024/07/12 06:42

MHDA- 2024/07/12 06:43

PMCR- 2024/06/15

CRDT- 2024/07/12 04:47

PHST- 2024/02/02 00:00 [received]

PHST- 2024/06/11 00:00 [revised]

PHST- 2024/06/12 00:00 [accepted]

PHST- 2024/07/12 06:43 [medline]

PHST- 2024/07/12 06:42 [pubmed]

PHST- 2024/07/12 04:47 [entrez]

PHST- 2024/06/15 00:00 [pmc-release]

AID - S2405-8440(24)09028-5 [pii]

AID - e32997 [pii]

AID - 10.1016/j.heliyon.2024.e32997 [doi]

PST - epublish

SO - Heliyon. 2024 Jun 15;10(12):e32997. doi: 10.1016/j.heliyon.2024.e32997.

eCollection 2024 Jun 30.

PMID- 38989355

OWN - NLM

STAT- PubMed-not-MEDLINE

LR - 20240712

IS - 2168-8184 (Print)

IS - 2168-8184 (Electronic)

IS - 2168-8184 (Linking)

VI - 16

IP - 6

DP - 2024 Jun

TI - Pulmonary Calcifying Fibrous Tumor in a Pediatric Patient: A Case Report.

PG - e62053

LID - 10.7759/cureus.62053 [doi]

LID - e62053

AB - A calcifying fibrous tumor (CFT), also known as calcifying fibrous pseudotumor,

is an uncommon non-cancerous neoplasm usually located in the gastrointestinal

tract. Its location in the lung is extremely rare, and only a few case reports

have been published. This case report describes our diagnostic approach in a

9-year-old male patient with an incidental pulmonary mass. The mass was initially

misdiagnosed, requiring multiple imaging tests and interventions to obtain the

definitive diagnosis of pulmonary CFT. This paper aims to contribute to the

limited information available on pulmonary CFT by presenting detailed findings

from computed tomography and magnetic resonance imaging.

CI - Copyright © 2024, Herrera Ortiz et al.

FAU - Herrera Ortiz, Andrés Felipe

AU - Herrera Ortiz AF

AD - Department of Radiology, Fundación Santa Fe de Bogotá, Bogotá D.C., COL.

AD - Department of Radiology, Universidad El Bosque, Bogotá D.C., COL.

FAU - Del Castillo, Valeria

AU - Del Castillo V

AD - Department of Radiology, Fundación Santa Fe de Bogotá, Bogotá D.C., COL.

AD - Department of Radiology, Universidad El Bosque, Bogotá D.C., COL.

FAU - Duarte, José N

AU - Duarte JN

AD - Department of Radiology, Fundación Santa Fe de Bogotá, Bogotá D.C., COL.

AD - Department of Radiology, Universidad El Bosque, Bogotá D.C., COL.

FAU - Gutiérrez, María J

AU - Gutiérrez MJ

AD - Department of Medicine and Health Sciences, Universidad del Rosario, Bogotá D.C.,

COL.

FAU - Noguera, Valeria

AU - Noguera V

AD - Department of Radiology, Fundación Santa Fe de Bogotá, Bogotá D.C., COL.

AD - Department of Radiology, Universidad El Bosque, Bogotá D.C., COL.

FAU - Martínez de Los Ríos, Daniel A

AU - Martínez de Los Ríos DA

AD - Department of Medicine and Health Sciences, Universidad El Bosque, Bogotá D.C.,

COL.

FAU - Maldonado Acevedo, Sandra P

AU - Maldonado Acevedo SP

AD - Department of Radiology, Fundación Santa Fe de Bogotá, Bogotá D.C., COL.

AD - Department of Radiology, Universidad El Bosque, Bogotá D.C., COL.

FAU - Torres, Jhon L

AU - Torres JL

AD - Department of Radiology, Fundación Santa Fe de Bogotá, Bogotá D.C., COL.

FAU - Pinzón, Bibiana

AU - Pinzón B

AD - Department of Radiology, Fundación Santa Fe de Bogotá, Bogotá D.C., COL.

AD - Department of Radiology, Universidad El Bosque, Bogotá D.C., COL.

FAU - Moreno, Angela

AU - Moreno A

AD - Department of Radiology, Fundación Santa Fe de Bogotá, Bogotá D.C., COL.

AD - Department of Radiology, Universidad El Bosque, Bogotá D.C., COL.

FAU - Quiroz Alfaro, Alejandro J

AU - Quiroz Alfaro AJ

AD - Department of Internal Medicine, North Mississippi Medical Center, Tupelo, USA.

AD - Department of Medicine and Health Sciences, Universidad del Rosario, Bogotá D.C.,

COL.

LA - eng

PT - Case Reports

PT - Journal Article

DEP - 20240610

PL - United States

TA - Cureus

JT - Cureus

JID - 101596737

PMC - PMC11234803

OTO - NOTNLM

OT - calcifying fibrous tumor

OT - high-resolution computed tomography

OT - inflammatory myofibroblastic tumor

OT - lung hamartoma

OT - lung neoplasm

OT - magnetic resonance imaging

OT - pediatrics

OT - psammomatous calcification

OT - pulmonary sequestration

OT - unusual diagnosis

COIS- Human subjects: Consent was obtained or waived by all participants in this study.

Conflicts of interest: In compliance with the ICMJE uniform disclosure form, all

authors declare the following: Payment/services info: All authors have declared

that no financial support was received from any organization for the submitted

work. Financial relationships: All authors have declared that they have no

financial relationships at present or within the previous three years with any

organizations that might have an interest in the submitted work. Other

relationships: All authors have declared that there are no other relationships or

activities that could appear to have influenced the submitted work.

EDAT- 2024/07/11 06:42

MHDA- 2024/07/11 06:43

PMCR- 2024/06/10

CRDT- 2024/07/11 04:38

PHST- 2024/06/10 00:00 [accepted]

PHST- 2024/07/11 06:43 [medline]

PHST- 2024/07/11 06:42 [pubmed]

PHST- 2024/07/11 04:38 [entrez]

PHST- 2024/06/10 00:00 [pmc-release]

AID - 10.7759/cureus.62053 [doi]

PST - epublish

SO - Cureus. 2024 Jun 10;16(6):e62053. doi: 10.7759/cureus.62053. eCollection 2024

Jun.

PMID- 38988714

OWN - NLM

STAT- PubMed-not-MEDLINE

LR - 20240712

IS - 2326-3253 (Print)

IS - 2326-3253 (Electronic)

IS - 2326-3253 (Linking)

VI - 11

IP - 7

DP - 2024 Jul

TI - A Diagnosis of Gastric Inflammatory Myofibroblast Tumor: A Challenge Like No

Other!

PG - e01416

LID - 10.14309/crj.0000000000001416 [doi]

LID - e01416

AB - Inflammatory myofibroblastic tumors (IMTs) are mesenchymal tumors of intermediate

malignant potential. Gastric IMTs are rare and commonly affect young adults. They

are typically confused with gastrointestinal stromal tumors, inflammatory fibroid

polyps, and leiomyosarcomas. The etiology of IMTs remains unclear, but is

theorized to be due to hyperinflammatory response to chronic infections. We

present a middle-aged woman found to have a gastric mass positive for

Helicobacter pylori, underwent multiple endoscopies with endoscopic ultrasound,

and a definitive diagnosis of gastric IMT was only made after a partial

gastrectomy with immunohistochemistry negative for CD-117, S-100, ALK-1, and

positive for vimentin and SMA.

CI - © 2024 The Author(s). Published by Wolters Kluwer Health, Inc. on behalf of The

American College of Gastroenterology.

FAU - Chukkalore, Divya

AU - Chukkalore D

AD - Department of Internal Medicine, Zucker School of Medicine at Hofstra/Northwell

at Staten Island University Hospital, Staten Island, NY.

FAU - Loeffler, Jeffrey

AU - Loeffler J

AD - Department of Internal Medicine, Zucker School of Medicine at Hofstra/Northwell

at Staten Island University Hospital, Staten Island, NY.

FAU - Rabah, Hussein

AU - Rabah H

AD - Department of Internal Medicine, Zucker School of Medicine at Hofstra/Northwell

at Staten Island University Hospital, Staten Island, NY.

FAU - Amarnath, Shivantha

AU - Amarnath S

AD - Department of Gastroenterology & Hepatology, Zucker School of Medicine at

Hofstra/Northwell at Staten Island University Hospital, Staten Island, NY.

FAU - Al Moussawi, Hassan

AU - Al Moussawi H

AD - Department of Gastroenterology & Hepatology, Zucker School of Medicine at

Hofstra/Northwell at Staten Island University Hospital, Staten Island, NY.

FAU - Deeb, Liliane

AU - Deeb L

AD - Department of Gastroenterology & Hepatology, Zucker School of Medicine at

Hofstra/Northwell at Staten Island University Hospital, Staten Island, NY.

LA - eng

PT - Case Reports

PT - Journal Article

DEP - 20240710

PL - United States

TA - ACG Case Rep J

JT - ACG case reports journal

JID - 101638398

PMC - PMC11236408

OTO - NOTNLM

OT - endoscopic ultrasound

OT - endoscopy

OT - gastric tumor

OT - pathology

OT - tumor

EDAT- 2024/07/11 06:41

MHDA- 2024/07/11 06:42

PMCR- 2024/07/10

CRDT- 2024/07/11 04:28

PHST- 2024/01/29 00:00 [received]

PHST- 2024/05/31 00:00 [accepted]

PHST- 2024/07/11 06:42 [medline]

PHST- 2024/07/11 06:41 [pubmed]

PHST- 2024/07/11 04:28 [entrez]

PHST- 2024/07/10 00:00 [pmc-release]

AID - ACGCR-24-0097 [pii]

AID - 10.14309/crj.0000000000001416 [doi]

PST - epublish

SO - ACG Case Rep J. 2024 Jul 10;11(7):e01416. doi: 10.14309/crj.0000000000001416.

eCollection 2024 Jul.

PMID- 38979776

OWN - NLM

STAT- MEDLINE

DCOM- 20241025

LR - 20250109

IS - 1532-0979 (Electronic)

IS - 0147-5185 (Linking)

VI - 48

IP - 11

DP - 2024 Nov 1

TI - ALK -rearranged Mesenchymal Neoplasms With Prominent Foamy/Pseudolipogenic Cell

Morphology : Expanding the Phenotypic Spectrum of ALK Fusion Neoplasms and Report

of Novel Fusion Partners.

PG - 1455-1463

LID - 10.1097/PAS.0000000000002283 [doi]

AB - The category of ALK -rearranged mesenchymal neoplasms has been evolving rapidly,

with reports of morphologically diverse lesions of cutaneous, soft tissue, and

visceral origin. While some of these represent morphologically defined entities

harboring recurrent ALK fusions (inflammatory myofibroblastic tumor and

epithelioid fibrous histiocytoma), others are unclassified by morphology with

variable overlap with the tyrosine kinase family of neoplasia and their

underlying ALK fusions cannot be suspected based on morphology. We herein report

3 cases that expand the anatomic, morphologic, and genotypic spectrum of ALK

-rearranged unclassified neoplasms. Patients were all adults aged 46 to 69

(median: 63) who presented with a mass located in the gingiva, subcutis of the

back, and submucosal posterior pharyngeal wall. The tumor size ranged from 1 to

2.7 cm (median: 1.6). Conservative surgery was the treatment in all patients.

Follow-up was available for one patient who remained disease-free at 14 months.

Histologically, all tumors displayed large polygonal cells with foamy to granular

and lipogenic-like microvacuolated copious cytoplasm and medium-sized round

nuclei with 1 or 2 prominent nucleoli. Mitoses and necrosis were not seen. The

initial diagnostic impression was PEComa, inflammatory rhabdomyoblastic tumor and

unclassified pseudolipogenic neoplasm. Strong cytoplasmic ALK was detected by

immunohistochemistry in all cases. Other positive markers include Cathepsin K

(2/2), desmin (1/3), focal MyoD1 (1/1), focal SMA (1/3), and focal EMA (1/2).

Targeted RNA sequencing revealed ALK fusions with exon 20 (2 cases) and exon 19

(one case) of ALK fused to RND3 (exon 3), SQSTM1 (exon 6), and desmin (intron 6).

Methylation profiling in the desmin-fused case (initially diagnosed as

inflammatory rhabdomyoblastic tumor) revealed an inflammatory myofibroblastic

tumor match with a low confidence score of 0.5 and a flat copy number variation

(CNV) profile. No NF1 mutation was detected in this case, altogether excluding an

inflammatory rhabdomyoblastic tumor. Our study highlights and expands the

morphologic and anatomic diversity of ALK- fused neoplasms and documents novel

fusion partners ( RND3 and desmin).

CI - Copyright © 2024 Wolters Kluwer Health, Inc. All rights reserved.

FAU - Agaimy, Abbas

AU - Agaimy A

AD - Institute of Pathology, Erlangen University Hospital, Friedrich Alexander

University of Erlangen-Nuremberg.

AD - Comprehensive Cancer Center, European Metropolitan Area Erlangen-Nuremberg (CCC

ER-EMN), Erlangen, Germany.

FAU - Stoehr, Robert

AU - Stoehr R

AD - Institute of Pathology, Erlangen University Hospital, Friedrich Alexander

University of Erlangen-Nuremberg.

AD - Comprehensive Cancer Center, European Metropolitan Area Erlangen-Nuremberg (CCC

ER-EMN), Erlangen, Germany.

FAU - Fisher, Cyril

AU - Fisher C

AD - Department of Cellular Pathology, University Hospitals Birmingham, Birmingham,

UK.

FAU - Chrisinger, John S A

AU - Chrisinger JSA

AD - Department of Pathology and Immunology, Washington University School of Medicine,

St. Louis, MO.

FAU - Demicco, Elizabeth G

AU - Demicco EG

AD - Department of Pathology and Laboratory Medicine, Mount Sinai Hospital and

Laboratory Medicine and Pathobiology, University of Toronto, Canada.

FAU - Tögel, Lars

AU - Tögel L

AD - Institute of Pathology, Erlangen University Hospital, Friedrich Alexander

University of Erlangen-Nuremberg.

AD - Comprehensive Cancer Center, European Metropolitan Area Erlangen-Nuremberg (CCC

ER-EMN), Erlangen, Germany.

FAU - Michal, Michal

AU - Michal M

AD - Department of Pathology, Faculty of Medicine, Charles University, Plzen, Czech

Republic.

AD - Bioptical Laboratory, Ltd., Plzen, Czech Republic.

FAU - Michal, Michael

AU - Michal M

AD - Department of Pathology, Faculty of Medicine, Charles University, Plzen, Czech

Republic.

AD - Bioptical Laboratory, Ltd., Plzen, Czech Republic.

LA - eng

PT - Case Reports

PT - Journal Article

DEP - 20240709

PL - United States

TA - Am J Surg Pathol

JT - The American journal of surgical pathology

JID - 7707904

RN - EC 2.7.10.1 (Anaplastic Lymphoma Kinase)

RN - EC 2.7.10.1 (ALK protein, human)

RN - 0 (Biomarkers, Tumor)

RN - EC 2.7.10.1 (Receptor Protein-Tyrosine Kinases)

SB - IM

MH - Humans

MH - *Anaplastic Lymphoma Kinase/genetics

MH - Middle Aged

MH - Male

MH - Female

MH - Aged

MH - *Gene Rearrangement

MH - *Phenotype

MH - *Biomarkers, Tumor/genetics

MH - Foam Cells/pathology/enzymology

MH - Immunohistochemistry

MH - Receptor Protein-Tyrosine Kinases/genetics

MH - Genetic Predisposition to Disease

COIS- Conflicts of Interest and Source of Funding: The authors have disclosed that they

have no significant relationships with, or financial interest in, any commercial

companies pertaining to this article.

EDAT- 2024/07/09 12:42

MHDA- 2024/10/25 12:24

CRDT- 2024/07/09 07:13

PHST- 2024/10/25 12:24 [medline]

PHST- 2024/07/09 12:42 [pubmed]

PHST- 2024/07/09 07:13 [entrez]

AID - 00000478-202411000-00013 [pii]

AID - 10.1097/PAS.0000000000002283 [doi]

PST - ppublish

SO - Am J Surg Pathol. 2024 Nov 1;48(11):1455-1463. doi: 10.1097/PAS.0000000000002283.

Epub 2024 Jul 9.

PMID- 38966066

OWN - NLM

STAT- PubMed-not-MEDLINE

LR - 20240706

IS - 2234-943X (Print)

IS - 2234-943X (Electronic)

IS - 2234-943X (Linking)

VI - 14

DP - 2024

TI - Case report: Splenic inflammatory pseudotumor-like follicular dendritic cell

sarcoma (IPT-like FDCS): a trial of immunotherapy and review of the literature.

PG - 1360726

LID - 10.3389/fonc.2024.1360726 [doi]

LID - 1360726

AB - Inflammatory pseudotumor-like follicular dendritic cell sarcoma (IPT-like FDCS)

is a rare malignancy with fewer than 150 cases in the literature. IPT-like FDCS

follows an indolent course with most cases definitively managed with surgical

resection. We present a case of IPT-like FDCS with multiple recurrences with a

trial of immunotherapy. The patient initially presented with splenic involvement

requiring splenectomy, subsequently recurring in the liver requiring hepatic

resections. Afterwards, there was recurrence with pelvic/small bowel involvement

for which treatment was trialed with ipilimumab and nivolumab. The patient

progressed despite dual immune checkpoint inhibitor therapy requiring a small

bowel resection. To date, this is the first case of immunotherapy use in IPT-like

FDCS. Therefore, more evidence is needed to support additional treatments in

recurrent IPT-like FDCS after resection.

CI - Copyright © 2024 Resnick, Monroe, Siddiqi and Tam.

FAU - Resnick, K A

AU - Resnick KA

AD - Department of Medicine, University of Southern California (USC)/Los Angeles

General, Los Angeles, CA, United States.

FAU - Monroe, C

AU - Monroe C

AD - Department of Hematopathology, Keck Medicine of USC, Los Angeles, CA, United

States.

FAU - Siddiqi, I

AU - Siddiqi I

AD - Department of Hematopathology, Keck Medicine of USC, Los Angeles, CA, United

States.

FAU - Tam, E

AU - Tam E

AD - Department of Bone Marrow Transplant, USC Norris Comprehensive Cancer Hospital,

Los Angeles, CA, United States.

LA - eng

PT - Case Reports

PT - Journal Article

DEP - 20240620

PL - Switzerland

TA - Front Oncol

JT - Frontiers in oncology

JID - 101568867

PMC - PMC11222608

OTO - NOTNLM

OT - IPT-like FDCS

OT - case report

OT - immunotherapy

OT - inflammatory pseudotumor-like follicular dendritic cell sarcoma

OT - recurrence

COIS- The authors declare that the research was conducted in the absence of any

commercial or financial relationships that could be construed as a potential

conflict of interest.

EDAT- 2024/07/05 06:42

MHDA- 2024/07/05 06:43

PMCR- 2024/01/01

CRDT- 2024/07/05 04:04

PHST- 2023/12/23 00:00 [received]

PHST- 2024/05/20 00:00 [accepted]

PHST- 2024/07/05 06:43 [medline]

PHST- 2024/07/05 06:42 [pubmed]

PHST- 2024/07/05 04:04 [entrez]

PHST- 2024/01/01 00:00 [pmc-release]

AID - 10.3389/fonc.2024.1360726 [doi]

PST - epublish

SO - Front Oncol. 2024 Jun 20;14:1360726. doi: 10.3389/fonc.2024.1360726. eCollection

2024.

PMID- 38948020

OWN - NLM

STAT- MEDLINE

DCOM- 20240701

LR - 20240702

IS - 1555-3906 (Electronic)

IS - 0965-0407 (Print)

IS - 0965-0407 (Linking)

VI - 32

IP - 7

DP - 2024

TI - Inflammatory myofibroblastic tumor from molecular diagnostics to current

treatment.

PG - 1141-1162

LID - 10.32604/or.2024.050350 [doi]

AB - Inflammatory myofibroblastic tumor (IMT) is a rare neoplasm with intermediate

malignancy characterized by a propensity for recurrence but a low metastatic

rate. Diagnostic challenges arise from the diverse pathological presentation,

variable symptomatology, and lack of different imaging features. However, IMT is

identified by the fusion of the anaplastic lymphoma kinase (ALK) gene, which is

present in approximately 70% of cases, with various fusion partners, including

ran-binding protein 2 (RANBP2), which allows confirmation of the diagnosis. While

surgery is the preferred approach for localized tumors, the optimal long-term

treatment for advanced or metastatic disease is difficult to define. Targeted

therapies are crucial for achieving sustained response to treatment within the

context of genetic alteration in IMT. Crizotinib, an ALK tyrosine kinase

inhibitor (TKI), was officially approved by the US Food and Drug Administration

(FDA) in 2020 to treat IMT with ALK rearrangement. However, most patients face

resistance and disease progression, requiring consideration of sequential

treatments. Combining radiotherapy with targeted therapy appears to be beneficial

in this indication. Early promising results have also been achieved with

immunotherapy, indicating potential for combined therapy approaches. However,

defined recommendations are still lacking. This review analyzes the available

research on IMT, including genetic disorders and their impact on the course of

the disease, data on the latest targeted therapy regimens and the possibility of

developing immunotherapy in this indication, as well as summarizing general

knowledge about prognostic and predictive factors, also in terms of resistance to

systemic therapy.

CI - © 2024 Chmiel et al.

FAU - Chmiel, Paulina

AU - Chmiel P

AD - Department of Soft Tissue/Bone Sarcoma and Melanoma, Maria Sklodowska-Curie

National Research Institute of Oncology, Warsaw, 02-781, Poland.

AD - Faculty of Medicine, Medical University of Warsaw, Warsaw, 02-091, Poland.

FAU - SłOWIKOWSKA, Aleksandra

AU - SłOWIKOWSKA A

AD - Department of Soft Tissue/Bone Sarcoma and Melanoma, Maria Sklodowska-Curie

National Research Institute of Oncology, Warsaw, 02-781, Poland.

AD - Faculty of Medicine, Medical University of Warsaw, Warsaw, 02-091, Poland.

FAU - Banaszek, Łukasz

AU - Banaszek Ł

AD - Department of Soft Tissue/Bone Sarcoma and Melanoma, Maria Sklodowska-Curie

National Research Institute of Oncology, Warsaw, 02-781, Poland.

AD - Faculty of Medicine, Medical University of Warsaw, Warsaw, 02-091, Poland.

FAU - Szumera-CIEćKIEWICZ, Anna

AU - Szumera-CIEćKIEWICZ A

AD - Department of Pathology, Maria Sklodowska Curie National Research Institute of

Oncology, Warsaw, 02-781, Poland.

FAU - Szostakowski, BARTłOMIEJ

AU - Szostakowski B

AD - Department of Soft Tissue/Bone Sarcoma and Melanoma, Maria Sklodowska-Curie

National Research Institute of Oncology, Warsaw, 02-781, Poland.

FAU - SPAłEK, Mateusz J

AU - SPAłEK MJ

AD - Department of Soft Tissue/Bone Sarcoma and Melanoma, Maria Sklodowska-Curie

National Research Institute of Oncology, Warsaw, 02-781, Poland.

AD - Department of Radiotherapy, Maria Sklodowska-Curie National Research Institute of

Oncology, Warsaw, 02-781, Poland.

FAU - Świtaj, Tomasz

AU - Świtaj T

AD - Department of Soft Tissue/Bone Sarcoma and Melanoma, Maria Sklodowska-Curie

National Research Institute of Oncology, Warsaw, 02-781, Poland.

FAU - Rutkowski, Piotr

AU - Rutkowski P

AD - Department of Soft Tissue/Bone Sarcoma and Melanoma, Maria Sklodowska-Curie

National Research Institute of Oncology, Warsaw, 02-781, Poland.

FAU - Czarnecka, Anna M

AU - Czarnecka AM

AD - Department of Soft Tissue/Bone Sarcoma and Melanoma, Maria Sklodowska-Curie

National Research Institute of Oncology, Warsaw, 02-781, Poland.

LA - eng

PT - Journal Article

PT - Review

DEP - 20240620

PL - United States

TA - Oncol Res

JT - Oncology research

JID - 9208097

RN - EC 2.7.10.1 (Anaplastic Lymphoma Kinase)

RN - 0 (Protein Kinase Inhibitors)

RN - EC 2.7.10.1 (ALK protein, human)

SB - IM

MH - Humans

MH - *Neoplasms, Muscle Tissue/genetics/diagnosis/pathology/therapy/drug therapy

MH - Anaplastic Lymphoma Kinase/genetics

MH - Molecular Targeted Therapy

MH - Protein Kinase Inhibitors/therapeutic use

PMC - PMC11209743

OTO - NOTNLM

OT - Anaplastic lymphoma kinase (ALK)

OT - Epithelioid inflammatory myofibroblastic sarcoma

OT - Inflammatory myofibroblastic tumor (IMT)

OT - Tyrosine kinase inhibitors (TKI)

COIS- The authors declare that they have no conflicts of interest to report regarding

the present study.

EDAT- 2024/07/01 06:41

MHDA- 2024/07/01 12:43

PMCR- 2024/06/20

CRDT- 2024/07/01 05:50

PHST- 2024/02/02 00:00 [received]

PHST- 2024/04/09 00:00 [accepted]

PHST- 2024/07/01 12:43 [medline]

PHST- 2024/07/01 06:41 [pubmed]

PHST- 2024/07/01 05:50 [entrez]

PHST- 2024/06/20 00:00 [pmc-release]

AID - 50350 [pii]

AID - 10.32604/or.2024.050350 [doi]

PST - epublish

SO - Oncol Res. 2024 Jun 20;32(7):1141-1162. doi: 10.32604/or.2024.050350. eCollection

2024.

PMID- 38918263

OWN - NLM

STAT- MEDLINE

DCOM- 20241105

LR - 20241205

IS - 1433-0350 (Electronic)

IS - 0256-7040 (Linking)

VI - 40

IP - 11

DP - 2024 Nov

TI - Pediatric skull inflammatory myofibroblastic tumor: a rare case report and

literature review.

PG - 3829-3835

LID - 10.1007/s00381-024-06512-7 [doi]

AB - Inflammatory myofibroblastic tumors (IMTs) represent rare neoplasms, particularly

infrequent in the pediatric skull. We present a novel case of a newborn male with

a 5 cm right temporal mass and discuss current diagnostic and treatment options

for IMTs. A multidisciplinary effort to surgically remove the lesion was

successful, and the patient's skull defect healed without neurological deficits.

The etiology of IMTs remains elusive, with proposed associations with chromosomal

mutations in the anaplastic lymphoma kinase (ALK) gene. Surgical excision remains

the primary treatment for IMTs. Promising pharmacological treatments, like

Crizotinib, warrant further research into understanding potential alternatives in

IMT management.

CI - © 2024. The Author(s), under exclusive licence to Springer-Verlag GmbH Germany,

part of Springer Nature.

FAU - Khurana, Eeshan

AU - Khurana E

AD - NJ Craniofacial Center, Morristown, NJ, 07960, USA. eekmd10@gmail.com.

FAU - Mody, Shaan

AU - Mody S

AD - NJ Craniofacial Center, Morristown, NJ, 07960, USA.

FAU - Shah, Tanisha

AU - Shah T

AD - NJ Craniofacial Center, Morristown, NJ, 07960, USA.

FAU - Bouffard, John-Paul

AU - Bouffard JP

AD - Department of Pathology, Atlantic Health System, Summit, NJ, 07960, USA.

FAU - Pedemonte, Maria

AU - Pedemonte M

AD - Department of Pathology, Atlantic Health System, Summit, NJ, 07960, USA.

FAU - Holover, Gianna

AU - Holover G

AD - NJ Craniofacial Center, Morristown, NJ, 07960, USA.

FAU - Lee, Jessica S

AU - Lee JS

AD - NJ Craniofacial Center, Morristown, NJ, 07960, USA.

FAU - Jacob, Gregg

AU - Jacob G

AD - NJ Craniofacial Center, Morristown, NJ, 07960, USA.

FAU - Scheid, Sara

AU - Scheid S

AD - NJ Craniofacial Center, Morristown, NJ, 07960, USA.

FAU - Morin, Robert

AU - Morin R

AD - NJ Craniofacial Center, Morristown, NJ, 07960, USA.

FAU - Mazzola, Catherine

AU - Mazzola C

AD - NJ Craniofacial Center, Morristown, NJ, 07960, USA.

LA - eng

PT - Case Reports

PT - Journal Article

PT - Review

DEP - 20240625

PL - Germany

TA - Childs Nerv Syst

JT - Child's nervous system : ChNS : official journal of the International Society for

Pediatric Neurosurgery

JID - 8503227

SB - IM

MH - Humans

MH - Male

MH - *Neoplasms, Muscle Tissue/surgery/pathology/diagnostic imaging/genetics

MH - Infant, Newborn

MH - Skull Neoplasms/surgery/diagnostic imaging/pathology/genetics

MH - Skull/diagnostic imaging/surgery/pathology

OTO - NOTNLM

OT - Anaplastic Lymphoma Kinase (ALK) expression

OT - Craniofacial

OT - Inflammatory Myofibroblastic Tumors (IMT)

OT - Pediatric skull lesion

EDAT- 2024/06/26 00:42

MHDA- 2024/11/05 19:20

CRDT- 2024/06/25 23:15

PHST- 2024/04/16 00:00 [received]

PHST- 2024/06/19 00:00 [accepted]

PHST- 2024/11/05 19:20 [medline]

PHST- 2024/06/26 00:42 [pubmed]

PHST- 2024/06/25 23:15 [entrez]

AID - 10.1007/s00381-024-06512-7 [pii]

AID - 10.1007/s00381-024-06512-7 [doi]

PST - ppublish

SO - Childs Nerv Syst. 2024 Nov;40(11):3829-3835. doi: 10.1007/s00381-024-06512-7.

Epub 2024 Jun 25.

PMID- 38867367

OWN - NLM

STAT- MEDLINE

DCOM- 20240725

LR - 20240830

IS - 1545-5017 (Electronic)

IS - 1545-5009 (Linking)

VI - 71

IP - 9

DP - 2024 Sep

TI - Population modeling analyses of crizotinib in pediatric patients with

ALK-positive advanced cancers.

PG - e31139

LID - 10.1002/pbc.31139 [doi]

AB - BACKGROUND: Alterations in the ALK (anaplastic lymphoma kinase) gene play a

critical role in pathogenesis of anaplastic large cell lymphoma (ALCL).

Crizotinib is a small molecule competitive inhibitor of ALK, ROS1, and MET

kinases and was approved for pediatric patients with ALK-positive relapsed or

refractory, systemic ALCL, and ALK-positive unresectable, recurrent, or

refractory inflammatory myofibroblastic tumors (IMT). PROCEDURE: Crizotinib data

from pediatric patients with relapsed or refractory solid tumors, IMT, or ALCL

were included in the analyses. All patients received crizotinib orally at doses

ranging from 100 to 365 mg/m(2) twice daily (BID). PopPK analyses were conducted

to characterize crizotinib disposition in pediatric patients. Exposure-response

(ER) safety and antitumor analyses were conducted to characterize relationships

between crizotinib dose or exposure with safety and antitumor activity endpoints

of interest. RESULTS: The population pharmacokinetic (popPK), ER safety, and ER

antitumor analysis included 98, 110, and 36 pediatric patients, respectively. A

one-compartment pharmacokinetic model with allometric scaling, first-order

elimination, and first-order absorption with lag time adequately described the

data. Natural log-transformed model-predicted crizotinib AUC(ss) (steady-state

area under the concentration-time curve) demonstrated a significant, positive

relationship with Grade ≥3 NEUTROPENIA and Any Grade VISION DISORDER. Crizotinib

dose demonstrated a positive relationship with objective response rate.

CONCLUSIONS: No significant differences in PK were identified across a wide range

of ages or across tumor types, suggesting body surface area (BSA)-based dosing

adequately adjusted for differences in patient size to achieve similar systemic

crizotinib exposures across young children and adolescent pediatric patients.

None of the myelosuppressive events except Grade ≥3 NEUTROPENIA had significant

relationships identified with crizotinib dose or exposure, suggesting crizotinib

is a tolerable treatment with less hematological toxicity than traditional

chemotherapy regimens for pediatric patients with ALK-mutated cancers. Results

from the presented analyses support the pediatric dosing recommendations in the

product label.

CI - © 2024 Wiley Periodicals LLC.

FAU - Jerry, Li

AU - Jerry L

AUID- ORCID: 0000-0002-8774-826X

AD - Clinical Pharmacology, Pfizer Inc., New York, New York, USA.

FAU - Swan, Lin

AU - Swan L

AD - Clinical Pharmacology, Neurocrine Biosciences, San Diego, California, USA.

FAU - Dana, Nickens

AU - Dana N

AD - Clinical Pharmacology, Pfizer Inc., New York, New York, USA.

FAU - Balis, Frank M

AU - Balis FM

AD - Department of Pediatrics, The Children's Hospital of Philadelphia, Philadelphia,

Pennsylvania, USA.

FAU - Greengard, Emily

AU - Greengard E

AUID- ORCID: 0000-0002-2963-5638

AD - Department of Pediatrics, University of Minnesota, Minneapolis, Minnesota, USA.

FAU - Huiping, Xu

AU - Huiping X

AD - Clinical Pharmacology, Pfizer Inc., New York, New York, USA.

LA - eng

PT - Journal Article

DEP - 20240612

PL - United States

TA - Pediatr Blood Cancer

JT - Pediatric blood & cancer

JID - 101186624

RN - 53AH36668S (Crizotinib)

RN - EC 2.7.10.1 (Anaplastic Lymphoma Kinase)

RN - EC 2.7.10.1 (ALK protein, human)

RN - 0 (Protein Kinase Inhibitors)

SB - IM

MH - Humans

MH - *Crizotinib/therapeutic use/pharmacokinetics

MH - Child

MH - *Anaplastic Lymphoma Kinase/antagonists & inhibitors/genetics

MH - Female

MH - Male

MH - Adolescent

MH - Child, Preschool

MH - *Protein Kinase Inhibitors/therapeutic use/pharmacokinetics/adverse effects

MH - Neoplasms/drug therapy/pathology

MH - Lymphoma, Large-Cell, Anaplastic/drug therapy/pathology

MH - Young Adult

MH - Infant

OTO - NOTNLM

OT - ALCL

OT - IMT

OT - crizotinib

OT - efficacy

OT - pharmacokinetics

OT - safety

EDAT- 2024/06/13 06:44

MHDA- 2024/07/26 12:39

CRDT- 2024/06/13 00:34

PHST- 2024/05/15 00:00 [revised]

PHST- 2024/02/08 00:00 [received]

PHST- 2024/05/24 00:00 [accepted]

PHST- 2024/07/26 12:39 [medline]

PHST- 2024/06/13 06:44 [pubmed]

PHST- 2024/06/13 00:34 [entrez]

AID - 10.1002/pbc.31139 [doi]

PST - ppublish

SO - Pediatr Blood Cancer. 2024 Sep;71(9):e31139. doi: 10.1002/pbc.31139. Epub 2024

Jun 12.

PMID- 38864087

OWN - NLM

STAT- PubMed-not-MEDLINE

LR - 20240613

IS - 1735-5303 (Print)

IS - 2345-3656 (Electronic)

IS - 1735-5303 (Linking)

VI - 19

IP - 1

DP - 2024 Winter

TI - Pediatric Inﬂammatory Myoﬁbroblastic Tumor of Rectosigmoid Junction: A Case

Report and Review of the Literature.

PG - 132-136

LID - 10.30699/ijp.2024.2003653.3122 [doi]

AB - The occurrence of rectosigmoid junction inflammatory myofibroblastic tumor (IMT)

is uncommon in children. This is a rare form of mesenchymal tumor, belonging to

the category of soft tissue tumors, and can be found at any anatomical site from

the central nervous system to the gastrointestinal tract. Our patient was a

10-year-old male subject complaining of lack of defecation and constipation. The

patient had decreased the frequency of defecation and constipation about two

weeks before his referral and had not improved despite the use of laxatives. The

abdomen was completely distended and there was no tenderness or guarding in the

examination. Several airfluid levels are shown on the abdominal X-ray. In the

ultrasound, free fluid was reported in the interlobular and pelvic spaces. The

patient was transferred into the operating room. A tumor of the rectosigmoid

junction was detected. Histopathologic studies showed evidence of IMT. IMT is a

rare neoplasm of unknown origin, which may occur in various sites of the body.

Complete surgical removal is usually curative, but early detection of recurrence

is required. Treatment options include chemotherapy, radiation therapy, and

immunotherapy. Further investigations are needed to improve the understanding and

management of this rare tumor.

CI - © 2024.

FAU - Soti Khiabani, Mahsa

AU - Soti Khiabani M

AD - Children's Medical Center, Pediatrics Center of Excellence, Tehran, Iran.

AD - Department of Pediatric Emergency, Tehran University of Medical Sciences, Tehran,

Iran.

FAU - Monajemzadeh, Maryam

AU - Monajemzadeh M

AD - Department of Pathology, Children Medical Center, Tehran University of Medical

Sciences, Tehran, Iran.

AD - Pediatric Gastroenterology and Hepatology Research Center, Pediatrics Centre of

Excellence, Children's Medical Center, Tehran University of Medical Sciences,

Tehran, Iran.

FAU - Raji, Hojatollah

AU - Raji H

AD - Children's Medical Center, Pediatrics Center of Excellence, Tehran, Iran.

AD - Department of Pediatric Surgery, Children's Medical Center, Tehran University of

Medical Sciences, Tehran, Iran.

FAU - Zamani, Fatemeh

AU - Zamani F

AD - Department of Radiology, Children Medical Center of Excellence, Tehran University

of Medical Science, Tehran, Iran.

AD - Advanced Diagnostic and Interventional Radiology Research Center, Tehran

University of Medical Sciences, Tehran, Iran.

FAU - Vaseie, Mohammad

AU - Vaseie M

AD - Emergency Medicine Department, Faculty of Medicine, Tehran University of Medical

Sciences, Tehran, Iran.

FAU - Pak, Neda

AU - Pak N

AD - Department of Radiology, Children Medical Centre of Excellence, Tehran University

of Medical Sciences, Tehran, Iran.

LA - eng

PT - Case Reports

PT - Journal Article

DEP - 20231229

PL - Iran

TA - Iran J Pathol

JT - Iranian journal of pathology

JID - 101515128

PMC - PMC11164313

OTO - NOTNLM

OT - Computed tomography

OT - Diagnosis

OT - Inflammatory myofibroblastic tumor

OT - X-ray

COIS- The authors declare that they have no competing interests.

EDAT- 2024/06/12 06:42

MHDA- 2024/06/12 06:43

PMCR- 2024/01/01

CRDT- 2024/06/12 04:15

PHST- 2023/05/31 00:00 [received]

PHST- 2023/09/14 00:00 [accepted]

PHST- 2024/06/12 06:43 [medline]

PHST- 2024/06/12 06:42 [pubmed]

PHST- 2024/06/12 04:15 [entrez]

PHST- 2024/01/01 00:00 [pmc-release]

AID - 10.30699/ijp.2024.2003653.3122 [doi]

PST - ppublish

SO - Iran J Pathol. 2024 Winter;19(1):132-136. doi: 10.30699/ijp.2024.2003653.3122.

Epub 2023 Dec 29.

PMID- 38856321

OWN - NLM

STAT- MEDLINE

DCOM- 20241107

LR - 20241130

IS - 1305-3612 (Electronic)

IS - 1305-3825 (Print)

IS - 1305-3825 (Linking)

VI - 30

IP - 6

DP - 2024 Nov 6

TI - Imaging findings of primary lung tumors in children.

PG - 419-426

LID - 10.4274/dir.2024.242714 [doi]

AB - PURPOSE: Pediatric lung tumors are primarily discussed in the surgical

literature. However, limited research has been reported on their imaging

findings, and only a few tumor types have been documented. Therefore, the aim of

this article is to describe the imaging features of primary lung tumors in

children. METHODS: The archives of the pediatric radiology unit were reviewed for

primary lung tumors documented between 2007 and 2023. In total, 24 patients (9

girls and 15 boys; aged 5 months to 16 years) were included in the study. Their

demographic characteristics, clinical presentation, and histopathologic results

were obtained. All imaging studies were reviewed by two radiologists for various

findings (e.g., lymphadenopathy, atelectasis, pleural effusion, calcification,

multiplicity, pneumothorax, axial and lobar location, laterality, tumor margin,

mediastinal shift, contrast enhancement pattern, signal intensity on T1- and

T2-weighted images, and diffusion pattern), and a final decision was made by

consensus. The mean tumor size was compared between the benign and malignant

groups using a t-test. RESULTS: There were 15 (62.5%) benign tumors, as follows:

inflammatory myofibroblastic tumor (IMT; n = 10, 41%), hemangioma (n = 2, 8%),

pneumocytoma (n = 2, 8%), and mature cystic teratoma (n = 1, 4%). Moreover, there

were 9 (37.5%) malignant tumors, as follows: pleuropulmonary blastoma (PPB; n =

6, 25%), adenocarcinoma (n = 2, 8%), and lymphoepithelioma-like carcinoma (LELC)

(n = 1, 4%). The most frequently reported symptoms were cough, fever, dyspnea,

chest pain, and recurrent infection; six patients reported no clinical symptoms.

Fifteen tumors (62%) were located in the right lung. The mean tumor diameter at

the time of diagnosis was 6.4 ± 3 cm (benign group: 6.7 ± 3.4 cm; malignant

group: 6 ± 2.3 cm, P > 0.050). Calcification was present in 80% of the patients

with IMT. At the time of diagnosis, two (8.3%) patients were found to have

metastasis: one was diagnosed with adenocarcinoma and the other with LELC. Tumors

were located peripherally in 18 (75%) patients. CONCLUSION: The symptoms

associated with lung masses are non-specific. There is no correlation between

tumor size and malignancy. The most common tumors observed in this study were IMT

and PPB, respectively. IMT is highly associated with calcification. CLINICAL

SIGNIFICANCE: Primary lung tumors are rarely seen in children, and they have

different histopathological types. Calcification might be an important

radiological clue for the diagnosis of IMT, which is the most common lung tumor

in children.

FAU - Özcan, H Nursun

AU - Özcan HN

AUID- ORCID: 0000-0003-4756-4359

AD - Hacettepe University Faculty of Medicine, Department of Radiology, Division of

Pediatric Radiology, Ankara, Türkiye

FAU - Atak, Fırat

AU - Atak F

AUID- ORCID: 0000-0003-1474-1582

AD - Hacettepe University Faculty of Medicine, Department of Radiology, Ankara,

Türkiye

FAU - Oğuz, Berna

AU - Oğuz B

AUID- ORCID: 0000-0003-0399-3741

AD - Hacettepe University Faculty of Medicine, Department of Radiology, Division of

Pediatric Radiology, Ankara, Türkiye

FAU - Kutluk, Tezer

AU - Kutluk T

AUID- ORCID: 0000-0002-1918-4407

AD - Hacettepe University Faculty of Medicine, Department of Pediatrics, Division of

Pediatric Oncology, Ankara, Türkiye

FAU - Haliloğlu, Mithat

AU - Haliloğlu M

AUID- ORCID: 0000-0002-8502-2422

AD - Hacettepe University Faculty of Medicine, Department of Radiology, Division of

Pediatric Radiology, Ankara, Türkiye

LA - eng

PT - Journal Article

DEP - 20240610

PL - Turkey

TA - Diagn Interv Radiol

JT - Diagnostic and interventional radiology (Ankara, Turkey)

JID - 101241152

SB - IM

MH - Humans

MH - Male

MH - Female

MH - Child

MH - Adolescent

MH - Child, Preschool

MH - Infant

MH - *Lung Neoplasms/diagnostic imaging/pathology

MH - Retrospective Studies

MH - Tomography, X-Ray Computed/methods

MH - Magnetic Resonance Imaging/methods

PMC - PMC11589528

OTO - NOTNLM

OT - CT

OT - Children

OT - MRI

OT - cancer

OT - inflammatory myofibroblastic tumor

OT - lung

COIS- Conflict of interest disclosure: The authors declared no conflicts of interest.

EDAT- 2024/06/10 13:21

MHDA- 2024/11/07 06:23

PMCR- 2024/11/06

CRDT- 2024/06/10 09:27

PHST- 2024/11/07 06:23 [medline]

PHST- 2024/06/10 13:21 [pubmed]

PHST- 2024/06/10 09:27 [entrez]

PHST- 2024/11/06 00:00 [pmc-release]

AID - 10.4274/dir.2024.242714 [doi]

PST - ppublish

SO - Diagn Interv Radiol. 2024 Nov 6;30(6):419-426. doi: 10.4274/dir.2024.242714. Epub

2024 Jun 10.

PMID- 38839088

OWN - NLM

STAT- Publisher

LR - 20240605

IS - 1472-4146 (Electronic)

IS - 0021-9746 (Linking)

DP - 2024 Jun 4

TI - ALK-rearranged mesenchymal neoplasms: a clinicopathological and molecular study

of eight additional cases of an emerging group of tyrosine kinase fusion

mesenchymal tumours.

LID - jcp-2024-209521 [pii]

LID - 10.1136/jcp-2024-209521 [doi]

AB - AIMS: Mesenchymal neoplasms characterised by ALK fusions mainly include

inflammatory myofibroblastic tumour (IMT) and epithelioid fibrous histiocytoma

(EFH). Most recently, ALK-rearranged mesenchymal tumours that are not IMT or EFH

have been reported. Our aim is to further characterise eight such neoplasms, with

a detailed clinicopathological, immunohistochemical and molecular analysis.

METHODS: Clinicopathological features were assessed and partner agnostic targeted

RNA-sequencing on clinically validated platforms was performed. RESULTS: The

patients consisted of seven males and one female with a median age of 47 years

(28 -59 years). The tumours ranged in size from 2.0 to 10.0 cm (mean=3.0 cm) and

involved superficial and deep soft tissue (n=6) and visceral locations (n=2). Of

the seven patients with follow-up (9-130 months), two developed distant

metastases and five had no disease recurrence or metastasis. The tumours

demonstrated diverse architectures and variable cellularity and cellular

morphologies. The main constitutive cells appeared in elongated spindled in

three, primitive to ovoid in two and round to epithelioid in three cases. We

expanded the histopathological spectrum to include mildly to moderately cellular

spindled to stellate cells in a multinodular growth in a prominent myxoid and

vascularised stroma (n=2). All tumours expressed ALK(D5F3); seven were positive

for S100 protein and six were positive for CD34. By fluorescence in situ

hybridisation, ALK rearrangement was identified in all eight tumours. ALK fusion

partners were identified by RNA-sequencing in all cases, including previously

reported: EML4 (n=3), DCTN (n=1), CLIP1 (n=1) and PLEKHH2 (n=1), and also two

novel fusion partners: TKT (n=1) and MMP2 (n=1). CONCLUSIONS: Our study expands

the clinicopathological and molecular spectrum of ALK-rearranged mesenchymal

neoplasms.

CI - © Author(s) (or their employer(s)) 2024. No commercial re-use. See rights and

permissions. Published by BMJ.

FAU - Zhao, Ming

AU - Zhao M

AUID- ORCID: 0000-0001-5971-7303

AD - Ningbo Clinical Pathology Diagnosis Center, Ningbo, China.

FAU - Song, Jing

AU - Song J

AD - Ningbo Clinical Pathology Diagnosis Center, Ningbo, China.

FAU - Yin, Xiaona

AU - Yin X

AD - Ningbo Clinical Pathology Diagnosis Center, Ningbo, China.

FAU - Xu, Jiayun

AU - Xu J

AD - Ningbo Clinical Pathology Diagnosis Center, Ningbo, China.

FAU - Teng, Xiaodong

AU - Teng X

AUID- ORCID: 0000-0002-8700-4770

AD - Department of Pathology, The First Affiliated Hospital, Zhejiang University

School of Medicine, Hangzhou, China.

FAU - Wang, Jian

AU - Wang J

AUID- ORCID: 0000-0003-4082-7750

AD - Department of Pathology, Fudan University Shanghai Cancer Center; Department of

Oncology, Shanghai Medical College, Fudan University; Institute of Pathology,

Fudan University, Shanghai, China softtissuetumor@163.com.

LA - eng

PT - Journal Article

DEP - 20240604

PL - England

TA - J Clin Pathol

JT - Journal of clinical pathology

JID - 0376601

SB - IM

OTO - NOTNLM

OT - GENETICS

OT - IMMUNOHISTOCHEMISTRY

OT - Soft Tissue Neoplasms

COIS- Competing interests: None declared.

EDAT- 2024/06/06 01:14

MHDA- 2024/06/06 01:14

CRDT- 2024/06/05 20:42

PHST- 2024/03/11 00:00 [received]

PHST- 2024/04/22 00:00 [accepted]

PHST- 2024/06/06 01:14 [medline]

PHST- 2024/06/06 01:14 [pubmed]

PHST- 2024/06/05 20:42 [entrez]

AID - jcp-2024-209521 [pii]

AID - 10.1136/jcp-2024-209521 [doi]

PST - aheadofprint

SO - J Clin Pathol. 2024 Jun 4:jcp-2024-209521. doi: 10.1136/jcp-2024-209521.

PMID- 38838115

OWN - NLM

STAT- MEDLINE

DCOM- 20240605

LR - 20240606

IS - 2078-5151 (Electronic)

IS - 0038-2361 (Linking)

VI - 62

IP - 2

DP - 2024 May

TI - Inflammatory myofibroblastic tumours of the liver - a systematic review.

PG - 23-27

AB - BACKGROUND: Hepatic inflammatory myofibroblastic tumours (HIMTs) are rare and

poorly described in the literature. Most publications are single patient case

reports and lack detailed reporting on characteristics, management, and outcomes.

This systematic review aimed to assess the demography, clinical presentation,

typical imaging features, histopathology, treatment, and outcomes of patients

presenting with HIMTs. METHODS: A systematic literature search was performed in

MEDLINE (PubMed), EMBASE (Scopus), JSTOR, Cochrane CENTRAL (Cochrane Library),

and the databases included in the Web of Science for studies published between

1940 and 2023 on HIMTs, including its reported synonyms. Case series or cohort

studies that reported on the management and outcomes of at least four patients

with histologically confirmed HIMTs were included in the analysis. RESULTS: After

screening 4553 publications, 22 articles including a total of 440 patients with

confirmed HIMTs were eligible for inclusion. The average age was 53.4 years

(range 42.0-65.0) with a male to female ratio of 1.7:1. Abdominal pain,

discomfort, fever, and loss of weight were the most common presenting symptoms.

Surgical resection is the standard of care for HIMTs and is associated with low

mortality of 3.4% and low disease recurrence. CONCLUSION: HIMT is a disease more

often affecting middle-aged males. The lesions are typically solitary with low

recurrence after treatment. The relative roles of surgical versus medical

treatment remain unclear. Differences in clinical presentation, histopathology,

and treatment of HIMTs compared to inflammatory myofibroblastic tumour (IMT) at

extrahepatic sites could challenge the current view of IMT as a single

pathological entity.

CI - Copyright© Authors.

FAU - Li, M

AU - Li M

AD - Division of General Surgery, Department of Surgery, Groote Schuur Hospital,

University of Cape Town, South Africa.

AD - University of Chicago Centre for Global Health, United States of America.

AD - Cancer Genomics Group, International Centre for Genetic Engineering and

Biotechnology, University of Cape Town, South Africa.

FAU - Sobnach, S

AU - Sobnach S

AD - Division of General Surgery, Department of Surgery, Groote Schuur Hospital,

University of Cape Town, South Africa.

FAU - Kotze, U K

AU - Kotze UK

AD - Division of General Surgery, Department of Surgery, Groote Schuur Hospital,

University of Cape Town, South Africa.

FAU - Zerbini, L F

AU - Zerbini LF

AD - Cancer Genomics Group, International Centre for Genetic Engineering and

Biotechnology, University of Cape Town, South Africa.

FAU - Millis, J M

AU - Millis JM

AD - Department of Surgery, University of Chicago Medicine, United States of America.

FAU - Hampton, D A

AU - Hampton DA

AD - Department of Surgery, University of Chicago Medicine, United States of America.

FAU - Bernon, M M

AU - Bernon MM

AD - Division of General Surgery, Department of Surgery, Groote Schuur Hospital,

University of Cape Town, South Africa.

FAU - Krige, J E J

AU - Krige JEJ

AD - Division of General Surgery, Department of Surgery, Groote Schuur Hospital,

University of Cape Town, South Africa.

FAU - Jonas, E G

AU - Jonas EG

AD - Division of General Surgery, Department of Surgery, Groote Schuur Hospital,

University of Cape Town, South Africa.

LA - eng

PT - Journal Article

PT - Systematic Review

PL - South Africa

TA - S Afr J Surg

JT - South African journal of surgery. Suid-Afrikaanse tydskrif vir chirurgie

JID - 2984854R

SB - IM

MH - Humans

MH - *Liver Neoplasms/pathology/therapy/surgery

MH - Granuloma, Plasma Cell/surgery/pathology/diagnosis

MH - Male

MH - Neoplasms, Muscle Tissue/surgery/pathology/diagnosis

MH - Female

MH - Middle Aged

EDAT- 2024/06/05 19:12

MHDA- 2024/06/05 19:13

CRDT- 2024/06/05 14:03

PHST- 2024/06/05 19:13 [medline]

PHST- 2024/06/05 19:12 [pubmed]

PHST- 2024/06/05 14:03 [entrez]

PST - ppublish

SO - S Afr J Surg. 2024 May;62(2):23-27.

PMID- 38834463

OWN - NLM

STAT- In-Process

LR - 20241108

IS - 0219-3108 (Electronic)

IS - 1015-9584 (Linking)

VI - 47

IP - 11

DP - 2024 Nov

TI - The role of multimodal CT imaging in a rare case of pediatric inflammatory

myofibroblastic trachea tumor.

PG - 4796-4797

LID - S1015-9584(24)01026-1 [pii]

LID - 10.1016/j.asjsur.2024.05.133 [doi]

FAU - Han, Limei

AU - Han L

AD - Department of Radiology, Affiliated Hospital of North Sichuan Medical College,

637000, Nanchong, Sichuan Province, PR China; Department of Radiology, Zigong

First People's Hospital, Zigong, Sichuan Province, 643000, PR China.

FAU - Zhang, Wei

AU - Zhang W

AD - Department of Radiology, Affiliated Hospital of North Sichuan Medical College,

637000, Nanchong, Sichuan Province, PR China; Department of Radiology, Zigong

First People's Hospital, Zigong, Sichuan Province, 643000, PR China.

FAU - Xu, Qiaomei

AU - Xu Q

AD - Department of Radiology, Zigong First People's Hospital, Zigong, Sichuan

Province, 643000, PR China.

FAU - Zhong, Jianquan

AU - Zhong J

AD - Department of Radiology, Zigong First People's Hospital, Zigong, Sichuan

Province, 643000, PR China. Electronic address: zhongjianquan.2010@qq.com.

LA - eng

PT - Letter

DEP - 20240603

PL - Netherlands

TA - Asian J Surg

JT - Asian journal of surgery

JID - 8900600

SB - IM

OTO - NOTNLM

OT - Inflammatory myofibroblastic tumor

OT - Inflammatory pseudotumor

OT - Multimodal CT imaging

EDAT- 2024/06/05 02:17

MHDA- 2024/06/05 02:17

CRDT- 2024/06/04 21:55

PHST- 2024/04/12 00:00 [received]

PHST- 2024/05/16 00:00 [accepted]

PHST- 2024/06/05 02:17 [pubmed]

PHST- 2024/06/05 02:17 [medline]

PHST- 2024/06/04 21:55 [entrez]

AID - S1015-9584(24)01026-1 [pii]

AID - 10.1016/j.asjsur.2024.05.133 [doi]

PST - ppublish

SO - Asian J Surg. 2024 Nov;47(11):4796-4797. doi: 10.1016/j.asjsur.2024.05.133. Epub

2024 Jun 3.

PMID- 38817997

OWN - NLM

STAT- PubMed-not-MEDLINE

LR - 20240601

IS - 0975-7651 (Print)

IS - 0976-6952 (Electronic)

IS - 0975-7651 (Linking)

VI - 15

IP - Suppl 2

DP - 2024 May

TI - Intra-abdominal Inflammatory Myofibroblastic Tumour (IMFT)-Uncommon Entity.

PG - 344-348

LID - 10.1007/s13193-023-01869-8 [doi]

AB - IMFT (inflammatory myofibroblastic tumour) is an uncommon tumour predominantly

affecting the lungs and mediastinum. Most of the published literature supports

that it affects children and young individuals. IMFT involving the

gastrointestinal tract is rare. We report a case of multifocal IMFT affecting the

GI tract which was managed with gross total excision followed by chemotherapy.

Surgical resection remains the treatment of choice. The role of chemotherapy and

radiation therapy remains limited. The aetiology of these tumours remains unclear

and is mostly ALK-positive that could be targeted. Local recurrences are common

and hence require close follow-up. The risk of recurrences and metastasis is

increased in cases with TP53 positivity, aneuploidy and recurrent lesions.

CI - © The Author(s), under exclusive licence to Indian Association of Surgical

Oncology 2023. Springer Nature or its licensor (e.g. a society or other partner)

holds exclusive rights to this article under a publishing agreement with the

author(s) or other rightsholder(s); author self-archiving of the accepted

manuscript version of this article is solely governed by the terms of such

publishing agreement and applicable law.

FAU - Dwivedi, Surjeet

AU - Dwivedi S

AUID- ORCID: 0000-0001-6595-0718

AD - Command Hospital Air Force, Bangalore, India. ROR: https://ror.org/05cx69s52.

GRID: grid.414640.3. ISNI: 0000 0004 1782 2908

FAU - Rakesh, C R

AU - Rakesh CR

AD - Command Hospital Air Force, Bangalore, India. ROR: https://ror.org/05cx69s52.

GRID: grid.414640.3. ISNI: 0000 0004 1782 2908

FAU - Anand, S

AU - Anand S

AD - Command Hospital Air Force, Bangalore, India. ROR: https://ror.org/05cx69s52.

GRID: grid.414640.3. ISNI: 0000 0004 1782 2908

FAU - Dogra, Natasha

AU - Dogra N

AD - Command Hospital Air Force, Bangalore, India. ROR: https://ror.org/05cx69s52.

GRID: grid.414640.3. ISNI: 0000 0004 1782 2908

FAU - Singh, Bhanu Pratap

AU - Singh BP

AD - Command Hospital Air Force, Bangalore, India. ROR: https://ror.org/05cx69s52.

GRID: grid.414640.3. ISNI: 0000 0004 1782 2908

LA - eng

PT - Case Reports

PT - Journal Article

DEP - 20240105

PL - India

TA - Indian J Surg Oncol

JT - Indian journal of surgical oncology

JID - 101532448

PMC - PMC11133245

OTO - NOTNLM

OT - ALK Rearrangement

OT - GI tract

OT - IMFT

OT - Recurrence

COIS- Conflict of InterestThe authors declare no competing interests.

EDAT- 2024/05/31 06:42

MHDA- 2024/05/31 06:43

PMCR- 2025/05/01

CRDT- 2024/05/31 03:55

PHST- 2023/11/23 00:00 [received]

PHST- 2023/12/18 00:00 [accepted]

PHST- 2025/05/01 00:00 [pmc-release]

PHST- 2024/05/31 06:43 [medline]

PHST- 2024/05/31 06:42 [pubmed]

PHST- 2024/05/31 03:55 [entrez]

AID - 1869 [pii]

AID - 10.1007/s13193-023-01869-8 [doi]

PST - ppublish

SO - Indian J Surg Oncol. 2024 May;15(Suppl 2):344-348. doi:

10.1007/s13193-023-01869-8. Epub 2024 Jan 5.

PMID- 38817466

OWN - NLM

STAT- PubMed-not-MEDLINE

LR - 20240601

IS - 2168-8184 (Print)

IS - 2168-8184 (Electronic)

IS - 2168-8184 (Linking)

VI - 16

IP - 4

DP - 2024 Apr

TI - Inflammatory Myofibroblastic Tumor After Receiving Treatment for Non-small Cell

Carcinoma.

PG - e59359

LID - 10.7759/cureus.59359 [doi]

LID - e59359

AB - Inflammatory pseudotumor encompasses a spectrum of both neoplastic and

non-neoplastic conditions characterized by a histological pattern featuring a

proliferation of cytologically bland spindle cells, accompanied by a prominent

chronic inflammatory infiltrate. Within this spectrum, inflammatory

myofibroblastic tumor (IMT) has emerged as a distinct entity over the past two

decades, marked by unique clinical, pathological, and molecular characteristics.

Typically affecting the visceral soft tissues of children and adolescents, IMT

exhibits a propensity for local recurrence while posing a minimal risk of distant

metastasis. They are extremely rare in adults, constituting less than 1% of adult

lung tumors. Our patient, a 63-year-old female, has an intricate medical

background, encompassing chronic obstructive pulmonary disease (COPD), a previous

history of smoking (35 pack-years, quit a year before admission), coronary artery

disease, non-obstructive hypertrophic cardiomyopathy, and obstructive sleep

apnea. Presenting with a diagnostic dilemma, she recently received treatment for

non-small cell carcinoma with radiation therapy, which has evolved into a swiftly

advancing case of IMT.

CI - Copyright © 2024, Parker et al.

FAU - Parker, Neil C

AU - Parker NC

AD - Internal Medicine, Southern Illinois University School of Medicine, Springfield,

USA.

FAU - Singanallur, Prashanth

AU - Singanallur P

AD - Pulmonary and Critical Care Medicine, Southern Illinois University School of

Medicine, Springfield, USA.

FAU - Faiek, Saif

AU - Faiek S

AD - Pulmonology and Critical Care, Southern Illinois University School of Medicine,

Springfield, USA.

FAU - Gao, John

AU - Gao J

AD - Pathology, Southern Illinois University School of Medicine, Springfield, USA.

FAU - White, Peter

AU - White P

AD - Pulmonology and Critical Care, Southern Illinois University School of Medicine,

Springfield, USA.

LA - eng

PT - Case Reports

PT - Journal Article

DEP - 20240430

PL - United States

TA - Cureus

JT - Cureus

JID - 101596737

PMC - PMC11138368

OTO - NOTNLM

OT - imt

OT - inflammatory myofibroblastic tumor (imt)

OT - lung tumor

OT - non-squamous cell lung cancer

OT - sbrt (stereotactic body radiotherapy)

COIS- The authors have declared that no competing interests exist.

EDAT- 2024/05/31 06:42

MHDA- 2024/05/31 06:43

PMCR- 2024/04/30

CRDT- 2024/05/31 03:47

PHST- 2024/04/27 00:00 [accepted]

PHST- 2024/05/31 06:43 [medline]

PHST- 2024/05/31 06:42 [pubmed]

PHST- 2024/05/31 03:47 [entrez]

PHST- 2024/04/30 00:00 [pmc-release]

AID - 10.7759/cureus.59359 [doi]

PST - epublish

SO - Cureus. 2024 Apr 30;16(4):e59359. doi: 10.7759/cureus.59359. eCollection 2024

Apr.

PMID- 38813309

OWN - NLM

STAT- PubMed-not-MEDLINE

LR - 20240531

IS - 2168-8184 (Print)

IS - 2168-8184 (Electronic)

IS - 2168-8184 (Linking)

VI - 16

IP - 4

DP - 2024 Apr

TI - A Case Report of Inflammatory Myofibroblastic Tumor: A Rare Benign Lung Tumor.

PG - e59237

LID - 10.7759/cureus.59237 [doi]

LID - e59237

AB - Inflammatory myofibroblastic tumors (IMTs) of the lung are a rare type of

mesenchymal tumors that tend to occur more in the lungs of children. They are

extremely rare in adults. IMTs require extensive pulmonary resection because they

are commonly locally invasive. The key to preventing recurrence is complete

resection, and the prognosis is excellent after surgery. We report a case of a

patient with an inflammatory pseudotumor of the lung. The patient is a

27-year-old female who presented with a dry cough. A chest radiograph and

computed tomography showed a lesion in the left main bronchus and near-total left

lung collapse. As surgery was necessary to establish the diagnosis, left

pneumonectomy was performed followed by a histological examination of the

surgical specimen which confirmed inflammatory pseudotumor.

CI - Copyright © 2024, Allama et al.

FAU - Allama, Amr M

AU - Allama AM

AD - Thoracic Surgery, King Fahad General Hospital, Madinah, SAU.

FAU - Almuhammadi, Ghaidaa A

AU - Almuhammadi GA

AD - College of Medicine, Taibah University, Madinah, SAU.

FAU - Alzughaibi, Rawia A

AU - Alzughaibi RA

AD - College of Medicine, Taibah University, Madinah, SAU.

FAU - Ishqi, Raha Z

AU - Ishqi RZ

AD - College of Medicine, Taibah University, Madinah, SAU.

FAU - Al-Refai, Mohammed A

AU - Al-Refai MA

AD - Thoracic Surgery, King Fahad General Hospital, Madinah, SAU.

LA - eng

PT - Case Reports

PT - Journal Article

DEP - 20240428

PL - United States

TA - Cureus

JT - Cureus

JID - 101596737

PMC - PMC11133774

OTO - NOTNLM

OT - benign tumor

OT - inflammatory myofibroblastic tumor

OT - inflammatory pseudotumor

OT - lung tumors

OT - pseudotumor

COIS- The authors have declared that no competing interests exist.

EDAT- 2024/05/30 06:35

MHDA- 2024/05/30 06:36

PMCR- 2024/04/28

CRDT- 2024/05/30 04:01

PHST- 2024/04/28 00:00 [accepted]

PHST- 2024/05/30 06:36 [medline]

PHST- 2024/05/30 06:35 [pubmed]

PHST- 2024/05/30 04:01 [entrez]

PHST- 2024/04/28 00:00 [pmc-release]

AID - 10.7759/cureus.59237 [doi]

PST - epublish

SO - Cureus. 2024 Apr 28;16(4):e59237. doi: 10.7759/cureus.59237. eCollection 2024

Apr.

PMID- 38803840

OWN - NLM

STAT- PubMed-not-MEDLINE

LR - 20240529

IS - 2042-8812 (Print)

IS - 2042-8812 (Electronic)

IS - 2042-8812 (Linking)

VI - 2024

IP - 5

DP - 2024 May

TI - Inflammatory myofibroblastic tumor of the cecum presenting as acute abdomen, a

rare pathology with a rarer presentation.

PG - rjae330

LID - 10.1093/jscr/rjae330 [doi]

LID - rjae330

AB - Inflammatory myofibroblastic tumor is an extremely rare neoplastic lesion with a

predilection for aggressive local and recurrent behavior. The tumor tends to

occur in the lungs of children and young adults, and although it can develop in

older patients and other organs, this is extremely rare. Symptoms are nonspecific

and depend on the location and size of the tumor. The gastrointestinal tract is

rarely this mass's primary site of origin, and the cecum is an even rarer

location. We present the case of an otherwise healthy 55-year-old female who

presented with an acute abdomen and a mass in her abdomen; after successful

surgery, she fully recovered. Inflammatory myofibroblastic tumor causing acute

abdomen was the final diagnosis.

CI - Published by Oxford University Press and JSCR Publishing Ltd. © The Author(s)

2024.

FAU - Molina, Gabriel A

AU - Molina GA

AUID- ORCID: 0000-0003-0001-9070

AD - USFQ (Universidad San Francisco de Quito) - Colegio de Ciencias de la Salud,

170902, Quito, Ecuador.

FAU - Ludeña, Carolina

AU - Ludeña C

AD - PGY3 UDLA (Universidad de las Américas) - School of Medicine, 170513, Quito,

Ecuador.

FAU - Carrera, Paul Alexander

AU - Carrera PA

AD - Department of Surgery Iess Quito Sur, 170111, Quito, Ecuador.

FAU - Heredia, Andrea E

AU - Heredia AE

AD - Department of Surgery Iess Quito Sur, 170111, Quito, Ecuador.

FAU - Jimenez, Galo E

AU - Jimenez GE

AD - Department of Surgery Iess Quito Sur, 170111, Quito, Ecuador.

FAU - Parrales, Diana E

AU - Parrales DE

AD - Department of Surgery Iess Quito Sur, 170111, Quito, Ecuador.

FAU - Portilla, Carolina Alexandra

AU - Portilla CA

AD - Universidad Catolica del Ecuador, 170143, Quito, Ecuador.

FAU - Martinez, Sebastian Nicolay

AU - Martinez SN

AD - USFQ (Universidad San Francisco de Quito) - Colegio de Ciencias de la Salud,

170902, Quito, Ecuador.

FAU - Ochoa-Andrade, Miguel Jacob

AU - Ochoa-Andrade MJ

AUID- ORCID: 0000-0001-5505-5285

AD - UCE (Universidad Central del Ecuador) - School of Medicine, 170129, Quito,

Ecuador.

LA - eng

PT - Case Reports

PT - Journal Article

DEP - 20240526

PL - England

TA - J Surg Case Rep

JT - Journal of surgical case reports

JID - 101560169

PMC - PMC11129661

OTO - NOTNLM

OT - acute abdomen

OT - colon

OT - inflammatory myofibroblastic tumor

COIS- None declared.

EDAT- 2024/05/28 06:43

MHDA- 2024/05/28 06:44

PMCR- 2024/05/26

CRDT- 2024/05/28 03:46

PHST- 2024/04/10 00:00 [received]

PHST- 2024/05/01 00:00 [accepted]

PHST- 2024/05/28 06:44 [medline]

PHST- 2024/05/28 06:43 [pubmed]

PHST- 2024/05/28 03:46 [entrez]

PHST- 2024/05/26 00:00 [pmc-release]

AID - rjae330 [pii]

AID - 10.1093/jscr/rjae330 [doi]

PST - epublish

SO - J Surg Case Rep. 2024 May 26;2024(5):rjae330. doi: 10.1093/jscr/rjae330.

eCollection 2024 May.

PMID- 38787978

OWN - NLM

STAT- MEDLINE

DCOM- 20240524

LR - 20240723

IS - 1536-5964 (Electronic)

IS - 0025-7974 (Print)

IS - 0025-7974 (Linking)

VI - 103

IP - 21

DP - 2024 May 24

TI - Two cases of inflammatory myofibroblastic tumor treated with targeted drugs: A

case report.

PG - e38136

LID - 10.1097/MD.0000000000038136 [doi]

LID - e38136

AB - INTRODUCTION: Inflammatory myofibroblastic tumor (IMT) is a rare invasive soft

tissue tumor. Many IMTs are positive for anaplastic lymphoma kinase (ALK) with

ALK gene fusion; other gene mutations have also been reported, which indicates a

key role for genetic testing and the development of target therapy to optimize

treatment strategies. PATIENT CONCERNS: We report 2 patients who obtained

clinical benefits following targeted treatment with ensartinib. DIAGNOSIS: The

first patient was diagnosed as IMT, with TFG-ROS1 fusion gene mutation. The

second patient was IMT harboring the ALK-STRN fusion gene mutation.

INTERVENTIONS: We performed gene testing for these 2 patients. According to the

test result, both patients received ensartinib 225 mg QD as targeted therapy for

a 30-day cycle. OUTCOMES: The first patient achieved partial remission and

maintained a stable state for 14.7 months. The second patient was treated for 10

months and reached complete remission after 5 months and is currently still

benefiting from treatment. Treatment-related side effects were mild in both

patients. CONCLUSION: Our cases provided some new insights and approaches for the

clinical diagnosis and treatment of IMT.

CI - Copyright © 2024 the Author(s). Published by Wolters Kluwer Health, Inc.

FAU - Liu, Mengyao

AU - Liu M

AUID- ORCID: 0009-0006-0416-6253

AD - Rare Tumors Department, Shandong Cancer Hospital and Institute, Shandong First

Medical University and Shandong Academy of Medical Sciences, Jinan, China.

FAU - Zhu, Dongyuan

AU - Zhu D

LA - eng

PT - Case Reports

PT - Journal Article

PL - United States

TA - Medicine (Baltimore)

JT - Medicine

JID - 2985248R

RN - EC 2.7.10.1 (Anaplastic Lymphoma Kinase)

RN - 0 (Antineoplastic Agents)

SB - IM

MH - Humans

MH - Anaplastic Lymphoma Kinase/genetics

MH - Antineoplastic Agents/therapeutic use

MH - *Neoplasms, Muscle Tissue/drug therapy/genetics/pathology

MH - Soft Tissue Neoplasms/drug therapy/genetics/pathology

PMC - PMC11124583

COIS- The authors have no funding and conflicts of interest to disclose.

EDAT- 2024/05/24 18:43

MHDA- 2024/05/24 18:44

PMCR- 2024/05/24

CRDT- 2024/05/24 14:13

PHST- 2024/05/24 18:44 [medline]

PHST- 2024/05/24 18:43 [pubmed]

PHST- 2024/05/24 14:13 [entrez]

PHST- 2024/05/24 00:00 [pmc-release]

AID - 00005792-202405240-00012 [pii]

AID - MD-D-24-00692 [pii]

AID - 10.1097/MD.0000000000038136 [doi]

PST - ppublish

SO - Medicine (Baltimore). 2024 May 24;103(21):e38136. doi:

10.1097/MD.0000000000038136.

PMID- 38766740

OWN - NLM

STAT- PubMed-not-MEDLINE

LR - 20240523

IS - 2383-7837 (Print)

IS - 2383-7845 (Electronic)

IS - 2383-7837 (Linking)

VI - 58

IP - 3

DP - 2024 May

TI - Primary epithelioid inflammatory myofibroblastic sarcoma of the brain with

EML4::ALK fusion mimicking intra-axial glioma: a case report and brief literature

review.

PG - 141-145

LID - 10.4132/jptm.2024.04.12 [doi]

AB - An aggressive subtype of inflammatory myofibroblastic tumor, epithelioid

inflammatory myofibroblastic sarcoma occurs primarily inside the abdominal

cavity, followed by a pulmonary localization. Most harbor anaplastic lymphoma

kinase (ALK) gene rearrangements, with RANBP2 and RRBP1 among the well-documented

fusion partners. We report the second case of primary epithelioid inflammatory

myofibroblastic sarcoma of the brain, with a well-known EML4::ALK fusion. The

case is notable for its intra-axial presentation that clinico-radiologically

mimicked glioma.

FAU - Kim, Eric Eunshik

AU - Kim EE

AD - Departments of Pathology, Seoul National University Hospital, Seoul National

University College of Medicine, Seoul, Korea.

FAU - Park, Chul-Kee

AU - Park CK

AD - Departments of Neurosurgery, Seoul National University Hospital, Seoul National

University College of Medicine, Seoul, Korea.

FAU - Kang, Koung Mi

AU - Kang KM

AD - Departments of Radiology, Seoul National University Hospital, Seoul National

University College of Medicine, Seoul, Korea.

FAU - Kwak, Yoonjin

AU - Kwak Y

AD - Departments of Pathology, Seoul National University Hospital, Seoul National

University College of Medicine, Seoul, Korea.

FAU - Park, Sung-Hye

AU - Park SH

AD - Departments of Pathology, Seoul National University Hospital, Seoul National

University College of Medicine, Seoul, Korea.

FAU - Won, Jae-Kyung

AU - Won JK

AD - Departments of Pathology, Seoul National University Hospital, Seoul National

University College of Medicine, Seoul, Korea.

LA - eng

PT - Journal Article

DEP - 20240514

PL - Korea (South)

TA - J Pathol Transl Med

JT - Journal of pathology and translational medicine

JID - 101650151

PMC - PMC11106608

OTO - NOTNLM

OT - Anaplastic lymphoma kinase

OT - Brain

OT - Epithelioid inflammatory myofibroblastic sarcoma

OT - Inflammatory myofibroblastic tumor

OT - Literature review

COIS- Conflicts of Interest The authors declare that they have no potential conflicts

of interest.

EDAT- 2024/05/20 06:42

MHDA- 2024/05/20 06:43

PMCR- 2024/05/01

CRDT- 2024/05/20 04:30

PHST- 2024/03/09 00:00 [received]

PHST- 2024/04/10 00:00 [accepted]

PHST- 2024/05/20 06:43 [medline]

PHST- 2024/05/20 06:42 [pubmed]

PHST- 2024/05/20 04:30 [entrez]

PHST- 2024/05/01 00:00 [pmc-release]

AID - jptm.2024.04.12 [pii]

AID - jptm-2024-04-12 [pii]

AID - 10.4132/jptm.2024.04.12 [doi]

PST - ppublish

SO - J Pathol Transl Med. 2024 May;58(3):141-145. doi: 10.4132/jptm.2024.04.12. Epub

2024 May 14.

PMID- 38761221

OWN - NLM

STAT- PubMed-not-MEDLINE

LR - 20240521

IS - 2730-6011 (Electronic)

IS - 2730-6011 (Linking)

VI - 15

IP - 1

DP - 2024 May 18

TI - Inflammatory myofibroblastic tumor of the liver after adrenal neuroblastoma

surgery: a case report.

PG - 174

LID - 10.1007/s12672-024-01039-4 [doi]

LID - 174

AB - A boy aged 55 months was diagnosed with stage IV Neuroblastoma (NB) of the right

adrenal gland 2 years ago. Preoperative chemotherapy was given and he was then

treated with retroperitoneal tumor resection and lymph node dissection. After

surgery, the children were transferred to the Hemato-Oncology Department for

chemotherapy according to the high-risk group NB, with outpatient follow-up every

6 months. In the second postoperative year, abdominal computed tomography (CT)

scan revealed a rounded hypodense area in the upper part of the right posterior

lobe of the liver, with marked inhomogeneous enhancement in the venous phase

after enhancement, which was surgically resected, and postoperative pathology

confirmed inflammatory myofibroblastic tumor (IMT) of liver. The patient was not

given any special treatment after surgery. In this study, whole transcriptome

sequencing was performed on the postoperative specimen of adrenal NB and the

specimen of IMT of liver. This unusual case emphasizes the need for close

monitoring of second tumor development in NB survivors even in the absence of

known predisposing factors.

CI - © 2024. The Author(s).

FAU - Shen, Qiyang

AU - Shen Q

AD - Department of Pediatric Surgery, Children's Hospital of Nanjing Medical

University, Nanjing, Jiangsu, China.

FAU - Liu, Xingyu

AU - Liu X

AD - Department of Pediatric Surgery, First Affiliated Hospital of Bengbu Medical

College, Bengbu, Anhui, China.

FAU - Zhang, Lijie

AU - Zhang L

AD - Xuzhou Medical University, Xuzhou, Jiangsu, China.

FAU - Li, Tao

AU - Li T

AD - Department of Pediatric Surgery, Children's Hospital of Nanjing Medical

University, Nanjing, Jiangsu, China. ltcrlcq@yeah.net.

FAU - Zhou, Jianfeng

AU - Zhou J

AD - Department of Pediatric Surgery, Children's Hospital of Nanjing Medical

University, Nanjing, Jiangsu, China. doctorzhoujianfeng@163.com.

LA - eng

PT - Journal Article

DEP - 20240518

PL - United States

TA - Discov Oncol

JT - Discover oncology

JID - 101775142

PMC - PMC11102410

OTO - NOTNLM

OT - ICG

OT - Inflammatory myofibroblastic tumor

OT - Neuroblastoma

OT - Secondary tumor

OT - Whole transcriptome sequencing

COIS- The authors declare that the research was conducted in the absence of any

commercial or financial relationships that could be construed as a potential

competing interests.

EDAT- 2024/05/18 19:47

MHDA- 2024/05/18 19:48

PMCR- 2024/05/18

CRDT- 2024/05/18 11:04

PHST- 2023/09/30 00:00 [received]

PHST- 2024/05/15 00:00 [accepted]

PHST- 2024/05/18 19:48 [medline]

PHST- 2024/05/18 19:47 [pubmed]

PHST- 2024/05/18 11:04 [entrez]

PHST- 2024/05/18 00:00 [pmc-release]

AID - 10.1007/s12672-024-01039-4 [pii]

AID - 1039 [pii]

AID - 10.1007/s12672-024-01039-4 [doi]

PST - epublish

SO - Discov Oncol. 2024 May 18;15(1):174. doi: 10.1007/s12672-024-01039-4.

PMID- 38752043

OWN - NLM

STAT- PubMed-not-MEDLINE

LR - 20240517

IS - 2168-8184 (Print)

IS - 2168-8184 (Electronic)

IS - 2168-8184 (Linking)

VI - 16

IP - 4

DP - 2024 Apr

TI - Endobronchial Inflammatory Myofibroblastic Tumour Masquerading as a Ruptured

Hydatid Cyst.

PG - e58283

LID - 10.7759/cureus.58283 [doi]

LID - e58283

AB - Inflammatory myofibroblastic tumours (IMTs) represent a rare group of neoplastic

lesions characterized by a diverse clinical presentation. Endobronchial

involvement is infrequently reported, and its manifestation mimicking the

symptoms of a ruptured hydatid cyst adds an additional layer of complexity to the

diagnostic challenge. This case report delves into an exceptional clinical

scenario where an endobronchial IMT masqueraded as a ruptured hydatid cyst,

initially confounding the diagnostic team. Through a detailed examination of the

patient's clinical history, radiological imaging, bronchoscopy findings and

subsequent histopathological analysis, we aim to contribute to the existing

medical literature and shed light on the nuances encountered in accurately

identifying and differentiating these two entities.

CI - Copyright © 2024, S et al.

FAU - S, Shiva

AU - S S

AD - Department of General Surgery, King George's Medical University, Lucknow, IND.

FAU - Kumar, Suresh

AU - Kumar S

AD - Department of General Surgery, King George's Medical University, Lucknow, IND.

FAU - Singh, Pankaj

AU - Singh P

AD - Department of General Surgery, King George's Medical University, Lucknow, IND.

FAU - Kumar, Sanjeev

AU - Kumar S

AD - Department of General Surgery, King George's Medical University, Lucknow, IND.

FAU - Agrawal, Vinita

AU - Agrawal V

AD - Department of Pathology, Sanjay Gandhi Postgraduate Institute of Medical Sciences

(SGPGIMS), Lucknow, IND.

LA - eng

PT - Case Reports

PT - Journal Article

DEP - 20240415

PL - United States

TA - Cureus

JT - Cureus

JID - 101596737

PMC - PMC11094534

OTO - NOTNLM

OT - alk rearrangement

OT - complex hydatid cyst

OT - endobronchial tumor

OT - inflammatory myofibroblastic tumor

OT - pulmonary tumors

COIS- The authors have declared that no competing interests exist.

EDAT- 2024/05/16 06:42

MHDA- 2024/05/16 06:43

PMCR- 2024/04/15

CRDT- 2024/05/16 03:53

PHST- 2024/04/15 00:00 [accepted]

PHST- 2024/05/16 06:43 [medline]

PHST- 2024/05/16 06:42 [pubmed]

PHST- 2024/05/16 03:53 [entrez]

PHST- 2024/04/15 00:00 [pmc-release]

AID - 10.7759/cureus.58283 [doi]

PST - epublish

SO - Cureus. 2024 Apr 15;16(4):e58283. doi: 10.7759/cureus.58283. eCollection 2024

Apr.

PMID- 38745658

OWN - NLM

STAT- MEDLINE

DCOM- 20240515

LR - 20240516

IS - 1664-3224 (Electronic)

IS - 1664-3224 (Linking)

VI - 15

DP - 2024

TI - A case report of IgG4-related hepatic inflammatory pseudotumor in a 3-year old

boy.

PG - 1376276

LID - 10.3389/fimmu.2024.1376276 [doi]

LID - 1376276

AB - BACKGROUND: Hepatic Inflammatory Pseudotumor (IPT) is an infrequent condition

often masquerading as a malignant tumor, resulting in misdiagnosis and

unnecessary surgical resection. The emerging concept of IgG4-related diseases

(IgG4-RD) has gained widespread recognition, encompassing entities like

IgG4-related hepatic IPT. Clinically and radiologically, corticosteroids and

immunosuppressive therapies have proven effective in managing this condition.

CASE PRESENTATION: A 3-year-old Chinese boy presented to the clinic with an

11-month history of anemia, fever of unknown origin, and a tender hepatic mass.

Blood examinations revealed chronic anemia (Hb: 6.4 g/L, MCV: 68.6 fl, MCH: 19.5

pg, reticulocytes: 1.7%) accompanied by an inflammatory reaction and an elevated

serum IgG4 level (1542.2 mg/L). Abdominal contrast-enhanced computed tomography

unveiled a 7.6 cm low-density mass in the right lateral lobe, while magnetic

resonance imaging demonstrated slight hypointensity on T1-weighted images and

slight hyperintensity on T2-weighted images, prompting suspicion of hepatic

malignancy. A subsequent liver biopsy revealed a mass characterized by fibrous

stroma and dense lymphoplasmacytic infiltration. Immunohistochemical analysis

confirmed the presence of IgG4-positive plasma cells, leading to the diagnosis of

IgG4-related hepatic IPT. Swift resolution occurred upon initiation of

corticosteroid and mycophenolate mofetil therapies. CONCLUSION: This study

underscores the diagnostic approach to hepatic IPT, utilizing histopathology,

immunostaining, imaging, serology, organ involvement, and therapeutic response.

Early histological examination plays a pivotal role in clinical guidance,

averting misdiagnosis as a liver tumor and unnecessary surgical interventions.

CI - Copyright © 2024 Wan, Xu, Liu, Wu, Zhong and Wu.

FAU - Wan, Qian

AU - Wan Q

AD - Department of Hematology, Jiangxi Provincial Children's Hospital, Nanchang,

China.

FAU - Xu, Zhongjin

AU - Xu Z

AD - Department of Hematology, Jiangxi Provincial Children's Hospital, Nanchang,

China.

FAU - Liu, Xiaohui

AU - Liu X

AD - Department of Rheumatology and Immunology, Jiangxi Provincial Children's

Hospital, Nanchang, China.

FAU - Wu, Zhuqiang

AU - Wu Z

AD - Nuclear Magnetic Resonance Room, Jiangxi Provincial Children's Hospital,

Nanchang, China.

FAU - Zhong, Qingmei

AU - Zhong Q

AD - Department of Pathology, The Ninth Hospital of Nanchang, Nanchang, China.

FAU - Wu, Chongjun

AU - Wu C

AD - Department of Hematology, Jiangxi Provincial Children's Hospital, Nanchang,

China.

LA - eng

PT - Case Reports

PT - Journal Article

DEP - 20240430

PL - Switzerland

TA - Front Immunol

JT - Frontiers in immunology

JID - 101560960

SB - IM

MH - Humans

MH - Male

MH - *Granuloma, Plasma Cell/diagnosis/immunology/drug therapy

MH - Child, Preschool

MH - *Immunoglobulin G/blood/immunology

MH - *Immunoglobulin G4-Related Disease/diagnosis

MH - Liver Diseases/diagnosis/immunology

MH - Diagnosis, Differential

MH - Liver/pathology/diagnostic imaging/immunology

MH - Tomography, X-Ray Computed

MH - Biopsy

MH - Immunosuppressive Agents/therapeutic use

PMC - PMC11091244

OTO - NOTNLM

OT - IgG4-related disease

OT - child

OT - hepatic inflammatory pseudotumor

OT - liver biopsy

OT - steroid

COIS- The authors declare that the research was conducted in the absence of any

commercial or financial relationships that could be construed as a potential

conflict of interest.

EDAT- 2024/05/15 06:42

MHDA- 2024/05/15 06:43

PMCR- 2024/01/01

CRDT- 2024/05/15 03:46

PHST- 2024/01/25 00:00 [received]

PHST- 2024/04/19 00:00 [accepted]

PHST- 2024/05/15 06:43 [medline]

PHST- 2024/05/15 06:42 [pubmed]

PHST- 2024/05/15 03:46 [entrez]

PHST- 2024/01/01 00:00 [pmc-release]

AID - 10.3389/fimmu.2024.1376276 [doi]

PST - epublish

SO - Front Immunol. 2024 Apr 30;15:1376276. doi: 10.3389/fimmu.2024.1376276.

eCollection 2024.

PMID- 38717131

OWN - NLM

STAT- MEDLINE

DCOM- 20240717

LR - 20240717

IS - 1532-0979 (Electronic)

IS - 0147-5185 (Linking)

VI - 48

IP - 8

DP - 2024 Aug 1

TI - Myxoid Inflammatory Myofibroblastic Sarcoma: Clinicopathologic Analysis of 25

Cases of a Distinctive Sarcoma With Deceptively Bland Morphology and Aggressive

Clinical Behavior.

PG - 1005-1016

LID - 10.1097/PAS.0000000000002231 [doi]

AB - The number of recognized sarcoma types harboring targetable molecular alterations

continues to increase. Here we present 25 examples of a distinctive

myofibroblastic tumor, provisionally termed "myxoid inflammatory myofibroblastic

sarcoma," which might be related to inflammatory myofibroblastic tumor, and which

occurred in 13 males (52%) and 12 females at a median age of 37 years (range: 7

to 79 years). Primary tumor sites were peritoneum (18 patients; 72%),

paratesticular (2; 8%), chest wall (1), upper extremity (1), esophagus (1),

retroperitoneum (1), and uterus (1). Nine peritoneal tumors (50%) were multifocal

at presentation; all other tumors were unifocal. Tumors showed

bland-to-mildly-atypical neoplastic myofibroblasts in a myxoid stroma, with

prominent inflammatory infiltrates in 22 cases (88%). Most tumors showed delicate

branching stromal vessels like those of myxoid liposarcoma, and most showed

infiltrative growth through non-neoplastic tissue. Immunohistochemistry

demonstrated expression of SMA (19/25 tumors; 76%), desmin (13/22; 59%), and CD30

(5/11; 45%), while ALK was expressed in 1 tumor (of 25; 4%) that was negative for

ALK rearrangement. Sequencing of 11 tumors showed seven to harbor tyrosine kinase

fusions (4 PDGFRB , 2 PML :: JAK1 , 1 SEC31A :: PDGFRA ). Two instead harbored

hot spot KRAS mutations (G12V and Q61H), and 2 were negative for known driving

alterations. Clinical follow-up was available for 18 patients (72%; median: 2.7

years; range: 4 mo-12.3 years). Nine patients (50%) were alive with no evidence

of disease, 5 (28%) died of disease, and 4 (22%) were alive with disease. Seven

patients (39%) experienced peritoneal relapse or distant metastasis. Two patients

showed disease progression on conventional, nontargeted chemotherapy. The patient

whose tumor harbored SEC31A :: PDGFRA was treated after multiple relapses with

imatinib and sunitinib therapy, with progression-free periods of 5 and 2 years,

respectively. Despite its bland appearance, myxoid inflammatory myofibroblastic

sarcoma harbors a significant risk for disseminated disease, particularly when it

occurs in the peritoneum. Targeted therapy could be considered for patients with

disseminated disease.

CI - Copyright © 2024 Wolters Kluwer Health, Inc. All rights reserved.

FAU - Papke, David J Jr

AU - Papke DJ Jr

AD - Department of Pathology, Brigham and Women's Hospital, and Harvard Medical

School, Boston, MA.

FAU - Odintsov, Igor

AU - Odintsov I

AD - Department of Pathology, Brigham and Women's Hospital, and Harvard Medical

School, Boston, MA.

FAU - Dickson, Brendan C

AU - Dickson BC

AD - Department of Pathology & Laboratory Medicine, Mount Sinai Hospital.

FAU - Nucci, Marisa R

AU - Nucci MR

AD - Department of Pathology, Brigham and Women's Hospital, and Harvard Medical

School, Boston, MA.

FAU - Agaimy, Abbas

AU - Agaimy A

AD - Institute of Pathology, Erlangen University Hospital, Friedrich Alexander

University of Erlangen-Nuremberg, Erlangen, Germany.

FAU - Fletcher, Christopher D M

AU - Fletcher CDM

AD - Department of Pathology, Brigham and Women's Hospital, and Harvard Medical

School, Boston, MA.

LA - eng

PT - Journal Article

DEP - 20240508

PL - United States

TA - Am J Surg Pathol

JT - The American journal of surgical pathology

JID - 7707904

RN - 0 (Biomarkers, Tumor)

SB - IM

MH - Humans

MH - Male

MH - Female

MH - Adult

MH - Middle Aged

MH - Aged

MH - Adolescent

MH - Young Adult

MH - Child

MH - *Biomarkers, Tumor/genetics/analysis

MH - *Myofibroblasts/pathology/chemistry

MH - Soft Tissue Neoplasms/pathology/genetics/mortality/therapy

MH - Sarcoma/pathology/genetics/chemistry/mortality

MH - Immunohistochemistry

COIS- Conflicts of Interest and Source of Funding: The authors have disclosed that they

have no significant relationships with, or financial interest in, any commercial

companies pertaining to this article.

EDAT- 2024/05/08 12:44

MHDA- 2024/07/17 12:44

CRDT- 2024/05/08 08:53

PHST- 2024/07/17 12:44 [medline]

PHST- 2024/05/08 12:44 [pubmed]

PHST- 2024/05/08 08:53 [entrez]

AID - 00000478-990000000-00341 [pii]

AID - 10.1097/PAS.0000000000002231 [doi]

PST - ppublish

SO - Am J Surg Pathol. 2024 Aug 1;48(8):1005-1016. doi: 10.1097/PAS.0000000000002231.

Epub 2024 May 8.

PMID- 38713995

OWN - NLM

STAT- MEDLINE

DCOM- 20240701

LR - 20240702

IS - 1532-2157 (Electronic)

IS - 0748-7983 (Linking)

VI - 50

IP - 7

DP - 2024 Jul

TI - Treatment and outcomes in pediatric inflammatory myofibroblastic tumors - A

systematic review of published studies.

PG - 108388

LID - S0748-7983(24)00440-2 [pii]

LID - 10.1016/j.ejso.2024.108388 [doi]

AB - Inflammatory myofibroblastic tumor (IMT) is a soft tissue neoplasm which can be

locally invasive, recur, or in rare cases metastasize. Often originating from the

abdomen or thorax, IMT most commonly affects children and young adults. Due to

its rarity comprehensive reports detailing clinical management and outcome(s) are

sparse and often based on limited index case numbers. This study systematically

analyzes outcome metrics of pediatric IMT and identifies risk factors for

mortality. Medline/Embase databases were searched in accordance with PRISMA

guidelines. Final analysis included 57 studies with 673 IMT patients (355 males,

53 %). Individual patient data was available for 405 cases with a median

follow-up period of 36 months. Tumor sites included abdomen/pelvis (n = 233,

58 %), thorax (n = 125, 31 %), head/neck (n = 34, 8 %), and extremities (n = 13,

3 %). Surgical tumor resection was the mainstay of treatment, while only 20

patients (5 %) were treated non-operatively. Recurrence(s) were reported in 80

patients (20 %) with 34 (12 %) requiring reoperation. Positive tumor margins were

a significant risk factor for tumor recurrence (p < 0.0001). Chemo/radiotherapy

was reported in 98 patients (25 %). Most patients (94 %) survived; 81 % (n = 237)

with no evidence of recurrent disease, 14 % (n = 41) were alive with disease, and

25 (6 %) died of disease. Positive margins at primary operation, and metastatic

disease were associated with mortality (p < 0.0001 for both). IMT is a rare tumor

with favorable outcome for the majority of patients. Whilst most patients will

present with benign tumors, complete surgical resection (R0) is crucial, as

positive surgical margins are a significant risk factor for tumor recurrence and

mortality.

CI - © 2024 Elsevier Ltd, BASO ∼ The Association for Cancer Surgery, and the European

Society of Surgical Oncology. All rights reserved.

FAU - Raitio, Arimatias

AU - Raitio A

AD - University of Turku and Turku University Hospital, Paediatric Surgery, Turku,

Finland.

FAU - Losty, Paul D

AU - Losty PD

AD - Institute of Systems Molecular and Integrative Biology, University of Liverpool,

Liverpool, UK; Department of Paediatric Surgery, Ramathibodi Hospital, Mahidol

University, Bangkok, Thailand. Electronic address: paul.losty@liverpool.ac.uk.

LA - eng

PT - Journal Article

PT - Review

PT - Systematic Review

DEP - 20240503

PL - England

TA - Eur J Surg Oncol

JT - European journal of surgical oncology : the journal of the European Society of

Surgical Oncology and the British Association of Surgical Oncology

JID - 8504356

SB - IM

MH - Humans

MH - Child

MH - *Neoplasm Recurrence, Local

MH - Margins of Excision

MH - Granuloma, Plasma Cell/therapy/pathology/surgery

MH - Risk Factors

MH - Abdominal Neoplasms/therapy/pathology

MH - Head and Neck Neoplasms/therapy/pathology/mortality/surgery

MH - Thoracic Neoplasms/therapy/pathology/mortality

MH - Soft Tissue Neoplasms/therapy/pathology/mortality

MH - Reoperation

MH - Neoplasms, Muscle Tissue/therapy/pathology

OTO - NOTNLM

OT - Inflammatory myofibroblastic tumor

OT - Outcome(s)

OT - Pediatric

OT - Plasma cell granuloma

COIS- Declaration of competing interest Dr Raitio reports research grants from Päivikki

and Sakari Sohlberg Foundation. Funder had no role in the current study.

EDAT- 2024/05/08 02:06

MHDA- 2024/07/02 00:42

CRDT- 2024/05/07 18:03

PHST- 2024/04/17 00:00 [received]

PHST- 2024/04/28 00:00 [revised]

PHST- 2024/05/02 00:00 [accepted]

PHST- 2024/07/02 00:42 [medline]

PHST- 2024/05/08 02:06 [pubmed]

PHST- 2024/05/07 18:03 [entrez]

AID - S0748-7983(24)00440-2 [pii]

AID - 10.1016/j.ejso.2024.108388 [doi]

PST - ppublish

SO - Eur J Surg Oncol. 2024 Jul;50(7):108388. doi: 10.1016/j.ejso.2024.108388. Epub

2024 May 3.

PMID- 38707731

OWN - NLM

STAT- PubMed-not-MEDLINE

LR - 20240507

IS - 2732-7787 (Electronic)

IS - 2732-7787 (Linking)

VI - 4

IP - 3

DP - 2024 May-Jun

TI - A Rare Inflammatory Myofibroblastic Tumor of the Spleen: A Case Report.

PG - 379-383

LID - 10.21873/cdp.10335 [doi]

AB - BACKGROUND/AIM: Inflammatory myofibroblastic tumors (IMTs) are rare, solid,

potentially malignant lesions of uncertain etiology. Histologically, IMTs exhibit

a combination of lymphocytes and inflammatory cells within a fibroblastic myxoid

layer. The diagnosis of IMTs poses a challenge for various medical specialties,

including surgeons, pathologists, and oncologists, due to their non-specific

clinical presentation. Furthermore, radiologists face difficulties in

interpreting computed tomography (CT) or magnetic resonance imaging (MRI)

results, which often yield polymorphic and inconclusive findings. Ultimately,

histopathologists play a crucial role in reaching a definitive diagnosis based on

the tumor's histological characteristics. They are detected in every system of

the human body, most commonly in the lungs. Here, we report an uncommon

occurrence of IMT in the spleen of a patient with nonspecific abdominal pain.

CASE REPORT: A 56-year-old Caucasian female presented to Konstantopouleio General

Hospital of Nea Ionia, Athens, Greece, with abdominal pain and discomfort. The

patient had no significant medical history and normal laboratory tests. An

abdominal CT revealed a large mass in the spleen. A splenectomy was performed.

Histopathological analysis of the tumor revealed IMTS. CONCLUSION: Splenic IMT is

a rare benign tumor with moderate malignant potential. It lacks a distinct

clinical presentation and is typically identified either incidentally or during

the examination of abdominal pain.

CI - Copyright 2024, International Institute of Anticancer Research.

FAU - Fagkrezos, Dimitris

AU - Fagkrezos D

AD - Computed Tomography Department, Konstantopouleio General Hospital, Athens,

Greece.

FAU - Kakavelou, Marina

AU - Kakavelou M

AD - Department of Anatomy, National and Kapodistrian University of Athens, Athens,

Greece.

FAU - Charitaki, Evgenia

AU - Charitaki E

AD - Surgery Department, Konstantopouleio General Hospital, Athens, Greece.

FAU - Delis, Spiros

AU - Delis S

AD - Surgery Department, Konstantopouleio General Hospital, Athens, Greece.

FAU - Papaparaskeva, Kleo

AU - Papaparaskeva K

AD - Pathology Department, Konstantopouleio General Hospital, Athens, Greece.

FAU - Triantopoulou, Charikleia

AU - Triantopoulou C

AD - Radiology Department, Konstantopouleio General Hospital, Athens, Greece.

FAU - Maniatis, Petros

AU - Maniatis P

AD - Computed Tomography Department, Konstantopouleio General Hospital, Athens,

Greece.

FAU - Chrysikos, Dimosthenis

AU - Chrysikos D

AD - Department of Anatomy, National and Kapodistrian University of Athens, Athens,

Greece.

FAU - Troupis, Theodore

AU - Troupis T

AD - Department of Anatomy, National and Kapodistrian University of Athens, Athens,

Greece.

LA - eng

PT - Journal Article

DEP - 20240503

PL - Greece

TA - Cancer Diagn Progn

JT - Cancer diagnosis & prognosis

JID - 9918316186306676

PMC - PMC11062170

OTO - NOTNLM

OT - Spleen

OT - inflammatory myofibroblastic tumor

OT - nonspecific abdominal pain

OT - oncology

OT - pseudotumor

COIS- The Authors assert that they do not have any conflicting interests in relation to

this study.

EDAT- 2024/05/06 06:43

MHDA- 2024/05/06 06:44

PMCR- 2024/05/03

CRDT- 2024/05/06 04:16

PHST- 2024/02/08 00:00 [received]

PHST- 2024/03/07 00:00 [accepted]

PHST- 2024/05/06 06:44 [medline]

PHST- 2024/05/06 06:43 [pubmed]

PHST- 2024/05/06 04:16 [entrez]

PHST- 2024/05/03 00:00 [pmc-release]

AID - 10.21873/cdp.10335 [doi]

PST - epublish

SO - Cancer Diagn Progn. 2024 May 3;4(3):379-383. doi: 10.21873/cdp.10335. eCollection

2024 May-Jun.

PMID- 31869057

STAT- Publisher

CTDT- 20240506

PB - StatPearls Publishing

DP - 2025 Jan

TI - Nonspecific Orbital Inflammation.

BTI - StatPearls

AB - Orbital pseudotumor, also known as orbital inflammatory pseudotumors (OIP),

idiopathic orbital inflammation (IOI), idiopathic orbital inflammatory syndrome

(IOIS), idiopathic orbital inflammatory pseudotumor (IOIP), or nonspecific

orbital inflammation (NSOI) is a benign, space-occupying, and noninfectious

inflammatory condition of the orbit but may extend in the periorbital area. No

identifiable infectious, systemic, or neoplastic disorder is associated with it.

It is the third most common orbital disease in adults, following thyroid

orbitopathy and lymphoproliferative diseases. Many orbital inflammations may be

associated with systemic conditions or remote organ dysfunction. Categories of

orbital pseudotumor according to location include anterior, diffuse, posterior,

or apical. Other classifications include myositis, dacryoadenitis, periscleritis,

perineuritis, and focal mass. Orbital pseudotumor is rare in children. The most

common ophthalmic findings include periorbital edema and blepharoptosis. A

palpable mass may be present. On orbital radiography, common findings are

dacryoadenitis, orbital mass, or myositis. In children, systemic signs are

present in up to 50% of patients. Headache, emesis, anorexia, lethargy, and fever

are the most common systemic signs. Additionally, there are likely associations

with iritis, uveitis, disc edema, and peripheral eosinophilia.  The

pathophysiology of orbital pseudotumor, first identified more than a century ago,

is still unknown, adding to the difficulties in diagnosis and uncertainty

surrounding treatment. However, advances in imaging, histology, and immunology

have illuminated the underlying mechanisms of the illness, previously believed to

be essentially idiopathic. These mechanisms involve a complex interaction of

immunological dysregulation, viral triggers, and genetic predisposition.

Understanding the clinical spectrum and diagnostic criteria of orbital

pseudotumor is essential for accurate diagnosis and effective treatment. Patients

commonly present with unilateral proptosis, discomfort, diplopia, and visual

abnormalities, which can be either acute or subacute. These symptoms often

resemble other orbital diseases such as neoplasms, infections, or thyroid eye

disease. Clinical assessment, imaging modalities (such as computed tomography

[CT] and magnetic resonance imaging [MRI]), and occasionally histological

examination acquired via biopsy are utilized to differentiate orbital pseudotumor

from these conditions.

CI - Copyright © 2025, StatPearls Publishing LLC.

FAU - Ronquillo, Yasmyne

AU - Ronquillo Y

AD - Hoopes Vision Research Center

FAU - Zeppieri, Marco

AU - Zeppieri M

AD - University Hospital of Udine, Italy

FAU - Patel, Bhupendra C

AU - Patel BC

AD - University of Utah

LA - eng

PT - Study Guide

PT - Book Chapter

PL - Treasure Island (FL)

COIS- Disclosure: Yasmyne Ronquillo declares no relevant financial relationships with

ineligible companies. Disclosure: Marco Zeppieri declares no relevant financial

relationships with ineligible companies. Disclosure: Bhupendra Patel declares no

relevant financial relationships with ineligible companies.

EDAT- 2024/05/06 00:00

CRDT- 2024/05/06 00:00

AID - NBK551576 [bookaccession]

PMID- 38693763

OWN - NLM

STAT- MEDLINE

DCOM- 20241121

LR - 20241125

IS - 1816-5370 (Electronic)

IS - 0218-4923 (Linking)

VI - 32

IP - 6-7

DP - 2024 Sep

TI - Surgically challenging inflammatory myofibroblastic tumor: A rare neoplasm of

lung.

PG - 421-424

LID - 10.1177/02184923241248681 [doi]

AB - Inflammatory myofibroblastic tumor is considered one of the rarest benign tumors

constituting 0.7% of all lung neoplasms. It was first described in 1939. We

report a case of a 10-year-old child who presented with recurrent cough and

fever. Chest radiography and computed tomography demonstrated complete

involvement of right lung by the tumor. The tumor along with the affected lung

was meticulously dissected from the surrounding structures and was delivered

outside. The histopathology of the specimen revealed it to be inflammatory

fibroblastoma.

FAU - Chakraborty, Nirupam Sekhar

AU - Chakraborty NS

AUID- ORCID: 0000-0003-1036-0125

AD - Department of Cardiothoracic and Vascular Surgery, All India Institute of Medical

Science, Raipur, India. RINGGOLD: 417408

FAU - Saurav, Gaind Kumar

AU - Saurav GK

AD - Department of Cardiothoracic and Vascular Surgery, All India Institute of Medical

Science, Raipur, India. RINGGOLD: 417408

FAU - Kashyap, Nitin

AU - Kashyap N

AUID- ORCID: 0000-0001-5696-1837

AD - Department of Cardiothoracic and Vascular Surgery, All India Institute of Medical

Science, Raipur, India. RINGGOLD: 417408

FAU - Suresh, Pranay Mehsare

AU - Suresh PM

AUID- ORCID: 0000-0003-2644-3187

AD - Department of Cardiothoracic and Vascular Surgery, All India Institute of Medical

Science, Raipur, India. RINGGOLD: 417408

FAU - Borkar, Nitin

AU - Borkar N

AD - Department of Pediatric Surgery, All India Institute of Medical Science, Raipur,

India. RINGGOLD: 417408

FAU - Gupta, Rakesh

AU - Gupta R

AD - Department of Pathology, All India Institute of Medical Science, Raipur, India.

RINGGOLD: 417408

LA - eng

PT - Case Reports

PT - Journal Article

DEP - 20240501

PL - England

TA - Asian Cardiovasc Thorac Ann

JT - Asian cardiovascular & thoracic annals

JID - 9503417

SB - IM

MH - Child

MH - Humans

MH - Biopsy

MH - *Lung Neoplasms/surgery/pathology/diagnostic imaging

MH - Neoplasms, Muscle Tissue/surgery/pathology/diagnostic imaging

MH - *Plasma Cell Granuloma, Pulmonary/surgery/diagnostic imaging/pathology

MH - *Pneumonectomy

MH - *Tomography, X-Ray Computed

MH - Treatment Outcome

MH - Female

OTO - NOTNLM

OT - Inflammatory myofibroblastoma

OT - lung

OT - rare tumor

OT - thoracotomy

COIS- Declaration of conflicting interestsThe author(s) declared no potential conflicts

of interest with respect to the research, authorship, and/or publication of this

article.

EDAT- 2024/05/02 06:42

MHDA- 2024/11/21 06:20

CRDT- 2024/05/02 02:33

PHST- 2024/11/21 06:20 [medline]

PHST- 2024/05/02 06:42 [pubmed]

PHST- 2024/05/02 02:33 [entrez]

AID - 10.1177/02184923241248681 [doi]

PST - ppublish

SO - Asian Cardiovasc Thorac Ann. 2024 Sep;32(6-7):421-424. doi:

10.1177/02184923241248681. Epub 2024 May 1.

PMID- 38686080

OWN - NLM

STAT- PubMed-not-MEDLINE

LR - 20240501

IS - 2577-171X (Electronic)

IS - 2577-171X (Linking)

VI - 7

IP - 3

DP - 2024 May

TI - A diagnostically challenging case of inflammatory myofibroblastic tumor primary

to the peritoneum.

PG - 206-209

LID - 10.1002/iju5.12701 [doi]

AB - INTRODUCTION: Inflammatory myofibroblastic tumors are difficult to diagnose

because of the lack of specific indicators. We describe a diagnostically

challenging case of an inflammatory myofibroblastic tumor primary to the

peritoneum. CASE PRESENTATION: The patient was a 25-year-old male who presented

at our hospital with lower abdominal pain. Computed tomography revealed a mass

lesion 80 mm in diameter just above the bladder. This was suspected to be a

bleeding tumor of the urachus. Since malignancy could not be ruled out, surgery

was planned. This revealed a fragile tumor arising from the peritoneum. Following

its removal, the tumor was diagnosed by histopathological analysis as an

inflammatory myofibroblastic tumor. CONCLUSION: We describe a case of

inflammatory myofibroblastic tumor primary to the peritoneum diagnosed by

histopathology. Inflammatory myofibroblastic tumor should be considered in the

differential diagnosis of abdominal wall and anterior bladder tumors.

CI - © 2024 The Authors. IJU Case Reports published by John Wiley & Sons Australia,

Ltd on behalf of Japanese Urological Association.

FAU - Karibe, Jurii

AU - Karibe J

AUID- ORCID: 0000-0001-8146-2861

AD - Department of Urology and Renal Transplantation Yokohama City University Medical

Center Yokohama Japan.

FAU - Teranishi, Jun-Ichi

AU - Teranishi JI

AD - Department of Urology and Renal Transplantation Yokohama City University Medical

Center Yokohama Japan.

FAU - Kawahara, Takashi

AU - Kawahara T

AUID- ORCID: 0000-0002-7049-3379

AD - Department of Urology and Renal Transplantation Yokohama City University Medical

Center Yokohama Japan.

FAU - Noguchi, Takeaki

AU - Noguchi T

AUID- ORCID: 0009-0006-5781-260X

AD - Department of Urology and Renal Transplantation Yokohama City University Medical

Center Yokohama Japan.

FAU - Takeshima, Teppei

AU - Takeshima T

AUID- ORCID: 0000-0003-2733-5487

AD - Department of Urology and Renal Transplantation Yokohama City University Medical

Center Yokohama Japan.

FAU - Osaka, Kimito

AU - Osaka K

AD - Department of Urology and Renal Transplantation Yokohama City University Medical

Center Yokohama Japan.

FAU - Kumagai, Eita

AU - Kumagai E

AD - Department of Pathology Yokohama City University Medical Center Yokohama Japan.

FAU - Sawazumi, Tomoe

AU - Sawazumi T

AD - Department of Pathology Yokohama City University Medical Center Yokohama Japan.

FAU - Fujii, Satoshi

AU - Fujii S

AD - Department of Pathology Yokohama City University Medical Center Yokohama Japan.

AD - Department of Pathology Yokohama City University Hospital Yokohama Japan.

AD - Department of Molecular Pathology Yokohama City University Graduate School of

Medicine Yokohama Japan.

FAU - Uemura, Hiroji

AU - Uemura H

AD - Department of Urology and Renal Transplantation Yokohama City University Medical

Center Yokohama Japan.

LA - eng

PT - Case Reports

PT - Journal Article

DEP - 20240214

PL - Australia

TA - IJU Case Rep

JT - IJU case reports

JID - 101764958

PMC - PMC11056253

OTO - NOTNLM

OT - abdominal wall

OT - anaplastic lymphoma kinase

OT - histopathological analysis

OT - inflammatory myofibroblastic tumor

OT - peritoneum

COIS- The authors declare no conflict of interest.

EDAT- 2024/04/30 06:46

MHDA- 2024/04/30 06:47

PMCR- 2024/02/14

CRDT- 2024/04/30 03:37

PHST- 2023/09/12 00:00 [received]

PHST- 2024/01/24 00:00 [accepted]

PHST- 2024/04/30 06:47 [medline]

PHST- 2024/04/30 06:46 [pubmed]

PHST- 2024/04/30 03:37 [entrez]

PHST- 2024/02/14 00:00 [pmc-release]

AID - IJU512701 [pii]

AID - 10.1002/iju5.12701 [doi]

PST - epublish

SO - IJU Case Rep. 2024 Feb 14;7(3):206-209. doi: 10.1002/iju5.12701. eCollection 2024

May.

PMID- 38676786

OWN - NLM

STAT- PubMed-not-MEDLINE

LR - 20241021

IS - 2366-1089 (Electronic)

IS - 2366-1070 (Print)

IS - 2366-1089 (Linking)

VI - 12

IP - 2

DP - 2024 Jun

TI - Podcast on Emerging Treatment Options for Pediatric Patients with ALK-Positive

Anaplastic Large Cell Lymphoma and Inflammatory Myofibroblastic Tumors.

PG - 247-255

LID - 10.1007/s40487-024-00275-6 [doi]

AB - Anaplastic large cell lymphoma (ALCL) and inflammatory myofibroblastic tumor

(IMT) are rare cancers observed predominantly in children and young adults. ALCL

accounts for 10-15% of all pediatric non-Hodgkin lymphomas and is commonly

diagnosed at an advanced stage of disease. In children, 84-91% of cases of ALCL

harbor an anaplastic lymphoma kinase (ALK) gene translocation. IMT is a rare

mesenchymal neoplasm that also tends to occur in children and adolescents.

Approximately 50-70% of IMT cases involve rearrangements in the ALK gene. A

combination of chemotherapeutic drugs is typically used for children with

ALK-positive ALCL, and the only known curative therapy for ALK-positive IMT is

complete surgical resection. Crizotinib, a first-generation ALK inhibitor, was

approved in the USA in 2021 for pediatric patients and young adults with relapsed

or refractory ALK-positive ALCL; however, its safety and efficacy have not been

established in older adults. In 2022, crizotinib was approved for adult and

pediatric patients with unresectable, recurrent, or refractory ALK-positive IMT.

This podcast provides an overview of ALK-positive ALCL and IMT. We discuss the

current treatment landscape, the role of ALK tyrosine kinase inhibitors, and

areas of future research.

CI - © 2024. The Author(s).

FAU - Lowe, Eric

AU - Lowe E

AD - Children's Hospital of The King's Daughters, Norfolk, VA, USA.

FAU - Mossé, Yael P

AU - Mossé YP

AD - Division of Oncology and Center for Childhood Cancer Research, Children's

Hospital of Philadelphia, Perelman School of Medicine at the University of

Pennsylvania, Philadelphia, PA, USA. mosse@chop.edu.

LA - eng

PT - Journal Article

DEP - 20240427

PL - New Zealand

TA - Oncol Ther

JT - Oncology and therapy

JID - 101677510

PMC - PMC11187053

OTO - NOTNLM

OT - ALK-positive NSCLC

OT - Anaplastic large cell lymphoma

OT - Inflammatory myofibroblastic tumors

COIS- Eric Lowe participated on the data safety monitoring board for the European study

of ALK inhibitors. Yael P Mossé reports consulting fees from Pfizer, served as a

principal investigator for the COG ADVL0912 trial, received crizotinib and

lorlatinib via an MTA for preclinical work in the laboratory, and is a member of

the ASCO TAPUR DSMB.

EDAT- 2024/04/28 07:23

MHDA- 2024/04/28 07:24

PMCR- 2024/04/27

CRDT- 2024/04/27 11:06

PHST- 2023/10/05 00:00 [received]

PHST- 2024/03/25 00:00 [accepted]

PHST- 2024/04/28 07:24 [medline]

PHST- 2024/04/28 07:23 [pubmed]

PHST- 2024/04/27 11:06 [entrez]

PHST- 2024/04/27 00:00 [pmc-release]

AID - 10.1007/s40487-024-00275-6 [pii]

AID - 275 [pii]

AID - 10.1007/s40487-024-00275-6 [doi]

PST - ppublish

SO - Oncol Ther. 2024 Jun;12(2):247-255. doi: 10.1007/s40487-024-00275-6. Epub 2024

Apr 27.

PMID- 38656257

OWN - NLM

STAT- Publisher

LR - 20240424

IS - 1940-2465 (Electronic)

IS - 1066-8969 (Linking)

DP - 2024 Apr 24

TI - Inflammatory Myofibroblastic Tumor of the Esophagus and Stomach Successfully

Treated With ALK Inhibitor in a Pediatric Patient: A Case Report and Concise

Review of Literature.

PG - 10668969241246470

LID - 10.1177/10668969241246470 [doi]

AB - An inflammatory myofibroblastic tumor (IMT) is a rare mesenchymal neoplasm of

borderline malignant potential. Nearly half of all IMTs have rearrangement of

anaplastic lymphoma kinase (ALK) locus on chromosome 2p23 which can be treated

with targeted therapy. Herein, we describe an unusual presentation of IMT

involving an anatomical region rarely implicated in this disease process. A

15-year-old male patient came to the ER with dysphagia and coffee ground emesis.

On esophagogastroscopy, a nodular luminal obstructing 30 × 50 mm mass in the

lower esophagus was found, which was continuous with a large, partially

circumferential gastric mass extending from the mid-body to the proximal antrum.

Biopsies from esophageal and gastric masses revealed submucosal lesions composed

of cytologically bland spindle and epithelioid cells, intermingled with

inflammatory infiltrate, for which several immunohistochemical (IHC) stains were

performed. The molecular study demonstrated ATIC::ALK fusion. Based on

morphological, IHC, and molecular study findings, the diagnosis of ALK-positive

IMT was rendered. Because surgical excision was deemed infeasible, the patient

was started on ALK-inhibiting therapy with crizotinib. The patient responded well

with no evidence of residual or recurrent disease on follow-up imaging or

surveillance esophagogastroduodenoscopy. Crizotinib was ultimately discontinued

after 10 months of therapy, and the patient continues to undergo surveillance

imaging for monitoring of disease burden.

FAU - Billingsley, Benjamin C

AU - Billingsley BC

AUID- ORCID: 0009-0007-2540-0050

AD - Department of Internal Medicine, University of Mississippi Medical Center,

Jackson, MS, USA. RINGGOLD: 21693

FAU - Chaudhary, Ritica

AU - Chaudhary R

AD - Department of Pathology, University of Mississippi Medical Center, Jackson, MS,

USA. RINGGOLD: 21693

FAU - Morris, Michael W

AU - Morris MW

AD - Division of Pediatric, Department of Surgery, University of Mississippi Medical

Center, Jackson, MS, USA. RINGGOLD: 21693

FAU - Cox, Jennifer A

AU - Cox JA

AD - Division of Hematology and Oncology, Department of Pediatrics, University of

Mississippi Medical Center, Jackson, MS, USA. RINGGOLD: 21693

FAU - Camacho-Gomez, Sandra M

AU - Camacho-Gomez SM

AD - Division of Gastroenterology, Hepatology and Nutrition, Department of Pediatrics,

University of Texas at Austin, Austin, TX, USA.

FAU - Varshney, Neha

AU - Varshney N

AUID- ORCID: 0000-0003-4065-1521

AD - Department of Pathology, University of Mississippi Medical Center, Jackson, MS,

USA. RINGGOLD: 21693

LA - eng

PT - Journal Article

DEP - 20240424

PL - United States

TA - Int J Surg Pathol

JT - International journal of surgical pathology

JID - 9314927

SB - IM

OTO - NOTNLM

OT - ALK

OT - GI bleed

OT - IMT

OT - anaplastic lymphoma kinase

OT - crizotinib

OT - gastroesophageal junction

OT - inflammatory myofibroblastic tumor

OT - inflammatory pseudotumor

OT - pediatric

OT - stomach

COIS- Declaration of Conflicting InterestsThe author(s) declared no potential conflicts

of interest with respect to the research, authorship, and/or publication of this

article.

EDAT- 2024/04/24 14:20

MHDA- 2024/04/24 14:20

CRDT- 2024/04/24 11:42

PHST- 2024/04/24 14:20 [medline]

PHST- 2024/04/24 14:20 [pubmed]

PHST- 2024/04/24 11:42 [entrez]

AID - 10.1177/10668969241246470 [doi]

PST - aheadofprint

SO - Int J Surg Pathol. 2024 Apr 24:10668969241246470. doi: 10.1177/10668969241246470.

PMID- 38629322

OWN - NLM

STAT- MEDLINE

DCOM- 20240714

LR - 20240714

IS - 1365-2559 (Electronic)

IS - 0309-0167 (Linking)

VI - 85

IP - 2

DP - 2024 Aug

TI - Advances in uterine inflammatory myofibroblastic tumours: Diagnostic challenges

and risk stratification.

PG - 215-223

LID - 10.1111/his.15194 [doi]

AB - The current understanding of inflammatory myofibroblastic tumours (IMTs) of the

gynaecological tract has recently been enhanced by their increased recognition.

This increase is largely due to greater accessibility to RNA-based molecular

assays used to identify their defining ALK rearrangements. This review summarises

the clinical characteristics, morphological spectrum, immunohistochemical profile

and molecular underpinnings of uterine IMT. Additionally, this review discusses

practical diagnostic considerations including overlap between uterine IMT and

smooth muscle tumours as well as pregnancy-associated uterine IMT. Finally, we

highlight recent literature demonstrating the potential for aggressive behaviour

in uterine IMT, including a novel risk stratification model for identifying

high-risk IMT.

CI - © 2024 John Wiley & Sons Ltd.

FAU - Umetsu, Sarah E

AU - Umetsu SE

AUID- ORCID: 0000-0003-2175-0671

AD - Department of Pathology, University of California San Francisco, San Francisco,

CA, USA.

FAU - Ladwig, Nicholas R

AU - Ladwig NR

AUID- ORCID: 0000-0002-2696-5121

AD - Department of Pathology, University of California San Francisco, San Francisco,

CA, USA.

LA - eng

PT - Journal Article

PT - Review

DEP - 20240417

PL - England

TA - Histopathology

JT - Histopathology

JID - 7704136

SB - IM

MH - Humans

MH - Female

MH - *Uterine Neoplasms/diagnosis/pathology

MH - Neoplasms, Muscle Tissue/diagnosis/pathology/genetics

MH - Pregnancy

MH - Risk Assessment

MH - Myofibroblasts/pathology

OTO - NOTNLM

OT - ALK

OT - IMT

OT - inflammatory myofibroblastic tumor

OT - risk stratification

OT - uterus

EDAT- 2024/04/17 06:42

MHDA- 2024/07/15 00:41

CRDT- 2024/04/17 05:23

PHST- 2024/03/28 00:00 [revised]

PHST- 2024/02/01 00:00 [received]

PHST- 2024/03/28 00:00 [accepted]

PHST- 2024/07/15 00:41 [medline]

PHST- 2024/04/17 06:42 [pubmed]

PHST- 2024/04/17 05:23 [entrez]

AID - 10.1111/his.15194 [doi]

PST - ppublish

SO - Histopathology. 2024 Aug;85(2):215-223. doi: 10.1111/his.15194. Epub 2024 Apr 17.

PMID- 38622850

OWN - NLM

STAT- MEDLINE

DCOM- 20240417

LR - 20240425

IS - 2324-9269 (Electronic)

IS - 2324-9269 (Linking)

VI - 12

IP - 4

DP - 2024 Apr

TI - Reclassification of a spindle cell sarcoma after identification of a TFG-ROS1

fusion: A case demonstrating the clinical benefit of next-generation sequencing

in sarcoma.

PG - e2423

LID - 10.1002/mgg3.2423 [doi]

LID - e2423

AB - BACKGROUND: Inflammatory myofibroblastic tumors (IMTs) are rare mesenchymal soft

tissue sarcomas that often present diagnostic challenges due to their wide and

varied morphology. A subset of IMTs have fusions involving ALK or ROS1. The role

of next-generation sequencing (NGS) for classification of unselected sarcomas

remains controversial. METHODS AND RESULTS: We report a case of a metastatic

sarcoma in a 34-year-old female originally diagnosed as an unclassified spindle

cell sarcoma with myofibroblastic differentiation and later reclassified as IMT

after NGS revealed a TFG-ROS1 rearrangement. Histologically, the neoplasm had

spindle cell morphology with a lobulated to focally infiltrative growth pattern

with scant inflammatory cell infiltrate. Immunohistochemistry demonstrated focal

desmin and variable smooth muscle actin staining but was negative for SOX10,

S100, and CD34. Fluorescence in situ hybridization was negative for USP6 or ALK

gene rearrangements. NGS revealed a TFG-ROS1 rearrangement and the patient was

treated with crizotinib with clinical benefit. CONCLUSIONS: We discuss the role

of NGS as well as its potential benefit in patients with unresectable,

ALK-negative metastatic disease. Considering this case and previous literature,

we support the use of NGS for patients requiring systemic treatment.

CI - © 2024 The Authors. Molecular Genetics & Genomic Medicine published by Wiley

Periodicals LLC.

FAU - Lim, John J

AU - Lim JJ

AD - Division of Medical Oncology, University of Washington, Seattle, Washington, USA.

FAU - Chen, Eleanor Y

AU - Chen EY

AUID- ORCID: 0000-0003-4372-7560

AD - Department of Laboratory Medicine and Pathology, University of Washington,

Seattle, Washington, USA.

FAU - Schaub, Stephanie K

AU - Schaub SK

AD - Division of Radiation Oncology, University of Washington, Seattle, Washington,

USA.

FAU - Wagner, Michael J

AU - Wagner MJ

AUID- ORCID: 0000-0002-0753-9282

AD - Division of Medical Oncology, University of Washington, Seattle, Washington, USA.

AD - Clinical Research Division, Fred Hutchinson Cancer Center, Seattle, Washington,

USA.

LA - eng

PT - Case Reports

PT - Journal Article

PL - United States

TA - Mol Genet Genomic Med

JT - Molecular genetics & genomic medicine

JID - 101603758

RN - EC 2.7.10.1 (Protein-Tyrosine Kinases)

RN - EC 2.7.10.1 (Anaplastic Lymphoma Kinase)

RN - 0 (Proto-Oncogene Proteins)

RN - EC 3.4.19.12 (USP6 protein, human)

RN - EC 3.4.19.12 (Ubiquitin Thiolesterase)

RN - EC 2.7.10.1 (ROS1 protein, human)

RN - 0 (TFG protein, human)

RN - 0 (Vesicular Transport Proteins)

SB - IM

MH - Female

MH - Humans

MH - Adult

MH - *Protein-Tyrosine Kinases/genetics

MH - Anaplastic Lymphoma Kinase/genetics

MH - In Situ Hybridization, Fluorescence

MH - Proto-Oncogene Proteins/genetics

MH - *Sarcoma/drug therapy/genetics/pathology

MH - High-Throughput Nucleotide Sequencing

MH - Ubiquitin Thiolesterase/genetics

MH - Vesicular Transport Proteins/genetics

PMC - PMC11019117

OTO - NOTNLM

OT - NGS

OT - ROS1

OT - crizotinib

OT - inflammatory myofibroblastic tumor (IMT)

OT - sarcoma

COIS- MJW – Consulting/Advisory fees from Adaptimmune, Deciphera, Epizyme, Aadi,

PharmaEssentia, Boehringer Inghelheim. JJL, EYC, and SKS have no potential

conflicts to disclose.

EDAT- 2024/04/16 12:42

MHDA- 2024/04/17 06:42

PMCR- 2024/04/15

CRDT- 2024/04/16 01:23

PHST- 2024/02/14 00:00 [revised]

PHST- 2023/10/17 00:00 [received]

PHST- 2024/03/19 00:00 [accepted]

PHST- 2024/04/17 06:42 [medline]

PHST- 2024/04/16 12:42 [pubmed]

PHST- 2024/04/16 01:23 [entrez]

PHST- 2024/04/15 00:00 [pmc-release]

AID - MGG32423 [pii]

AID - 10.1002/mgg3.2423 [doi]

PST - ppublish

SO - Mol Genet Genomic Med. 2024 Apr;12(4):e2423. doi: 10.1002/mgg3.2423.

PMID- 38615503

OWN - NLM

STAT- MEDLINE

DCOM- 20240515

LR - 20240515

IS - 1872-7727 (Electronic)

IS - 0720-048X (Linking)

VI - 175

DP - 2024 Jun

TI - Imaging of pediatric gastrointestinal tumors: A tertiary center experience over

19 years.

PG - 111461

LID - S0720-048X(24)00177-3 [pii]

LID - 10.1016/j.ejrad.2024.111461 [doi]

AB - PURPOSE: Gastrointestinal tract (GIT) tumors in children are rare and there is a

scarcity of data on their imaging features. The purpose of this study was to

determine thefrequency of various GIT tumor types in children and to identify key

imaging characteristics. METHODS: This retrospective, single-center study was

approved by the local ethics committee. Children with histologically proven GIT

tumours (malignantand benign) who had imaging available on the institutional PACS

between May 1, 2000 and Dec 31, 2019 were included. Demographic data and

available imaging was reviewed by two blinded radiologists. RESULTS: In total, 90

children (45 male, mean age 9.3 ± 4.3 years) with GIT tumours were included. The

final diagnoses included polyps (n = 28), lymphomas/PTLD (n = 27), neuroendocrine

tumours (n = 16), adenocarcinoma (n = 6), adenoma (n = 5), gastrointestinal

stromal tumor (GIST) (n = 3), inflammatory myofibroblastic tumours (n = 2) and

lastly leiomyoblastoma, leiomyoma and lipoma (1 each). All GIT segments were

affected, but overall the small and large bowel had most lesions. Eighty-one

percent children had a single lesion while remaining 19 % had multiple lesions.

The neoplastic process manifested as intra-luminal lesion (58 %) or wall

thickening (42 %) on imaging. Multiple cystic areas and vascular pedicle for

polyps; and hypoechogenecity of the mass or wall thickening and aneurysmal

dilatation for lymphomas, were the characteristic imaging features. None of the

neuroendocrine tumours affecting appendix were seen on pre-resection imaging.

CONCLUSIONS: Variety of benign and malignant tumors are seen throughout the

childhood. Polyps, lymphomas and appendiceal neuroendocrine tumors are common

lesions. Characteristic imaging features of juvenile polyps and lymphomas on

ultrasound may help narrowing the differentials, and guide further work up.

CI - Copyright © 2024 Elsevier B.V. All rights reserved.

FAU - Kraus, Mareen S

AU - Kraus MS

AD - Department of Diagnostic Imaging, Hospital for Sick Children, 555 University Ave,

Toronto, M5G 1X8, Canada; Department of Diagnostic Radiology, Dalhousie

University/IWK, 5850/5980 University Ave, Halifax, NS B3K 6R8, Canada.

FAU - Selvam, Swathi

AU - Selvam S

AD - Department of Diagnostic Imaging, Hospital for Sick Children, 555 University Ave,

Toronto, M5G 1X8, Canada; Medical Imaging Department, Royal Children s Hospital,

50 Flemington Rd, Parkville, VIC 3052, Australia.

FAU - Siddiqui, Iram

AU - Siddiqui I

AD - Department of Pathology, Hospital for Sick Children, 555 University Ave, Toronto,

M5G 1X8, Canada.

FAU - Reyes, Jeanette A

AU - Reyes JA

AD - Department of Pathology, Hospital for Sick Children, 555 University Ave, Toronto,

M5G 1X8, Canada.

FAU - Chavhan, Govind B

AU - Chavhan GB

AD - Department of Diagnostic Imaging, Hospital for Sick Children, 555 University Ave,

Toronto, M5G 1X8, Canada; Department of Medical Imaging, University of Toronto,

Canada. Electronic address: drgovindchavhan@yahoo.com.

LA - eng

PT - Journal Article

DEP - 20240410

PL - Ireland

TA - Eur J Radiol

JT - European journal of radiology

JID - 8106411

SB - IM

MH - Humans

MH - Male

MH - Female

MH - Child

MH - Retrospective Studies

MH - *Gastrointestinal Neoplasms/diagnostic imaging/pathology

MH - Tertiary Care Centers

MH - Adolescent

MH - Child, Preschool

MH - Magnetic Resonance Imaging/methods

OTO - NOTNLM

OT - Children

OT - Gastrointestinal tumour

OT - Imaging

OT - Paediatric

OT - Pathology

OT - Polyposis

COIS- Declaration of competing interest The authors declare that they have no known

competing financial interests or personal relationships that could have appeared

to influence the work reported in this paper.

EDAT- 2024/04/15 00:42

MHDA- 2024/05/16 00:44

CRDT- 2024/04/14 18:06

PHST- 2023/12/09 00:00 [received]

PHST- 2024/04/04 00:00 [revised]

PHST- 2024/04/08 00:00 [accepted]

PHST- 2024/05/16 00:44 [medline]

PHST- 2024/04/15 00:42 [pubmed]

PHST- 2024/04/14 18:06 [entrez]

AID - S0720-048X(24)00177-3 [pii]

AID - 10.1016/j.ejrad.2024.111461 [doi]

PST - ppublish

SO - Eur J Radiol. 2024 Jun;175:111461. doi: 10.1016/j.ejrad.2024.111461. Epub 2024

Apr 10.

PMID- 38594735

OWN - NLM

STAT- MEDLINE

DCOM- 20240411

LR - 20240412

IS - 1752-1947 (Electronic)

IS - 1752-1947 (Linking)

VI - 18

IP - 1

DP - 2024 Apr 10

TI - Anaplastic lymphoma kinase-positive pulmonary inflammatory myofibroblastic

tumour: a case report.

PG - 167

LID - 10.1186/s13256-024-04472-9 [doi]

LID - 167

AB - BACKGROUND: Pulmonary inflammatory myofibroblastic tumour (IMT) is a rare

condition that usually presents in young individuals and is associated with

anaplastic lymphoma kinase (ALK)-translocation. CASE PRESENTATION: We report a

case of an 18-year-old Caucasian man with ALK-translocated pulmonary IMT treated

with multimodality therapy. The patient presented with breathlessness and was

found to have a collapsed left lung. Further investigations revealed an

ALK-translocated pulmonary IMT. This is usually treated with an ALK-inhibitor but

patient declined after discussing potential side-effects and had repeated rigid

bronchoscopic interventions for local disease control. Due to persistent local

recurrence, patient received radical external beam radiotherapy (EBRT) with pulse

steroids, and one year later started on Ibuprofen, a non-steroidal

anti-inflammatory agent (NSAID). Following multimodality treatment, he developed

a complete response. He remains treatment-free for the past seven years. Eleven

years on from his diagnosis, he remains in remission with a ECOG performance

status of zero. CONCLUSIONS: Achieving long-term local control in pulmonary IMT

can be challenging. Multimodality treatment is sometimes needed but the overall

outlook remains good.

CI - © 2024. Crown.

FAU - Tong, Daniel

AU - Tong D

AUID- ORCID: 0000-0003-1764-3830

AD - Lung Unit, The Royal Marsden NHS Foundation Trust, Sutton, UK. dtong@nhs.net.

FAU - Chisholm, Julia

AU - Chisholm J

AD - Children and Young People's Unit, Institute of Cancer Research, Royal Marsden

Hospital, Sutton, SM2 5NG, UK.

FAU - Madden, Brendan

AU - Madden B

AD - Department of Cardiothoracic Medicine, St Georges Hospital, Blackshaw Road,

London, SW17 0QT, UK.

FAU - Ahmed, Merina

AU - Ahmed M

AD - Lung Unit, The Royal Marsden NHS Foundation Trust, Sutton, UK.

LA - eng

PT - Case Reports

PT - Journal Article

DEP - 20240410

PL - England

TA - J Med Case Rep

JT - Journal of medical case reports

JID - 101293382

RN - EC 2.7.10.1 (Anaplastic Lymphoma Kinase)

RN - 0 (Protein Kinase Inhibitors)

SB - IM

MH - Male

MH - Humans

MH - Adolescent

MH - Anaplastic Lymphoma Kinase/genetics

MH - *Protein Kinase Inhibitors/therapeutic use

MH - Translocation, Genetic

MH - Pathologic Complete Response

MH - *Neoplasms

PMC - PMC11005263

OTO - NOTNLM

OT - ALK

OT - Long-term remission

OT - Multimodality

OT - Pulmonary inflammatory myofibroblastic tumour

OT - Radiotherapy and pulse steroids

COIS- The authors declare that they have no competing interests.

EDAT- 2024/04/10 00:43

MHDA- 2024/04/11 06:42

PMCR- 2024/04/10

CRDT- 2024/04/09 23:44

PHST- 2023/07/14 00:00 [received]

PHST- 2024/02/23 00:00 [accepted]

PHST- 2024/04/11 06:42 [medline]

PHST- 2024/04/10 00:43 [pubmed]

PHST- 2024/04/09 23:44 [entrez]

PHST- 2024/04/10 00:00 [pmc-release]

AID - 10.1186/s13256-024-04472-9 [pii]

AID - 4472 [pii]

AID - 10.1186/s13256-024-04472-9 [doi]

PST - epublish

SO - J Med Case Rep. 2024 Apr 10;18(1):167. doi: 10.1186/s13256-024-04472-9.

PMID- 38590947

OWN - NLM

STAT- PubMed-not-MEDLINE

LR - 20240410

IS - 2049-9442 (Electronic)

IS - 2049-9434 (Print)

IS - 2049-9434 (Linking)

VI - 20

IP - 5

DP - 2024 May

TI - Rare histopathological finding of ovarian pseudotumor: A case report.

PG - 79

LID - 10.3892/br.2024.1767 [doi]

LID - 79

AB - An inflammatory pseudotumor (IPT) is a benign, rare chronic inflammatory process

that is destructive to normal histology of the involved organs. While IPT most

frequently affects the lung and orbits, it can occur in almost any part of the

body. Additionally, histopathological examination is often difficult to

interpret, typically showing myofibroblasts and mixed inflammatory and

spindle-shaped cells. The histopathological picture may resemble low grade

fibrosarcoma with inflammatory cells, making the differentiation between benign

and malignant diseases more difficult and potentially requiring specialized

histopathological studies. In the present study, a 39-year-old healthy female

patient with no history of sexual activity presented to The Specialty Hospital

(Amman Jordan) in January 2023 with mild lower abdominal pain. A pelvic

ultrasound scan showed a complex right ovarian cyst measuring 6.0x6.5 cm. Tumor

markers were normal. The patient underwent laparotomy with an ovarian cystectomy

and left ovarian, omental and peritoneal biopsies. The histopathology of the cyst

was suggestive of IPT. Other histopathological results were normal. The patient

was followed up for 1 year after surgery with no recurrence of the disease.

CI - Copyright: © 2024 Samara et al.

FAU - Samara, Batool

AU - Samara B

AD - Department of Obstetrics and Gynaecology, Specialty Hospital, Amman 11194,

Jordan.

FAU - Shriam, Suha

AU - Shriam S

AD - Department of Obstetrics and Gynaecology, Specialty Hospital, Amman 11194,

Jordan.

FAU - Al-Balas, Aseel

AU - Al-Balas A

AD - Department of Obstetrics and Gynaecology, Specialty Hospital, Amman 11194,

Jordan.

FAU - Isied, Reham

AU - Isied R

AD - Department of Obstetrics and Gynaecology, Specialty Hospital, Amman 11194,

Jordan.

FAU - Abu Mahfouz, Ismaiel

AU - Abu Mahfouz I

AD - Department of Obstetrics and Gynaecology, Faculty of Medicine, Al-Balqa Applied

University, Al Salt 19117, Jordan.

FAU - Almasri, Anwar

AU - Almasri A

AD - Department of Histopathology, Specialty Hospital, Amman 11194, Jordan.

LA - eng

PT - Case Reports

PT - Journal Article

DEP - 20240319

PL - England

TA - Biomed Rep

JT - Biomedical reports

JID - 101613227

PMC - PMC10999899

OTO - NOTNLM

OT - inflammatory pseudotumor

OT - ovarian cyst

COIS- The authors declare that they have no competing interests.

EDAT- 2024/04/09 06:45

MHDA- 2024/04/09 06:46

PMCR- 2024/03/19

CRDT- 2024/04/09 03:56

PHST- 2023/06/27 00:00 [received]

PHST- 2024/01/15 00:00 [accepted]

PHST- 2024/04/09 06:46 [medline]

PHST- 2024/04/09 06:45 [pubmed]

PHST- 2024/04/09 03:56 [entrez]

PHST- 2024/03/19 00:00 [pmc-release]

AID - BR-20-5-01767 [pii]

AID - 10.3892/br.2024.1767 [doi]

PST - epublish

SO - Biomed Rep. 2024 Mar 19;20(5):79. doi: 10.3892/br.2024.1767. eCollection 2024

May.

PMID- 38584790

OWN - NLM

STAT- PubMed-not-MEDLINE

LR - 20240409

IS - 1301-5680 (Print)

IS - 2149-8156 (Electronic)

IS - 1301-5680 (Linking)

VI - 32

IP - Suppl1

DP - 2024 Jan

TI - Pulmonary tumors in childhood.

PG - S73-S77

LID - 10.5606/tgkdc.dergisi.2024.25863 [doi]

AB - Pulmonary tumors in childhood are rare, but the majority are malignant. The

histopathologic spectrum is quite diverse, including inflammatory myofibroblastic

tumor, hamartoma, primary pulmonary paraganglioma, carcinoid tumor,

mucoepidermoid carcinoma, pleuropulmonary blastoma, adenocarcinoma, squamous cell

carcinoma, and sarcomas. Nonspecific clinical and radiological findings result in

late and incorrect diagnoses. Although surgical resection is the initial and

proper treatment method, additional adjuvant therapy is dependent on both tumor

stage and histopathologic type.

CI - Copyright © 2024, Turkish Society of Cardiovascular Surgery.

FAU - Özkan, Murat

AU - Özkan M

AD - Department of Thoracic Surgery, Ankara University Faculty of Medicine, Ankara,

Türkiye.

LA - eng

PT - Journal Article

PT - Review

DEP - 20240205

PL - Turkey

TA - Turk Gogus Kalp Damar Cerrahisi Derg

JT - Turk gogus kalp damar cerrahisi dergisi

JID - 100887967

PMC - PMC10995685

OTO - NOTNLM

OT - Childhood

OT - lung

OT - pulmonary tumor.

COIS- Conflict of Interest: The author declared no conflicts of interest with respect

to the authorship and/or publication of this article.

EDAT- 2024/04/08 06:43

MHDA- 2024/04/08 06:44

PMCR- 2024/02/05

CRDT- 2024/04/08 04:19

PHST- 2023/12/18 00:00 [received]

PHST- 2024/01/04 00:00 [accepted]

PHST- 2024/04/08 06:44 [medline]

PHST- 2024/04/08 06:43 [pubmed]

PHST- 2024/04/08 04:19 [entrez]

PHST- 2024/02/05 00:00 [pmc-release]

AID - 10.5606/tgkdc.dergisi.2024.25863 [doi]

PST - epublish

SO - Turk Gogus Kalp Damar Cerrahisi Derg. 2024 Feb 5;32(Suppl1):S73-S77. doi:

10.5606/tgkdc.dergisi.2024.25863. eCollection 2024 Jan.

PMID- 38579046

OWN - NLM

STAT- MEDLINE

DCOM- 20240408

LR - 20240506

IS - 1536-5964 (Electronic)

IS - 0025-7974 (Print)

IS - 0025-7974 (Linking)

VI - 103

IP - 14

DP - 2024 Apr 5

TI - EBER-negative inflammatory pseudotumor-like follicular dendritic cell sarcoma of

liver: A case report.

PG - e37651

LID - 10.1097/MD.0000000000037651 [doi]

LID - e37651

AB - RATIONALE: Inflammatory pseudotumor-like follicular dendritic cell sarcoma

(IPT-like FDCS) of the liver is rare. It was previously believed that

Epstein-Barr virus (EBV) positivity was a necessary criterion for pathological

diagnosis. However, we found that there were also cases of EBV negativity.

Therefore, clinicians and pathologists are reminded that EBV positivity is not a

necessary condition for diagnosis. PATIENT CONCERNS: A 70-year-old female

underwent computed tomography (CT) examination for upper abdominal discomfort,

which revealed the presence of a liver tumor. Follow-up revealed that the tumor

had progressively increased in size. DIAGNOSIS: The final diagnosis was an

IPT-like follicular cell sarcoma, based on CT, MRI, HE staining, and

immunohistochemical staining. INTERVENTIONS: The patient underwent a laparoscopic

left hemihepatectomy. OUTCOMES: The patient has not undergone any special

treatment, such as radiotherapy and chemotherapy, and has been followed up for

over 3 years without experiencing any recurrence. LESSONS: IPT-like FDCS is a

rare tumor that lacks definitive criteria, and its diagnosis mainly relies on

pathological findings. Previously, it was believed that being EBV-positive was an

important condition for diagnosis. Primary IPT-like FDCS in the liver is even

rarer, and the patient in this case tested negative for EBV. It may be necessary

for pathologists to consider the role of EBV in the diagnosis of IPT-like FDCS.

CI - Copyright © 2024 the Author(s). Published by Wolters Kluwer Health, Inc.

FAU - Zhang, Qiang

AU - Zhang Q

AUID- ORCID: 0009-0002-0748-352

AD - Department of Hepatobiliary Surgery, Affiliated Hospital of Xuzhou Medical

University, Xuzhou, Jiangsu Province, China.

FAU - Gao, Jialin

AU - Gao J

AD - Department of Pathology, Affiliated Hospital of Xuzhou Medical University,

Xuzhou, Jiangsu Province, China.

LA - eng

PT - Case Reports

PT - Journal Article

PL - United States

TA - Medicine (Baltimore)

JT - Medicine

JID - 2985248R

SB - IM

MH - Female

MH - Humans

MH - Aged

MH - *Dendritic Cell Sarcoma, Follicular/diagnosis/surgery/pathology

MH - *Epstein-Barr Virus Infections/complications/diagnosis

MH - *Granuloma, Plasma Cell/diagnosis/surgery/pathology

MH - Herpesvirus 4, Human

MH - Liver/diagnostic imaging/pathology

PMC - PMC10994431

COIS- The authors have no funding and conflicts of interest to disclose.

EDAT- 2024/04/05 18:42

MHDA- 2024/04/08 06:42

PMCR- 2024/04/05

CRDT- 2024/04/05 14:13

PHST- 2024/04/08 06:42 [medline]

PHST- 2024/04/05 18:42 [pubmed]

PHST- 2024/04/05 14:13 [entrez]

PHST- 2024/04/05 00:00 [pmc-release]

AID - 00005792-202404050-00025 [pii]

AID - MD-D-23-10402 [pii]

AID - 10.1097/MD.0000000000037651 [doi]

PST - ppublish

SO - Medicine (Baltimore). 2024 Apr 5;103(14):e37651. doi:

10.1097/MD.0000000000037651.

PMID- 38570104

OWN - NLM

STAT- MEDLINE

DCOM- 20240530

LR - 20240530

IS - 1879-1336 (Electronic)

IS - 1054-8807 (Linking)

VI - 71

DP - 2024 Jul-Aug

TI - Rare cardiac inflammatory pseudotumor in a toddler: Complementary roles of

cardiac magnetic resonance and positron emission tomography.

PG - 107639

LID - S1054-8807(24)00035-8 [pii]

LID - 10.1016/j.carpath.2024.107639 [doi]

AB - We present a rare pediatric case of cardiac inflammatory pseudotumor (IPT) with a

unique presentation of fever of unknown origin with markedly elevated

inflammatory markers. A right atrial mass was discovered incidentally by

echocardiography. The cardiac magnetic resonance (CMR) signal characteristics and

mass location were not consistent with any of the common benign cardiac tumors of

childhood. The presence of high signal intensity on T2 imaging and late

gadolinium enhancement, in conjunction with intense metabolic activity at the

mass site on positron emission tomography (PET), raised the possibility of an

inflammatory or malignant mass. The diagnosis of IPT was confirmed by biopsy. Our

case highlights the utility of PET imaging to confirm the inflammatory nature and

extent of an IPT.

CI - Copyright © 2024 Elsevier Inc. All rights reserved.

FAU - Mejia-Bautista, Melissa

AU - Mejia-Bautista M

AD - Department of Pathology, Boston Children's Hospital, Harvard Medical School,

Boston, MA, USA. Electronic address: Melissa.mejiabautista@childrens.harvard.edu.

FAU - Romanowicz, Jennifer

AU - Romanowicz J

AD - Department of Cardiology, Department of Pediatrics, Boston Children's Hospital,

Harvard Medical School, Boston, MA, USA.

FAU - Hollowell, Monica

AU - Hollowell M

AD - Department of Pathology, Boston Children's Hospital, Harvard Medical School,

Boston, MA, USA.

FAU - Geva, Tal

AU - Geva T

AD - Department of Cardiology, Department of Pediatrics, Boston Children's Hospital,

Harvard Medical School, Boston, MA, USA.

FAU - Carreon, Chrystalle Katte

AU - Carreon CK

AD - Department of Pathology, Boston Children's Hospital, Harvard Medical School,

Boston, MA, USA.

FAU - Beroukhim, Rebecca S

AU - Beroukhim RS

AD - Department of Cardiology, Department of Pediatrics, Boston Children's Hospital,

Harvard Medical School, Boston, MA, USA.

LA - eng

PT - Case Reports

PT - Journal Article

DEP - 20240401

PL - United States

TA - Cardiovasc Pathol

JT - Cardiovascular pathology : the official journal of the Society for Cardiovascular

Pathology

JID - 9212060

SB - IM

MH - Humans

MH - *Granuloma, Plasma Cell/diagnostic imaging/pathology

MH - *Positron-Emission Tomography

MH - Magnetic Resonance Imaging

MH - Biopsy

MH - Child, Preschool

MH - Male

MH - Echocardiography

MH - Incidental Findings

MH - Fever of Unknown Origin/diagnostic imaging/etiology

MH - Predictive Value of Tests

MH - Heart Diseases/diagnostic imaging/pathology

MH - Female

OTO - NOTNLM

OT - Cardiac MRI

OT - Cardiac inflammatory pseudotumor

OT - Cardiac mass

OT - Pediatric cardiology

OT - Tissue characterization

COIS- Declaration of competing interest The authors declare that they have no known

competing financial interests or personal relationships that could have appeared

to influence the work reported in this paper.

EDAT- 2024/04/04 00:43

MHDA- 2024/05/31 00:42

CRDT- 2024/04/03 21:38

PHST- 2023/11/22 00:00 [received]

PHST- 2024/03/27 00:00 [revised]

PHST- 2024/03/28 00:00 [accepted]

PHST- 2024/05/31 00:42 [medline]

PHST- 2024/04/04 00:43 [pubmed]

PHST- 2024/04/03 21:38 [entrez]

AID - S1054-8807(24)00035-8 [pii]

AID - 10.1016/j.carpath.2024.107639 [doi]

PST - ppublish

SO - Cardiovasc Pathol. 2024 Jul-Aug;71:107639. doi: 10.1016/j.carpath.2024.107639.

Epub 2024 Apr 1.

PMID- 38556256

OWN - NLM

STAT- MEDLINE

DCOM- 20240611

LR - 20240711

IS - 1600-0560 (Electronic)

IS - 0303-6987 (Linking)

VI - 51

IP - 7

DP - 2024 Jul

TI - Fusion-driven cutaneous and superficial mesenchymal and adnexal tumors-A

clinicopathologic and molecular study of 15 cases, including a novel case of

ACTB::ZMIZ2-rearranged adnexal carcinoma.

PG - 538-548

LID - 10.1111/cup.14610 [doi]

AB - BACKGROUND: While the list of fusion-driven soft tissue neoplasms is expanding

rapidly, their importance among cutaneous and superficial mesenchymal and adnexal

neoplasms remains poorly understood. This challenge is especially evident in

cases with ambiguous histopathology that are difficult to classify based on

morphology. AIMS: Our goal was to investigate the benefits of next-generation

sequencing in diagnosing complex cutaneous neoplasms. MATERIALS & METHODS:

Departmental archives were searched for fusion-driven cutaneous neoplasms. Slides

were retrieved and clinical information including follow-up was obtained.

RESULTS: Fifteen cases occurred in eight female and seven male patients, with a

median age of 26 years (range: 1-83) at diagnosis. Tumors involved the

extremities (9), scalp (5), and head and neck (1). Predominant features included

myoepithelial (5), nested spindled with clear cytoplasm (2), atypical

adnexal/squamoid (2), small round blue cell (2), cellular spindled (3), and

fibrohistiocytic morphology (1). Most frequently encountered fusions involved

EWSR1 (6) fused to ERG (1), FLI1 (1), CREB1 (2), CREM (1), PBX3 (1), followed by

PLAG1 (4) with LIFR (2), TRPS1 (1) and CHCHD7. Additional fusions encountered

were YAP1::NUTM1, EML4::ALK, SS18::SSX1 (2), and a novel fusion: ACTB::ZMIZ2.

Integration of histologic features and molecular findings led to final diagnoses

of primary cutaneous Ewing sarcoma (2), soft tissue myoepithelioma (4), cutaneous

syncytial myoepithelioma (1), cutaneous adnexal carcinoma (1), porocarcinoma (1),

inflammatory myofibroblastic tumor (1), synovial sarcoma (2), clear cell sarcoma

(2), and angiomatoid fibrous histiocytoma (1). DISCUSSION AND CONCLUSION: Our

results show that fusion testing can be a helpful diagnostic tool, especially in

cases with unusual or uncommon morphology in superficial sites. Furthermore, it

can allow for the identification of potential therapeutic targets in some

instances.

CI - © 2024 John Wiley & Sons A/S. Published by John Wiley & Sons Ltd.

FAU - Dehner, Carina A

AU - Dehner CA

AD - Department of Laboratory Medicine and Pathology, Mayo Clinic, Rochester,

Minnesota, USA.

AD - Department of Anatomic Pathology and Laboratory Medicine, Indiana University,

Indianapolis, Indiana, USA.

FAU - Johnson, Emma F

AU - Johnson EF

AD - Department of Laboratory Medicine and Pathology, Mayo Clinic, Rochester,

Minnesota, USA.

AD - Department of Dermatology, Mayo Clinic, Rochester, Minnesota, USA.

FAU - Wieland, Carrie N

AU - Wieland CN

AD - Department of Laboratory Medicine and Pathology, Mayo Clinic, Rochester,

Minnesota, USA.

AD - Department of Dermatology, Mayo Clinic, Rochester, Minnesota, USA.

FAU - Camilleri, Michael J

AU - Camilleri MJ

AD - Department of Laboratory Medicine and Pathology, Mayo Clinic, Rochester,

Minnesota, USA.

AD - Department of Dermatology, Mayo Clinic, Rochester, Minnesota, USA.

FAU - Kajdacsy-Balla, Andre

AU - Kajdacsy-Balla A

AD - Department of Pathology, University of Chicago Illinois, Chicago, Illinois, USA.

FAU - Oliveira, Andre M

AU - Oliveira AM

AD - Department of Laboratory Medicine and Pathology, Mayo Clinic, Rochester,

Minnesota, USA.

FAU - Halling, Kevin C

AU - Halling KC

AD - Department of Laboratory Medicine and Pathology, Mayo Clinic, Rochester,

Minnesota, USA.

FAU - Gupta, Sounak

AU - Gupta S

AD - Department of Laboratory Medicine and Pathology, Mayo Clinic, Rochester,

Minnesota, USA.

FAU - Guo, Ruifeng

AU - Guo R

AD - Department of Laboratory Medicine and Pathology, Mayo Clinic, Rochester,

Minnesota, USA.

AD - Department of Laboratory Medicine and Pathology, Mayo Clinic, Jacksonville,

Florida, USA.

LA - eng

PT - Journal Article

DEP - 20240331

PL - United States

TA - J Cutan Pathol

JT - Journal of cutaneous pathology

JID - 0425124

RN - 0 (Oncogene Proteins, Fusion)

RN - 0 (Transcription Factors)

SB - IM

MH - Humans

MH - Female

MH - Male

MH - Adult

MH - *Skin Neoplasms/pathology/genetics/metabolism

MH - Middle Aged

MH - Aged

MH - Child

MH - Adolescent

MH - Aged, 80 and over

MH - Child, Preschool

MH - Infant

MH - Oncogene Proteins, Fusion/genetics

MH - High-Throughput Nucleotide Sequencing/methods

MH - Transcription Factors/genetics

MH - Neoplasms, Adnexal and Skin Appendage/pathology/genetics/diagnosis

MH - Young Adult

MH - Gene Rearrangement

OTO - NOTNLM

OT - adnexal neoplasm

OT - fusion‐driven neoplasia

OT - sarcoma

EDAT- 2024/04/01 00:42

MHDA- 2024/06/11 06:42

CRDT- 2024/03/31 21:03

PHST- 2024/02/22 00:00 [revised]

PHST- 2023/09/27 00:00 [received]

PHST- 2024/03/13 00:00 [accepted]

PHST- 2024/06/11 06:42 [medline]

PHST- 2024/04/01 00:42 [pubmed]

PHST- 2024/03/31 21:03 [entrez]

AID - 10.1111/cup.14610 [doi]

PST - ppublish

SO - J Cutan Pathol. 2024 Jul;51(7):538-548. doi: 10.1111/cup.14610. Epub 2024 Mar 31.

PMID- 38546252

OWN - NLM

STAT- MEDLINE

DCOM- 20240329

LR - 20240411

IS - 0974-5998 (Electronic)

IS - 0189-6725 (Print)

IS - 0974-5998 (Linking)

VI - 21

IP - 2

DP - 2024 Apr 1

TI - Inflammatory Myofibroblastic Tumour in Children: A Report of Two Cases and Review

of Literature.

PG - 134-137

LID - 10.4103/ajps.ajps_106_22 [doi]

AB - Inflammatory myofibroblastic tumour in paediatric patients present with a

diagnostic dilemma because of its clinical, radiological and histopathological

features overlapping with other mesenchymal tumours common in this age. Because

of its rarity, the exact features are still unclear. Here, we are reporting

clinical, radiological and histopathological appearances of two such cases. In

both cases, the exact diagnosis was confirmed only after immunohistochemistry.

There is a need for further detailed study to exactly determine the natural

course and prognosis of these tumours.

CI - Copyright © 2023 Copyright: © 2023 African Journal of Paediatric Surgery.

FAU - Sreepriya, P P

AU - Sreepriya PP

AD - Department of General Surgery, All India Institute of Medical Sciences, Patna,

Bihar, India.

FAU - Ali, Md Mokarram

AU - Ali MM

AD - Department of Paediatric Surgery, Pt B. D. S. PGIMS, Rohtak, Haryana, India.

FAU - Rashi, Rashi

AU - Rashi R

AD - Department of Paediatric Surgery, All India Institute of Medical Sciences, Patna,

Bihar, India.

FAU - Kumar, Amit

AU - Kumar A

AD - Department of Paediatric Surgery, All India Institute of Medical Sciences, Patna,

Bihar, India.

FAU - Sinha, Amit Kumar

AU - Sinha AK

AD - Department of Paediatric Surgery, All India Institute of Medical Sciences, Patna,

Bihar, India.

FAU - Raj, Surabhi

AU - Raj S

AD - Department of Pathology, All India Institute of Medical Sciences, Patna, Bihar,

India.

FAU - Kumar, Bindey

AU - Kumar B

AD - Department of Paediatric Surgery, All India Institute of Medical Sciences, Patna,

Bihar, India.

LA - eng

PT - Case Reports

PT - Journal Article

PT - Review

DEP - 20230214

PL - India

TA - Afr J Paediatr Surg

JT - African journal of paediatric surgery : AJPS

JID - 101255062

SB - IM

MH - Child

MH - Humans

MH - *Neoplasms, Muscle Tissue/diagnosis

PMC - PMC11003571

COIS- There are no conflicts of interest.

EDAT- 2024/03/28 12:46

MHDA- 2024/03/29 06:46

PMCR- 2024/04/01

CRDT- 2024/03/28 09:14

PHST- 2022/08/08 00:00 [received]

PHST- 2022/10/10 00:00 [accepted]

PHST- 2024/03/29 06:46 [medline]

PHST- 2024/03/28 12:46 [pubmed]

PHST- 2024/03/28 09:14 [entrez]

PHST- 2024/04/01 00:00 [pmc-release]

AID - 01434821-202421020-00011 [pii]

AID - AJPS-21-134 [pii]

AID - 10.4103/ajps.ajps_106_22 [doi]

PST - ppublish

SO - Afr J Paediatr Surg. 2024 Apr 1;21(2):134-137. doi: 10.4103/ajps.ajps_106_22.

Epub 2023 Feb 14.

PMID- 38529455

OWN - NLM

STAT- PubMed-not-MEDLINE

LR - 20240327

IS - 2168-8184 (Print)

IS - 2168-8184 (Electronic)

IS - 2168-8184 (Linking)

VI - 16

IP - 2

DP - 2024 Feb

TI - A Huge Abdominal Wall Inflammatory Myofibroblastic Tumor: A Report of a Rare Case

and Literature Review.

PG - e54795

LID - 10.7759/cureus.54795 [doi]

LID - e54795

AB - An inflammatory myofibroblastic tumor (IMT), frequently misdiagnosed as a

malignant neoplasm, is a rare tumor characterized by the presence of

myofibroblastic spindle cells and infiltration of inflammatory cells. In the

current study, a 49-year-old female patient with a huge abdominal mass in the

left abdominal wall was examined. Diagnostic procedures included blood tests, as

well as ultrasound, Doppler, and computed tomography (CT) scans, which revealed

the presence of a huge complex multiloculated cystic lesion measuring 30 x 37

x 20 cm. The patient underwent complete excision of the mass. Histopathological

examination confirmed the benign nature of the tumor and revealed no evidence of

malignancy. A comprehensive review of the available literature shows that the

current case is one of the few documented cases. The report concluded by

emphasizing the importance of surgical intervention as the primary therapeutic

strategy and the crucial role of histopathology in the diagnostic process.

CI - Copyright © 2024, Ali et al.

FAU - Ali, Aqeed A

AU - Ali AA

AD - Biomedical Sciences, Komar University of Science and Technology, Sulaymaniyah,

IRQ.

AD - Surgery, Sulaimani Teaching Hospital, Sulaymaniyah, IRQ.

FAU - Khurshid, Dalshad H

AU - Khurshid DH

AD - Urology, Sulaimani Teaching Hospital, Sulaymaniyah, IRQ.

FAU - Shareef, Farman O

AU - Shareef FO

AD - Medical Laboratory Science, Charmo University, Chamchamal, IRQ.

FAU - Abdul Aziz, Jeza M

AU - Abdul Aziz JM

AD - Baxshin Research Center, Baxshin Hospital, Sulaymaniyah, IRQ.

AD - Biomedical Sciences, Komar University of Science and Technology, Sulaymaniyah,

IRQ.

FAU - Majeed, Nasreen G

AU - Majeed NG

AD - Obstetrics and Gynaecology, Baxshin Hospital, Sulaymaniyah, IRQ.

AD - Nursing, Azmar Technical and Vocational Institute, Sulaymaniyah, IRQ.

AD - Research Center, University of Halabja, Halabja, IRQ.

LA - eng

PT - Case Reports

PT - Journal Article

DEP - 20240223

PL - United States

TA - Cureus

JT - Cureus

JID - 101596737

PMC - PMC10961601

OTO - NOTNLM

OT - abdominal wall mass

OT - inflammatory myofibroblastic tumor

OT - myofibroblastic spindle cells

OT - rare case report

OT - surgical intervention

COIS- The authors have declared that no competing interests exist.

EDAT- 2024/03/26 06:44

MHDA- 2024/03/26 06:45

PMCR- 2024/02/23

CRDT- 2024/03/26 03:45

PHST- 2024/02/23 00:00 [accepted]

PHST- 2024/03/26 06:45 [medline]

PHST- 2024/03/26 06:44 [pubmed]

PHST- 2024/03/26 03:45 [entrez]

PHST- 2024/02/23 00:00 [pmc-release]

AID - 10.7759/cureus.54795 [doi]

PST - epublish

SO - Cureus. 2024 Feb 23;16(2):e54795. doi: 10.7759/cureus.54795. eCollection 2024

Feb.

PMID- 38529377

OWN - NLM

STAT- PubMed-not-MEDLINE

LR - 20240327

IS - 2234-943X (Print)

IS - 2234-943X (Electronic)

IS - 2234-943X (Linking)

VI - 14

DP - 2024

TI - A rare huge bladder inflammatory myofibroblastic tumor treated by en bloc

resection with diode laser: a case report and literature review.

PG - 1327899

LID - 10.3389/fonc.2024.1327899 [doi]

LID - 1327899

AB - BACKGROUND: Inflammatory myofibroblastic tumor (IMT) is a rare neoplasm with

malignant potential. Bladder IMT is even rarer and mainly treated by surgical

resection However, partial or radical cystectomy would affect the quality of life

of patients due to major surgical trauma, and classical TURBT is hard to avoid

intraoperative complications including obturator nerve reflex and bleeding etc.

Therefore, the safe and effective better choice of surgical approaches become

critical to bladder IMT. CASE PRESENTATION: A 42-year-old male patient was

admitted to the department of urology with persistent painless gross hematuria

for more than 10 days without the presentation of hypertension. Preoperative

routine urine examination of red blood cells was 7738.9/HPF (normal range ≤

3/HPF). CTU indicated a space occupying lesion (6.0 cm×5.0 cm) in the left

posterior wall of the bladder with heterogeneous enhancement in the excretory

phase. MRI also indicated bladder tumor with slightly equal SI on T1WI and mixed

high SI on T2WI (6.0 cm×5.1cm×3.5cm) in the left posterior wall of the bladder.

En bloc resection of bladder IMT with 1470 nm diode laser in combination of

removing the enucleated tumor by the morcellator system was performed.

Postoperative pathological examination revealed bladder IMT, with IHC positive

for Ki-67 (15-20%), CK AE1/AE3, SMA, and Desmin of bladder IMT and negative for

ALK of bladder IMT as well as FISH negative for ALK gene rearrangement. Second

TUR with 1470 nm diode laser was performed within 6 weeks to reduce postoperative

risk of recurrence due to highly malignant potential for the high expression of

Ki-67 (15-20%) and negative ALK in IHC staining. The second postoperative

pathology report showed chronic inflammation concomitant with edema of the

bladder mucosa without bladder IMT, furthermore no tumor was observed in

muscularis propria layer of bladder. No recurrence occurred during the period of

24-month follow-up. CONCLUSION: En bloc resection of bladder IMT in combination

of the following second transurethral resection with 1470 nm diode laser is a

safe and effective surgical approach for the huge bladder IMT with highly

malignant potential.

CI - Copyright © 2024 Yuan, Wang, Sun, Chu, Duan and Wang.

FAU - Yuan, Huisheng

AU - Yuan H

AD - Department of Urology, Shandong Provincial Hospital Affiliated to Shandong First

Medical University, Jinan, China.

FAU - Wang, Zilong

AU - Wang Z

AD - Department of Andrology, The Seventh Affiliated Hospital, Sun Yat-sen University,

Shenzhen, China.

AD - Department of Urology, Shandong Provincial Hospital, Cheeloo College of Medicine,

Shandong University, Jinan, China.

FAU - Sun, Jiaxing

AU - Sun J

AD - Department of Urology, Shandong Provincial Hospital Affiliated to Shandong First

Medical University, Jinan, China.

FAU - Chu, Junhao

AU - Chu J

AD - Department of Urology, Shandong Provincial Hospital Affiliated to Shandong First

Medical University, Jinan, China.

FAU - Duan, Shishuai

AU - Duan S

AD - Department of Urology, Shandong Provincial Hospital Affiliated to Shandong First

Medical University, Jinan, China.

FAU - Wang, Muwen

AU - Wang M

AD - Department of Urology, Shandong Provincial Hospital Affiliated to Shandong First

Medical University, Jinan, China.

AD - Department of Urology, Shandong Provincial Hospital, Cheeloo College of Medicine,

Shandong University, Jinan, China.

LA - eng

PT - Case Reports

PT - Journal Article

DEP - 20240311

PL - Switzerland

TA - Front Oncol

JT - Frontiers in oncology

JID - 101568867

PMC - PMC10961466

OTO - NOTNLM

OT - 1470 nm diode laser

OT - bladder cancer

OT - case report

OT - en bloc resection

OT - inflammatory myofibroblastic tumor

OT - second transurethral resection

COIS- The authors declare that the research was conducted in the absence of any

commercial or financial relationships that could be construed as a potential

conflict of interest.

EDAT- 2024/03/26 06:44

MHDA- 2024/03/26 06:45

PMCR- 2024/01/01

CRDT- 2024/03/26 03:44

PHST- 2023/10/25 00:00 [received]

PHST- 2024/02/28 00:00 [accepted]

PHST- 2024/03/26 06:45 [medline]

PHST- 2024/03/26 06:44 [pubmed]

PHST- 2024/03/26 03:44 [entrez]

PHST- 2024/01/01 00:00 [pmc-release]

AID - 10.3389/fonc.2024.1327899 [doi]

PST - epublish

SO - Front Oncol. 2024 Mar 11;14:1327899. doi: 10.3389/fonc.2024.1327899. eCollection

2024.

PMID- 38503969

OWN - NLM

STAT- MEDLINE

DCOM- 20240520

LR - 20250103

IS - 1432-2307 (Electronic)

IS - 0945-6317 (Linking)

VI - 484

IP - 5

DP - 2024 May

TI - Utility of LEF1 to differentiate desmoid fibromatosis from its histologic mimics.

PG - 807-813

LID - 10.1007/s00428-024-03782-z [doi]

AB - Diagnosis of desmoid-type fibromatosis (DF) may be challenging on biopsy due to

morphologic overlap with reactive fibrosis (scar) and other uniform spindle cell

neoplasms. Evaluation of nuclear β-catenin, a surrogate of Wnt pathway

activation, is often difficult in DF due to weak nuclear expression and high

background membranous/cytoplasmic staining. Lymphoid enhancer-factor 1 (LEF1) is

a recently characterized effector partner of β-catenin which activates the

transcription of target genes. We investigated the performance of LEF1 and

β-catenin immunohistochemistry in a retrospective series of 156 soft tissue

tumors, including 35 DF, 3 superficial fibromatosis, and 121 histologic mimics

(19 soft tissue perineurioma, 8 colorectal perineurioma, 4 intraneural

perineurioma, 26 scars, 23 nodular fasciitis, 6 low-grade fibromyxoid sarcomas, 6

angioleiomyomas, 5 neurofibromas, 5 dermatofibrosarcoma protuberans, 3 low-grade

myofibroblastic sarcomas, 3 synovial sarcomas, 3 inflammatory myofibroblastic

tumors, 2 schwannomas, and 1 each of Gardner-associated fibroma,

radiation-associated spindle cell sarcoma, sclerotic fibroma, dermatofibroma, and

glomus tumor). LEF1 expression was not only seen in 33/35 (94%) of DF but also

observed in 19/23 (82%) nodular fasciitis, 7/19 (37%) soft tissue perineurioma,

2/3 (66%) synovial sarcoma, and 6/26 (23%) scar, as well as in 1

radiation-associated spindle cell sarcoma. The sensitivity and specificity of

LEF1 IHC for diagnosis of DF were 94% and 70%, respectively. By comparison,

β-catenin offered similar sensitivity, 94%, but 88% specificity. Positivity for

LEF1 and β-catenin in combination showed sensitivity of 89%, lower than the

sensitivity of β-catenin alone (94%); however, the combination of both LEF1 and

β-catenin improved specificity (96%) compared to the specificity of β-catenin

alone (88%). Although LEF1 has imperfect specificity in isolation, this stain has

diagnostic utility when used in combination with β-catenin.

CI - © 2024. The Author(s), under exclusive licence to Springer-Verlag GmbH Germany,

part of Springer Nature.

FAU - Jobbagy, Soma

AU - Jobbagy S

AUID- ORCID: 0000-0002-6242-5595

AD - Department of Pathology, Massachusetts General Hospital and Harvard Medical

School, WRN2, 55 Fruit St, Boston, MA, 02114, USA.

FAU - Lozano-Calderon, Santiago

AU - Lozano-Calderon S

AUID- ORCID: 0000-0002-0681-9004

AD - Department of Orthopedic Surgery, Massachusetts General Hospital and Harvard

Medical School, Boston, MA, USA.

FAU - Mullen, John T

AU - Mullen JT

AD - Department of Surgical Oncology, Massachusetts General Hospital and Harvard

Medical School, Boston, MA, USA.

FAU - Nielsen, G Petur

AU - Nielsen GP

AUID- ORCID: 0000-0002-9671-7211

AD - Department of Pathology, Massachusetts General Hospital and Harvard Medical

School, WRN2, 55 Fruit St, Boston, MA, 02114, USA.

FAU - Hung, Yin P

AU - Hung YP

AUID- ORCID: 0000-0002-8568-1591

AD - Department of Pathology, Massachusetts General Hospital and Harvard Medical

School, WRN2, 55 Fruit St, Boston, MA, 02114, USA.

FAU - Chebib, Ivan

AU - Chebib I

AUID- ORCID: 0000-0002-6826-0226

AD - Department of Pathology, Massachusetts General Hospital and Harvard Medical

School, WRN2, 55 Fruit St, Boston, MA, 02114, USA. ichebib@mgh.harvard.edu.

LA - eng

PT - Journal Article

DEP - 20240319

PL - Germany

TA - Virchows Arch

JT - Virchows Archiv : an international journal of pathology

JID - 9423843

RN - 0 (beta Catenin)

RN - 0 (Biomarkers, Tumor)

RN - 0 (CTNNB1 protein, human)

RN - 0 (LEF1 protein, human)

RN - 0 (Lymphoid Enhancer-Binding Factor 1)

SB - IM

MH - Female

MH - Humans

MH - Male

MH - *beta Catenin/analysis/metabolism

MH - *Biomarkers, Tumor/analysis

MH - Diagnosis, Differential

MH - *Desmoid Tumors/diagnosis/pathology

MH - *Immunohistochemistry

MH - *Lymphoid Enhancer-Binding Factor 1/analysis

MH - Retrospective Studies

MH - *Soft Tissue Neoplasms/diagnosis/pathology

OTO - NOTNLM

OT - Desmoid-type fibromatosis

OT - Immunohistochemistry

OT - LEF1

OT - Wnt pathway

OT - β-catenin

EDAT- 2024/03/20 06:45

MHDA- 2024/05/20 12:42

CRDT- 2024/03/20 00:29

PHST- 2023/04/12 00:00 [received]

PHST- 2024/03/10 00:00 [accepted]

PHST- 2024/03/05 00:00 [revised]

PHST- 2024/05/20 12:42 [medline]

PHST- 2024/03/20 06:45 [pubmed]

PHST- 2024/03/20 00:29 [entrez]

AID - 10.1007/s00428-024-03782-z [pii]

AID - 10.1007/s00428-024-03782-z [doi]

PST - ppublish

SO - Virchows Arch. 2024 May;484(5):807-813. doi: 10.1007/s00428-024-03782-z. Epub

2024 Mar 19.

PMID- 38485843

OWN - NLM

STAT- PubMed-not-MEDLINE

LR - 20240317

IS - 2198-7793 (Print)

IS - 2198-7793 (Electronic)

IS - 2198-7793 (Linking)

VI - 10

IP - 1

DP - 2024 Mar 15

TI - Gastric inflammatory myofibroblastic tumor: a case report.

PG - 62

LID - 10.1186/s40792-024-01844-7 [doi]

LID - 62

AB - BACKGROUND: Inflammatory myofibroblastic tumor (IMT) of the stomach is an

uncommon mesenchymal neoplasm. We present a case of gastric submucosal tumor

(SMT) where the final diagnosis was IMT. CASE PRESENTATION: A 69-year-old man

presented with a 24-mm SMT on the posterior wall of the middle third of the

stomach that was detected by screening upper gastrointestinal endoscopy.

Abdominal contrast-enhanced computed tomography showed that the tumor was

well-enhanced. Although endoscopic ultrasonography-guided biopsy was performed,

the histological diagnosis was not confirmed preoperatively. Since the tumor was

clinically suspected to be a gastrointestinal stromal tumor, we performed gastric

wedge resection by laparoscopic-endoscopic cooperative surgery. Pathologically,

proliferative spindle cells with a positive reaction for smooth muscle actin,

negativity for c-kit, desmin, s-100, CD34, STAT-6, β-catenin and anaplastic

lymphoma kinase 1 were identified. Hence, the tumor was finally diagnosed as an

IMT originating from the stomach. CONCLUSIONS: When an SMT of the stomach is

identified, the possibility of gastric IMT should be considered.

CI - © 2024. The Author(s).

FAU - Hattori, Taku

AU - Hattori T

AD - Divisions of Gastric Surgery, Shizuoka Cancer Center, 1007 Shimonagakubo,

Nagaizumi-Cho, Sunto-Gun, Shizuoka, 411-8777, Japan.

FAU - Tanizawa, Yutaka

AU - Tanizawa Y

AUID- ORCID: 0000-0002-3685-7300

AD - Divisions of Gastric Surgery, Shizuoka Cancer Center, 1007 Shimonagakubo,

Nagaizumi-Cho, Sunto-Gun, Shizuoka, 411-8777, Japan. y.tanizawa@scchr.jp.

FAU - Shimoda, Tadakazu

AU - Shimoda T

AD - Divisions of Pathology, Shizuoka Cancer Center, 1007 Shimonagakubo,

Nagaizumi-Cho, Sunto-Gun, Shizuoka, 411-8777, Japan.

FAU - Koseki, Yusuke

AU - Koseki Y

AD - Divisions of Gastric Surgery, Shizuoka Cancer Center, 1007 Shimonagakubo,

Nagaizumi-Cho, Sunto-Gun, Shizuoka, 411-8777, Japan.

FAU - Furukawa, Kenichiro

AU - Furukawa K

AD - Divisions of Gastric Surgery, Shizuoka Cancer Center, 1007 Shimonagakubo,

Nagaizumi-Cho, Sunto-Gun, Shizuoka, 411-8777, Japan.

FAU - Fujiya, Keiichi

AU - Fujiya K

AD - Divisions of Gastric Surgery, Shizuoka Cancer Center, 1007 Shimonagakubo,

Nagaizumi-Cho, Sunto-Gun, Shizuoka, 411-8777, Japan.

FAU - Aizawa, Daisuke

AU - Aizawa D

AD - Divisions of Pathology, Shizuoka Cancer Center, 1007 Shimonagakubo,

Nagaizumi-Cho, Sunto-Gun, Shizuoka, 411-8777, Japan.

FAU - Sugino, Takashi

AU - Sugino T

AD - Divisions of Pathology, Shizuoka Cancer Center, 1007 Shimonagakubo,

Nagaizumi-Cho, Sunto-Gun, Shizuoka, 411-8777, Japan.

FAU - Terashima, Masanori

AU - Terashima M

AD - Divisions of Gastric Surgery, Shizuoka Cancer Center, 1007 Shimonagakubo,

Nagaizumi-Cho, Sunto-Gun, Shizuoka, 411-8777, Japan.

FAU - Bando, Etsuro

AU - Bando E

AD - Divisions of Gastric Surgery, Shizuoka Cancer Center, 1007 Shimonagakubo,

Nagaizumi-Cho, Sunto-Gun, Shizuoka, 411-8777, Japan.

LA - eng

PT - Journal Article

DEP - 20240315

PL - Germany

TA - Surg Case Rep

JT - Surgical case reports

JID - 101662125

PMC - PMC10940569

OTO - NOTNLM

OT - Gastric tumor

OT - Inflammatory myofibroblastic tumor

OT - Stomach

OT - Submucosal tumor

COIS- The authors declare no competing interests and did not receive support for the

submitted work from any organization.

EDAT- 2024/03/15 06:43

MHDA- 2024/03/15 06:44

PMCR- 2024/03/15

CRDT- 2024/03/15 00:21

PHST- 2023/11/16 00:00 [received]

PHST- 2024/02/13 00:00 [accepted]

PHST- 2024/03/15 06:44 [medline]

PHST- 2024/03/15 06:43 [pubmed]

PHST- 2024/03/15 00:21 [entrez]

PHST- 2024/03/15 00:00 [pmc-release]

AID - 10.1186/s40792-024-01844-7 [pii]

AID - 1844 [pii]

AID - 10.1186/s40792-024-01844-7 [doi]

PST - epublish

SO - Surg Case Rep. 2024 Mar 15;10(1):62. doi: 10.1186/s40792-024-01844-7.

PMID- 38481869

OWN - NLM

STAT- PubMed-not-MEDLINE

LR - 20240315

IS - 2223-4691 (Print)

IS - 2223-4691 (Electronic)

IS - 2223-4683 (Linking)

VI - 13

IP - 2

DP - 2024 Feb 29

TI - Inflammatory myofibroblastic tumor of the genitourinary tract: a narrative

review.

PG - 308-319

LID - 10.21037/tau-23-471 [doi]

AB - BACKGROUND AND OBJECTIVE: Inflammatory myofibroblastic tumor (IMT) is a rare

entity that is described in several organ systems. This comprehensive review aims

to identify IMTs occurring at various genitourinary (GU) organ sites and describe

patterns of clinical management in adult and pediatric patients. METHODS: A

comprehensive search of PubMed and Web of Science was conducted according to the

Preferred Reporting Items for Systematic Review and meta-analyses statement. Two

reviewers performed independent initial screening of abstracts. Eligible articles

underwent full review and data extraction. The clinical features, diagnostic

tests, treatment, and outcomes at each GU organ site were analyzed individually

and summarized into a comprehensive review. KEY CONTENT AND FINDINGS: Of the 270

articles identified, 112 met inclusion criteria. Articles primarily consisted of

case reports or small series describing a total of 167 cases, of which 30 (18%)

occurred in children. Most patients (96%) were symptomatic at presentation. The

most frequently involved sites included bladder (106 cases) and kidney (n=33)

followed by epididymis (n=6), urachus (n=6), ureter (n=5), prostate (n=4), testis

(n=4), and spermatic cord (n=3). Complete surgical excision of the mass including

partial or total removal of involved organs provided excellent outcomes.

Incomplete excision was associated with early local recurrence and progression.

Late recurrence or metastatic transformation was rarely noted (<2%). CONCLUSIONS:

IMTs exhibit locally invasive, symptomatic and progressive phenotypes that affect

all urologic organs in adults and children. Clinical features and imaging results

are similar to those noted with urologic cancers. These tumors require complete

surgical excision since incomplete resection increases the risk of symptomatic

recurrence.

CI - 2024 Translational Andrology and Urology. All rights reserved.

FAU - Moring, Nikolas

AU - Moring N

AD - Department of Urology, Albany Medical Center, Albany, NY, USA.

FAU - Swerdloff, Daniel

AU - Swerdloff D

AD - Department of Urology, Albany Medical Center, Albany, NY, USA.

FAU - Htoo, Arkar

AU - Htoo A

AD - Department of Pathology, Albany Medical Center, Albany, NY, USA.

FAU - Akgul, Mahmut

AU - Akgul M

AD - Department of Pathology, Albany Medical Center, Albany, NY, USA.

FAU - Nazeer, Tipu

AU - Nazeer T

AD - Department of Pathology, Albany Medical Center, Albany, NY, USA.

FAU - Mian, Badar M

AU - Mian BM

AD - Department of Urology, Albany Medical Center, Albany, NY, USA.

LA - eng

PT - Journal Article

PT - Review

DEP - 20240222

PL - China

TA - Transl Androl Urol

JT - Translational andrology and urology

JID - 101581119

PMC - PMC10932645

OTO - NOTNLM

OT - Anaplastic lymphoma kinase (ALK)

OT - crizotinib

OT - cystectomy

OT - genitourinary neoplasm (GU neoplasm)

OT - inflammatory pseudotumor

COIS- Conflicts of Interest: All authors have completed the ICMJE uniform disclosure

form (available at

https://tau.amegroups.com/article/view/10.21037/tau-23-471/coif). The authors

declare no conflicts of interest.

EDAT- 2024/03/14 06:46

MHDA- 2024/03/14 06:47

PMCR- 2024/02/29

CRDT- 2024/03/14 04:15

PHST- 2023/09/13 00:00 [received]

PHST- 2023/12/10 00:00 [accepted]

PHST- 2024/03/14 06:47 [medline]

PHST- 2024/03/14 06:46 [pubmed]

PHST- 2024/03/14 04:15 [entrez]

PHST- 2024/02/29 00:00 [pmc-release]

AID - tau-13-02-308 [pii]

AID - 10.21037/tau-23-471 [doi]

PST - ppublish

SO - Transl Androl Urol. 2024 Feb 29;13(2):308-319. doi: 10.21037/tau-23-471. Epub

2024 Feb 22.

PMID- 38476732

OWN - NLM

STAT- PubMed-not-MEDLINE

LR - 20240314

IS - 2236-1960 (Print)

IS - 2236-1960 (Electronic)

IS - 2236-1960 (Linking)

VI - 14

DP - 2024

TI - Mesenteric cystic lymphangioma in adults: a rare entity presenting as acute

abdomen - a report of two cases.

PG - e2024470

LID - 10.4322/acr.2024.470 [doi]

LID - e2024470

AB - Lymphangiomas are rare benign tumors that mainly involve the head and neck region

in pediatric patients. Lymphangiomas of the small bowel mesentery in adults are

rarer. We present two cases of mesenteric lymphangioma with acute abdominal pain

on presentation. Case 1: A 38-year-old female presented with abdominal pain,

vomiting, fever, and difficult evacuation. On abdominal examination, she had an

ill-defined, tender lump, and radiological findings raised a possibility of

perforation peritonitis. Thus, exploratory laparotomy was planned.

Per-operatively, a mesenteric mass was found, which, on histopathological

evaluation, was found to be a mesenteric lymphangioma involving the bowel. Case

2: A 27-year-old male presented with abdominal pain and difficult evacuation.

Radiological evaluation revealed a multilobulated lesion involving the mesentery

and with differential diagnoses of mesenteric fibromatoses and inflammatory

pseudotumor. Histopathological assessment of the resected mass revealed a

lymphangioma that was limited to the mesentery. Owing to their rarity and

non-specific presentation, mesenteric lymphangiomas are often misdiagnosed on

clinical examination and imaging. Thus, histopathological examination is the gold

standard to reach a definitive diagnosis.

CI - Copyright © 2024 The Author(s).

FAU - Jha, Tanvi

AU - Jha T

AUID- ORCID: 0000-0001-5494-9324

AD - Atal Bihari Vajpayee Institute of Medical Sciences and Dr. Ram Manohar Lohia

Hospital, Department of Pathology, New Dehli, India.

FAU - Sharma, Monika

AU - Sharma M

AUID- ORCID: 0000-0002-1701-0569

AD - Atal Bihari Vajpayee Institute of Medical Sciences and Dr. Ram Manohar Lohia

Hospital, Department of Pathology, New Dehli, India.

FAU - Ahuja, Arvind

AU - Ahuja A

AUID- ORCID: 0000-0001-7003-5952

AD - Atal Bihari Vajpayee Institute of Medical Sciences and Dr. Ram Manohar Lohia

Hospital, Department of Pathology, New Dehli, India.

LA - eng

PT - Case Reports

PT - Journal Article

DEP - 20240208

PL - Brazil

TA - Autops Case Rep

JT - Autopsy & case reports

JID - 101640070

PMC - PMC10927243

OTO - NOTNLM

OT - Abdomen, Acute

OT - Adult

OT - Lymphangioma

OT - Lymphatics

OT - Mesentery

COIS- Conflict of interest: None.

EDAT- 2024/03/13 06:47

MHDA- 2024/03/13 06:48

PMCR- 2024/02/08

CRDT- 2024/03/13 03:57

PHST- 2023/12/03 00:00 [received]

PHST- 2024/01/08 00:00 [accepted]

PHST- 2024/03/13 06:48 [medline]

PHST- 2024/03/13 06:47 [pubmed]

PHST- 2024/03/13 03:57 [entrez]

PHST- 2024/02/08 00:00 [pmc-release]

AID - acrep192623_EN [pii]

AID - 10.4322/acr.2024.470 [doi]

PST - epublish

SO - Autops Case Rep. 2024 Feb 8;14:e2024470. doi: 10.4322/acr.2024.470. eCollection

2024.

PMID- 38455747

OWN - NLM

STAT- PubMed-not-MEDLINE

LR - 20240309

IS - 2224-4344 (Print)

IS - 2224-4344 (Electronic)

IS - 2224-4336 (Linking)

VI - 13

IP - 2

DP - 2024 Feb 29

TI - Clinical features, treatment strategies, and prognosis of epithelioid

inflammatory myofibroblastic sarcoma in children: a multicenter experience.

PG - 288-299

LID - 10.21037/tp-23-590 [doi]

AB - BACKGROUND: Inflammatory myofibroblastic tumors (IMTs) are a spectrum of tumors

that range in morphology and biological behavior from benign, intermediate, to

apparently malignant and epithelioid inflammatory myofibroblastic sarcoma (EIMS)

is one of the malignant subtypes. This study tried to provide experience and new

ideas for treating this rare disease. METHODS: This study retrospectively

analyzed and followed up 12 children with EIMS admitted to Beijing Children's

Hospital, Baoding Children's Hospital, and Children's Hospital of Chongqing

Medical University from August 2016 to May 2022. RESULTS: Of the 12 children, 7

were male and 5 were female, with a median age of 74.50 [interquartile range

(IQR), 61.50-90.00] months. Of these patients, eight had a single lesion and four

had multiple lesions. The maximum diameter of the single tumor foci was 19.30 cm,

the full meridian of the multiple tumor foci target lesions was 32.67 cm, and the

median maximum tumor size was 11.99 (IQR, 7.80-15.70) cm. The site of disease was

the abdominopelvic cavity in eight cases, the thoracic cavity in two cases, the

maxillofacial region in one case, and the larynx in one case. The clinical

manifestations were predominantly elevated body temperature (n=8). There was one

case of ROS1 fusion mutation and nine cases of ALK fusion mutation. Of the 12

children, 6 were biopsied at the initial diagnosis and 6 were surgically treated.

Follow-up treatment included preoperative neoadjuvant chemotherapy (n=4),

peritoneal thermal perfusion therapy (n=2), targeted therapy (n=3), postoperative

chemotherapy (n=5), and radiotherapy (n=3). The follow-up time was 14.50 (IQR,

10.50-31.50) months, with eight cases of tumor-free survival, two cases of death,

and two cases of loss of follow-up. CONCLUSIONS: EIMS in children is extremely

rare and clinically aggressive. The clinical presentation is nonspecific, and the

initial diagnosis of the tumor is often large. Mutations in the ALK gene are

common in EIMS. Surgery is the mainstay of EIMS treatment, and patients benefit

from a multidisciplinary combination that includes targeted therapies, with

long-term prognosis remaining subject to ongoing follow-up.

CI - 2024 Translational Pediatrics. All rights reserved.

FAU - Cheng, Haiyan

AU - Cheng H

AD - Department of Oncology Surgery, Beijing Children's Hospital, Capital Medical

University, National Center for Children's Health, Beijing, China.

FAU - Lin, Yu

AU - Lin Y

AD - Department of Oncology Surgery, Beijing Children's Hospital, Capital Medical

University, National Center for Children's Health, Beijing, China.

FAU - Zhu, Jin

AU - Zhu J

AD - Department of Pathology, Children's Hospital of Chongqing Medical University,

Chongqing, China.

AD - Department of Pathology, College of Basic Medicine, Chongqing Medical University,

Chongqing, China.

FAU - Qin, Hong

AU - Qin H

AD - Department of Oncology Surgery, Beijing Children's Hospital, Capital Medical

University, National Center for Children's Health, Beijing, China.

FAU - Yang, Wei

AU - Yang W

AD - Department of Oncology Surgery, Beijing Children's Hospital, Capital Medical

University, National Center for Children's Health, Beijing, China.

FAU - Chang, Xiaofeng

AU - Chang X

AD - Department of Oncology Surgery, Beijing Children's Hospital, Capital Medical

University, National Center for Children's Health, Beijing, China.

FAU - Feng, Jun

AU - Feng J

AD - Department of Oncology Surgery, Beijing Children's Hospital, Capital Medical

University, National Center for Children's Health, Beijing, China.

FAU - Yang, Shen

AU - Yang S

AD - Department of Oncology Surgery, Beijing Children's Hospital, Capital Medical

University, National Center for Children's Health, Beijing, China.

FAU - Fu, Libing

AU - Fu L

AD - Department of Pathology, Beijing Children's Hospital, Capital Medical University,

National Center for Children's Health, Beijing, China.

FAU - Zhang, Nan

AU - Zhang N

AD - Department of Pathology, Beijing Children's Hospital, Capital Medical University,

National Center for Children's Health, Beijing, China.

FAU - Shi, Kui

AU - Shi K

AD - Department of Oncology Surgery, Baoding Children's Hospital, Baoding, China.

FAU - Sun, Jian

AU - Sun J

AD - Department of Oncology Surgery, Children's Hospital of Chongqing Medical

University, Chongqing, China.

FAU - Su, Yan

AU - Su Y

AD - Department of Medical Oncology, Beijing Children's Hospital, Capital Medical

University, National Center for Children's Health, Beijing, China.

FAU - Jin, Mei

AU - Jin M

AD - Department of Medical Oncology, Beijing Children's Hospital, Capital Medical

University, National Center for Children's Health, Beijing, China.

FAU - Wang, Shan

AU - Wang S

AD - Department of Oncology Surgery, Children's Hospital of Chongqing Medical

University, Chongqing, China.

FAU - Wang, Huanmin

AU - Wang H

AD - Department of Oncology Surgery, Beijing Children's Hospital, Capital Medical

University, National Center for Children's Health, Beijing, China.

AD - MOE Key Laboratory of Major Diseases in Children, Beijing Children's Hospital,

Capital Medical University, National Center for Children's Health, Beijing,

China.

LA - eng

PT - Journal Article

DEP - 20240226

PL - China

TA - Transl Pediatr

JT - Translational pediatrics

JID - 101649179

PMC - PMC10915437

OTO - NOTNLM

OT - Epithelioid inflammatory myofibroblast sarcoma (EIMS)

OT - children

OT - clinical features

OT - precise treatment

OT - prognosis

COIS- Conflicts of Interest: All authors have completed the ICMJE uniform disclosure

form (available at

https://tp.amegroups.com/article/view/10.21037/tp-23-590/coif). The authors have

no conflicts of interest to declare.

EDAT- 2024/03/08 06:42

MHDA- 2024/03/08 06:43

PMCR- 2024/02/29

CRDT- 2024/03/08 04:04

PHST- 2023/12/07 00:00 [received]

PHST- 2024/01/10 00:00 [accepted]

PHST- 2024/03/08 06:43 [medline]

PHST- 2024/03/08 06:42 [pubmed]

PHST- 2024/03/08 04:04 [entrez]

PHST- 2024/02/29 00:00 [pmc-release]

AID - tp-13-02-288 [pii]

AID - 10.21037/tp-23-590 [doi]

PST - ppublish

SO - Transl Pediatr. 2024 Feb 29;13(2):288-299. doi: 10.21037/tp-23-590. Epub 2024 Feb

26.

PMID- 38382144

OWN - NLM

STAT- PubMed-not-MEDLINE

LR - 20240318

IS - 2210-2612 (Print)

IS - 2210-2612 (Electronic)

IS - 2210-2612 (Linking)

VI - 116

DP - 2024 Mar

TI - Inflammatory myofibroblastic tumor of the bladder turn malignant: A case report.

PG - 109348

LID - S2210-2612(24)00129-9 [pii]

LID - 10.1016/j.ijscr.2024.109348 [doi]

LID - 109348

AB - INTRODUCTION AND IMPORTANCE: Inflammatory myofibroblastic tumor (IMT) of the

bladder is a rare sight, which can be distinguished by the proliferation of

spindle cells and the presence of a persistent chronic inflammatory infiltrate.

IMT is usually benign, but in a few cases it has a tendency for malignant

transformation and metastases. CASE PRESENTATIONS: A 30-year-old male with a

history of recurrent hematuria. His initial symptoms was unfrequent painless

hematuria. Abdomen multislice computerized tomography (MSCT) with contrast shows

an enhancing solid mass with necrotized center measuring +/-

12.9 × 16.5 × 18.9 cm and extending from cavum pelvis to cavum abdomen.

Cystectomy and bilateral ureterocutaneostomy were performed. The histology report

found an IMT with mitotic cells, a necrotic region, and a positive ki67, which

suggest the tumor's malignant transformation. Unfortunately, the patient's

overall condition continued to deteriorate, and he passed away seven days after

hospital discharge. CLINICAL DISCUSSIONS: IMT is comprised of spindel cells and

inflammatory cells. IMT might become aggressive locally, recurring, or progress

to malignancy. Fifty percent of IMTs are caused by rearrangements of the

anaplastic lymphoma kinase (ALK) gene on chromosome 2p23, resulting in ALK-1

overexpression. A change from uniform spindled cells to atypical polygonal cells

or plump cells with oval vesicular nuclei, prominent nucleoli, and mitoses is

indicative of malignant transformation. CONCLUSION: This case emphasizes the

importance of continuous monitoring and raising awareness about the possibility

of malignant transformation of IMT. Understanding the characteristics of the

findings could result in better decision-making and outcomes.

CI - Copyright © 2024 The Authors. Published by Elsevier Ltd.. All rights reserved.

FAU - Djatisoesanto, Wahjoe

AU - Djatisoesanto W

AD - Department of Urology, Faculty of Medicine, Airlangga University, Indonesia.

Electronic address: wahjoe.djatisoesanto@fk.unair.ac.id.

FAU - Yatindra, Ida Bagus Gde Tirta Yoga

AU - Yatindra IBGTY

AD - Department of Urology, Faculty of Medicine, Airlangga University, Indonesia.

FAU - Heryawati

AU - Heryawati

AD - Department of Anatomy Pathology, Faculty of Medicine, Airlangga University,

Indonesia.

FAU - Lesmana, Tomy

AU - Lesmana T

AD - Department of Surgery, Faculty of Medicine, Airlangga University, Indonesia.

LA - eng

PT - Case Reports

PT - Journal Article

DEP - 20240202

PL - Netherlands

TA - Int J Surg Case Rep

JT - International journal of surgery case reports

JID - 101529872

PMC - PMC10943658

OTO - NOTNLM

OT - Case report

OT - Inflamatory Myofibroblastic tumor

OT - Malignant

OT - Transformation

COIS- Conflict of interest statement The authors declare that there are no conflicts of

interest.

EDAT- 2024/02/21 18:43

MHDA- 2024/02/21 18:44

PMCR- 2024/02/02

CRDT- 2024/02/21 18:00

PHST- 2023/12/10 00:00 [received]

PHST- 2024/01/31 00:00 [revised]

PHST- 2024/02/01 00:00 [accepted]

PHST- 2024/02/21 18:44 [medline]

PHST- 2024/02/21 18:43 [pubmed]

PHST- 2024/02/21 18:00 [entrez]

PHST- 2024/02/02 00:00 [pmc-release]

AID - S2210-2612(24)00129-9 [pii]

AID - 109348 [pii]

AID - 10.1016/j.ijscr.2024.109348 [doi]

PST - ppublish

SO - Int J Surg Case Rep. 2024 Mar;116:109348. doi: 10.1016/j.ijscr.2024.109348. Epub

2024 Feb 2.

PMID- 38378420

OWN - NLM

STAT- MEDLINE

DCOM- 20240604

LR - 20240816

IS - 0219-3108 (Electronic)

IS - 1015-9584 (Linking)

VI - 47

IP - 6

DP - 2024 Jun

TI - Synchronous primary minimally invasive adenocarcinoma with inflammatory

myofibroblastic tumor of the lungs in a 9-year-old child.

PG - 2926-2927

LID - S1015-9584(24)00298-7 [pii]

LID - 10.1016/j.asjsur.2024.02.040 [doi]

FAU - Wen, Gang

AU - Wen G

AD - Department of Pediatric Surgery, Affiliated Women and Children's Hospital of

Ningbo University, Ningbo, Zhejiang, China. Electronic address:

nbwengang@163.com.

FAU - Shou, Tiejun

AU - Shou T

AD - Department of Pediatric Surgery, Affiliated Women and Children's Hospital of

Ningbo University, Ningbo, Zhejiang, China.

FAU - Chen, Junxian

AU - Chen J

AD - Department of Pediatric Surgery, Affiliated Women and Children's Hospital of

Ningbo University, Ningbo, Zhejiang, China.

FAU - Song, Lei

AU - Song L

AD - Department of Pediatric Surgery, Affiliated Women and Children's Hospital of

Ningbo University, Ningbo, Zhejiang, China.

LA - eng

PT - Case Reports

PT - Letter

DEP - 20240220

PL - Netherlands

TA - Asian J Surg

JT - Asian journal of surgery

JID - 8900600

SB - IM

MH - Humans

MH - Child

MH - *Lung Neoplasms/surgery/pathology/diagnostic imaging/diagnosis

MH - *Neoplasms, Multiple Primary/surgery/pathology

MH - Male

MH - Adenocarcinoma/surgery/pathology

MH - Neoplasms, Muscle Tissue/surgery/pathology

MH - Pneumonectomy/methods

MH - Tomography, X-Ray Computed

EDAT- 2024/02/21 11:14

MHDA- 2024/06/04 06:42

CRDT- 2024/02/20 21:57

PHST- 2024/01/07 00:00 [received]

PHST- 2024/02/07 00:00 [accepted]

PHST- 2024/06/04 06:42 [medline]

PHST- 2024/02/21 11:14 [pubmed]

PHST- 2024/02/20 21:57 [entrez]

AID - S1015-9584(24)00298-7 [pii]

AID - 10.1016/j.asjsur.2024.02.040 [doi]

PST - ppublish

SO - Asian J Surg. 2024 Jun;47(6):2926-2927. doi: 10.1016/j.asjsur.2024.02.040. Epub

2024 Feb 20.

PMID- 38368344

OWN - NLM

STAT- MEDLINE

DCOM- 20240219

LR - 20240220

IS - 2047-783X (Electronic)

IS - 0949-2321 (Print)

IS - 0949-2321 (Linking)

VI - 29

IP - 1

DP - 2024 Feb 17

TI - A rare inflammatory myofibroblastic tumor appearing both inside and outside the

heart.

PG - 132

LID - 10.1186/s40001-024-01710-0 [doi]

LID - 132

AB - BACKGROUND: Inflammatory myofibroblastic tumor (IMT) is an uncommon cardiac tumor

that primarily affects infants, children, and young adults. While complete

surgical resection generally leads to a favorable prognosis, accurate diagnostic

tests remain limited. CASE PRESENTATION: We describe the case of a 26-year-old

female who had a dual tumor inside and outside the heart and was misdiagnosed by

echocardiography and MRI. We also review 71 cases of cardiac IMTs from the

literature regarding their epidemiology, clinical presentation, and outcome.

CONCLUSION: Early detection of this rare disorder is essential for optimal

surgical management.

CI - © 2024. The Author(s).

FAU - Li, Jiarong

AU - Li J

AD - Department of Cardiovascular Surgery, The Second Xiangya Hospital, Central South

University, Middle Renmin Road 139, Changsha, 410011, China.

FAU - Liu, Jijia

AU - Liu J

AD - Department of Cardiovascular Surgery, The Second Xiangya Hospital, Central South

University, Middle Renmin Road 139, Changsha, 410011, China.

FAU - Yao, Xingwang

AU - Yao X

AD - Clinical Nursing Teaching and Research Section, The Second Xiangya Hospital,

Central South University, Changsha, 410011, China. 739479210@qq.com.

FAU - Yang, Jinfu

AU - Yang J

AD - Department of Cardiovascular Surgery, The Second Xiangya Hospital, Central South

University, Middle Renmin Road 139, Changsha, 410011, China.

yjf19682005@csu.edu.cn.

LA - eng

PT - Case Reports

PT - Journal Article

DEP - 20240217

PL - England

TA - Eur J Med Res

JT - European journal of medical research

JID - 9517857

SB - IM

MH - Child

MH - Infant

MH - Female

MH - Humans

MH - Adult

MH - *Granuloma, Plasma Cell/diagnostic imaging/surgery

MH - *Heart Neoplasms/diagnostic imaging/surgery

MH - Prognosis

MH - Echocardiography

MH - Diagnosis, Differential

PMC - PMC10874007

OTO - NOTNLM

OT - Cardiac tumor

OT - Inflammatory myofibroblastic tumor

OT - Rare

OT - Surgical management

OT - cIMT

COIS- The authors declare that they have no competing interests.

EDAT- 2024/02/18 00:42

MHDA- 2024/02/19 06:42

PMCR- 2024/02/17

CRDT- 2024/02/17 23:14

PHST- 2024/01/08 00:00 [received]

PHST- 2024/02/02 00:00 [accepted]

PHST- 2024/02/19 06:42 [medline]

PHST- 2024/02/18 00:42 [pubmed]

PHST- 2024/02/17 23:14 [entrez]

PHST- 2024/02/17 00:00 [pmc-release]

AID - 10.1186/s40001-024-01710-0 [pii]

AID - 1710 [pii]

AID - 10.1186/s40001-024-01710-0 [doi]

PST - epublish

SO - Eur J Med Res. 2024 Feb 17;29(1):132. doi: 10.1186/s40001-024-01710-0.

PMID- 38344482

OWN - NLM

STAT- PubMed-not-MEDLINE

LR - 20240213

IS - 2168-8184 (Print)

IS - 2168-8184 (Electronic)

IS - 2168-8184 (Linking)

VI - 16

IP - 1

DP - 2024 Jan

TI - Inflammatory Myofibroblastic Tumor With Rapid Recurrence and Distant Metastasis:

Report of a Rare Case.

PG - e52069

LID - 10.7759/cureus.52069 [doi]

LID - e52069

AB - Inflammatory myofibroblastic tumors (IMTs) are rare spindle cell tumors

clinically, morphologically, and genetically heterogeneous, mimicking many other

reactive and neoplastic lesions and creating great diagnostic problems. Although

it is generally characterized by oncogene-derived proliferation of myofibroblasts

in a background of polyclonal inflammatory cell infiltrates, morphological

variations do occur requiring immunohistochemistry and molecular genetics to

confirm the diagnosis. It encompasses a wide age range, and locations, mostly

said to be of intermediate grade having a low risk of recurrence and metastasis.

However, its biological behavior and course are variable and unpredictable. Here,

we report a case of thoracic IMT in a 32-year-old adult female presenting with a

history of fever, cough, and chest pain associated with neutrophilic

leukocytosis. Radiological investigations revealed a large mass in the thoracic

region with possibilities of hydatid cyst and neurogenic tumor. Initial core

needle biopsy specimen and subsequent local resection specimen revealed the

diagnosis of IMT on histopathology and immunohistochemistry, having conventional

morphology with expression of Anaplastic lymphoma kinase (ALK) protein. The

patient developed rapid local recurrence and was started with first-generation

ALK inhibitor Crizotinib. After a brief period of response, she developed

vertebral and brain metastasis within a short span of time and was switched to a

third-generation ALK inhibitor, Lorlatinib. The patient is on regular follow-up,

has stable disease, and maintains a good quality of life after two years of

diagnosis.

CI - Copyright © 2024, Kalita et al.

FAU - Kalita, Dipti

AU - Kalita D

AD - Laboratory Medicine, Histopathology and Cytopathology, Batra Hospital and Medical

Research Centre, New Delhi, IND.

FAU - Rastogi, Ruchi

AU - Rastogi R

AD - Laboratory Medicine, Histopathology and Cytopathology, Batra Hospital and Medical

Research Centre, New Delhi, IND.

FAU - Bhatnagar, Gunmala

AU - Bhatnagar G

AD - Laboratory Medicine, Histopathology and Cytopathology, Batra Hospital and Medical

Research centre, New Delhi, IND.

FAU - Medhi, Kunjahari

AU - Medhi K

AD - Medical Oncology, Batra Hospital and Medical Research Centre, New Delhi, IND.

FAU - Pandey, Sanjay K

AU - Pandey SK

AD - Cardiac/Thoracic/Vascular Surgery, Batra Hospital and Medical Research Centre,

New Delhi, IND.

LA - eng

PT - Case Reports

PT - Journal Article

DEP - 20240110

PL - United States

TA - Cureus

JT - Cureus

JID - 101596737

PMC - PMC10858332

OTO - NOTNLM

OT - alk-inhibitor

OT - brain metastasis

OT - histomorphology of inflammatory myofibroblastic tumor

OT - inflammatory myofibroblastic tumor (imt)

OT - recurrence

COIS- The authors have declared that no competing interests exist.

EDAT- 2024/02/12 15:42

MHDA- 2024/02/12 15:43

PMCR- 2024/01/10

CRDT- 2024/02/12 04:24

PHST- 2024/01/10 00:00 [accepted]

PHST- 2024/02/12 15:43 [medline]

PHST- 2024/02/12 15:42 [pubmed]

PHST- 2024/02/12 04:24 [entrez]

PHST- 2024/01/10 00:00 [pmc-release]

AID - 10.7759/cureus.52069 [doi]

PST - epublish

SO - Cureus. 2024 Jan 10;16(1):e52069. doi: 10.7759/cureus.52069. eCollection 2024

Jan.

PMID- 38339401

OWN - NLM

STAT- PubMed-not-MEDLINE

LR - 20240212

IS - 2072-6694 (Print)

IS - 2072-6694 (Electronic)

IS - 2072-6694 (Linking)

VI - 16

IP - 3

DP - 2024 Feb 2

TI - Anaplastic Lymphoma Kinase (ALK) in Posterior Cranial Fossa Tumors: A Scoping

Review of Diagnostic, Prognostic, and Therapeutic Perspectives.

LID - 10.3390/cancers16030650 [doi]

LID - 650

AB - Anaplastic Lymphoma Kinase (ALK) has been implicated in several human cancers.

This review aims at mapping the available literature on the involvement of ALK in

non-glial tumors localized in the posterior cranial fossa and at identifying

diagnostic, prognostic, and therapeutic considerations. Following the PRISMA-ScR

guidelines, studies were included if they investigated ALK's role in primary CNS,

non-glial tumors located in the posterior cranial fossa. A total of 210

manuscripts were selected for full-text review and 16 finally met the inclusion

criteria. The review included 55 cases of primary, intracranial neoplasms with

ALK genetic alterations and/or protein expression, located in the posterior

fossa, comprising of medulloblastoma, anaplastic large-cell lymphoma,

histiocytosis, inflammatory myofibroblastic tumors, and intracranial myxoid

mesenchymal tumors. ALK pathology was investigated via immunohistochemistry or

genetic analysis. Several studies provided evidence for potential diagnostic and

prognostic value for ALK assessment as well as therapeutic efficacy in its

targeting. The available findings on ALK in posterior fossa tumors are limited.

Nevertheless, previous findings suggest that ALK assessment is of diagnostic and

prognostic value in medulloblastoma (WNT-activated). Interestingly, a substantial

proportion of ALK-positive/altered CNS histiocytoses thus far identified have

been localized in the posterior fossa. The therapeutic potential of ALK

inhibition in histiocytosis warrants further investigation.

FAU - Mousa, Danai-Priskila V

AU - Mousa DV

AD - Department of General Surgery, Penteli Children's Hospital, 15236 Athens, Greece.

FAU - Mavrovounis, Georgios

AU - Mavrovounis G

AD - Department of Neurosurgery, Faculty of Medicine, School of Health Sciences,

University of Thessaly, 41334 Larissa, Greece.

AD - Department of Neurosurgery, Evangelismos Hospital, School of Medicine, Faculty of

Health Sciences, National and Kapodistrian University of Athens, 10676 Athens,

Greece.

FAU - Argyropoulos, Dionysios

AU - Argyropoulos D

AD - Department of Psychiatry, Eginition Hospital, National and Kapodistrian

University of Athens, 11528 Athens, Greece.

FAU - Stranjalis, George

AU - Stranjalis G

AD - Department of Neurosurgery, Evangelismos Hospital, School of Medicine, Faculty of

Health Sciences, National and Kapodistrian University of Athens, 10676 Athens,

Greece.

FAU - Kalamatianos, Theodosis

AU - Kalamatianos T

AD - Department of Neurosurgery, Evangelismos Hospital, School of Medicine, Faculty of

Health Sciences, National and Kapodistrian University of Athens, 10676 Athens,

Greece.

LA - eng

PT - Journal Article

PT - Review

DEP - 20240202

PL - Switzerland

TA - Cancers (Basel)

JT - Cancers

JID - 101526829

PMC - PMC10854950

OTO - NOTNLM

OT - ALK inhibition

OT - Anaplastic Lymphoma Kinase (ALK)

OT - central nervous system

OT - histiocytosis

OT - medulloblastoma

COIS- The authors declare no conflicts of interest.

EDAT- 2024/02/10 10:53

MHDA- 2024/02/10 10:54

PMCR- 2024/02/02

CRDT- 2024/02/10 01:14

PHST- 2023/12/30 00:00 [received]

PHST- 2024/01/25 00:00 [revised]

PHST- 2024/01/29 00:00 [accepted]

PHST- 2024/02/10 10:54 [medline]

PHST- 2024/02/10 10:53 [pubmed]

PHST- 2024/02/10 01:14 [entrez]

PHST- 2024/02/02 00:00 [pmc-release]

AID - cancers16030650 [pii]

AID - cancers-16-00650 [pii]

AID - 10.3390/cancers16030650 [doi]

PST - epublish

SO - Cancers (Basel). 2024 Feb 2;16(3):650. doi: 10.3390/cancers16030650.

PMID- 38333308

OWN - NLM

STAT- PubMed-not-MEDLINE

LR - 20240210

IS - 2049-0801 (Print)

IS - 2049-0801 (Electronic)

IS - 2049-0801 (Linking)

VI - 86

IP - 2

DP - 2024 Feb

TI - Retroperitoneal Castleman's disease in a young Nepalese girl: A rare cause of

childhood abdominal mass.

PG - 1080-1084

LID - 10.1097/MS9.0000000000001579 [doi]

AB - INTRODUCTION: Castleman's disease (CD), or benign angio-follicular lymph node

hyperplasia, is an uncommon condition in childhood. When a child presents with a

huge retroperitoneal mass and inconclusive findings on clinico-radiological

evaluation or tissue sampling, management becomes exceedingly difficult. CD

herein becomes an uncommon presentation of an uncommon diagnosis. CASE

PRESENTATION: A six-year-old girl with no past medical problems presented to the

office with a slowly progressive, painless mass over the right lumbar region for

a year. Abdominal ultrasound showed a well-defined oval mass in the right

periumbilical region, further evaluation of which with a computed tomography scan

suggested lymphoma. A preoperative core-cut biopsy could not confirm the findings

and suggested a neoplastic lesion, probably an inflammatory myofibroblastic

tumour or small round cell tumour. She underwent an exploratory laparotomy with

in-toto excision of the mass. Intraoperatively, a solid retroperitoneal tumour

measuring 8×8×6 cm was found. Histopathology and immunohistochemistry confirmed a

unicentric CD of the hyaline-vascular type. At two years of follow-up, she

remained asymptomatic and disease-free. CONCLUSION: While CD in children is rare,

retroperitoneal localization of the same can further add to the diagnostic

conundrum. However, if carefully considered, an en-bloc surgical resection offers

complete treatment.

CI - Copyright © 2023 The Author(s). Published by Wolters Kluwer Health, Inc.

FAU - Shrestha, Ashish Lal

AU - Shrestha AL

AD - Departments ofPediatric and Neonatal Surgery.

FAU - Mishra, Aakash

AU - Mishra A

AD - Kathmandu Medical College Teaching Hospital, Kathmandu, Nepal.

FAU - Khadka, Sagar

AU - Khadka S

AD - Radiology.

FAU - Dhakhwa, Ramesh

AU - Dhakhwa R

AD - Pathology.

LA - eng

PT - Case Reports

PT - Journal Article

DEP - 20240103

PL - England

TA - Ann Med Surg (Lond)

JT - Annals of medicine and surgery (2012)

JID - 101616869

PMC - PMC10849377

OTO - NOTNLM

OT - Castleman’s disease

OT - children

OT - lymphoproliferative disorder

OT - retroperitoneal tumour

OT - unicentric castleman’s disease

COIS- None of the authors has any conflict of interest to disclose. We confirm that we

have read the Journal’s position on issues involved in ethical publication and

affirm that this report is consistent with those guidelines.Sponsorships or

competing interests that may be relevant to content are disclosed at the end of

this article.

EDAT- 2024/02/09 06:43

MHDA- 2024/02/09 06:44

PMCR- 2024/01/03

CRDT- 2024/02/09 03:57

PHST- 2023/08/16 00:00 [received]

PHST- 2023/11/22 00:00 [accepted]

PHST- 2024/02/09 06:44 [medline]

PHST- 2024/02/09 06:43 [pubmed]

PHST- 2024/02/09 03:57 [entrez]

PHST- 2024/01/03 00:00 [pmc-release]

AID - AMSU-D-23-01754 [pii]

AID - 10.1097/MS9.0000000000001579 [doi]

PST - epublish

SO - Ann Med Surg (Lond). 2024 Jan 3;86(2):1080-1084. doi:

10.1097/MS9.0000000000001579. eCollection 2024 Feb.

PMID- 38317667

OWN - NLM

STAT- PubMed-not-MEDLINE

LR - 20241026

IS - 2050-0904 (Print)

IS - 2050-0904 (Electronic)

IS - 2050-0904 (Linking)

VI - 12

IP - 2

DP - 2024 Feb

TI - IgG4-related inflammatory pancreatic head pseudotumor mirrors pancreatic head

tumor: A novel case series with a review of the literature.

PG - e8467

LID - 10.1002/ccr3.8467 [doi]

LID - e8467

AB - KEY CLINICAL MESSAGE: In this noteworthy case series regarding pancreatic pseudo

tumors, we intend to spread knowledge among physicians for the diagnostic and

therapeutic approach and eventual disease prognosis. ABSTRACT: Inflammatory

pseudotumor of pancreatic head greatly mimics pancreatic head tumor. One of them

is IgG4-related pancreatic disease, which is commonly mistaken as neoplastic

disease on imaging. In our novel case series, we report three cases of

IgG4-related pancreatic head pseudotumor with patients ranging from 35 to

72 years of age. Patients presented with jaundice and abdominal pain. Alongside

initial laboratory workup, abdominal CTs and serum IgG4 levels were also

obtained. Imaging features in conjunction with IgG4 levels confirmed the

diagnosis of IgG4-related autoimmune pancreatitis. Pancreatic pseudotumors are

notorious for being often reported as real tumors. Through our noteworthy case

series, we intend to highlight the imaging features and laboratory markers that

are crucial in such cases to avoid invasive procedures.

CI - © 2024 The Authors. Clinical Case Reports published by John Wiley & Sons Ltd.

FAU - Khan, Faheemullah

AU - Khan F

AUID- ORCID: 0000-0001-6369-743X

AD - Department of Radiology Aga Khan University Hospital Karachi Pakistan.

FAU - Shahid, Jehanzeb

AU - Shahid J

AD - Department of Radiology Aga Khan University Hospital Karachi Pakistan.

FAU - Saleem, Amna

AU - Saleem A

AD - Jinnah Medical and Dental College Karachi Pakistan.

FAU - Khawaja, Uzzam Ahmed

AU - Khawaja UA

AD - Jinnah Medical and Dental College Karachi Pakistan.

FAU - Memon, Wasim Ahmed

AU - Memon WA

AD - Department of Radiology Aga Khan University Hospital Karachi Pakistan.

FAU - Zafar, Uffan

AU - Zafar U

AD - Department of Radiology Aga Khan University Hospital Karachi Pakistan.

FAU - Hameed, Tariq Abdul

AU - Hameed TA

AD - Department of Radiology Indiana University School of Medicine Indianapolis

Indiana USA.

FAU - Abbasher Hussien Mohamed Ahmed, Khabab

AU - Abbasher Hussien Mohamed Ahmed K

AUID- ORCID: 0000-0003-4608-5321

AD - Faculty of Medicine University of Khartoum Khartoum Sudan.

LA - eng

PT - Case Reports

PT - Journal Article

DEP - 20240204

PL - England

TA - Clin Case Rep

JT - Clinical case reports

JID - 101620385

PMC - PMC10839121

OTO - NOTNLM

OT - abdominal CT

OT - pancreatic tumor

OT - pseud tumor

OT - serum IgG4

COIS- None to declare.

EDAT- 2024/02/06 06:43

MHDA- 2024/02/06 06:44

PMCR- 2024/02/04

CRDT- 2024/02/06 03:45

PHST- 2023/04/04 00:00 [received]

PHST- 2024/01/12 00:00 [revised]

PHST- 2024/01/19 00:00 [accepted]

PHST- 2024/02/06 06:44 [medline]

PHST- 2024/02/06 06:43 [pubmed]

PHST- 2024/02/06 03:45 [entrez]

PHST- 2024/02/04 00:00 [pmc-release]

AID - CCR38467 [pii]

AID - 10.1002/ccr3.8467 [doi]

PST - epublish

SO - Clin Case Rep. 2024 Feb 4;12(2):e8467. doi: 10.1002/ccr3.8467. eCollection 2024

Feb.

PMID- 38313745

OWN - NLM

STAT- PubMed-not-MEDLINE

LR - 20240206

IS - 2432-3853 (Electronic)

IS - 2432-3853 (Linking)

VI - 8

IP - 1

DP - 2024

TI - Inflammatory Myofibroblastic Tumor of the Anus: A Case Report.

PG - 39-42

LID - 10.23922/jarc.2022-043 [doi]

AB - Inflammatory myofibroblastic tumors (IMTs) are neoplastic lesions characterized

by the proliferation of spindle cells with myofibroblastic features and

lymphocyte infiltration. Primary lesions can develop in several locations but

rarely arise in the colon as described herein. The present case was that of a

69-year-old woman who visited our hospital with complaints of bloody bowel

discharge and a prolapsed mass from the anus. A 20-mm tumor was identified on

visual and digital examination. Lower gastrointestinal endoscopy revealed a

pedunculated, elevated lesion above the dentate line, which showed contrast

enhancement on abdominal computed tomography. The patient was preoperatively

diagnosed with an anal polyp, which was resected transanally. During the

procedure, a mobile tumor coated by anal epithelium was observed at the 11

o'clock position above the dentate line. Deeper parts of the tumor were

contiguous with the internal anal sphincter (IAS) muscle. Suspecting a neoplastic

lesion, we resected the mass en bloc with part of the IAS. Tumor histopathology

after surgery led to a final diagnosis of an IMT of the anus. IMT is difficult to

diagnose preoperatively. No adjuvant therapy has been formally established; thus,

an adequate surgical margin and close monitoring are essential.

CI - Copyright © 2024 The Japan Society of Coloproctology.

FAU - Takayama, Tetsuyoshi

AU - Takayama T

AD - Department of General Surgery, Saitama Medical University, Saitama, Japan.

FAU - Nakame, Ayako

AU - Nakame A

AD - Department of General Surgery, Saitama Medical University, Saitama, Japan.

FAU - Suzuki, Masaomi

AU - Suzuki M

AD - Department of General Surgery, Saitama Medical University, Saitama, Japan.

FAU - Asano, Hiroshi

AU - Asano H

AD - Department of General Surgery, Saitama Medical University, Saitama, Japan.

FAU - Jin, Ling

AU - Jin L

AD - Department of Pathology, Saitama Medical University, Saitama, Japan.

LA - eng

PT - Case Reports

PT - Journal Article

DEP - 20240125

PL - Japan

TA - J Anus Rectum Colon

JT - Journal of the anus, rectum and colon

JID - 101718055

PMC - PMC10831981

OTO - NOTNLM

OT - anaplastic lymphoma kinase

OT - case report

OT - inflammatory myofibroblastic tumor

OT - transanal tumor resection

COIS- Conflicts of Interest There are no conflicts of interest.

EDAT- 2024/02/05 06:43

MHDA- 2024/02/05 06:44

PMCR- 2024/01/25

CRDT- 2024/02/05 04:49

PHST- 2023/06/19 00:00 [received]

PHST- 2023/11/08 00:00 [accepted]

PHST- 2024/02/05 06:44 [medline]

PHST- 2024/02/05 06:43 [pubmed]

PHST- 2024/02/05 04:49 [entrez]

PHST- 2024/01/25 00:00 [pmc-release]

AID - 10.23922/jarc.2022-043 [doi]

PST - epublish

SO - J Anus Rectum Colon. 2024 Jan 25;8(1):39-42. doi: 10.23922/jarc.2022-043.

eCollection 2024.

PMID- 38304501

OWN - NLM

STAT- PubMed-not-MEDLINE

LR - 20241023

IS - 0973-029X (Print)

IS - 1998-393X (Electronic)

IS - 0973-029X (Linking)

VI - 27

IP - 4

DP - 2023 Oct-Dec

TI - A multicentric case study of fibroblastic and myofibroblastic oral spindle cell

lesions.

PG - 629-641

LID - 10.4103/jomfp.jomfp_282_23 [doi]

AB - CONTEXT: Spindle cell lesions comprise a vast plethora of benign and malignant

lesions with similar clinical and radiographic features. Their overlapping

histopathologic features ensure a diagnostic dilemma. AIM: The current

multicentric study aims to delineate fibroblastic and myofibroblastic oral

spindle cell lesions based on cytomorphology and comprehensive

immunohistochemical analysis. SETTINGS AND DESIGN: The experimental study was

conducted at MS Ramaiah University of Applied Sciences, Bangalore, and All India

Institute of Applied Sciences, Delhi. METHODS AND MATERIAL: A comprehensive

histological scoring criteria and panel of immunohistochemical makers (STAT6,

CD31, CD34, S100, SMA, vimentin, pan-CK, HHF-35, Ki67, ALK, desmin, HMB-45,

SATB2, ERG, EMA and CD99) were employed concurrently for the first time for

fibroblastic and myofibroblastic oral spindle cell lesions. The data obtained was

tabulated and studied. STATISTICAL ANALYSIS USED: NA. Results: Using cytological

scoring criteria and panel of immunohistochemical makers, the cases analysed and

characterized were desmoplastic fibroma, fibrosarcoma, leiomyosarcoma, nodular

fasciitis, neurofibroma and epithelioid inflammatory myofibroblastic sarcoma

(EIMS). CONCLUSIONS: The diagnostic strategies need to be upgraded for the

diagnosis of spindle cell lesions. Emphasis must be placed on cytomorphology, an

immunohistochemistry (IHC) panel of markers is imperative for the accurate

diagnosis of fibroblastic and myofibroblastic oral spindle cell lesions.

CI - Copyright: © 2023 Journal of Oral and Maxillofacial Pathology.

FAU - Jot, Kiran

AU - Jot K

AD - Department of Oral Pathology and Microbiology, Fifth Floor, Centre for Dental

Education and Research, All India Institute of Medical Sciences, Ansari Nagar,

New Delhi, India.

FAU - Nayyar, Vivek

AU - Nayyar V

AD - Department of Oral Pathology and Microbiology, Fifth Floor, Centre for Dental

Education and Research, All India Institute of Medical Sciences, Ansari Nagar,

New Delhi, India.

FAU - Surya, Varun

AU - Surya V

AD - Department of Oral Pathology and Microbiology, Fifth Floor, Centre for Dental

Education and Research, All India Institute of Medical Sciences, Ansari Nagar,

New Delhi, India.

FAU - Mishra, Deepika

AU - Mishra D

AD - Department of Oral Pathology and Microbiology, Fifth Floor, Centre for Dental

Education and Research, All India Institute of Medical Sciences, Ansari Nagar,

New Delhi, India.

FAU - Sowmya, S V

AU - Sowmya SV

AD - Department of Oral Pathology and Microbiology, Faculty of Dental Sciences,

Ramaiah University of Applied Sciences, MSR Nagar, Bengaluru, Karnataka, India.

FAU - Augustine, Dominic

AU - Augustine D

AD - Department of Oral Pathology and Microbiology, Faculty of Dental Sciences,

Ramaiah University of Applied Sciences, MSR Nagar, Bengaluru, Karnataka, India.

FAU - Indu, M

AU - Indu M

AD - Department of Oral Pathology, Government Dental College, Kottayam, Kerala, India.

FAU - Haragannavar, Vanishri C

AU - Haragannavar VC

AD - Department of Oral Pathology and Microbiology, Faculty of Dental Sciences,

Ramaiah University of Applied Sciences, MSR Nagar, Bengaluru, Karnataka, India.

LA - eng

PT - Journal Article

DEP - 20231220

PL - India

TA - J Oral Maxillofac Pathol

JT - Journal of oral and maxillofacial pathology : JOMFP

JID - 101227995

PMC - PMC10829460

OTO - NOTNLM

OT - Fibroblastic

OT - immunohistochemistry

OT - multicentric

OT - myofibroblastic origin

OT - spindle cell

COIS- There are no conflicts of interest.

EDAT- 2024/02/02 06:43

MHDA- 2024/02/02 06:44

PMCR- 2023/10/01

CRDT- 2024/02/02 04:08

PHST- 2023/06/26 00:00 [received]

PHST- 2023/11/18 00:00 [revised]

PHST- 2023/11/20 00:00 [accepted]

PHST- 2024/02/02 06:44 [medline]

PHST- 2024/02/02 06:43 [pubmed]

PHST- 2024/02/02 04:08 [entrez]

PHST- 2023/10/01 00:00 [pmc-release]

AID - JOMFP-27-629 [pii]

AID - 10.4103/jomfp.jomfp_282_23 [doi]

PST - ppublish

SO - J Oral Maxillofac Pathol. 2023 Oct-Dec;27(4):629-641. doi:

10.4103/jomfp.jomfp_282_23. Epub 2023 Dec 20.

PMID- 38303518

OWN - NLM

STAT- MEDLINE

DCOM- 20240924

LR - 20241001

IS - 1940-2465 (Electronic)

IS - 1066-8969 (Linking)

VI - 32

IP - 7

DP - 2024 Oct

TI - ALK-Rearranged Epithelioid and Spindle Cell Neoplasm of the Sinonasal Tract.

PG - 1332-1338

LID - 10.1177/10668969241226699 [doi]

AB - Anaplastic lymphoma kinase (ALK)-rearranged mesenchymal neoplasms

(non-inflammatory myofibroblastic tumor and non-epithelioid fibrous histiocytoma)

have been recently described which tend to occur in the superficial and deep soft

tissues. Occurrence as a primary sinonasal neoplasm has not been reported thus

far. Herein, we describe the first case of sinonasal ALK-rearranged mesenchymal

tumor that harbored remarkable epithelioid and spindle cell morphology. The tumor

affected a 40-year-old man who presented with flu-like symptoms and was thought

to have influenza A. However, computed tomography demonstrated a nasal polypoid

lesion causing curvature of the nasal septum. Histological examination revealed a

heterogeneous tumor composed of round to epithelioid cells with foci of spindle

cells. The tumor cells exhibited moderate pleomorphism and mitotic activity. By

immunohistochemistry, they showed diffuse staining of CD34, S100, ALK (D5F3) and

CD30. Fluorescence in situ hybridization analysis demonstrated ALK rearrangement.

Subsequent next-generation sequencing (RNA-seq) identified a rare

PLEKHH2exon6::ALKexon20 fusion. This study further demonstrates the importance of

molecular profiling in identifying kinase fusion-positive soft tissue tumors,

particularly for those that arise at unusual sites and display atypical

cytomorphology.

FAU - Zhu, Peipei

AU - Zhu P

AD - Department of Pathology, Fudan University Shanghai Cancer Center, Shanghai,

China. RINGGOLD: 89667

AD - Department of Oncology, Shanghai Medical College, Fudan University, Shanghai,

China. RINGGOLD: 58305

FAU - Wang, Jian

AU - Wang J

AUID- ORCID: 0000-0001-7175-9856

AD - Department of Pathology, Fudan University Shanghai Cancer Center, Shanghai,

China. RINGGOLD: 89667

AD - Department of Oncology, Shanghai Medical College, Fudan University, Shanghai,

China. RINGGOLD: 58305

LA - eng

PT - Case Reports

PT - Journal Article

DEP - 20240201

PL - United States

TA - Int J Surg Pathol

JT - International journal of surgical pathology

JID - 9314927

RN - EC 2.7.10.1 (Anaplastic Lymphoma Kinase)

RN - EC 2.7.10.1 (ALK protein, human)

RN - 0 (Biomarkers, Tumor)

SB - IM

MH - Humans

MH - Male

MH - *Anaplastic Lymphoma Kinase/genetics/metabolism

MH - Adult

MH - *Gene Rearrangement

MH - Biomarkers, Tumor/genetics/analysis/metabolism

MH - Paranasal Sinus Neoplasms/pathology/genetics/diagnosis

MH - Epithelioid Cells/pathology

MH - In Situ Hybridization, Fluorescence

MH - Nose Neoplasms/pathology/genetics/diagnosis

OTO - NOTNLM

OT - ALK::PLEKHH2 fusion transcript

OT - CD34 and S100 co-expression

OT - epithelioid and spindle cell neoplasm

OT - nasal cavity

COIS- Declaration of Conflicting InterestsThe authors declared no potential conflicts

of interest with respect to the research, authorship, and/or publication of this

article.

EDAT- 2024/02/02 06:43

MHDA- 2024/09/24 22:18

CRDT- 2024/02/02 02:12

PHST- 2024/09/24 22:18 [medline]

PHST- 2024/02/02 06:43 [pubmed]

PHST- 2024/02/02 02:12 [entrez]

AID - 10.1177/10668969241226699 [doi]

PST - ppublish

SO - Int J Surg Pathol. 2024 Oct;32(7):1332-1338. doi: 10.1177/10668969241226699. Epub

2024 Feb 1.

PMID- 38303312

OWN - NLM

STAT- MEDLINE

DCOM- 20240209

LR - 20240209

IS - 0385-0684 (Print)

IS - 0385-0684 (Linking)

VI - 50

IP - 13

DP - 2023 Dec

TI - [A Case of Right Hemicolectomy for Inflammatory Myofibroblastic Tumor Arising in

the Right Transverse Colon].

PG - 1474-1476

AB - The patient was a 21-year-old male. He presented with right lower abdominal pain

and showed tenderness in the same area. An abdominal contrast-enhanced CT

examination revealed a 45 mm tumor that continuously stained in the right

transverse colon. The patient presented with tenderness and rebound tenderness.

Due to a suspected submucosal tumor, laparotomy was performed and an elastic hard

tumor of 5 cm in size was found on the serous membrane side of the right

transverse colon. As malignancy could not be ruled out, a right hemicolectomy

with lymph node dissection was performed. The pathological diagnosis was an

inflammatory myofibroblastic tumor(IMT), characterized by the proliferation of

spindle- shaped spindle-shaped fibroblast-like cells with inflammatory cell

infiltration. As of 9 years post-surgery, there has been no recurrence. However,

long-term surveillance is necessary.

FAU - Takeda, Takashi

AU - Takeda T

AD - Dept. of Surgery, Minoh City Hospital.

FAU - Takahashi, Kenta

AU - Takahashi K

FAU - Danno, Katsuki

AU - Danno K

FAU - Nakamichi, Itsuko

AU - Nakamichi I

FAU - Takada, Naoya

AU - Takada N

FAU - Murao, Shuhei

AU - Murao S

FAU - Yamamoto, Kei

AU - Yamamoto K

FAU - Higashiguchi, Masaya

AU - Higashiguchi M

FAU - Noguchi, Kozo

AU - Noguchi K

FAU - Toyoda, Yasuhiro

AU - Toyoda Y

FAU - Nakane, Shigeru

AU - Nakane S

FAU - Yamamoto, Hitoshi

AU - Yamamoto H

FAU - Hirao, Takafumi

AU - Hirao T

FAU - Oka, Yoshio

AU - Oka Y

LA - jpn

PT - Case Reports

PT - English Abstract

PT - Journal Article

PL - Japan

TA - Gan To Kagaku Ryoho

JT - Gan to kagaku ryoho. Cancer & chemotherapy

JID - 7810034

SB - IM

MH - Humans

MH - Male

MH - Young Adult

MH - Colectomy

MH - *Colon, Transverse/surgery/pathology

MH - Lymph Node Excision

EDAT- 2024/02/02 06:43

MHDA- 2024/02/05 06:43

CRDT- 2024/02/02 01:01

PHST- 2024/02/05 06:43 [medline]

PHST- 2024/02/02 06:43 [pubmed]

PHST- 2024/02/02 01:01 [entrez]

PST - ppublish

SO - Gan To Kagaku Ryoho. 2023 Dec;50(13):1474-1476.

PMID- 38288137

OWN - NLM

STAT- PubMed-not-MEDLINE

LR - 20241023

IS - 2213-0071 (Print)

IS - 2213-0071 (Electronic)

IS - 2213-0071 (Linking)

VI - 47

DP - 2024

TI - Traumatic tumor hemorrhage of inflammatory myofibroblastic tumor of the lung.

PG - 101981

LID - 10.1016/j.rmcr.2024.101981 [doi]

LID - 101981

AB - A 23-year-old female with a history of idiopathic epilepsy was found to have a

right chest cavity shadow in a school health checkup 5 years before. CT revealed

a thin-walled cavity lesion in the right middle lobe containing a ball-like mass,

showing air crescent sign. After falling due to a seizure, she was transported by

ambulance and admitted. CT revealed diffuse ground-glass opacities throughout the

right lung field. Bronchoscopy revealed bloody bronchial alveolar lavage fluid.

Due to the tumor hemorrhage, an elective simple right middle lobe resection was

performed without complications. The initial immunohistochemical staining was

negative for ALK using ALK1 clone; however, subsequent staining of ALK by D5F3

and 5A4 clone was positive. Immunostaining findings led to a diagnosis of

inflammatory myofibroblastic tumor. The patient remains under regular observation

and has experienced no recurrence over the 6-year postoperative period. This case

contains two different points: the first is that a cavity lesion of inflammatory

myofibroblastic tumor may cause traumatic bleeding and should be treated with

caution; the second is that attention should be paid to differences in

stainability among clones when diagnosing inflammatory myofibroblastic tumor.

CI - © 2024 The Authors.

FAU - Yamashita, Takashi

AU - Yamashita T

AD - Department of Thoracic Surgery, Iwata City Hospital, 512-3, Ohkubo, Iwata,

Shizuoka, 438-8550, Japan.

FAU - Matsubayashi, Yuta

AU - Matsubayashi Y

AD - Department of Thoracic Surgery, Iwata City Hospital, 512-3, Ohkubo, Iwata,

Shizuoka, 438-8550, Japan.

FAU - Mochizuki, Takahiro

AU - Mochizuki T

AD - Department of Thoracic Surgery, Iwata City Hospital, 512-3, Ohkubo, Iwata,

Shizuoka, 438-8550, Japan.

LA - eng

PT - Case Reports

PT - Journal Article

DEP - 20240112

PL - England

TA - Respir Med Case Rep

JT - Respiratory medicine case reports

JID - 101604463

PMC - PMC10823134

OTO - NOTNLM

OT - Anaplastic lymphoma kinase (ALK)

OT - Hemorrhage

OT - Inflammatory myofibroblastic tumor (IMT)

OT - Lung

OT - Trauma

COIS- The authors declare that they have no known competing financial interests or

personal relationships that could have appeared to influence the work reported in

this paper.

EDAT- 2024/01/30 06:42

MHDA- 2024/01/30 06:43

PMCR- 2024/01/12

CRDT- 2024/01/30 03:38

PHST- 2023/08/30 00:00 [received]

PHST- 2023/12/31 00:00 [revised]

PHST- 2024/01/08 00:00 [accepted]

PHST- 2024/01/30 06:43 [medline]

PHST- 2024/01/30 06:42 [pubmed]

PHST- 2024/01/30 03:38 [entrez]

PHST- 2024/01/12 00:00 [pmc-release]

AID - S2213-0071(24)00004-2 [pii]

AID - 101981 [pii]

AID - 10.1016/j.rmcr.2024.101981 [doi]

PST - epublish

SO - Respir Med Case Rep. 2024 Jan 12;47:101981. doi: 10.1016/j.rmcr.2024.101981.

eCollection 2024.

PMID- 38287440

OWN - NLM

STAT- MEDLINE

DCOM- 20240131

LR - 20241023

IS - 1749-8090 (Electronic)

IS - 1749-8090 (Linking)

VI - 19

IP - 1

DP - 2024 Jan 29

TI - Gastric and cardiac inflammatory myofibroblastic tumor: an extremely rare case.

PG - 31

LID - 10.1186/s13019-024-02481-9 [doi]

LID - 31

AB - BACKGROUND: Inflammatory myofibroblastic tumor (IMT) is a unique, rarely

metastatic tumor composed of myofibroblasts and fibrous spindle cells with

inflammatory cell infiltration that can affect any organ in the human body. By

reviewing the relevant literature on PubMed, we found that this is the first case

report of IMT with both gastric and cardiac involvement. CASE PRESENTATION: A

57-year-old male patient was admitted to the hospital with complaints of malaise,

poor appetite, and epigastric pain with black stools. We found a mass in the

patient's stomach and left atrium by contrast-enhanced computed tomography,

18 F-fluorodeoxyglucose positron emission tomography/computed tomography, and

other tests. The patient underwent laparoscopic Billroth II subtotal gastrectomy

and Braun's gastrointestinal reconstruction under general anesthesia. On the 46th

day following stomach surgery, the cardiac tumor was removed under general

anesthesia. The patient has treated with doxorubicin 70 mg of D1 chemotherapy two

months after cardiac surgery. Postoperative pathological immunohistochemistry of

the mass confirmed the diagnosis of an IMT. His review three months after the

cardiac surgery suggested the progression of the left atrial mass, but he

declined further treatment and finally died one month after the review.

CONCLUSIONS: As a unique class of tumors that rarely metastasize, IMTs have an

unknown etiology and pathogenesis, and distant metastasis is primarily observed

in patients with negative activin receptor-like kinase (ALK) expression. The

preferred treatment for IMT is complete surgical resection, and the effectiveness

of adjuvant therapy for patients with distant metastases is still being

determined. The clinical presentation of IMT lacks specificity and is often

related to the location of tumor growth, which poses a diagnostic challenge.

Pathological immunohistochemistry is the only way to confirm the diagnosis at

present. Our case report reminds clinicians that a category of ALK-negative IMT

with a tendency toward distant metastasis should not be ignored.

CI - © 2024. The Author(s).

FAU - Huang, Yueqi

AU - Huang Y

AD - Department of Ultrasound Medicine, The First Affiliated Hospital, Hengyang

Medical School, University of South China, Hengyang, 421001, Hunan, People's

Republic of China.

FAU - Zhang, Mingqi

AU - Zhang M

AD - Department of General Surgery, The First Affiliated Hospital, Hengyang Medical

School, University of South China, Hengyang, 421001, Hunan, People's Republic of

China.

FAU - Li, Qingchun

AU - Li Q

AD - Department of Image, The First Affiliated Hospital, Hengyang Medical School,

University of South China, Hengyang, 421001, Hunan, People's Republic of China.

FAU - Huang, Qiulin

AU - Huang Q

AD - Department of General Surgery, The First Affiliated Hospital, Hengyang Medical

School, University of South China, Hengyang, 421001, Hunan, People's Republic of

China. 2018011993@usc.edu.cn.

LA - eng

GR - 2020JJ6055/the Natural Science Foundation of Hunan Province, China/

GR - 2020JJ6055/the Natural Science Foundation of Hunan Province, China/

GR - 2021JJ70118/the Natural Science Foundation of Hunan Province, China/

GR - 2020JJ6055/the Natural Science Foundation of Hunan Province, China/

PT - Case Reports

PT - Journal Article

DEP - 20240129

PL - England

TA - J Cardiothorac Surg

JT - Journal of cardiothoracic surgery

JID - 101265113

RN - EC 2.7.10.1 (Anaplastic Lymphoma Kinase)

SB - IM

MH - Male

MH - Humans

MH - Middle Aged

MH - Anaplastic Lymphoma Kinase

MH - Stomach

MH - *Laparoscopy

MH - *Heart Neoplasms/diagnosis/surgery

PMC - PMC10823716

OTO - NOTNLM

OT - Activin receptor-like kinase (ALK)

OT - Cardiac

OT - Gastric

OT - Inflammatory myofibroblastic tumor (IMT)

OT - Prognosis

COIS- The authors declare no competing interests.

EDAT- 2024/01/30 06:42

MHDA- 2024/01/31 06:42

PMCR- 2024/01/29

CRDT- 2024/01/30 00:03

PHST- 2023/04/12 00:00 [received]

PHST- 2024/01/14 00:00 [accepted]

PHST- 2024/01/31 06:42 [medline]

PHST- 2024/01/30 06:42 [pubmed]

PHST- 2024/01/30 00:03 [entrez]

PHST- 2024/01/29 00:00 [pmc-release]

AID - 10.1186/s13019-024-02481-9 [pii]

AID - 2481 [pii]

AID - 10.1186/s13019-024-02481-9 [doi]

PST - epublish

SO - J Cardiothorac Surg. 2024 Jan 29;19(1):31. doi: 10.1186/s13019-024-02481-9.

PMID- 38261452

OWN - NLM

STAT- MEDLINE

DCOM- 20240718

LR - 20241121

IS - 1998-4138 (Electronic)

IS - 1998-4138 (Linking)

VI - 20

IP - 3

DP - 2024 Apr 1

TI - Inflammatory myofibroblastic disease of right petrous apex: A rare case with

review of literature.

PG - 1077-1080

LID - 10.4103/jcrt.JCRT_1451_20 [doi]

AB - Inflammatory myofibroblastic tumor (IMFT) is a rare tumor of unknown etiology. It

can involve any part of the body. The IMFT involving the base of skull is rare

with only 36 cases reported in the literature. We report a rare case of IMFT of

temporal bone with review of literature. A 42 year old male presented with

complaints of headache and double vision and MRI brain showed lesion in the right

petrous apex region suggestive of a neurogenic mass. He had excision of lesion

and histopathology was suggestive of IMFT with IgG4 and ALK positive. He had

complete clinical response but a month later he presented with right eyelid

ptosis and decreased rotation of eye medially with recurrent lesion on MRI.

Patient received radiation by SRT technique and then started on Ceretinib with

partial response. The IMFT is rare tumor of unknown etiology and tumors of

temporal bone are more aggressive. It is benign but locally invasive tumor.

Treatment of IMFT is controversial. Extensive surgery with complete excision has

about 80% response rates and with intracranial extension, adjuvant radiation is

need. In head and neck IMFT response rates are lower (30 to 40%). Monoclonal

antibodies and steroids are used in IMFT at recurrence. In advanced or metastatic

ALK positive tumors, Crizotinib is used with a response rate of 50%. Radiotherapy

(25 to 30 Gy) induces remission and helps to taper the steroids. Temporal bone

IMFT is a rare tumor with multimodality approach and variable response to

treatment.

CI - Copyright © 2024 Copyright: © 2024 Journal of Cancer Research and Therapeutics.

FAU - Mahadev, Doddala Sankara

AU - Mahadev DS

AD - Nuclear Medicine, King George Hospital, Andhra Medical College, Visakhapatnam,

Andhra Pradesh, India.

FAU - Praveen, N V S

AU - Praveen NVS

AD - Consultant Radiation Oncologist, Omega Hospital, Guntur, Andhra Pradesh, India.

FAU - Suryadevara, Aparna

AU - Suryadevara A

AD - Department of Radiation Oncology, Mehdi Nawaz Jung Institute of Oncology and

Regional Cancer Centre, Hyderabad, Telangana, India.

FAU - Naga Kishore, M G

AU - Naga Kishore MG

AD - Consultant Surgical Oncologist, Omega Hospital, Guntur, Andhra Pradesh, India.

LA - eng

PT - Case Reports

PT - Letter

PT - Review

DEP - 20240122

PL - India

TA - J Cancer Res Ther

JT - Journal of cancer research and therapeutics

JID - 101249598

SB - IM

MH - Humans

MH - Male

MH - Adult

MH - *Granuloma, Plasma Cell/diagnosis/pathology/surgery/diagnostic imaging/therapy

MH - Magnetic Resonance Imaging

MH - Petrous Bone/pathology/diagnostic imaging/surgery

EDAT- 2024/01/23 18:42

MHDA- 2024/07/18 12:43

CRDT- 2024/01/23 12:23

PHST- 2020/10/02 00:00 [received]

PHST- 2023/01/12 00:00 [accepted]

PHST- 2024/07/18 12:43 [medline]

PHST- 2024/01/23 18:42 [pubmed]

PHST- 2024/01/23 12:23 [entrez]

AID - 01363817-990000000-00069 [pii]

AID - 10.4103/jcrt.JCRT_1451_20 [doi]

PST - ppublish

SO - J Cancer Res Ther. 2024 Apr 1;20(3):1077-1080. doi: 10.4103/jcrt.JCRT_1451_20.

Epub 2024 Jan 22.

PMID- 38254862

OWN - NLM

STAT- PubMed-not-MEDLINE

LR - 20241023

IS - 2072-6694 (Print)

IS - 2072-6694 (Electronic)

IS - 2072-6694 (Linking)

VI - 16

IP - 2

DP - 2024 Jan 15

TI - Trap-Door Thoracotomy and Clamshell Thoracotomy as Surgical Approaches for

Neuroblastoma and Other Thoracic Tumors in Children.

LID - 10.3390/cancers16020373 [doi]

LID - 373

AB - Solid tumors of the cervicothoracic junction, the posterior mediastinum, or

bilateral dorsal thoracic tumors represent a challenge in pediatric surgical

oncology. The aim of this study was to evaluate trap-door thoracotomy and

clamshell thoracotomy as surgical approaches. A single-center retrospective study

of children with solid tumors in these specific localizations was performed. From

2015 to 2023, 26 children (17 girls; 9 boys) were treated at a median age of 54

months (range 8-229). Tumor resection was performed for neuroblastoma (n = 11);

metastatic disease (n = 7); malignant rhabdoid tumor (n = 4); Ewing sarcoma (n =

1); inflammatory myofibroblastic tumor (n = 1); rhabdomyosarcoma (n = 1); and

neurofibroma (n = 1). The surgical goal of macroscopic complete excision was

achieved in all of the 14 children who underwent trap-door thoracotomy and in 11

of the 12 children who underwent clamshell thoracotomy. There were no major

complications. At a median follow-up of 8 months (range 0-60), the disease was

under local control or in complete remission in 66.7% of the children. In

conclusion, surgical resection of solid tumors of the cervicothoracic junction in

children can be performed safely and successfully with trap-door thoracotomy and

with clamshell thoracotomy for posterior mediastinal or bilateral dorsal thoracic

tumors.

FAU - Mayer, Benjamin F B

AU - Mayer BFB

AUID- ORCID: 0000-0002-1025-3496

AD - Department of Pediatric Surgery and Pediatric Urology, University Children's

Hospital Tübingen, Hoppe-Seyler Straße 3, 72076 Tübingen, Germany.

FAU - Schunn, Matthias C

AU - Schunn MC

AD - Department of Pediatric Surgery and Pediatric Urology, University Children's

Hospital Tübingen, Hoppe-Seyler Straße 3, 72076 Tübingen, Germany.

FAU - Urla, Cristian

AU - Urla C

AUID- ORCID: 0000-0001-8909-0484

AD - Department of Pediatric Surgery and Pediatric Urology, University Children's

Hospital Tübingen, Hoppe-Seyler Straße 3, 72076 Tübingen, Germany.

FAU - Schäfer, Jürgen F

AU - Schäfer JF

AUID- ORCID: 0000-0003-3279-8726

AD - Division of Pediatric Radiology, Department of Diagnostic Radiology, University

Hospital Tübingen, Hoppe-Seyler Straße 1, 72076 Tübingen, Germany.

FAU - Fideler, Frank

AU - Fideler F

AUID- ORCID: 0000-0002-8667-8992

AD - Department of Anesthesiology and Intensive Care Medicine, University Hospital

Tübingen, Hoppe-Seyler Straße 1, 72076 Tübingen, Germany.
[truncated: 4,830,299 more chars]
