# Supplementary material for: Inflammatory myofibroblastic tumors of the colon in pediatrics: clinical presentation, management, and outcomes—A case report and systematic review of literature
Source: Int J Colorectal Dis. 2025 Apr 15;40(1):94. doi: 10.1007/s00384-025-04869-y (PMC12000112; doi:10.1007/s00384-025-04869-y)
Supplement: Supplementary file 4 — Supplementary file4 (DOCX 87 KB) [file 384_2025_4869_MOESM4_ESM.docx]

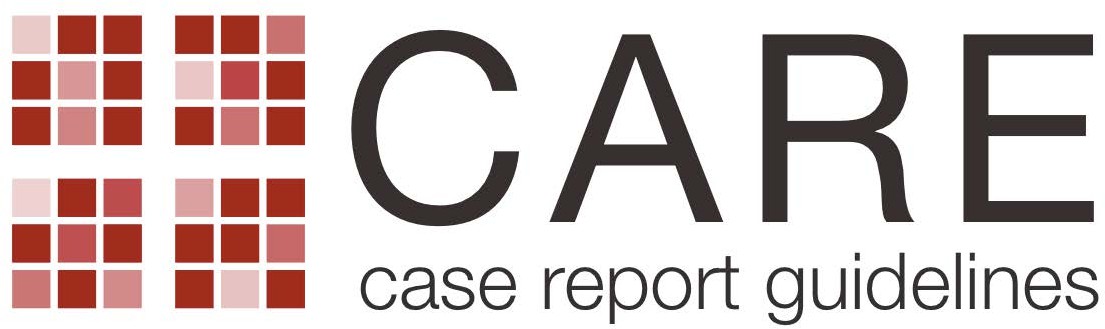
CARE Checklist of information to include when writing a case report
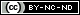


**Topic Item Checklist item description Reported on Line**

**Title 1** The diagnosis or intervention of primary focus followed by the words “case report” . . . . . . . . . . . . . . . . . . Page 1

**Key Words 2** 2 to 5 key words that identify diagnoses or interventions in this case report, including "case report" Page 2

**Abstract**

**(no references)**

**3a** Introduction: What is unique about this case and what does it add to the scientific literature? Page 2

**3b** Main symptoms and/or important clinical findings . . . . . . . . . . . . . . . . . . . . . . . . . . . . . . . . . . . . . . . . . . . . . . . . . . . Page 2

**3c** The main diagnoses, therapeutic interventions, and outcomes Page 2

**3d** Conclusion—What is the main “take-away” lesson(s) from this case? Page 2

**Introduction 4** One or two paragraphs summarizing why this case is unique (**may include** reference**s**) Page 3

**Patient Information 5a** De-identified patient specific information Pages 5,6

**5b** Primary concerns and symptoms of the patient Pages 5,6

**5c** Medical, family, and psycho-social history including relevant genetic information Pages 5,6

**5d** Relevant past interventions with outcomes Pages 5,6

**Clinical Findings**

**Timeline**

**Diagnostic Assessment**

**Therapeutic Intervention**

**Follow-up and Outcomes**

1. Describe significant physical examination (PE) and important clinical findings Pages 5,6
2. Historical and current information from this episode of care organized as a timeline Pages 5,6

**8a** Diagnostic testing (such as PE, laboratory testing, imaging, surveys). Pages 5,6

**8b** Diagnostic challenges (such as access to testing, financial, or cultural) Pages 5,6

**8c** Diagnosis (including other diagnoses considered) Page 6

**8d** Prognosis (such as staging in oncology) where applicable Page 6

**9a** Types of therapeutic intervention (such as pharmacologic, surgical, preventive, self-care) . . . . . . . . . . . . . . . . . Page 6

**9b** Administration of therapeutic intervention (such as dosage, strength, duration) Page 6

**9c** Changes in therapeutic intervention (with rationale) N/A

**10a** Clinician and patient-assessed outcomes (if available) Page 6

**10b** Important follow-up diagnostic and other test results Page 6

**10c** Intervention adherence and tolerability (How was this assessed?) Page 6

**10d** Adverse and unanticipated events Page 6

**Discussion 11a** A scientific discussion of the strengths AND limitations associated with this case report Pages 8,9,10,11,12

**11b** Discussion of the relevant medical literature **with references** Pages 8,9,10,11,12

**11c** The scientific rationale for any conclusions (including assessment of possible causes) Pages 8,9,10,11,12

**11d** The primary “take-away” lessons of this case report (without references) in a one paragraph conclusion Pages 12

**Patient Perspective 12** The patient should share their perspective in one to two paragraphs on the treatment(s) they received . . . . N/A

**Informed Consent 13** Did the patient give informed consent? Please provide if requested . . . . . . . . . . . . . . . . . . . . . . . . . . . . . . . . . . . . . . **Yes
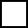
** **No
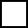
**
